# Substrate‐Controlled Enantiodivergence in Ni‐Catalyzed Access to Phosphorylated Oxindoles With Quaternary Stereocenters

**DOI:** 10.1002/anie.4623924

**Published:** 2026-06-07

**Authors:** Haimeng Zhu, Lewen Wang, Shihui Luo, Xinglong Zhang, Jun (Joelle) Wang

**Affiliations:** ^1^ Department of Chemistry Hong Kong Baptist University Hong Kong China; ^2^ Department of Chemistry The Chinese University of Hong Kong Hong Kong China

**Keywords:** asymmetric synthesis, cascade reactions, heck reactions, homogeneous catalysis, nickel

## Abstract

A Ni‐catalyzed enantioselective intermolecular Heck‐phosphorylation of N‐aryl acrylamides with phosphine oxides is developed. This redox‐neutral cascade simultaneously forges C─C and C─P bonds, providing direct access to valuable 3,3‐disubstituted phosphorylated oxindoles with quaternary stereocenters in good yields and high ee values. Notably, the reaction exhibits a unique substrate‐controlled enantiodivergence: simply changing the leaving group (I vs. OTf) on the alkene partner switches the configuration of the product while employing the same enantiomer of the chiral catalyst. The synthetic utility is further demonstrated through product derivatizations. Density functional theory (DFT) calculations reveal that the leaving group dictates the order of migratory insertion versus anion exchange and, crucially, inverts the chiral environment at the enantioselectivity‐determining oxidative addition transition state, providing a clear rationale for the observed stereodivergence.

## Graphical abstract

An efficient, enantiodivergent synthesis of phosphorylated oxindoles with quaternary stereocenters is achieved via a nickel‐catalyzed intermolecular Heck‐phosphorylation cascade. Employing a single chiral catalyst, either product enantiomer is selectively accessed simply by changing the alkene leaving group. DFT calculations trace this stereodivergence to leaving‐group‐controlled chiral environments at the enantioselectivity‐determining transition state.

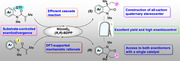

## 1 Introduction

Organophosphorus compounds are of significant importance in pharmaceuticals, agrochemistry, organic synthesis, and functional materials [1, 2, 3, 4, 5, 6, 7]. Among these, phosphine oxides are particularly valuable motifs; their incorporation into drug candidates is known to enhance biological activity, metabolic stability, and aqueous solubility [8, 9]. Concurrently, chiral 3,3‐disubstituted oxindoles constitute a privileged structural motif in numerous pharmaceuticals and natural products [10, 11, 12, 13]. Therefore, the development of enantioselective methods for introducing phosphine oxides into oxindole frameworks is highly desirable, offering a direct strategy to access novel, three‐dimensional chemical space for drug discovery and diversification.

**SCHEME 1:**
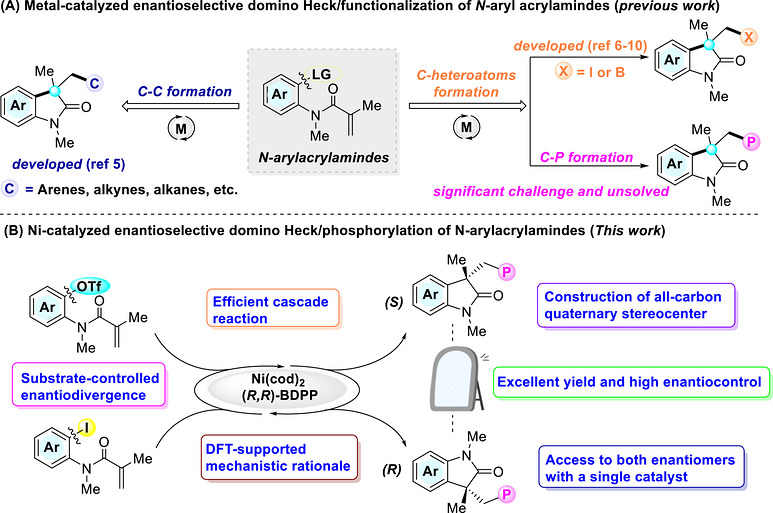
Transition metal catalyzed enantioselective heck/cyclization of tethered alkenes.

The asymmetric cascade Heck reaction has emerged as a powerful and atom‐economical strategy for constructing heterocycles with challenging all‐carbon quaternary stereocenters [14, 15, 16, 17, 18, 19, 20, 21, 22, 23, 24, 25, 26, 27, 28, 29]. In particular, enantioselective Heck/cyclization cascades of anilide‐tethered alkenes have been developed, enabling C–C bond formations alongside the introduction of diverse functional groups (Scheme 1A) [30, 31, 32, 33, 34, 35, 36, 37, 38, 39, 40, 41, 42, 43]. However, methodologies that forge both a C─C and a C─X (X = heteroatom) bond in a single catalytic operation to directly access chiral heteroatom‐substituted oxindoles remain very limited (Scheme 1A). Notable progress includes enantioselective intramolecular C─I bond formation. Lautens pioneered the nickel‐catalyzed asymmetric intramolecular Heck reaction in 2018, affording 3,3‐disubstituted iodooxindoles, albeit with a limited substrate scope [44]. Then in 2020, Lautens and Glorius reported enantioselective nickel‐catalyzed carbamoyl iodination by employing a 1,1‐disubstituted styrene and KI, thereby expanding the substrate scope [45]. For the C─B bond formation, Lautens [46], Wang [47], and Lu [48] independently developed metal‐catalyzed asymmetric borylation/cyclization sequences to access borylated heterocycles. Despite these advances, the direct enantioselective construction of other C–heteroatom bonds, particularly C─P bonds, via a Heck‐type cyclization remains an unmet challenge. While racemic synthesis of phosphorylated oxindoles exists [49, 50, 51, 52, 53, 54, 55], the competitive coordination of phosphorus species to the metal centers often complicates catalytic cycles and erodes the enantioselectivity.

A distinct and compelling challenge in asymmetric catalysis is achieving enantiodivergence, the ability to access either enantiomer of the products from the same chiral catalyst. This is especially valuable in drug discovery, where enantiomers can exhibit vastly different biological activities [56, 57, 58, 59, 60, 61, 62, 63, 64]. In Heck chemistry, such control is rare and typically requires altering the chiral ligand [31]. A strategy to invert enantioselectivity by simply modulating an achiral component of the substrate, while retaining the same chiral catalyst, represents a sophisticated yet undeveloped approach in this field.

Building upon our group's experience in transition metal‐catalyzed C─P bond formation [65, 66, 67, 68, 69, 70, 71, 72, 73, 74, 75, 76, 77, 78]. and the construction of all‐carbon quaternary stereocenters [79], we hypothesized the Ni‐catalyzed enantioselective intermolecular Heck‐phosphorylation of N‐aryl acrylamides with phosphine oxides to merge these domains. While Ni‐catalyzed enantioselective reductive Heck reactions typically proceed through a Ni(I)/Ni(III) redox manifold requiring stoichiometric reductants [20, 31, 32, 33, 34, 35, 36, 80, 81, 82], established Ni(0)‐catalyzed asymmetric C─P couplings operate via a Ni(0)/Ni(II) cycle [83, 84, 85, 86]. In light of these factors, the Ni(0)‐catalyzed redox‐neutral pathway could enable an intermolecular Heck phosphorylation cascade without an external reductant (Scheme 1B). This method directly converts N‐aryl acrylamides and phosphine oxides into valuable phosphorylated oxindoles bearing quaternary stereocenters in excellent yields and with high enantioselectivity. A particularly powerful feature of this system is its enantiodivergent nature: simply changing the leaving group on the alkene substrate, while employing the same chiral catalyst, provides selective access to either enantiomer of the product.

## 2 Results and Discussion

We commenced our study with *o*‐iodoaryl anilide (**1a**) and Ph_2_P(O)H catalyzed by Ni(cod)_2_ in DMF (Table 1). An initial ligand screen revealed that the (*R*,*R*)‐**BDPP** (**L3**) was the most effective, affording the expected product **3a** with 50% yield in 75% ee (Table 1, entry 3). The enantioselectivity was significantly increased to 85% with an identical yield by employing Li_2_CO_3_ as the base, while lower ees were obtained by K_2_CO_3_, Na_2_CO_3_, or Na_3_PO_4_ (Table 1, entries 7–9). Further optimization by lowering the reaction temperature and extending the time increased the yield to 90% while maintaining high enantioselectivity (84% ee, entry 10). Notably, switching the substrate to aryl triflate (**2a**) inverted the configuration of the product and provided 80% ee (entry 13), clearly demonstrating the feasibility of substrate‐controlled enantiodivergence.

**TABLE 1:** Optimization of reaction conditions.^a^

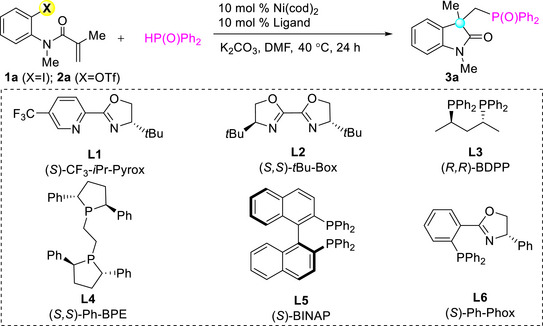						
Entry	X	Ligand	Sol.	Base	Yield (%)^b^	*ee* (%)^c^
1	I	L1	DMF	K_2_CO_3_	—	—
2	I	L2	DMF	K_2_CO_3_	31	13(*R*)
3	I	L3	DMF	K_2_CO_3_	50	75(*R*)
4	I	L4	DMF	K_2_CO_3_	21	60(*R*)
5	I	L5	DMF	K_2_CO_3_	—	—
6	I	L6	DMF	K_2_CO_3_	49	20(*R*)
7	I	L3	DMF	Na_2_CO_3_	55	76(*R*)
8	I	L3	DMF	Na_3_PO_4_	51	75(*R*)
9	I	L3	DMF	Li_2_CO_3_	48	85(*R*)
**10** ^d^	**I**	**L3**	**DMF**	**Li_2_CO_3_**	**94(90)** ^f^	**84(*R*)**
11	Br	L3	DMF	K_2_CO_3_	31	12(*R*)
12	OTs	L3	DMF	K_2_CO_3_	—	—
13	OTf	L3	DMF	K_2_CO_3_	49	80(*S*)
14	OTf	L3	DMA	K_2_CO_3_	39	65(*S*)
15	OTf	L3	CH_3_CN	K_2_CO_3_	95	70(*S*)
16	OTf	L3	DMSO	K_2_CO_3_	70	78(*S*)
17^e^	OTf	L3	DMSO	K_2_CO_3_	70	83(*S*)
18^e^	OTf	L3	DMSO	Na_2_CO_3_	90	82(*S*)
**19** ^e^	**OTf**	**L3**	**DMSO**	**Na_3_PO_4_**	**97(94)** ^f^	**83(*S*)**
20^e^	OTf	L3	DMSO	Et_3_N	54	79(*S*)
21^e^	OTf	L3	DMSO	TMG	17	80(*S*)

^a^ Conditions: **1a** (0.1 mmol), **2a** (0.15 mmol), Ni(cod)_2_ (10 mol %), Ligand (10 mol %), Base (2 equiv.), Sol. (1 mL), reaction at 40°C for 24 h.

^b^ Yields were determined by ^1^H NMR analysis.

^c^ Determined by chiral HPLC analysis.

^d^ Reaction at 35°C for 6 days.

^e^ Reaction at rt for 48 h.

^f^ Isolated yield.

Based on these results, conditions were further optimized for the reaction of triflate substrate (**2a**) and Ph_2_P(O)H. A solvent evaluation identified DMSO as optimal, affording **3a** in high yield (entry 16). Lowering the temperature and extending the time to 48 h increased the ee to 83% (entry 17). Subsequent base screening showed that Na_3_PO_4_ provided an excellent 94% yield while maintaining high enantiocontrol (entries 18–21). Thus, the optimal reaction conditions for aryl iodides were established as Ni(cod)_2_ (10 mol%), **L3** (10 mol%), and Li_2_CO_3_ (2 equiv.) in dry DMF at 35°C for 6 days; for aryl triflates, the optimal conditions were Ni(cod)_2_ (10 mol%), **L3** (10 mol%), and Na_3_PO_4_ (2 equiv) in dry DMSO at room temperature for 48 h.

With the optimized conditions in hand, the substrate scope was then investigated and is shown in Table 2. A series of aryl triflates with electron‐donating groups (e.g., Me, OMe, *t*Amyl and *t*Bu) at the 6‐position of the phenyl rings proceeded well, providing products (**3b**‐**3e**) in 97%–99% yields with 84–90% ees. Yet, the phenyl group (**3f**) and fluoro group (**3g**) at the 6‐position induced a slightly lower ee value (70–73% ee). 5‐Methyl substituted acrylamide **1h** with diphenylphosphine oxide, also worked well, affording product **3h** in 98% yield with 89% ee. Aryl triflates bearing electron‐withdrawing groups at the 5‐position, such as F and Cl groups, also led to diminished enantioselectivities (99% yield and 79% ee for both **3i** and **3j**). Substrate **1k**, which has a 7‐methyl group on the phenyl ring, exhibited excellent enantioselectivity and yield (99% yield, 94% ee). Subsequently, various organophosphorus reagents were examined. Gratifyingly, diethyl phosphite was also compatible and furnished the expected product **3l** with 78% yield and 84% ee. A wide range of diarylphosphine oxides bearing either electron‐donating (Me, OMe, Ph, NMe_2_, *t*Bu) or an electron‐withdrawing group (F) proceeded smoothly, affording chiral phosphorylated oxindoles in good yields with consistently excellent enantioselectivities ranging from 90% to 94% ees (**3m**‐**3x**). The absolute configuration of product **3u** was confirmed as (*S*) by single‐crystal x‐ray structure analysis [87]. Meanwhile, different substituents (‐Et, ‐Ph, and ‐Bn) were also introduced at the quaternary carbon center, with none affording better results than the methyl group (see Supporting Information for details).

**TABLE 2:** Scope of (S)‐phosphorylated oxindoles.^a^

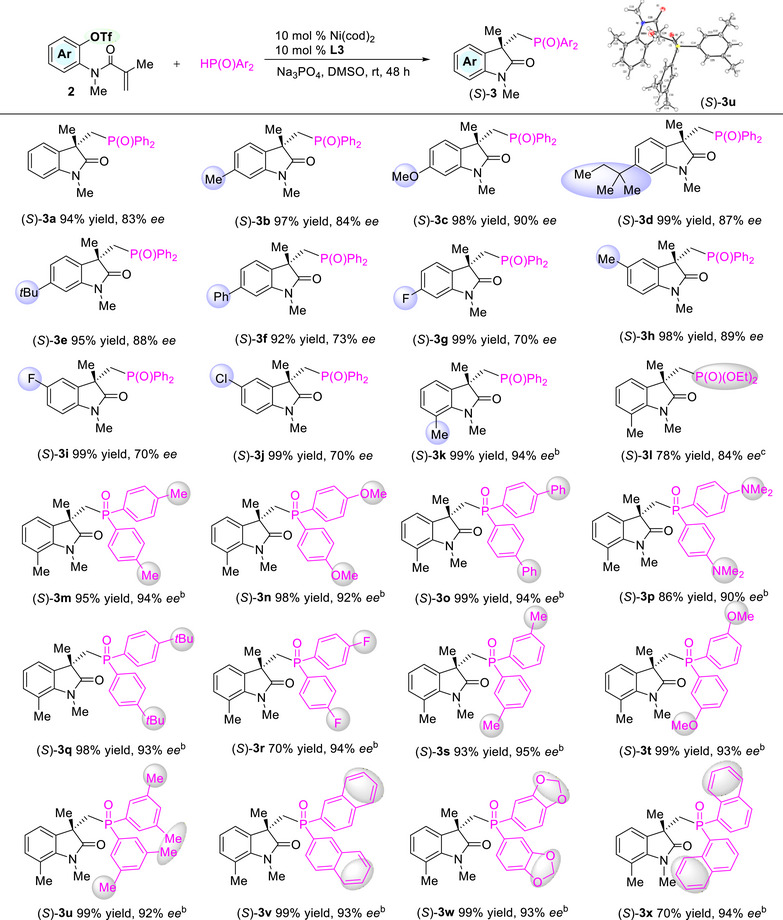

^a^ Reaction conditions: **2a** (0.1 mmol), HP(O)Ar_2_ (0.15 mmol), Ni(cod)_2_ (10 mol %), **L3** (10 mol %), Na_3_PO_4_ (2 equiv), DMSO (1 mL), reaction at rt for 48 h under argon; Isolated yields of compounds **3**; ee values are determined by HPLC. ^b^Reacted at rt for 4 days. ^c^Reacted at 55°C for 5 days.

Next, we systematically demonstrated that enantiodivergence could be generalized by simply varying the leaving group from OTf to I while employing the same enantiomer of the BDPP ligand and Ni(cod)_2_ catalyst (Table 3). Aryl iodides reacted efficiently to give (*R*)‐enantiomers of 3,3‐disubstituted oxindoles with good yield and high enantioselectivities. Significantly, aryl iodides bearing electron‐withdrawing groups on the phenyl ring exhibited remarkably high reactivity and enantioselectivity, but the ee values of (*S*) enantiomers were attenuated (**3g**,**3i,** and **3j**). The reaction scope was further extended to various diarylphosphine oxides, which reliably provided both the (*R*)‐ and (*S*)‐enantiomers (**3ba–3ha**) in high yields and with good enantioselectivity in each series. The absolute configuration of the (*R*)‐enantiomer derived from an iodide substrate was unambiguously assigned as (*R*) for product **3j** via single‐crystal x‐ray diffraction analysis [87]. We also attempted an iodide substrate bearing *β*‑substituents and the synthesis of six‑membered rings under our standard reaction conditions, yet none furnished satisfactory results (see Supporting Information for details).

**TABLE 3:** Enantiodivergent Synthesis of chiral phosphorylated oxindoles.^a^

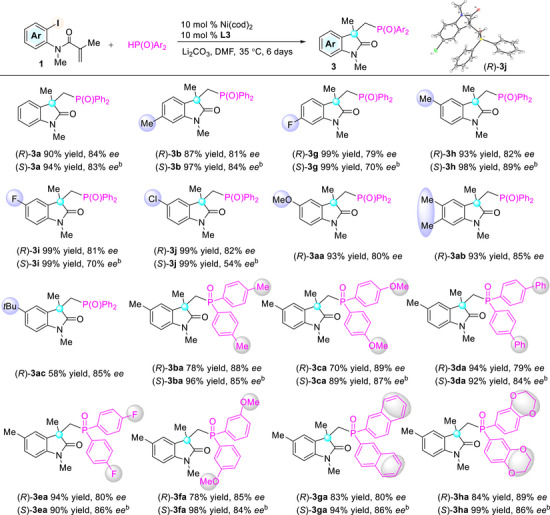

^a^ Reaction conditions: **1** (0.1 mmol), HP(O)Ar_2_ (0.15 mmol), Ni(cod)_2_ (10 mol%), **L3** (10 mol%), Li_2_CO_3_ (2 equiv), DMF (1 mL), reaction at 35°C for 6 days under argon; Isolated yields of compounds **3**; ee values are determined by HPLC. ^b^ **2 (X = OTf) were used instead of 1 (X = I)** (0.1 mmol), HP(O)Ar_2_ (0.15 mmol), Ni(cod)_2_ (10 mol %), **L3** (10 mol%), Na_3_PO_4_ (2 equiv), DMSO (1 mL), reaction at rt for 48 h under argon.

**SCHEME 2:**
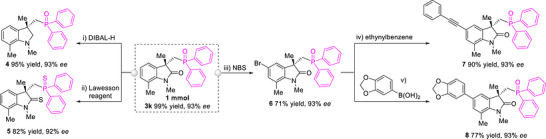
Reaction scale‐up and product derivatization: (A) 1mmol‐scale synthesis and synthetic transformations (B) enantioselective aldol reaction. Transformations of chiral product **3k**. i) DIBAL‐H (8 equiv), toluene, −78°C, 6 h. ii) Lawesson's reagent (1.1 equiv.), toluene, reflux, 12 h. iii) NBS (1.3 equiv.), CH_3_CN, rt, 12 h. iv) ethynylbenzene (1.5 equiv), PPh_3_(0.2 equiv.), potassium phosphate (1.2 equiv), Pd(OAc)_2_ (0.05 equiv), DMSO, 100°C, 24 h. v) boronic acid (2 equiv.), Pd_2_(dba)_3_ (0.05 equiv.), PCy_3_ (0.1 equiv.), potassium carbonate (2 equiv.), toluene, 100°C, 20 h.

The scale‐up reaction was performed on the 1 mmol scale, and product **3k** was obtained in 99% yield and 93% ee (Scheme 2A). With compound **3k**, it is feasible to construct a variety of structurally diverse phosphorylated oxindoles. Reduction of **3k** with DIBAL‐H yielded enantioenriched oxindole **4** in 95% yield with retention of the enantiopurity. **3k** was readily converted into the thioamide **5** in 82% yield and 92% ee by reaction with Lawesson's reagent. Furthermore, selective bromination at the C5 position of **3k** proceeded smoothly, affording product **6** in 71% yield with 93% ee. The bromination product **6** was further transformed by Sonogashira or Suzuki‐Miyaura coupling reaction, respectively, to produce the desired product **7** or **8** without erosion of stereochemical integrity.

**SCHEME 3:**
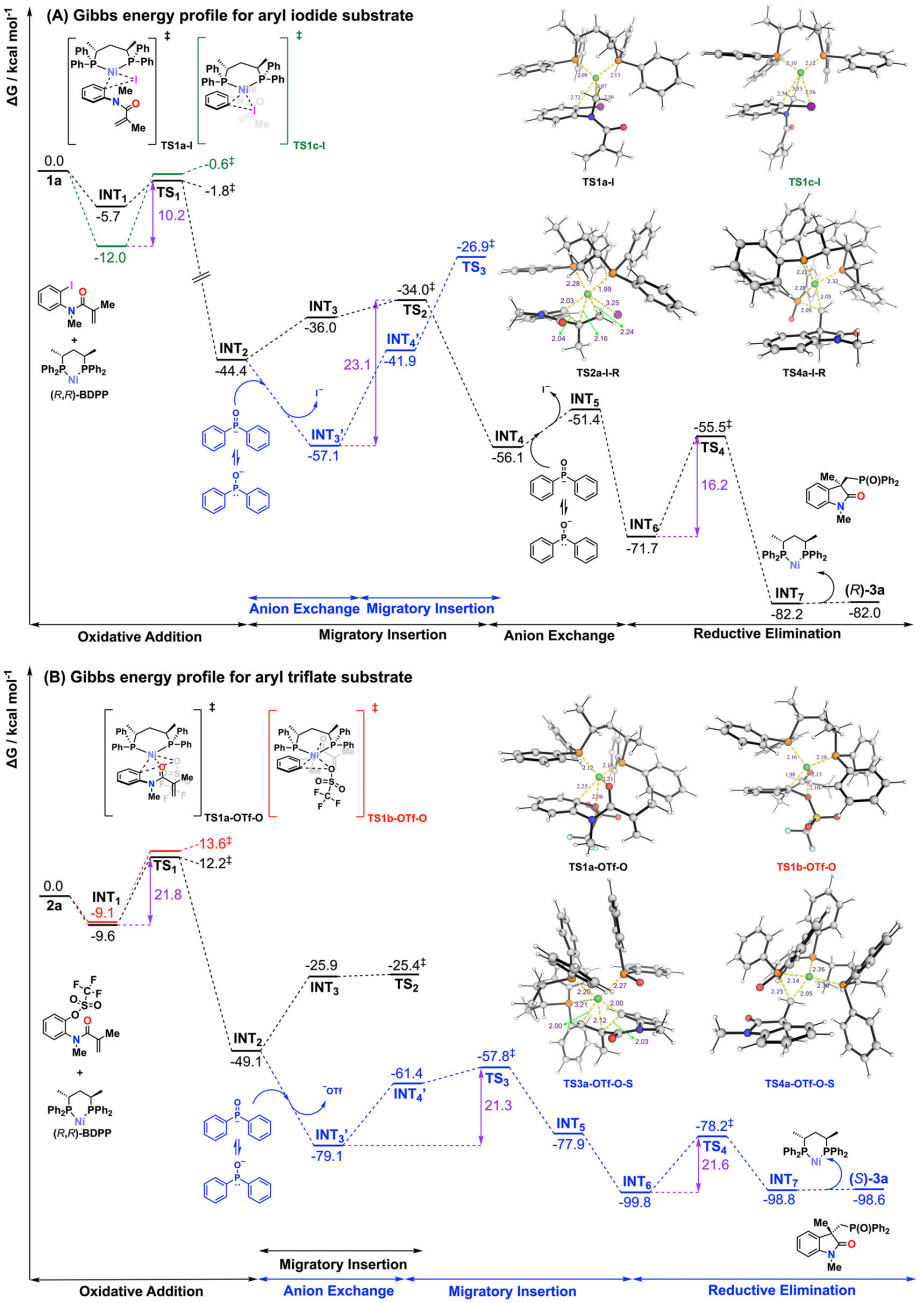
Gibbs energy profiles for model reactions: (A) using aryl iodide and (B) using aryl triflate as starting material.

To understand the reaction mechanism involving different leaving groups and the factors determining product chirality, density functional theory (DFT) calculations were performed (see Supporting Information for details). The calculated Gibbs energy profiles are shown in Scheme 3. We first determine if the Heck cyclization occurs before or after anion exchange. For the reaction using aryl iodide as the starting material (Scheme 3A), the Heck cyclization transition state after anion exchange (**TS3**) required overcoming a barrier of approximately 30.2 kcal·mol^−^^1^ (from **INT3′**), whereas the cyclization‐first pathway (**TS2**) required only 23.1 kcal·mol^−^^1^ (from **INT3′**, Scheme 3A). This energy difference indicates that, in the case of aryl iodide substrates, the preferred mechanism proceeds through oxidative addition, followed by Heck cyclization to establish the chiral center, then by anion exchange to replace the leaving group with phosphine oxide, and finally reductive elimination to yield the product.

In contrast, for the reaction employing aryl triflate as the starting material (Scheme 3B), the mechanism sequence is reversed. Here, the reaction favors anion exchange prior to Heck cyclization, as the transition state for migratory insertion prior to anion exchange (**TS2**) exhibits a barrier that is 32.4 kcal·mol^−^^1^ higher than that of the pathway involving anion exchange followed by migratory insertion (**TS3**). Thus, for aryl triflate, the preferred mechanism proceeds through oxidative addition, followed by anion exchange that replaces the leaving group with a deprotonated phosphine oxide, and then Heck cyclization to establish the chiral center, and finally reductive elimination to afford the product.

For further analysis on enantioselectivity, the enantioselectivity‐determining step should be decided first. The Ni(0) oxidative addition process in both reactions is highly exergonic; different isomeric insertions have been fully considered (Figures S2 and S8). A downhill Gibbs energy change is observed from the transition state (**TS1**) to the insertion intermediate (**INT2**). For the reaction using aryl iodide, the energy drop from **TS1** to **INT2** is approximately 42.6 kcal·mol^−^^1^, whereas for the aryl triflate reaction, this value is about 61.3 kcal·mol^−^^1^. These results indicate that the oxidative addition step is irreversible, as reverting from **INT2** to **INT1** via **TS1** will incur very high barriers, as shown in Scheme 3. Furthermore, due to steric hindrance, the isomerization between oxidative addition intermediates (e.g., **INT2a‐I** and **INT2c‐I**; see Supplementary Information for structural details) is prohibited. The steric repulsion between the phenyl groups of BDPP and the aryl ring of the substrate favors a similar relative orientation of the BDPP–Ni fragment with respect to the substrate in both pathways; in addition, the acrylamide side chain can only approach the reactive site from a single direction to undergo migratory insertion. Consequently, once the oxidative addition proceeds through specific **TS1**, the chirality of the final product is already determined. Therefore, we regard the oxidative addition step as the enantioselectivity‐determining step of the reaction; similar conclusions have been arrived in previous research [34].

We next analyzed the molecular origins underlying the enantioselectivity for the experimentally observed single chiral product formation. The DFT‐optimized structures, frontier molecular orbitals (HOMO and LUMO), and non‐covalent interaction (NCI) plots of the main competing transition states (**TS1a‐I** and **TS1c‐I** for reaction involving aryl iodide, **TS1a‐OTf‐O** and **TS1b‐OTf‐O** for reaction involving aryl triflate) are shown in Figure S3 (aryl iodide) and Figure S9 (aryl triflate), distortion‐interaction analysis are shown in Table S1 (aryl iodide) and Table S2 (aryl triflate). For **TS1a‐I** and **TS1c‐I**, the frontier molecular orbital structures and NCI plots are similar, distortion‐interaction analysis reveals that **TS1a‐I** has a lower distortion energy (by 1.3 kcal·mol^−^^1^), while having a similar interaction energy as **TS1c‐I**, therefore **TS1a‐I** is favored over **TS1c‐I** due to lower steric clashes. For the **TS1a‐OTf‐O** and **TS1b‐OTf‐O** pair, **TS1a‐OTf‐O** exhibits a significantly lower distortion energy in **TS1a‐OTf‐O** than in **TS1b‐OTf‐O** by 10.6 kcal·mol^−1^, despite it having less favorable interaction (by 9.5 kcal·mol^−1^) such that overall **TS1a‐OTf‐O** is favored over **TS1b‐OTf‐O**. In summary, our DFT calculations indicate that the leaving‐group identity controls the preferred oxidative‐addition geometry. In the aryl iodide pathway, amide O → Ni coordination is disfavored because the gain in interaction energy cannot compensate for the much higher distortion energy, making the non‐coordinated **TS1a‐I** lowest in energy (Table S1). By contrast, in the aryl triflate pathway, the oxygen‐coordinated **TS1a‐OTf‐O** is slightly favored (Table S2). Thus, triflate promotes an oxygen‐coordinated oxidative‐addition mode, whereas iodide does not, and this change in coordination environment, together with the overall balance between steric and electronic influences arising from ligand‐catalyst‐substrate orientations, leads to the observed enantiodivergence.

Theoretical enantiomeric excess (ee) values for these two model reactions were calculated (see Sections 6.3.4 and 6.4.4 in Supplementary Information). For the reaction using aryl iodide as a starting material at 35°C, by comparing the barriers of **TS1a‐I** and **TS1c‐I**, the ratio of rate constants for forming the *R*‐product and *S*‐product can be estimated as k[*R*]/k[*S*] ≈ 7.1, corresponding to an 87.7% proportion of the *R*‐enantiomer and a calculated ee of approximately 75%. As for the reaction using aryl triflate as starting material at 25°C, by comparing the barriers of **TS1a‐OTf‐O** and **TS1b‐OTf‐O**, the ratio of rate constants for forming the *R*‐product and *S*‐product can be estimated as k[*R*]/k[*S*] ≈ 0.094, corresponding to an *S*‐enantiomer ratio of approximately 91.4% and a calculated ee value of about 83%. The calculated ee values are in good agreement with experimental results.

**SCHEME 4:**
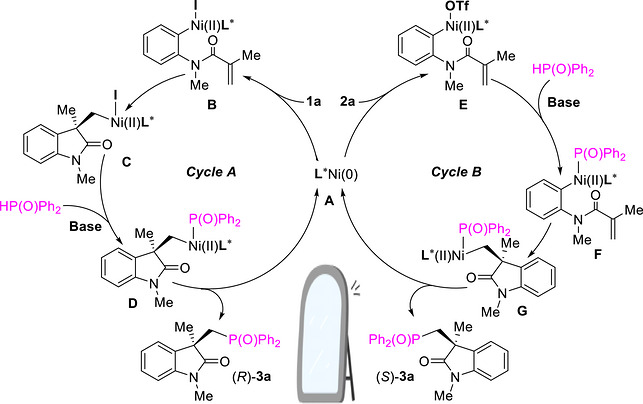
Proposed mechanistic cycles.

Based on previous reports [30, 31, 32, 33, 34, 35, 36, 37, 38, 39, 40, 41, 42, 43] and our computational results, two distinct catalytic cycles, denoted as cycle A and cycle B, corresponding to the reaction pathways for substrates **1a** and **2a**, respectively, are proposed (Scheme 4). Both cycles start from chiral nickel complex **A**, which is formed from Ni(cod)_2_ and (*R,R*)‐BDPP. As for cycle A, the oxidative addition of C–I bonds to L*Ni(0) gave the Ni(II) specie **B**, followed by an enantioselective 5‐exo‐trig cyclization to produce intermediate **C**. Then a ligand exchange generates the Ni(II)–P species **D**, accelerated by the base in the presence of the HP(O)Ph_2_. Further reductive elimination yields the chiral phosphorylated oxindoles (*R*)‐**3a** and regenerates reactive L*Ni(0) **A**. Although substrate **2a** in Cycle B similarly first undergoes oxidative addition to form intermediate **E**, DFT calculations reveal that **E** subsequently undergoes a ligand exchange, leading to the formation of intermediate **F**. Following the stereoselective 5‐exo‐trig intramolecular carbonickelation/migratory insertion, an alkyl‐L*Ni(II) intermediate **G** is formed, which can undergo reductive elimination to yield the desired product (*S*)‐**3a** and regenerate **A**.

## 3 Conclusion

In summary, we have developed an unprecedented Ni‐catalyzed enantioselective Heck‐phosphorylation cascade between phosphine oxides and anilide‐tethered alkenes. This redox‐neutral, intermolecular process provides efficient and straightforward access to enantioenriched phosphorylated oxindoles bearing quaternary stereocenters in high yields and excellent enantioselectivities. The methodology features a practical enantiodivergent capability: simply changing the leaving group (I vs. OTf) on the alkene substrate, while using the same chiral catalyst. The synthetic utility was demonstrated through a gram‐scale reaction and versatile downstream transformations of the oxindole products. DFT calculations established that the oxidative addition is enantioselectivity‐determining and revealed that the divergent stereochemical outcomes originate from leaving‐group‐controlled chiral environments at this key transition state, providing a clear mechanistic rationale for the observed substrate‐driven enantiodivergence.

## Author Contributions

**Haimeng Zhu**: methodology, writing – original draft, conceptualization, and investigation. **Lewen Wang**: writing – original draft, validation, and investigation. **Shihui Luo**: methodology and investigation. **Xinglong Zhang**: funding acquisition, supervision, resources, and writing – review and editing. **Jun (Joelle) Wang**: funding acquisition, conceptualization, project administration, writing – review and editing, and supervision.

## Conflicts of Interest

The authors declare no conflicts of interest.

## Supporting information




**Supporting File**: anie73012‐sup‐0001‐SuppMat.docx.

## Data Availability

The data that support the findings of this study are available in the supplementary material of this article. DFT optimized structures have been deposited and uploaded to https://zenodo.org/records/17636365 and are freely available.
