# Supplementary material for: Substrate‐Controlled Enantiodivergence in Ni‐Catalyzed Access to Phosphorylated Oxindoles With Quaternary Stereocenters
Source: Angew Chem Int Ed Engl. 2026 Jun 7;65(32):e4623924. doi: 10.1002/anie.4623924 (PMC13427142; doi:10.1002/anie.4623924)
Supplement: Supplementary file 1 — Supporting File: anie73012‐sup‐0001‐SuppMat.docx. [file ANIE-65-e4623924-s001.docx]

**Supplemental information**

**Substrate-Controlled Enantiodivergence in Ni-Catalyzed Access to Phosphorylated Oxindoles with Quaternary Stereocenters**

*Haimeng Zhu,^1§^ Lewen Wang,* *^1,2§^ Shihui Luo,^1^ Xinglong Zhang^2^*and Jun (Joelle) Wang^1^**

^1^Department of Chemistry, Hong Kong Baptist University, Kowloon, Hong Kong, China

^2^Department of Chemistry, The Chinese University of Hong Kong, Shatin, N.T., Hong Kong, China

**Supplemental Experimental Procedures**

**Table of contents**

[1. General information. 1](#_Toc227747027)

[2. General procedure for the synthesis of aryl iodides or triflates 2](#_Toc227747028)

[3. General procedure for the asymmetric synthesis of Phosphorylated Oxindoles 6](#_Toc227747029)

[4. Scale-up Reactions 7](#_Toc227747030)

[5. Synthetic Transformations 8](#_Toc227747031)

[6. Supplementary Experiments 14](#_Toc227747032)

[7. DFT calculations 25](#_Toc227747033)

[8. X-Ray crystallographic analysis. 61](#_Toc227747052)

[9. Characterization of products 64](#_Toc227747053)

[10. NMR spectrum 91](#_Toc227747054)

[11. HPLC spectrum 156](#_Toc227747055)

## 1. General information.

NMR Spectra were recorded on a Bruker DPX-500 (400) spectrometer at 600 MHz or

400 MHz for ^1^H NMR, 160 MHz for ^31^P NMR, 376 MHz for ^19^F NMR and 101 MHz or 125 MHz for ^13^C NMR in CDCl_3_ with tetramethylsilane (TMS) or the residual deuterated solvent peaks as internal standard. Chemical shifts (*δ*) are reported in ppm, and coupling constants (J) are in Hertz (Hz). Flash column chromatograph was carried out using 200-300 mesh silica gel at medium pressure. High resolution mass spectra (HRMS) were recorded on a LC-TOF spectrometer. ESI-HRMS data were acquired using a Thermo LTQ Orbitrap XL Instrument equipped with an ESI source. Optical rotation was obtained on a Rudolph Research Analytical (Atopol I). HPLC analysis was performed on Agilent 1260 series. Unless otherwise noted, all reagents were purchased from commercial suppliers and used without purification. All air- and moisture-sensitive manipulations were carried out with standard Schlenk techniques under nitrogen or in a glove box under argon. Anhydrous toluene was distilled from sodium benzophenone prior to use.

## 2. General procedure for the synthesis of aryl iodides or triflates

Aryl iodides (**1a**-**1b**, **1g**-**1j**, **1aa** and **1ac**)^1,2^ and aryl triflates (**2a**-**2c**, **2e**-**2i** and **2k**-**2n**)^3,4^ were prepared according to the literature procedures. The rest of aryl iodides and aryl triflates were prepared as below.

**2.1 General procedure for the preparation of the aryl iodide (1ab)**

Reaction conditions

Under a N_2_ atmosphere, a solution of substituted 2-iodo-4,5-dimethylaniline (1 equiv, 10 mmol) and triethylamine (2 equiv) in 20 mL dichloromethane was prepared and cooled to 0 °C. To this cooled solution, a solution of methacryloyl chloride (1.5 equiv) in 10 mL dichloromethane was added dropwise. After complete addition, the reaction mixture was stirred at 0 °C for 30 minutes before being allowed to warm to room temperature. The reaction was stirred overnight at room temperature. The reaction was then quenched by the addition of saturated aqueous sodium bicarbonate solution. The mixture was extracted with dichloromethane three times. The combined organic extracts were washed with brine, dried over sodium sulfate, filtered, and concentrated under reduced pressure to provide crude amide which was used without further purification.

This crude amide was dissolved in 40 mL tetrahydrofuran and cooled to 0 °C. Sodium hydride (60% dispersion in mineral oil, 2 equiv.) was added in portions to this cooled solution. After the addition was complete, the mixture was stirred for 20 minutes at 0 °C. Iodomethane was then added dropwise to the reaction mixture. After complete addition, the reaction was allowed to warm to room temperature and stirred for 2 hours. The reaction was quenched by the addition of water. The resulting mixture was extracted with ethyl acetate three times. The combined organic extracts were washed with brine, dried over sodium sulfate, filtered, and concentrated under reduced pressure. The crude residue was purified by flash chromatography on silica gel using a mixture of petroleum ether and ethyl acetate as eluent to afford the desired N-(2-iodo-4,5-dimethylphenyl)-N-methylmethacrylamide product.

**N-(2-iodo-4,5-dimethylphenyl)-N-methylmethacrylamide (1ab)**

**^1^H NMR** (600 MHz, Chloroform-*d*) δ 7.60 (s, 1H), 6.92 (s, 1H), 5.06 (s, 1H), 4.96 (s, 1H), 3.19 (s, 3H), 2.19 (d, *J* = 11.1 Hz, 6H), 1.82 (s, 3H).

**^13^C NMR** (151 MHz, Chloroform-*d*) δ 171.86, 144.46, 140.50, 140.26, 138.43, 138.29, 130.08, 118.70, 94.93, 36.88, 20.67, 19.42, 18.94.

**2.2 General procedure for the synthesis of aryl triflates (2d and 2j)**

Reaction conditions

To a 10 mL DMF containing 2-aminophenol (1 equiv, 10 mmol) and imidazole (1 equiv), a solution of TBSCl (1.1 equiv) in 5 mL DMF was introduced. The resulting mixture was allowed to react at ambient temperature for 12 hours. Upon completion of the reaction, as determined by TLC analysis, the mixture was treated with a saturated ammonium chloride aqueous solution and extracted with ethyl acetate. The collected organic phases were subsequently washed with brine, dried over anhydrous sodium sulfate, and filtered. Concentration of the filtrate under reduced pressure afforded a yellowish oily residue, which was directly employed in the subsequent synthetic step without additional purification.

A solution of TBS-protected aminophenol and triethylamine (2 equiv) in 20 mL dichloromethane was prepared and cooled to 0 °C. To this cooled mixture, a solution of acryloyl chloride (1.2 equiv) in 10 mL dichloromethane was added dropwise over 15 minutes. After the addition was complete, the reaction was stirred for an additional 30 minutes at 0 °C before being allowed to warm to room temperature. The mixture was stirred at room temperature until TLC analysis indicated complete consumption of the starting amine. The resulting solution was concentrated, and the residue was dissolved in EtOAc and filtered. The organic layer was washed with 5% HCl solution 3 times, saturated NaHCO_3_ solution and brine, dried over anhydrous Na_2_SO_4_, filtered, and concentrated in vacuo. The residue was used for the next step without further purification.

A suspension of sodium hydride (60% dispersion in mineral oil, 1.5 equiv) in 25 mL anhydrous tetrahydrofuran was prepared under an inert nitrogen atmosphere and cooled to 0 °C. To this cooled suspension, a solution of amide in 10 mL dry THF was introduced dropwise. The resulting mixture was stirred at room temperature for 30 minutes before being cooled to 0 °C. Subsequently, the MeI was added dropwise. The reaction was then allowed to proceed at room temperature overnight. Upon completion, as confirmed by TLC analysis, the reaction was carefully quenched with a saturated ammonium chloride solution at 0 °C. The aqueous mixture was extracted multiple times with ethyl acetate. The combined organic extracts were washed with water followed by brine, then dried over anhydrous sodium sulfate, filtered, and concentrated under reduced pressure. The crude material was purified by silica gel column chromatography to yield the desired N-alkylated product.

To a solution of N-alkylated compound in 25 mL tetrahydrofuran was introduced tetrabutylammonium fluoride (1.1 equiv). The reaction mixture was stirred at ambient temperature, with progress monitored by TLC until complete consumption of the starting material was observed. Upon reaction completion, the mixture was diluted with water and extracted with ethyl acetate. The combined organic extracts were washed with brine, dried over anhydrous sodium sulfate, and filtered. After removal of the solvent under reduced pressure, the resulting crude material was subjected to purification by silica gel column chromatography affording the desired phenol as a yellow solid.

A solution of phenol and pyridine (2 equiv) in 25 mL dichloromethane was prepared and maintained at 0 °C under a nitrogen atmosphere. To this cooled solution, triflic anhydride (1.5 equiv) was added dropwise. Upon completion of the addition, the reaction mixture was allowed to warm to room temperature and stirred for 2 hours. The reaction progress was monitored by TLC until completion was confirmed. The mixture was then poured into water and extracted with dichloromethane. The combined organic extracts were dried over sodium sulfate and concentrated under reduced pressure. The resulting crude material was purified by silica gel column chromatography to obtain aryl triflate product**s**.

**2-(N-methylmethacrylamido)-4-(tert-pentyl) phenyl trifluoromethanesulfonate (2d)**

**^1^H NMR** (600 MHz, Chloroform-*d*) δ 7.29 (dd, *J* = 8.7, 2.3 Hz, 1H), 7.25 – 7.20 (m, 2H), 5.01 (s, 1H), 4.81 (s, 1H), 3.34 (s, 3H), 1.83 (s, 3H), 1.63 (q, *J* = 7.4 Hz, 2H), 1.28 (s, 6H), 0.66 (t, *J* = 7.4 Hz, 3H).

**^13^C NMR** (151 MHz, Chloroform-*d*) δ 171.61, 151.34, 141.83, 139.93, 136.86, 127.17, 126.45, 121.76, 119.35, 118.45 (q, *J* = 320.4 Hz), 38.14, 37.28, 36.81, 28.22, 19.93, 8.95.

**^19^F NMR** (565 MHz, Chloroform-*d*) δ -73.86.

**5-chloro-2-(N-methylmethacrylamido) phenyl trifluoromethanesulfonate (2j)**

**^1^H NMR** (600 MHz, Chloroform-*d*) δ 7.43 – 7.335 (M, 1H), 7.35 – 7.26 (m, 2H), 5.12 (s, 1H), 4.79 (s, 1H), 3.33 (s, 3H), 1.90 (s, 3H).

**^19^F NMR** (565 MHz, Chloroform-*d*) δ -73.53.

**^13^C NMR** (101 MHz, Chloroform-*d*) δ 171.56, 144.36, 139.47, 136.19, 133.79, 129.72, 129.64, 123.06, 120.02, 118.43 (q, *J* = 320.3 Hz), 37.23, 19.79.

**2.3 References**

1. Xu, R.-R., Bao, X., Huo, Y.-W., Miao, R.-G., Wen, D., Dai, W., Qi, X., and Wu, X.-F. Palladium-Catalyzed Domino Carbopalladation/Carbonylative Cyclization: Synthesis of Heterocycles bearing Oxindoles and 3-Acylbenzofuran/3-Acylindole Moieties. *Org. Lett.* 24, 6477-6482 (2022).

2. Cheng, C., Xiang, J.-N., Zhu, Y.-P., Peng, Z.-H., and Li, J.-H. Nickel-Catalyzed Arylcarbamoylation of Alkenes of N-(o-Iodoaryl)acrylamides with Nitroarenes via Reductive Aminocarbonylation: Facile Synthesis of Carbamoyl-Substituted Oxindoles. *Org. Lett.* 23, 9543-9547 (2021).

3. Chen, Z., and Shen, Z. Nickel-catalyzed asymmetric reductive arylcyanation of alkenes with acetonitrile as the cyano source. *Org. Chem. Front*. 10, 745-751 (2023).

4. Li, Y., Wang, K., Ping, Y., Wang, Y., and Kong, W. Nickel-Catalyzed Domino Heck Cyclization/Suzuki Coupling for the Synthesis of 3,3-Disubstituted Oxindoles. *Org. Lett.* 20, 921-924 (2018).

## 3. General procedure for the asymmetric synthesis of Phosphorylated Oxindoles

**3.1 aryl iodide as starting materials**

In an argon-filled glovebox, a flame-dried screw-cap reaction tube equipped with a magnetic stir bar was charged with Ni(cod)_2_ (10 mol %) and **L3** (10 mol %). DMF (1 mL) was added, then the mixture was stirred at room temperature for 15 min. Secondary phosphine oxides (0.15 mmol, 1.5 equiv.), Li_2_CO_3_ (2 equiv) and aryl iodides (0.1 mmol, 1 equiv.) were added. The Schleck reaction vial was sealed with a rubber plug and taken out glovebox. The resulting solution was allowed to stir at the indicated temperature under argon atmosphere. After vacuum evaporation of the solvent, the residue was purified by silica gel column chromatography to provide the desired enantioenriched phosphorylated oxindoles.

**3.2 aryl triflates as starting materials**

In an argon-filled glovebox, a flame-dried screw-cap reaction tube equipped with a magnetic stir bar was charged with Ni(cod)_2_ (10 mol %) and **L3** (10 mol %). DMSO (1 mL) was added, then the mixture was stirred at room temperature for 15 min. Secondary phosphine oxides (0.15 mmol, 1.5 equiv.), Na_3_PO_4_ (2 equiv) and aryl triflates (0.1 mmol, 1 equiv.) were added. The Schleck reaction vial was sealed with a rubber plug and taken out glovebox. The resulting solution was allowed to stir at the indicated temperature under argon atmosphere. After vacuum evaporation of the solvent, the residue was purified by silica gel column chromatography to provide the desired enantioenriched phosphorylated oxindoles.

## 4. Scale-up Reactions

In an argon-filled glovebox, a flame-dried screw-cap reaction tube equipped with a magnetic stir bar was charged with Ni(cod)_2_ (0.15 mmol, 77.1 mg) and **L3** (0.165 mmol, 67.2 mg). DMSO (15 mL) was added, then the mixture was stirred at room temperature for 15 min. Secondary phosphine oxides (1.5 mmol, 1.5 equiv.), Na_3_PO_4_ (2 equiv) and **2k** (1 mmol, 1 equiv.) were added. The Schleck reaction vial was sealed with a rubber plug and taken out glovebox. The resulting solution was allowed to stir at room temperature under argon atmosphere for 5 days. After vacuum evaporation of the solvent, the residue was purified by silica gel column chromatography to to give product **3k** (mg, 98% yield, 93% *ee*).

## 5. Synthetic Transformations

A solution of compound **3k** (0.1 mmol, 1 equiv.) in toluene was cooled to -78 °C. To this solution, a solution of DIBAL-H (1.0 M in toluene, 2.4 mmol, 8 equiv) in toluene was added dropwise. The reaction mixture was maintained at -78 °C and stirred for 6 hours. After completion of the reaction, the mixture was carefully quenched with an aqueous sodium hydroxide solution. The resulting mixture was then extracted with ethyl acetate. The combined organic extracts were dried over sodium sulfate and concentrated under reduced pressure. The crude residue was purified by flash chromatography on silica gel to afford the desired product 4 as a colorless oil (mg, 95% yield, 93% ee).

**(*S*)-diphenyl((1,3,7-trimethylindolin-3-yl)methyl)phosphine oxide (4)**

Colorless oil; 95% yield, 93% ee; [α]^20^_D_= -18.6 (c = 1.7, CHCl_3_); HPLC analysis: Chiralcel IA column (hexane / 2-propanol 95:5, 1.0 mL/min, 254 nm); t_r_ (major) = 28.5 min, t_r_ (minor) = 31.7 min.

**HRMS (ESI) m/z**: calcd for C_24_H_26_NOP [M+H]^+^ 376.1830, found 376.1824.

**^1^H NMR** (400 MHz, Chloroform-*d*) δ 7.81 – 7.68 (m, 4H), 7.48 – 7.38 (m, 6H), 6.87 (dd, *J* = 7.4, 1.3 Hz, 1H), 6.80 (d, *J* = 7.4 Hz, 1H), 6.59 (t, *J* = 7.4 Hz, 1H), 3.60 (d, *J* = 9.7 Hz, 1H), 3.02 (d, *J* = 9.7 Hz, 1H), 2.88 (s, 3H), 2.74 – 2.58 (m, 2H), 2.34 (s, 3H), 1.38 (s, 3H).

**^13^C NMR** (101 MHz, Chloroform-*d*) δ 149.49, 139.35 (d, *J* = 12.1 Hz), 135.27 (d, *J* = 42.2 Hz), 134.30 (d, *J* = 41.5 Hz), 131.39 (d, *J* = 6.0 Hz), 131.35 (d, *J* = 7.1 Hz), 130.57 (d, *J* = 9.1 Hz), 130.36 (d, *J* = 9.2 Hz), 128.59 (d, *J* = 5.7 Hz), 128.56, 128.47 (d, *J* = 5.5 Hz), 120.23, 119.87, 119.07, 69.39 (d, *J* = 3.0 Hz), 42.75 (d, *J* = 4.1 Hz), 39.26, 39.18 (d, *J* = 68.0 Hz), 25.74 (d, *J* = 2.6 Hz), 19.60.

**^31^P NMR** (162 MHz, Chloroform-*d*) δ 28.45.

A mixture of compound **3k** (0.1 mmol, 1 equiv.) and Lawesson's reagent (0.11 mmol, 1.1 equiv.) in 1 mL toluene was heated under reflux with continuous stirring. After 12 hours, the reaction mixture was concentrated under reduced pressure. The resulting residue was purified by flash chromatography on silica gel to afford the desired product **5** as a colorless oil (mg, 82% yield, 92% ee).

**(*S*)-3-((diphenylphosphorothioyl)methyl)-1,3,7-trimethylindoline-2-thione (5)**

Colorless oil; 82% yield, 92% ee; [α]^20^_D_= -29.3 (c = 1.5, CHCl_3_); HPLC analysis: Chiralcel AD-H column (hexane / 2-propanol 90:10, 1.0 mL/min, 254 nm); t_r_ (minor) = 12.0 min, t_r_ (major) = 23.2 min.

**HRMS (ESI) m/z**: calcd for C_24_H_24_NPS_2_ [M+H]^+^ 422.1166, found 422.1162.

**^1^H NMR** (400 MHz, Chloroform-*d*) δ 7.87 – 7.74 (m, 2H), 7.56 – 7.32 (m, 6H), 7.28 – 7.24 (m, 2H), 6.94 (dt, *J* = 7.6, 1.1 Hz, 1H), 6.87 (dd, *J* = 7.5, 1.3 Hz, 1H), 6.70 (t, *J* = 7.5 Hz, 1H), 3.88 (dd, *J* = 14.7, 10.3 Hz, 1H), 3.72 (s, 3H), 3.23 (dd, *J* = 14.7, 11.0 Hz, 1H), 2.52 (s, 3H), 1.43 (d, *J* = 2.8 Hz, 3H).

**^13^C NMR** (101 MHz, Chloroform-*d*) δ 208.99 (d, *J* = 1.8 Hz), 143.00, 135.95 (d, *J* = 3.5 Hz), 133.14 (d, *J* = 17.2 Hz), 132.35 (d, *J* = 16.8 Hz), 132.01, 131.78 (d, *J* = 5.1 Hz), 131.68 (d, *J* = 4.9 Hz), 131.13 (d, *J* = 3.0 Hz), 130.82 (d, *J* = 2.9 Hz), 128.29 (d, *J* = 12.1 Hz), 127.82 (d, *J* = 12.2 Hz), 123.48, 123.35, 120.51, 55.60 (d, *J* = 3.3 Hz), 43.63 (d, *J* = 56.2 Hz), 35.78, 32.78 (d, *J* = 13.5 Hz), 19.95.

**^31^P NMR** (162 MHz, Chloroform-*d*) δ 35.79.

A solution of compound **3k** (0.1 mmol, 1 equiv.) in 2 mL acetonitrile was treated with N-bromosuccinimide (0.13 mmol, 1.3 equiv.) at room temperature. The reaction mixture was stirred for 12 hours, after which the solvent was removed under reduced pressure. The crude residue was purified by flash column chromatography on silica gel to afford the brominated product **6** as a white solid (mg, 71% yield, 93% ee).

**(*S*)-5-bromo-3-((diphenylphosphoryl)methyl)-1,3,7-trimethylindolin-2-one (6)**

Colorless oil; 71% yield, 93% ee; [α]^20^_D_= -46.7 (c = 3.3, CHCl_3_); HPLC analysis: Chiralcel IH column (hexane / 2-propanol 95:5, 1.0 mL/min, 254 nm); t_r_ (major) = 24.0 min, t_r_ (minor) = 32.2 min.

**HRMS (ESI) m/z**: calcd for C_24_H_23_BrNO_2_P [M+H]^+^ 468.0728, found 468.0724.

**^1^H NMR** (600 MHz, Chloroform-*d*) δ 7.61 – 7.53 (m, 2H), 7.47 – 7.41 (m, 4H), 7.39 – 7.30 (m, 4H), 7.01 – 6.92 (m, 1H), 6.78 (d, *J* = 2.0 Hz, 1H), 3.37 (s, 3H), 3.11 (dd, *J* = 15.1, 11.6 Hz, 1H), 2.74 (dd, *J* = 15.1, 8.6 Hz, 1H), 2.47 (s, 3H), 1.38 (d, *J* = 2.0 Hz, 3H).

**^13^C NMR** (151 MHz, Chloroform-*d*) δ 179.76, 140.44, 134.14, 133.77 (d, *J* = 2.6 Hz), 133.51 (d, *J* = 62.6 Hz), 132.85 (d, *J* = 63.1 Hz), 131.61 (d, *J* = 3.2 Hz), 131.59 (d, *J* = 3.8 Hz), 130.64 (d, *J* = 9.3 Hz), 130.37 (d, *J* = 9.4 Hz), 128.39, 128.31, 125.48, 121.44, 114.40, 44.90 (d, *J* = 3.8 Hz), 38.07 (d, *J* = 71.1 Hz), 29.90, 27.13 (d, *J* = 12.6 Hz), 18.75.

**^31^P NMR** (243 MHz, Chloroform-*d*) δ 25.57.

A reaction tube was charged with compound **6** (0.1 mmol, 1 equiv.), ethynylbenzene(0.15 mmol, 1.5 equiv), triphenylphosphine (0.02 mmol, 0.2 equiv), potassium phosphate (0.12 mmol, 1.2 equiv), palladium acetate (0.005 mmol, 0.05 equiv), and 1 mL dimethyl sulfoxide. The mixture was stirred at 100 °C under an argon atmosphere for 24 hours. After completion of the reaction, the mixture was diluted with ethyl acetate and washed sequentially with water and brine. The organic layer was dried over anhydrous sodium sulfate and concentrated under reduced pressure. The crude residue was purified by silica gel column chromatography to afford the coupled product **7** (mg, % yield, % ee).

**(*S*)-3-((diphenylphosphoryl)methyl)-1,3,7-trimethyl-5-(phenylethynyl)indolin-2-one (7)**

Colorless oil; 90% yield, 93% ee; [α]^20^_D_= -96.9 (c = 2.3, CHCl_3_); HPLC analysis: Chiralcel AD-H column (hexane / 2-propanol 70:30, 1.0 mL/min, 254 nm); t_r_ (minor) = 10.5 min, t_r_ (major) = 14.3 min.

**HRMS (ESI) m/z**: calcd for C_32_H_28_NO_2_P [M+H]^+^ 490.1936, found 490.1932.

**^1^H NMR** (400 MHz, Chloroform-*d*) δ 7.59 – 7.54 (m, 2H), 7.51 – 7.41 (m, 6H), 7.37 – 7.31 (m, 7H), 7.08 – 7.03 (m, 1H), 6.88 – 6.83 (m, 1H), 3.37 (s, 3H), 3.17 (dd, *J* = 15.1, 11.3 Hz, 1H), 2.81 (dd, *J* = 15.1, 9.2 Hz, 1H), 2.49 (s, 3H), 1.42 (d, *J* = 2.1 Hz, 3H).

**^13^C NMR** (101 MHz, Chloroform-*d*) δ 180.20 (d, *J* = 2.8 Hz), 141.36, 135.27, 133.29 (d, *J* = 27.8 Hz), 132.30 (d, *J* = 28.6 Hz), 131.86 (d, *J* = 2.8 Hz), 131.61 (d, *J* = 2.8 Hz), 131.55 (d, *J* = 3.2 Hz), 131.37, 130.81 (d, *J* = 9.6 Hz), 130.51 (d, *J* = 9.4 Hz), 128.40 (d, *J* = 5.9 Hz), 128.37, 128.28 (d, *J* = 5.7 Hz), 127.95, 125.63, 123.64, 119.68, 116.61, 89.51, 88.44, 44.63 (d, *J* = 3.9 Hz), 38.00 (d, *J* = 71.5 Hz), 29.89, 27.38 (d, *J* = 12.9 Hz), 18.90.

**^31^P NMR** (162 MHz, Chloroform-*d*) δ 26.82.

An oven-dried Schlenk tube equipped with a stir bar was charged with Pd_2_(dba)_3_ (0.005 mmol, 0.05 equiv.), PCy_3_ (0.01 mmol, 0.1 equiv.), and potassium carbonate (0.2 mmol, 2 equiv.) in 1 mL toluene under an argon atmosphere. The mixture was stirred at room temperature for 10 minutes. Subsequently, substrate **6** (0.1 mmol, 1 equiv.) and the boronic acid (0.2 mmol, 2 equiv.) were added, and the reaction mixture was heated to 80 °C for 20 hours. After completion, the solvent was removed under reduced pressure, and the crude residue was purified by flash chromatography on silica gel using a mixture of ethyl acetate and petroleum ether as eluent to afford the product **8** (mg, 77% yield, 93% ee).

**(*S*)-5-(benzo[d][1,3]dioxol-5-yl)-3-((diphenylphosphoryl)methyl)-1,3,7-trimethylindolin-2-one (8)**

Colorless oil; 77% yield, 93% ee; [α]^20^_D_= -90.5 (c = 1.5, CHCl_3_); HPLC analysis: Chiralcel AD-H column (hexane / 2-propanol 70:30, 1.0 mL/min, 254 nm); t_r_ (major) = 10.7 min, t_r_ (minor) = 12.6 min.

**HRMS (ESI) m/z**: calcd for C_31_H_28_NO_4_P [M+H]^+^ 510.1834, found 510.1831.

**^1^H NMR** (400 MHz, Chloroform-*d*) δ 7.56 – 7.38 (m, 5H), 7.32 – 7.26 (m, 5H), 7.09 – 6.97 (m, 2H), 6.88 – 6.76 (m, 3H), 5.97 (s, 2H), 3.33 (s, 3H), 3.21 (dd, *J* = 15.2, 10.4 Hz, 1H), 2.88 (dd, *J* = 15.2, 10.7 Hz, 1H), 2.49 (s, 3H), 1.44 (d, *J* = 2.0 Hz, 3H).

**^13^C NMR** (101 MHz, Chloroform-*d*) δ 180.31 (d, *J* = 2.9 Hz), 147.89, 146.62, 140.08, 134.95 (d, *J* = 6.3 Hz), 134.92, 133.32 (d, *J* = 50.8 Hz), 132.43 (d, *J* = 3.0 Hz), 132.33 (d, *J* = 49.9 Hz), 131.46 (d, *J* = 2.3 Hz), 131.43 (d, *J* = 2.6 Hz), 130.91 (d, *J* = 9.5 Hz), 130.59 (d, *J* = 9.4 Hz), 130.23, 128.31 (d, *J* = 11.9 Hz), 128.14 (d, *J* = 11.9 Hz), 121.29, 120.35, 119.68, 108.39, 107.44, 101.05, 44.96 (d, *J* = 3.8 Hz), 37.89 (d, *J* = 71.4 Hz), 29.83, 27.77 (d, *J* = 12.8 Hz), 19.08.

**^31^P NMR** (243 MHz, Chloroform-*d*) δ 26.70.

## 6. Supplementary Experiments

**6.1 Effects of Base and Solvent**

**6.2 Screening of Substituents at the Quaternary Center**


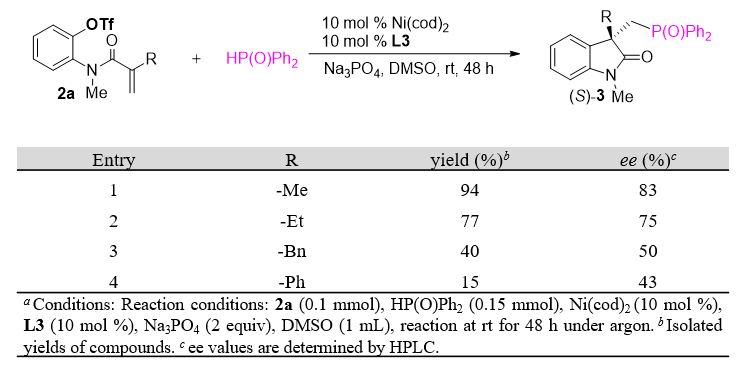


**(S)-3-((diphenylphosphoryl)methyl)-3-ethyl-1,5-dimethylindolin-2-one**

77% yield, 75% ee; Colorless oil; HPLC analysis: Chiralcel ID column (hexane / 2-propanol 50:50, 1.0 mL/min, 254 nm); t_r_ (major) = 13.5 min, t_r_ (minor) = 17.7 min. **^1^H NMR** (600 MHz, Chloroform-*d*) δ 7.53 (ddd, *J* = 11.3, 8.1, 1.5 Hz, 2H), 7.50 – 7.36 (m, 4H), 7.32 (dtd, *J* = 17.8, 7.8, 2.7 Hz, 4H), 7.16 (td, *J* = 7.7, 1.4 Hz, 1H), 7.03 (d, *J* = 7.3 Hz, 1H), 6.76 (t, *J* = 7.5 Hz, 1H), 6.64 (d, *J* = 7.8 Hz, 1H), 3.10 (dd, *J* = 15.2, 10.0 Hz, 1H), 2.98 (s, 3H), 2.84 (dd, *J* = 15.2, 11.3 Hz, 1H), 1.90 (p, *J* = 6.7 Hz, 2H), 0.51 (t, *J* = 7.3 Hz, 3H). **^13^C NMR** (101 MHz, Chloroform-*d*) δ 178.73, 143.99, 133.82 (d, *J* = 100.7 Hz), 133.23, 131.37, 131.23 (d, *J* = 2.6 Hz), 130.79 (d, *J* = 9.5 Hz), 130.47 (d, *J* = 9.1 Hz), 129.11 (d, *J* = 3.2 Hz), 128.39 (d, *J* = 11.7 Hz), 128.15 (d, *J* = 11.7 Hz), 127.97, 125.04, 122.07, 107.64, 49.92 (d, *J* = 3.9 Hz), 37.04 (d, *J* = 71.5 Hz), 33.67 (d, *J* = 12.2 Hz), 26.20, 7.95. **^31^P NMR** (240 MHz, Chloroform-*d*) δ 26.11. **HRMS (ESI) m/z**: calcd for C_24_H_24_KNO_2_P ^+^ [M+K]^+^ 428.1176, found 428.1182.

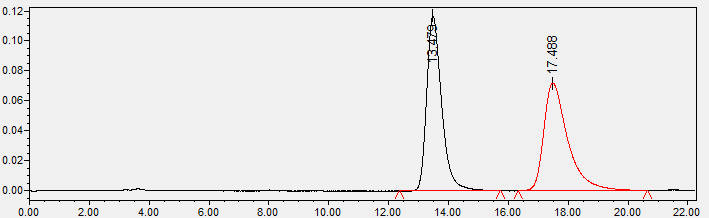

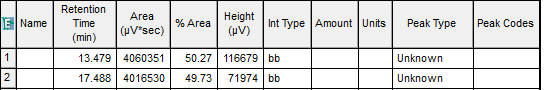


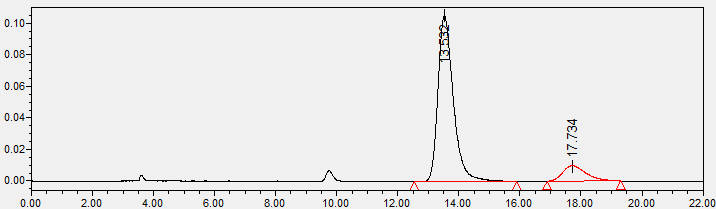

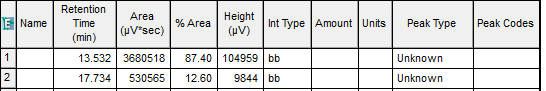


**(S)-3-benzyl-3-((diphenylphosphoryl)methyl)-1,5-dimethylindolin-2-one**

40% yield, 50% ee; Colorless oil; HPLC analysis: Chiralcel ID column (hexane / 2-propanol 50:50, 1.0 mL/min, 254 nm); t_r_ (major) = 14.2 min, t_r_ (minor) = 25.6 min. **^1^H NMR** (600 MHz, Chloroform-*d*) δ 7.65 – 7.58 (m, 2H), 7.50 – 7.44 (m, 3H), 7.43 – 7.34 (m, 3H), 7.34 – 7.28 (m, 2H), 7.16 – 6.98 (m, 5H), 6.81 (td, *J* = 7.5, 1.0 Hz, 1H), 6.77 – 6.72 (m, 2H), 6.38 (d, *J* = 7.8 Hz, 1H), 3.27 (dd, *J* = 15.2, 9.8 Hz, 1H), 3.20 – 3.06 (m, 2H), 3.01 (dd, *J* = 15.2, 11.8 Hz, 1H), 2.70 (s, 3H). **^13^C NMR** (151 MHz, Chloroform-*d*) δ 177.91 (d, *J* = 3.6 Hz), 143.53, 134.61, 133.70 (d, *J* = 99.6 Hz), 132.66 (d, *J* = 98.7 Hz), 131.39 (d, *J* = 2.7 Hz), 131.39 (d, *J* = 2.7 Hz), 130.93 (d, *J* = 9.7 Hz), 130.58 (d, *J* = 9.1 Hz), 130.02, 128.47 (d, *J* = 11.8 Hz), 128.45, 128.09 (d, *J* = 11.9 Hz), 128.08, 127.39, 126.70, 125.90, 121.72, 107.51, 51.03 (d, *J* = 3.7 Hz), 46.40 (d, *J* = 12.3 Hz), 36.48 (d, *J* = 71.2 Hz), 25.88. **^31^P NMR** (240 MHz, Chloroform-*d*) δ 26.31. **HRMS (ESI) m/z**: calcd for C_29_H_26_NNaO_2_P ^+^ [M+Na]^+^ 474.1593, found 474.1560.

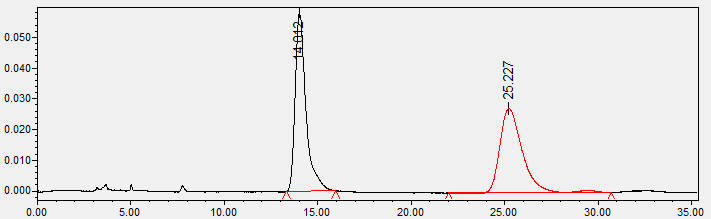

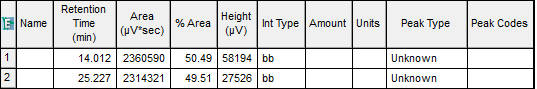


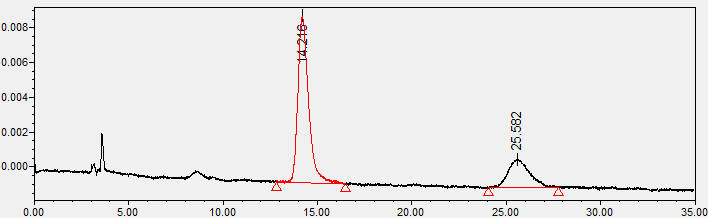

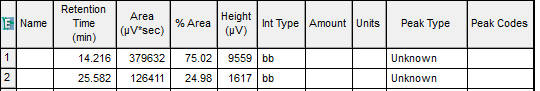


**(S)-3-((diphenylphosphoryl)methyl)-1-methyl-3-phenylindolin-2-one**

15% yield, 43% ee; Colorless oil; HPLC analysis: Chiralcel ID column (hexane / 2-propanol 50:50, 1.0 mL/min, 254 nm); t_r_ (minor) = 14.6 min, t_r_ (major) = 24.0 min. **^1^H NMR** (600 MHz, Chloroform-*d*) δ 7.58 – 7.52 (m, 2H), 7.50 – 7.39 (m, 4H), 7.39 – 7.26 (m, 6H), 7.26 – 7.14 (m, 4H), 7.06 – 6.95 (m, 1H), 6.78 – 6.65 (m, 2H), 3.72 (dd, *J* = 15.0, 11.0 Hz, 1H), 3.23 (dd, *J* = 15.0, 10.0 Hz, 1H), 3.01 (d, *J* = 10.4 Hz, 3H). **^13^C NMR** (101 MHz, Chloroform-*d*) δ 177.56 (d, *J* = 2.1 Hz), 144.22, 140.67 (d, *J* = 12.4 Hz), 133.54 (d, *J* = 43.9 Hz), 132.88 (d, *J* = 42.7 Hz), 131.52 (d, *J* = 2.7 Hz), 131.20 (d, *J* = 2.8 Hz), 130.82 (d, *J* = 9.3 Hz), 130.50 (d, *J* = 9.1 Hz), 129.01 (d, *J* = 3.4 Hz), 128.60, 128.45 (d, *J* = 13.6 Hz), 128.34 (d, *J* = 2.5 Hz), 128.27, 127.55, 126.91, 126.50, 121.96, 108.25, 52.62 (d, *J* = 3.0 Hz), 38.48 (d, *J* = 70.2 Hz), 26.72. **^31^P NMR** (240 MHz, Chloroform-*d*) δ 25.47. **HRMS (ESI) m/z**: calcd for C_28_H_24_KNO_2_P^+^ [M+K]^+^ 476.1176, found 476.1182.

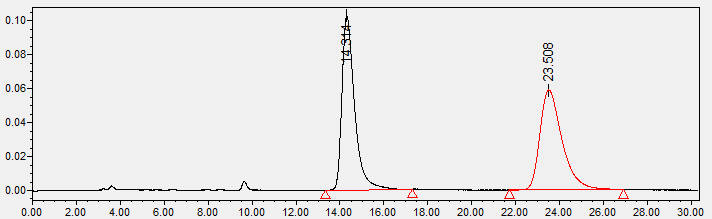

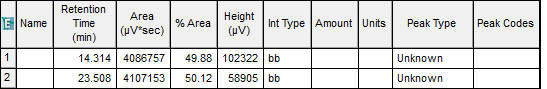


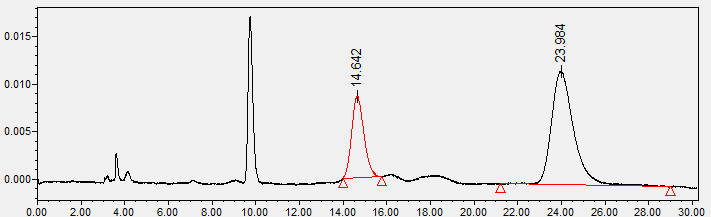

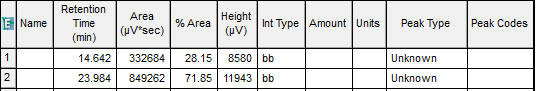


**6.3 Competition Experiments**

## 7. DFT calculations

**7.1 Computational Methods**

**7.1.1 Conformational sampling**

Grimme’s *CREST* program,^1,2^ which used meta-dynamics (MTD) with genetic z-matrix crossing (GC), was utilized to perform conformational sampling at the GFN2-xTB^3–5^ extended semiempirical tight-binding level of theory with ‘*--optlev tight’* setting. After the searching procedure, isolated conformers with lowest xTB energies were then collected for further density functional theory (DFT) calculations.

**7.1.2 Density Functional Theory (DFT) calculations**

Geometry optimizations were performed in the gas phase with Gaussian 16 rev. B.01 software,^6^ employing the global-hybrid meta-NGA (nonseparable gradient approximation) MN15 functional^7^ in combination with the def2-SVP^8,9^ basis set for all atoms. Harmonic frequency analysis was used to verify minima and transition structures on the potential energy surface (PES), which were characterized respectively by zero and one imaginary frequency. Quick reaction coordinate (QRC)^10^ calculations were carried out to verify the transition state structures.

To improve the accuracy of the corrected Gibbs energy profile, single point (SP) calculations on the gas phase optimized geometries were performed at MN15 with def2-QZVP^8,9^ basis set for all atoms in the implicit SMD solvation model^11^. The effect of solvent on the potential energy surface was modeled with the N,N-dimethylformamide model for the reaction analysis in which aryl iodide served as the starting material, whereas for the case involving aryl triflate as the starting material, the dimethyl sulfoxide model was adopted, in accordance with the experiments.

Gibbs energies were evaluated at the reaction temperature of 35 ºC for aryl iodide substrate and 25 ºC for aryl triflate substrate, respectively, using Grimme’s scheme of quasi-RRHO treatment of vibrational entropies^12^ (frequencies below 100 cm^-1^ were obtained) and Head-Gordon's quasi-RRHO treatment of vibrational enthalpies^13^ (frequencies below 100 cm^-1^ were obtained), using *Chemsmart* automatic computation framework.^14^

The final corrected Gibbs energy in SMD(N,N-dimethylformamide/dimethyl sulfoxide)-MN15/def2-QZVP//MN15/def2-SVP are shown in the figures. All Gibbs energy values in the text and figures are quoted in kcal·mol^-1^.

Non-covalent interaction(NCI) analysis was conducted using *NCIPLOT*^15^ version 4.2. The *.wfn* files for *NCIPLT* were generated at MN15/DGDZVP^16,17^ level of theory. Optimized structures, NCI plots and molecular orbitals were visualized using *PyMOL* software where the .*pse* files have been automatically generated using the *Chemsmart* toolkit.^14,18^

**7.2 Model Reactions7**

Scheme S1 shows the model reactions that we used for computational studies of the reaction mechanism. In experiment, for reaction using aryl iodide, target product was formed in 90% yield with 84% ee. On the other hand, target product, using aryl triflate as starting material, was formed in 94% yield with 83 ee.


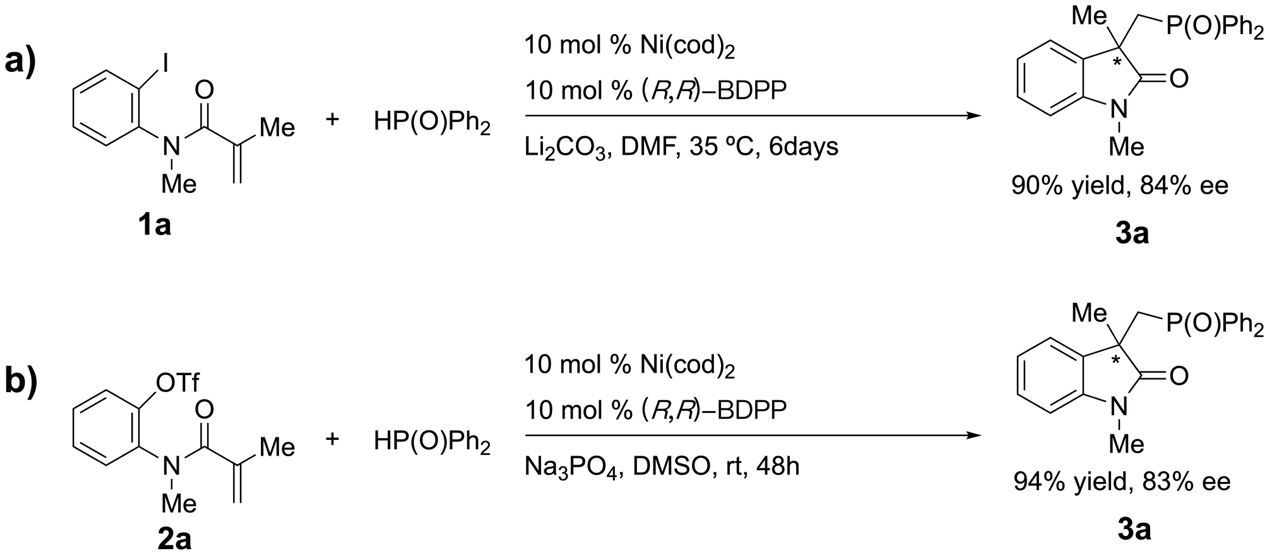


**Scheme S1.** Model reaction for computational studies, a) using aryl iodide as starting material and b) using aryl triflate as starting material.

One of the key issues to be addressed in the DFT calculations is the determination of the specific reaction pathway for the two proposed model reactions. We propose two possible mechanistic scenarios: in the first pathway, Heck cyclization occurs prior to anion exchange, in which the leaving group is subsequently replaced by a (deprotonated) phosphine oxide; in the second pathway, anion exchange precedes the Heck cyclization. These two pathways are illustrated in the main text.

**7.3 Aryl Iodide as Starting Material**

**7.3.1 Gibbs energy profile**

Conformation sampling was first conducted on the substrate and the Ni(0) model coordinated with ligand molecule, DFT geometry optimization and SP calculation were then performed to locate conformers with lowest energy of substrate, ligand and Ni(0)-ligand complex. Subsequently, transition state (TS) search was performed based on the lowest energy conformers. Once a transition state was located, QRC calculations were carried out to ensure proper connectivity between the adjacent stationary points on the potential energy surface.^10^ The Gibbs free energies of the reactants, intermediates, transition states, and products were obtained from thermochemistry calculations corrected by single-point energies with solvation effects. These energies together constitute the Gibbs free energy profile, shown in Figure S1.

As a note, in the following discussion, the TS corresponding to the oxidative addition step is denoted as **TS1**. For the pathway involving Heck cyclization prior to anion exchange, the TS of the migratory insertion step is labeled **TS2**. In contrast, for the pathway in which anion exchange precedes the cyclization, the TS of the migratory insertion step is labeled **TS3**. The TS of the reductive elimination step in both pathways are denoted as **TS4**.


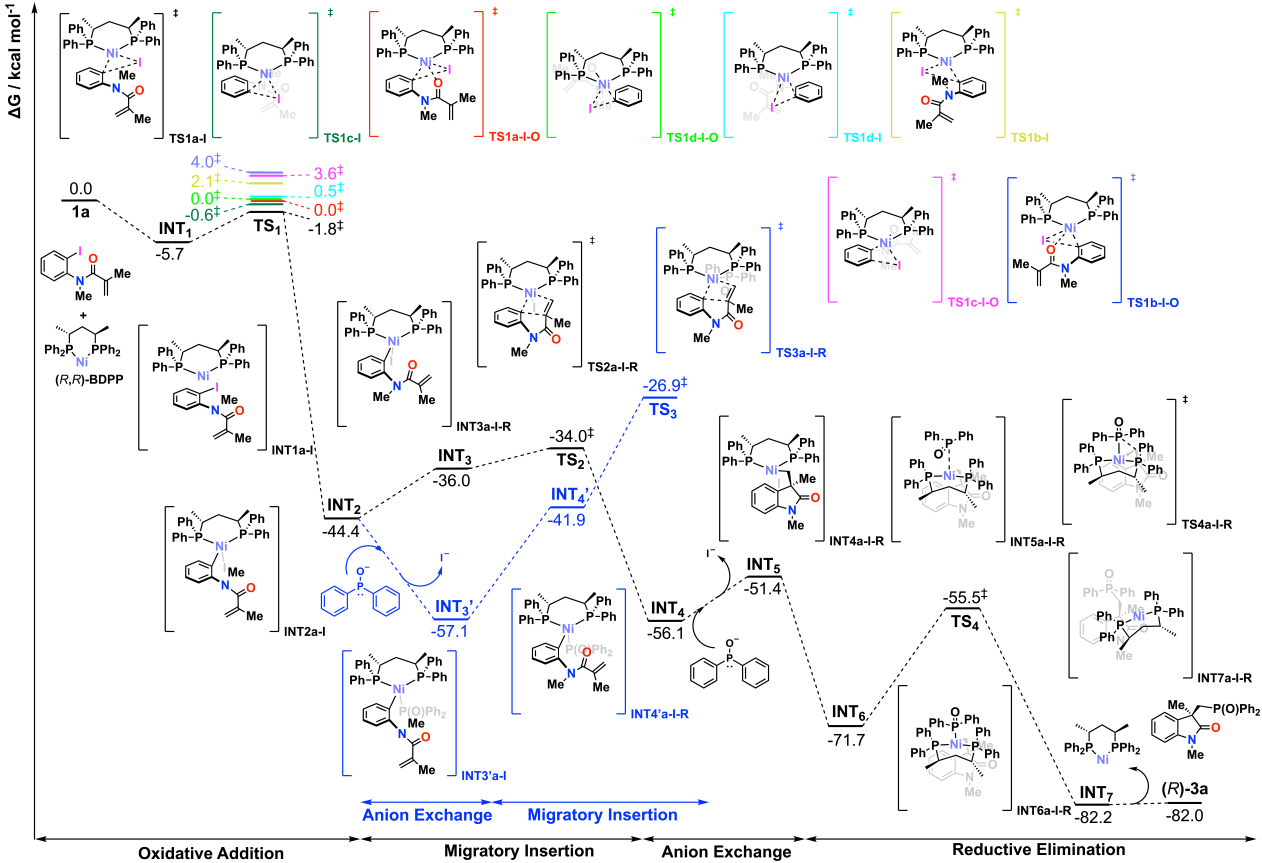


**Figure S1.** Gibbs energy profile for reaction using aryl iodide as starting material. Gibbs energies are given in SMD(N,N-dimethylformamide)-MN15/def2-QZVP//MN15/def2-SVP level of theories.

We located TSs with different ligand orientations for **TS1**. It is worth noting that the oxidative addition step is highly exergonic, with a downhill Gibbs energy change of 42.6 kcal·mol⁻¹, upon the formation of **INT2** after Ni insertion. Under the reaction temperature of 35 °C, this transformation was considered irreversible. This irreversibility had significant implications for the subsequent calculation of enantiomeric product ratios and for evaluating the competing transition states associated with **TS2**.

We analyzed the reaction pathway beginning from **INT2**. According to the energy profile, the pathway involving anion exchange prior to Heck cyclization (**TS3**) required overcoming a barrier of approximately 30.2 kcal·mol⁻¹, whereas the cyclization-first pathway (**TS2**) required only about 23.1 kcal·mol⁻¹. This significant energy difference indicated that, for reactions using aryl iodide as starting material, the preferred mechanism proceeded through oxidative addition, followed by Heck cyclization to establish the chiral center, anion exchange to replace the leaving group with phosphine oxide, and finally reductive elimination to yield the product.

**7.3.2 Competing transition states for oxidative addition step**

The structures of all 8 conformers of **TS1** are shown in Figure S2.

| **TS1a-I** | **TS1a-I-O** |
| --- | --- |
| ΔG^‡^ = -1.8 | ΔG^‡^ = 0.0 |
| 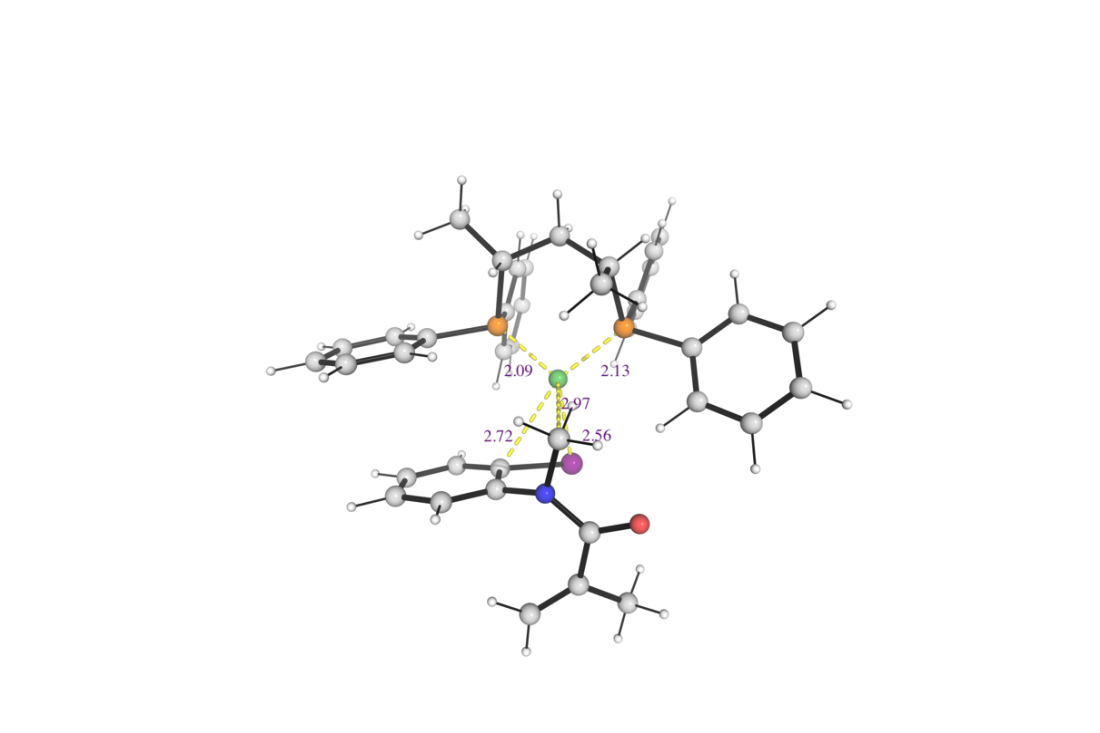 | 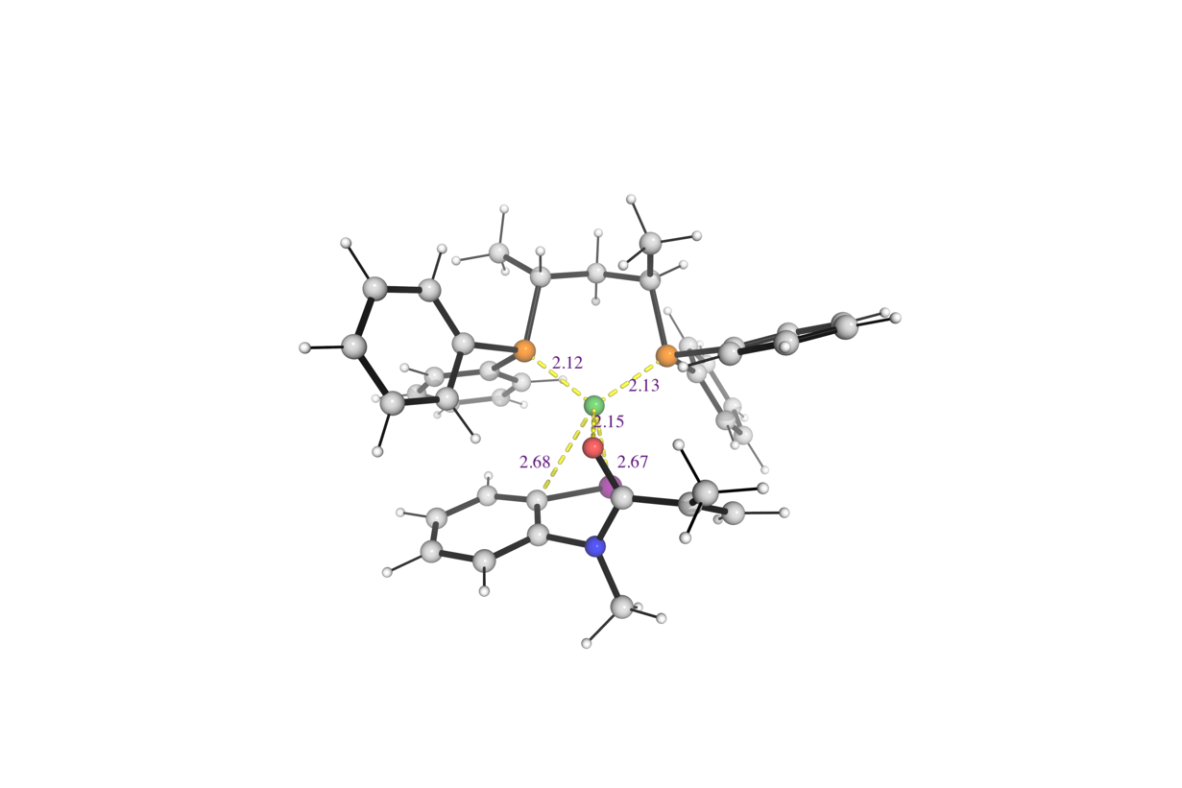 |
| **TS1b-I** | **TS1b-I-O** |
| ΔG^‡^ = 2.1 | ΔG^‡^ = 4.0 |
| 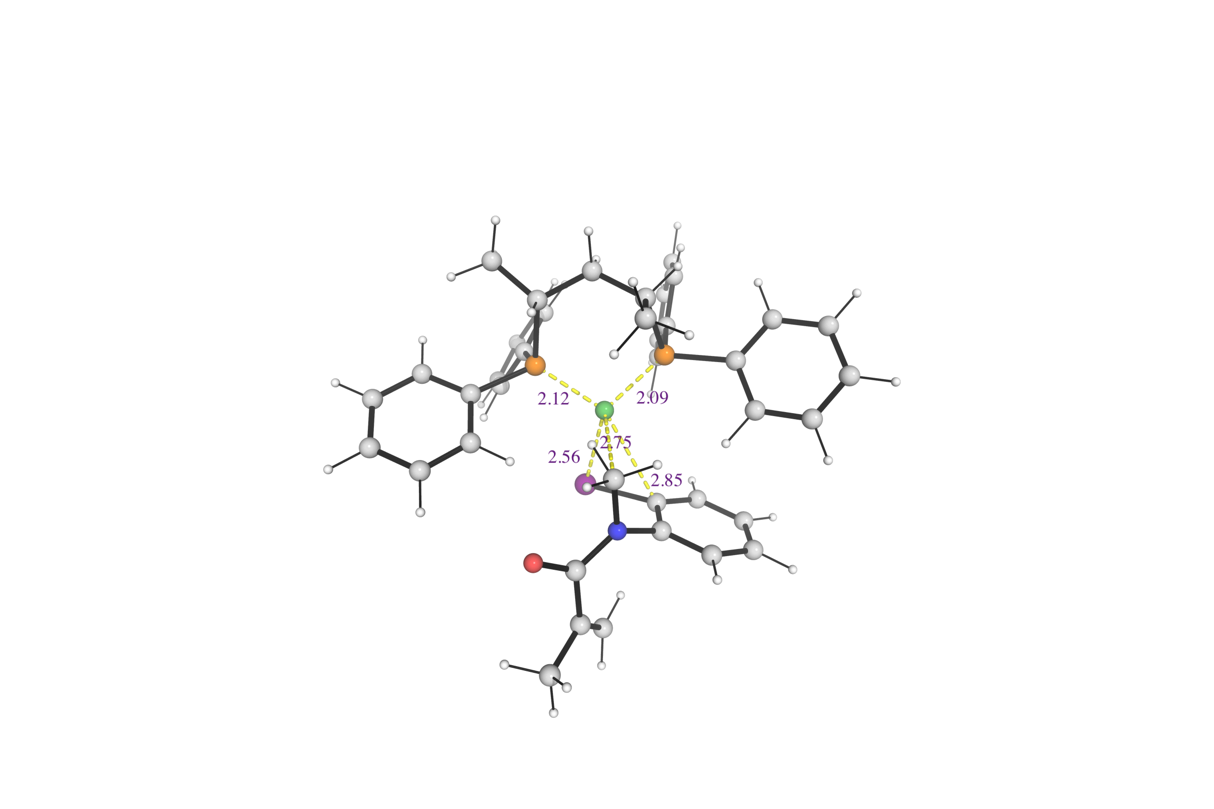 | 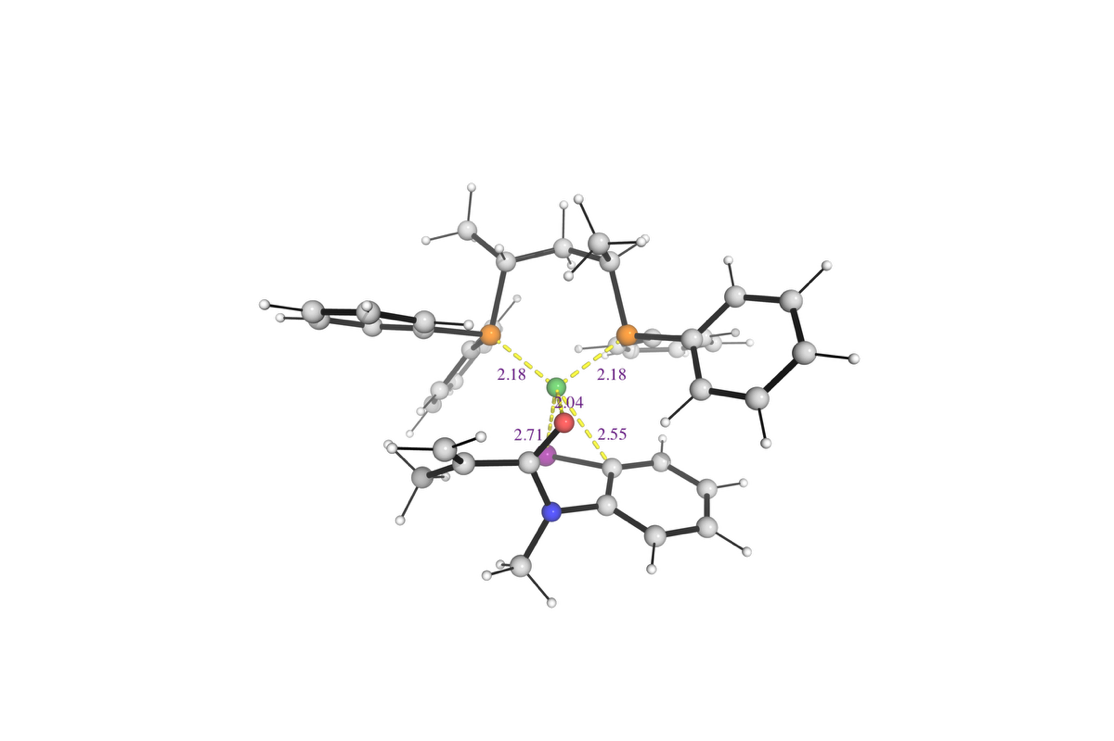 |
| **TS1c-I** | **TS1c-I-O** |
| ΔG^‡^ = -0.6 | ΔG^‡^ = 3.6 |
| 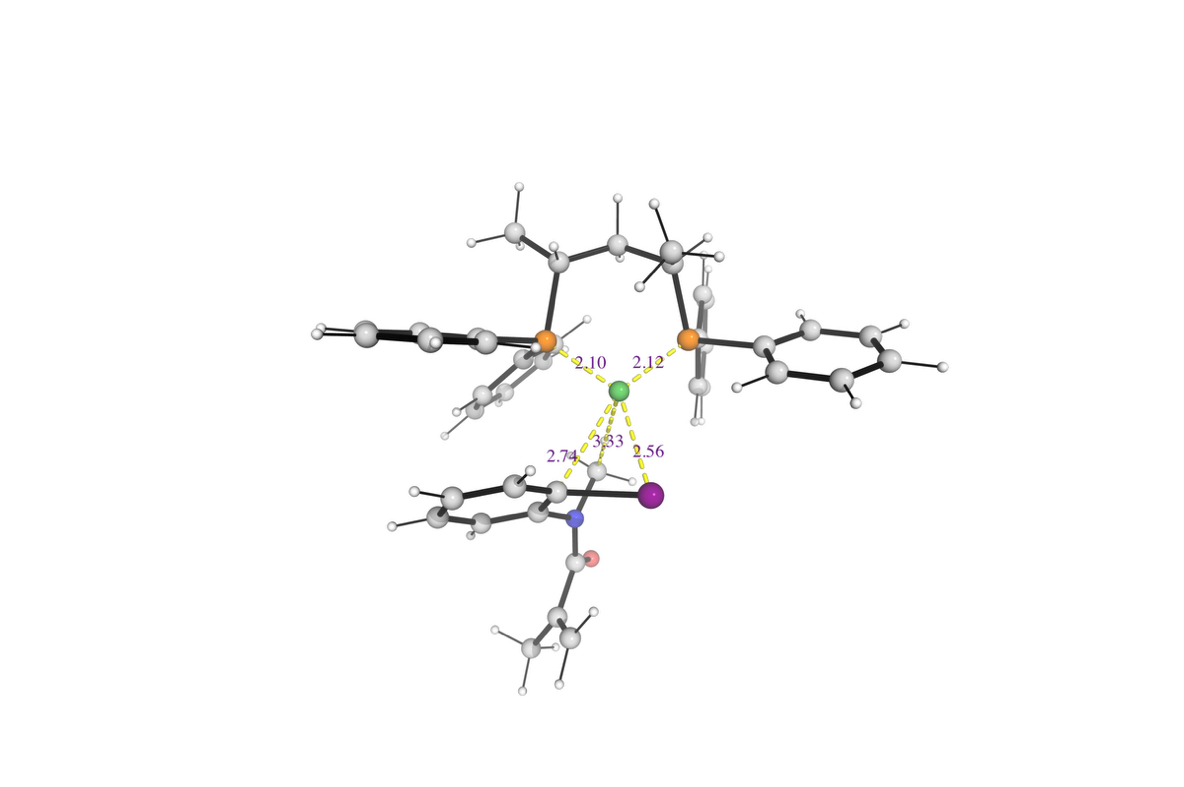 | 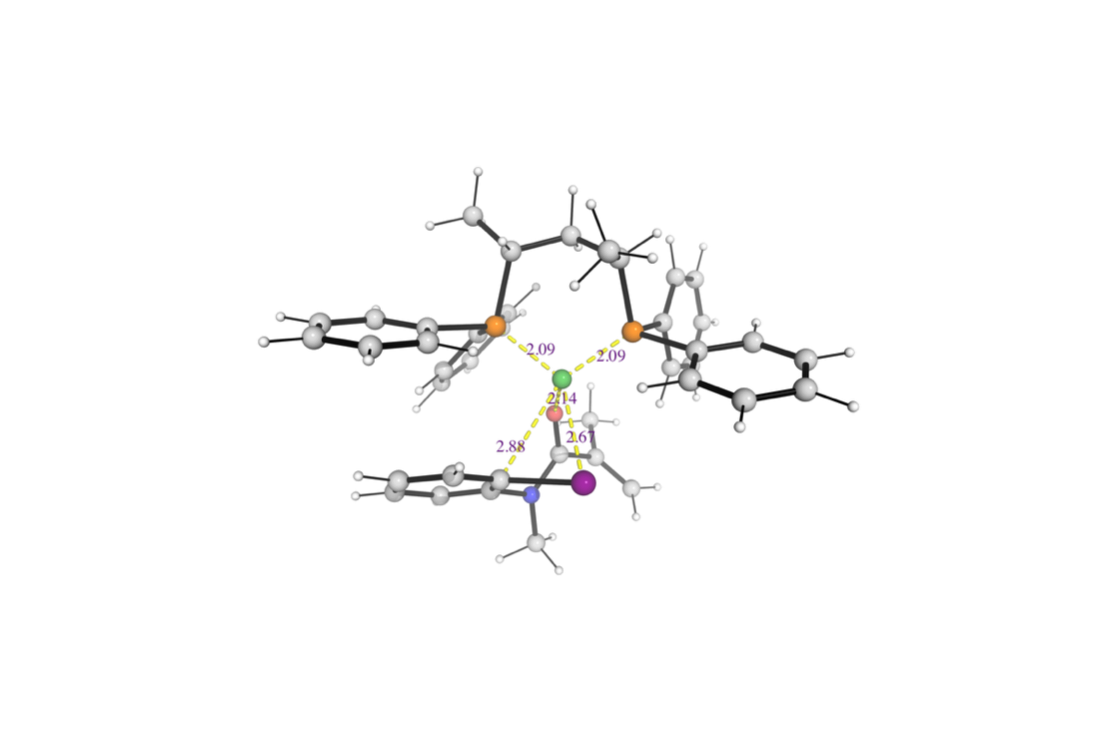 |
| **TS1d-I** | **TS1d-I-O** |
| ΔG^‡^ = 0.5 | ΔG^‡^ = 0.0 |
| 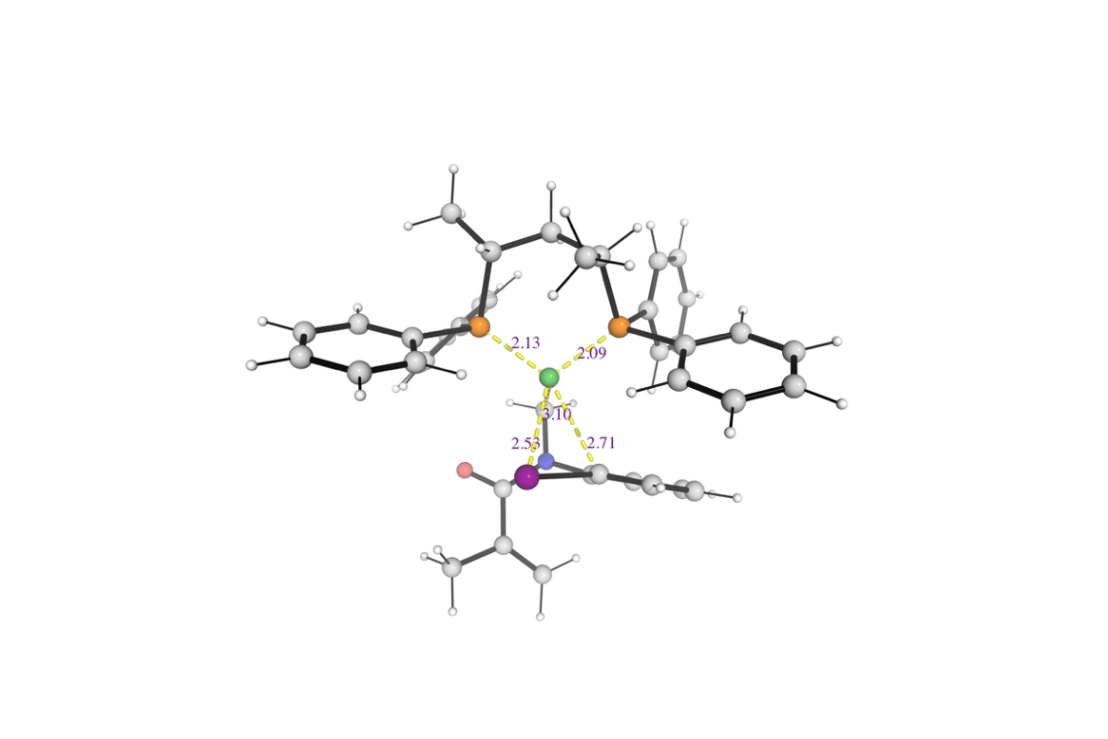 | 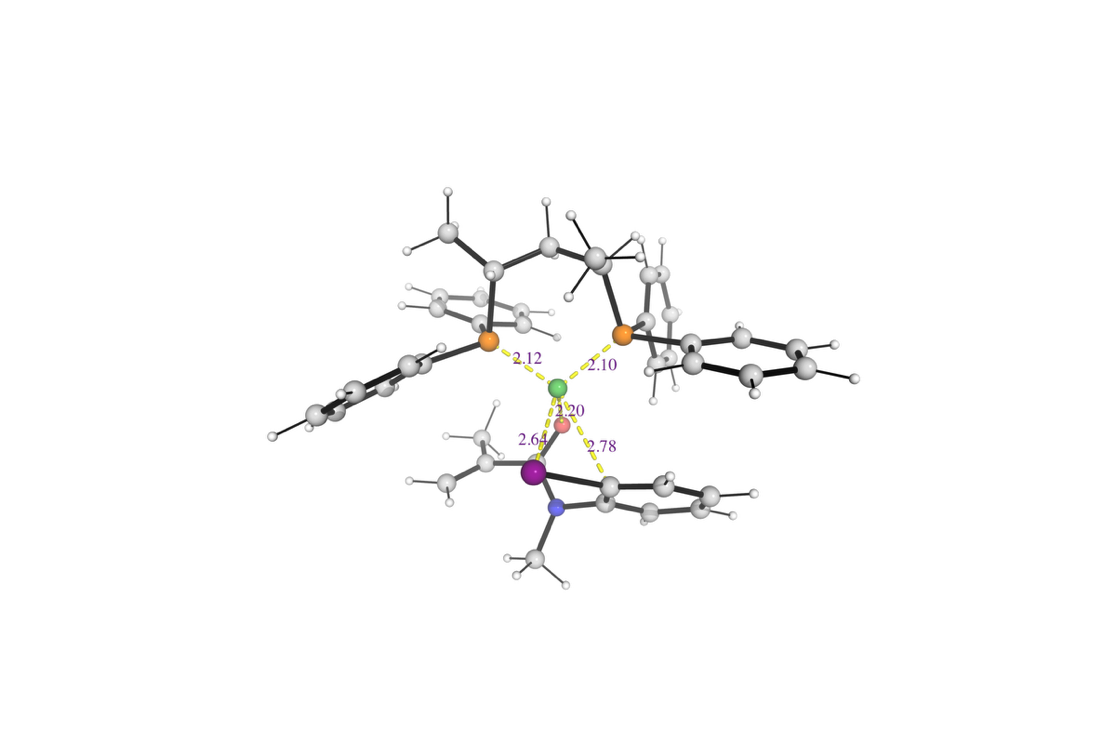 |

**Figure S2.** DFT-optimized structures of different conformers of Ni insertion (oxidative addition) transition states (**TS1**s) using aryl iodide as starting material. Gibbs free energies are given relative to the ground state of the combined reactants and ligand. Gibbs energies are given in SMD(N,N-dimethylformamide)-MN15/def2-QZVP//MN15/def2-SVP level of theories.

It should be noted that, to facilitate visual comparison among different transition state structures, the molecular orientations shown in Figure S2 were chosen such that the two chiral centers of the (*R,R*)-BDPP ligand were fixed in the same positional order for all structures. The “O” suffix was used to indicate the presence of an interaction between Ni and O in the corresponding structure. The labels “a”, “b”, “c”, and “d” in the structure names are used to distinguish different approaching orientations of the ligand.

By comparison, the structure **TS1a-I** had the lowest barrier of -1.8 kcal·mol⁻¹, on the contrary, **TS1b-I-O** had the highest barrier of 4.0 kcal·mol⁻¹. Notably, in **TS1b-I-O** the distances between the two P atoms and Ni (2.18 Å) were significantly longer than those observed in the other structures (2.09–2.13 Å). We assume that higher barrier observed for **TS1b-I-O** arose from the ligand entry orientation, which positioned the phenyl rings of the ligand near those of the aryl iodide. The resulting steric hindrance distorted the coordination geometry around the Ni center, weakened the P–Ni interactions, and consequently raised the transition state barrier. A similar steric effect also played an important role in determining the barrier heights of the other transition states.

We regarded **TS1c-I**, having a second lowest barrier of -0.6 kcal·mol⁻¹, as the main competing transition states of **TS1a-I**.

We further analyze the factors influencing the barrier by comparing the frontier molecular orbitals (FMOs), non-covalent interaction (NCI) and distortion-interaction analysis in the lowest energy competing TSs, **TS1a-I** and **TS1c-I**, as well as their corresponding TSs with Ni–O interactions, **TS1a-I-O** and **TS1c-I-O**. The results are shown in Figure S3 and Table S1.

|  | **TS1a-I** | **TS1a-I-O** |
| --- | --- | --- |
| **barrier** | ΔG^‡^ = -1.8 | ΔG^‡^ = 0.0 |
| **DFT**  **Structure** | 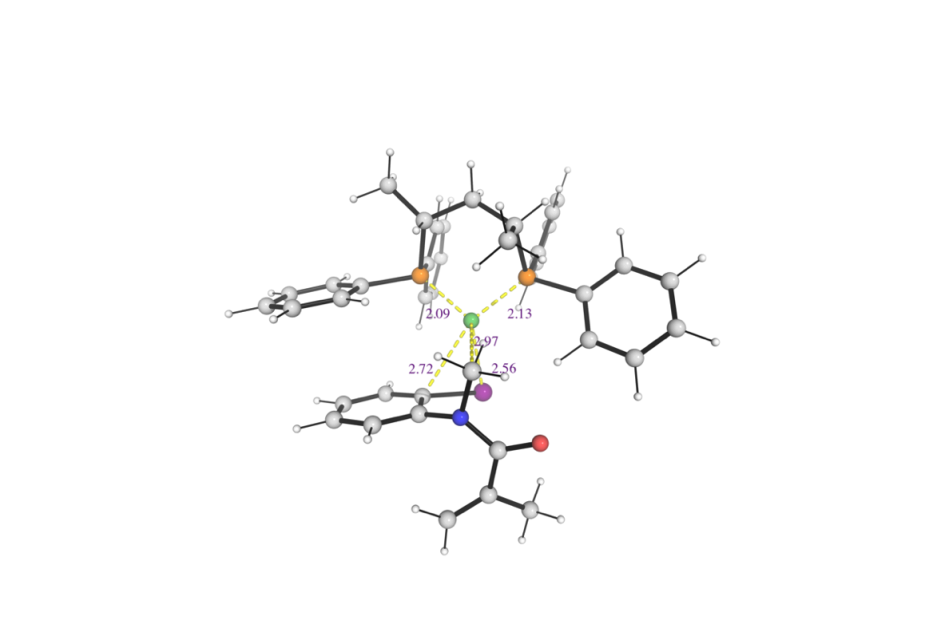 | 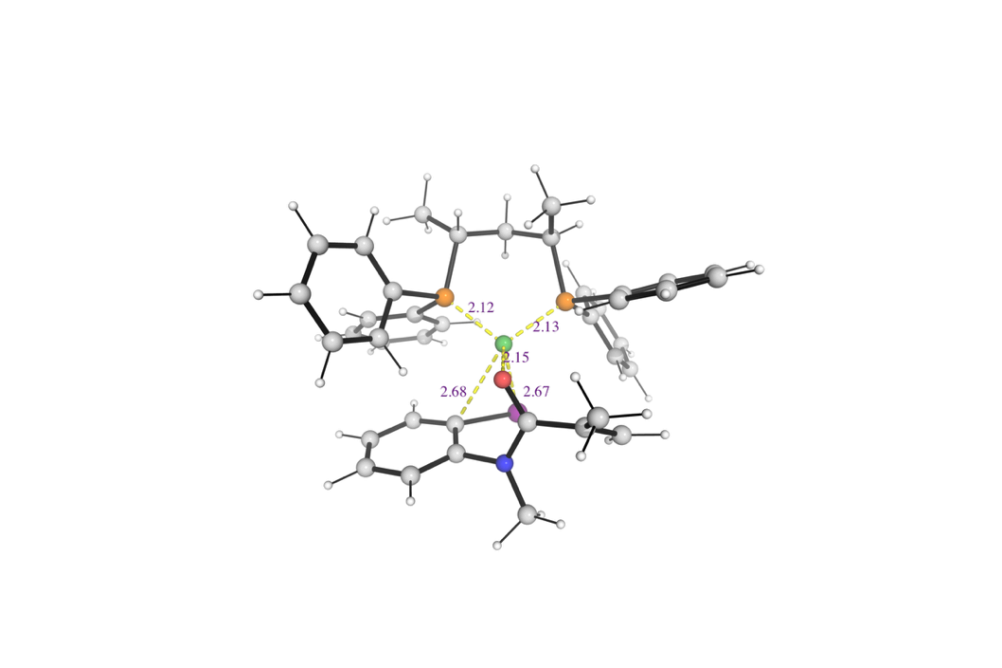 |
| **HOMO** | 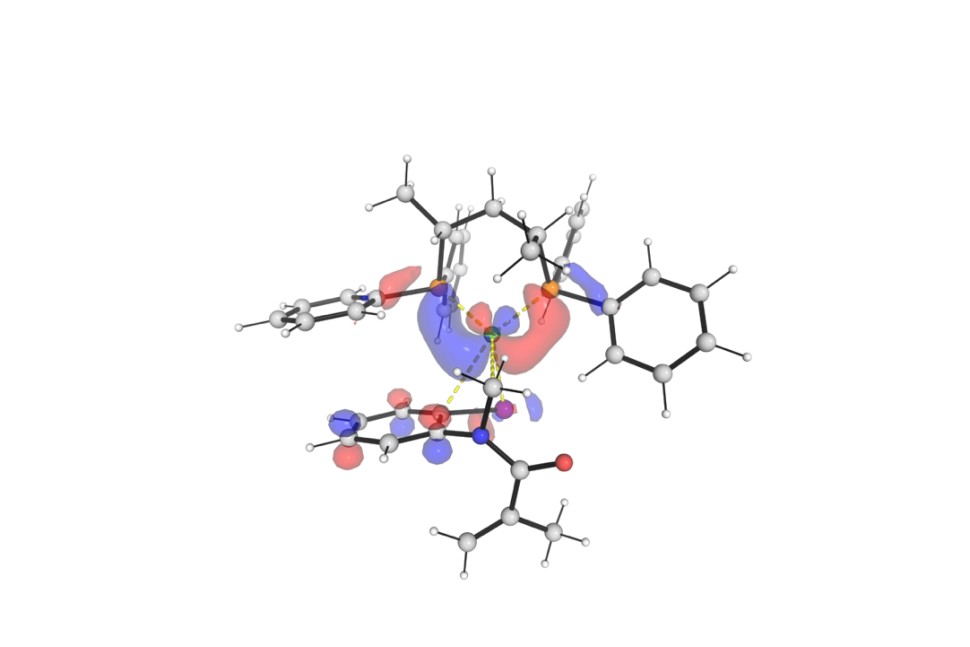 | 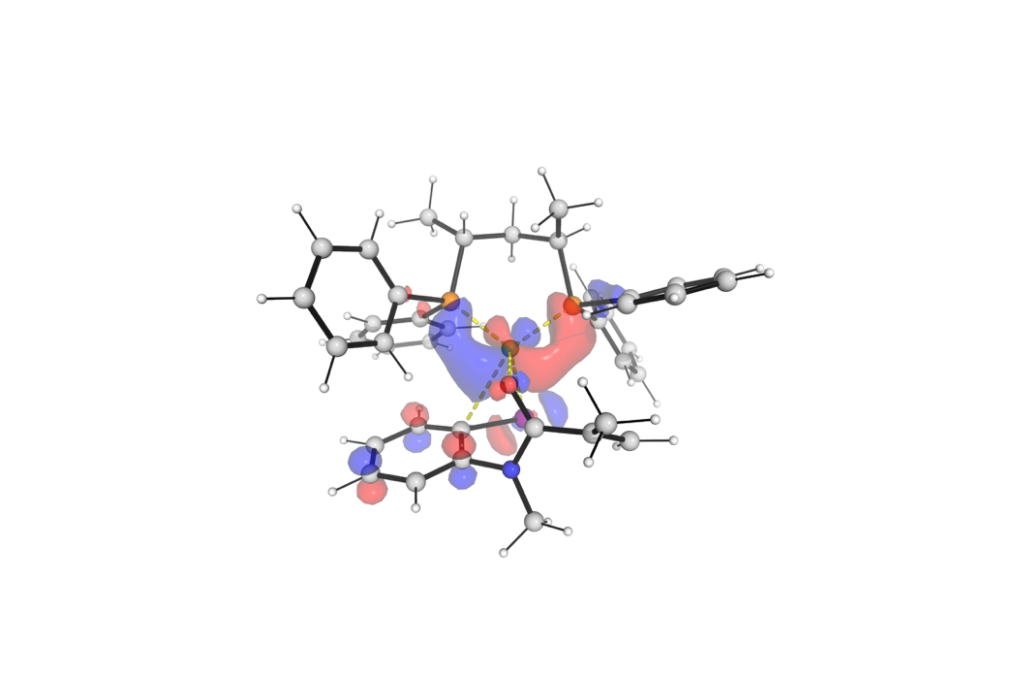 |
| **LUMO** | 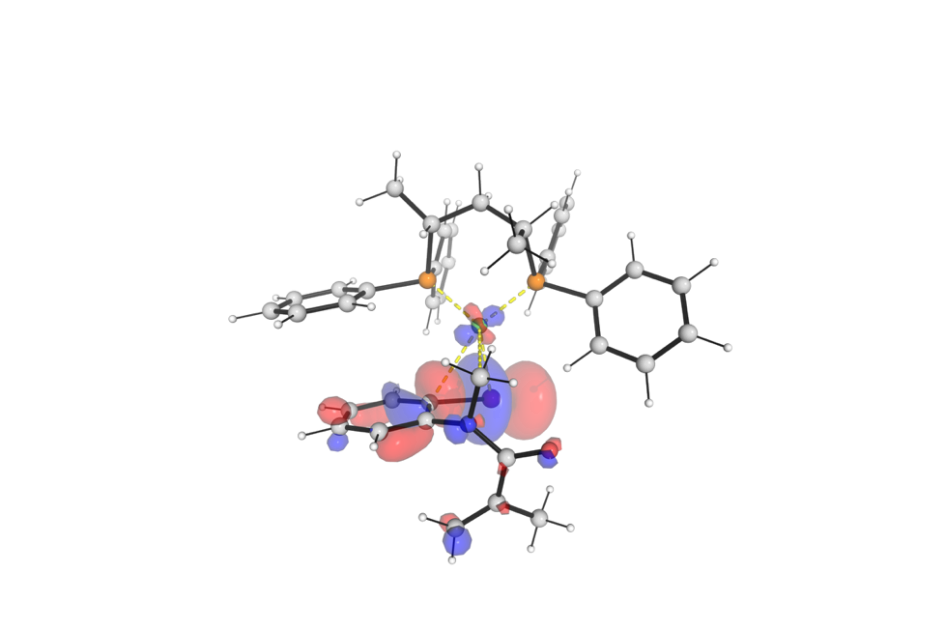 | 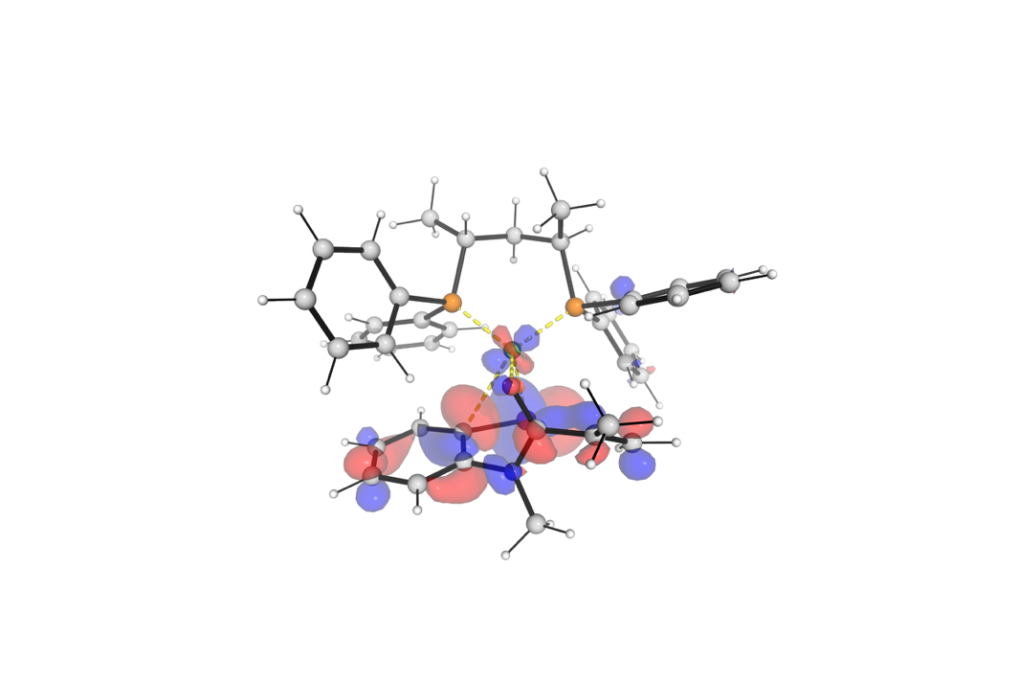 |
| **NCI** | 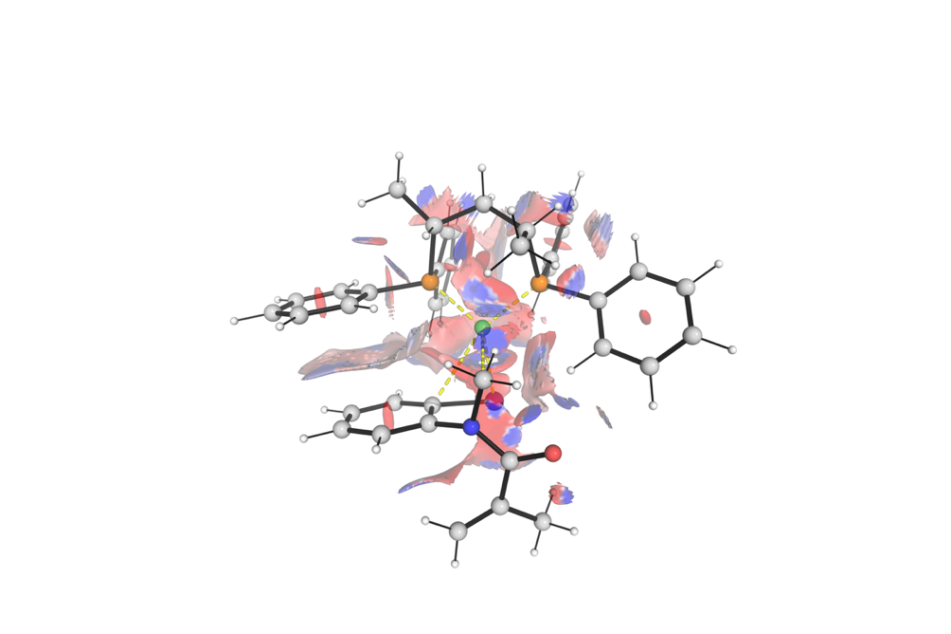 | 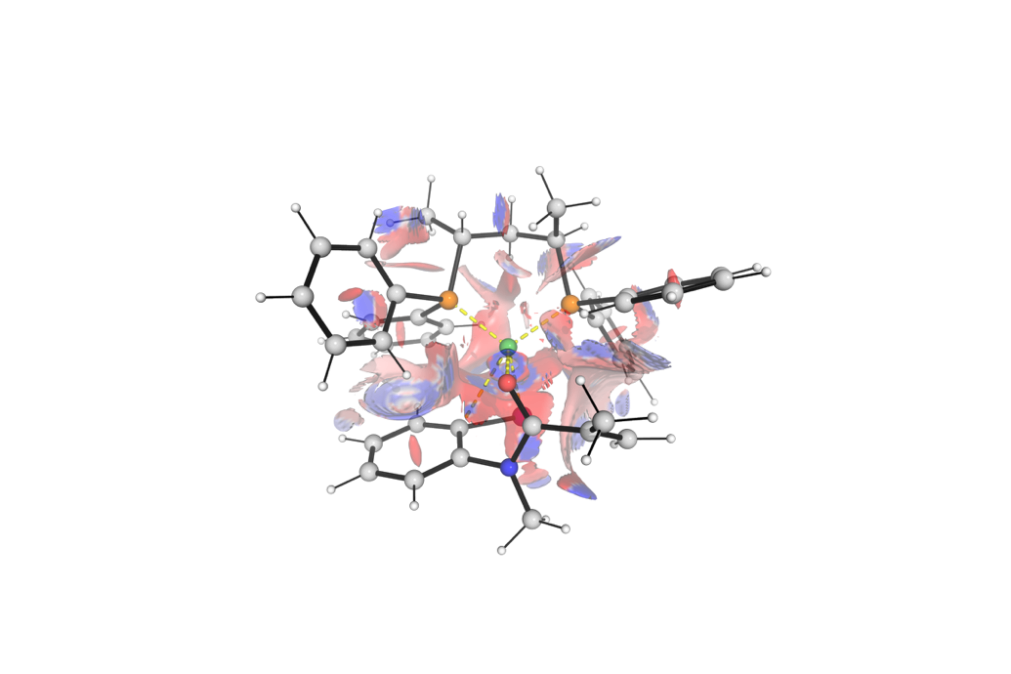 |
|  | 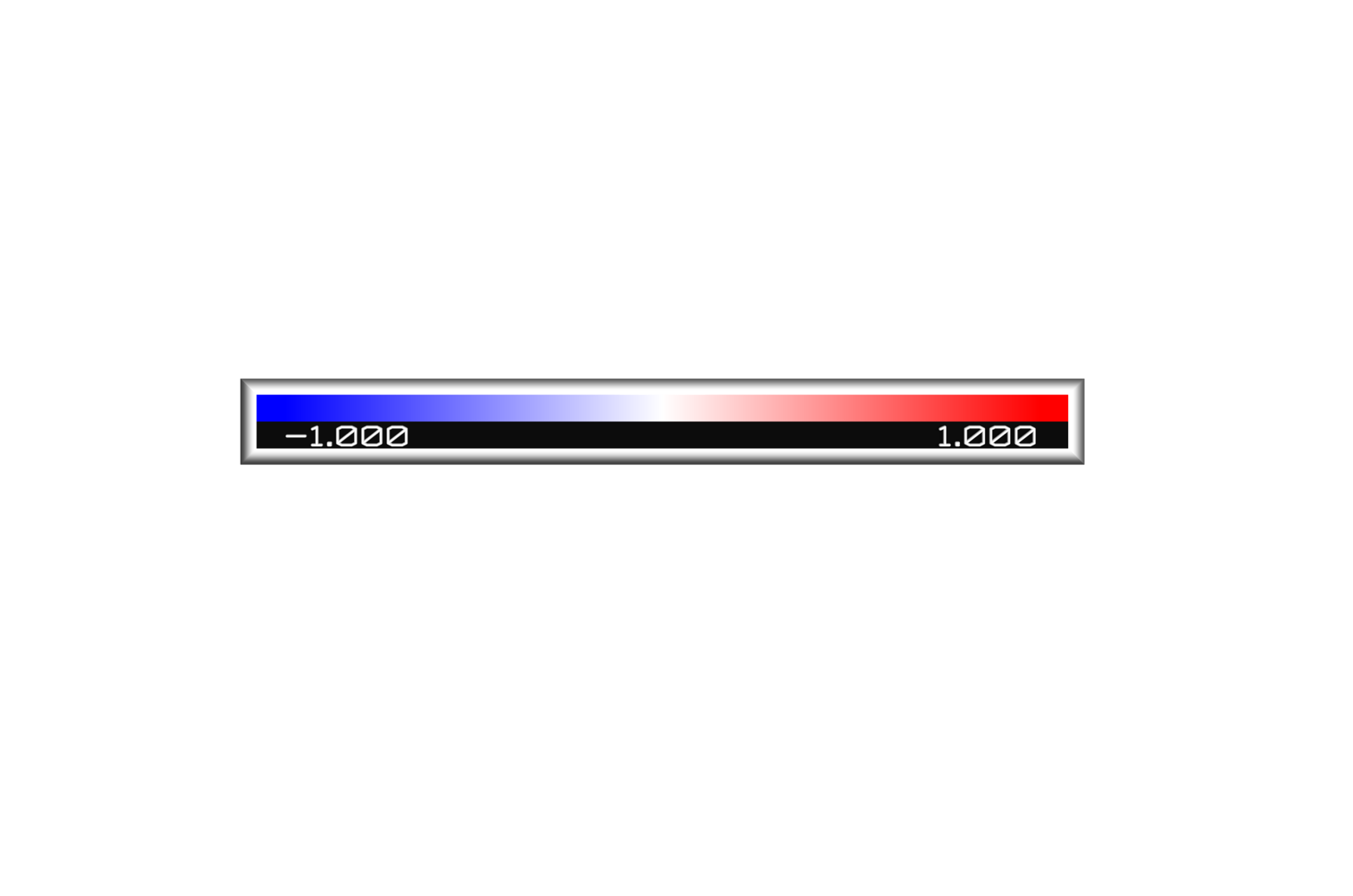 | |
|  | **TS1c-I** | **TS1c-I-O** |
| **barrier** | ΔG^‡^ = -0.6 | ΔG^‡^ = 3.6 |
| **DFT**  **Structure** | 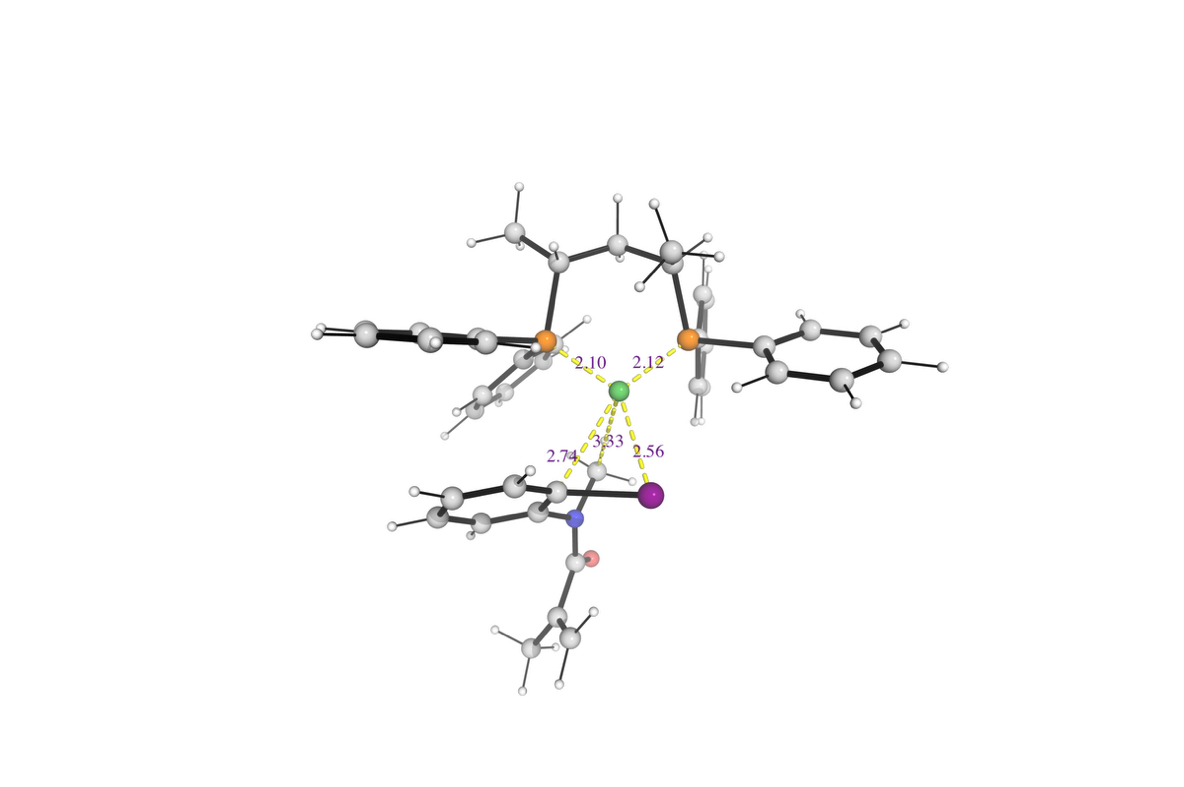 | 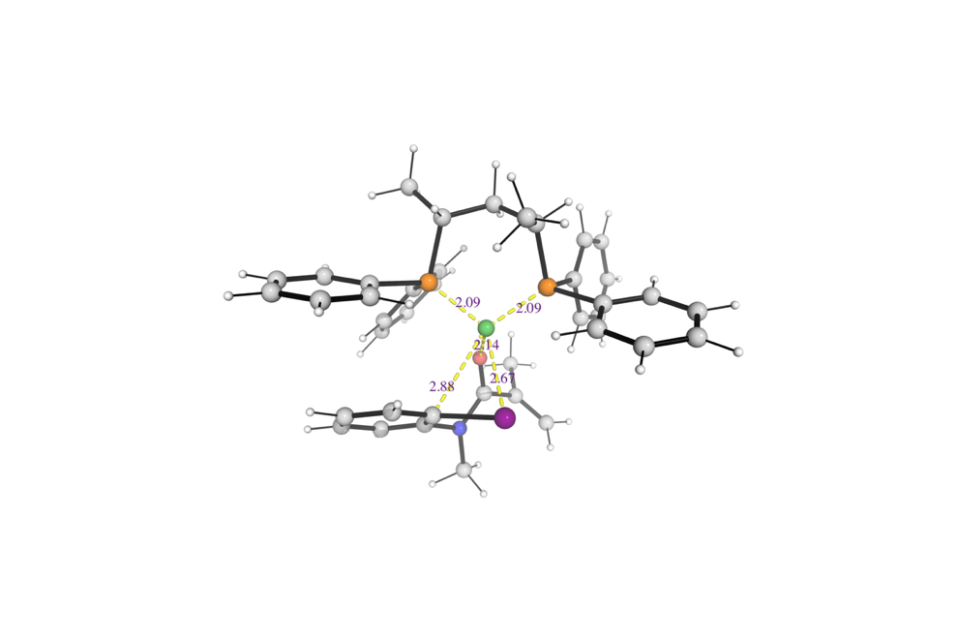 |
| **HOMO** | 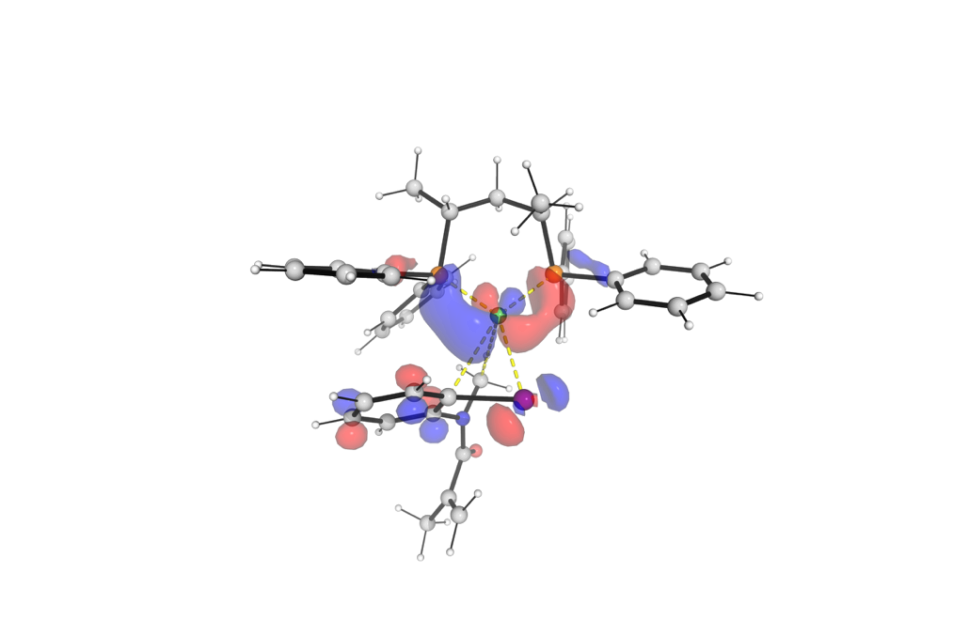 | 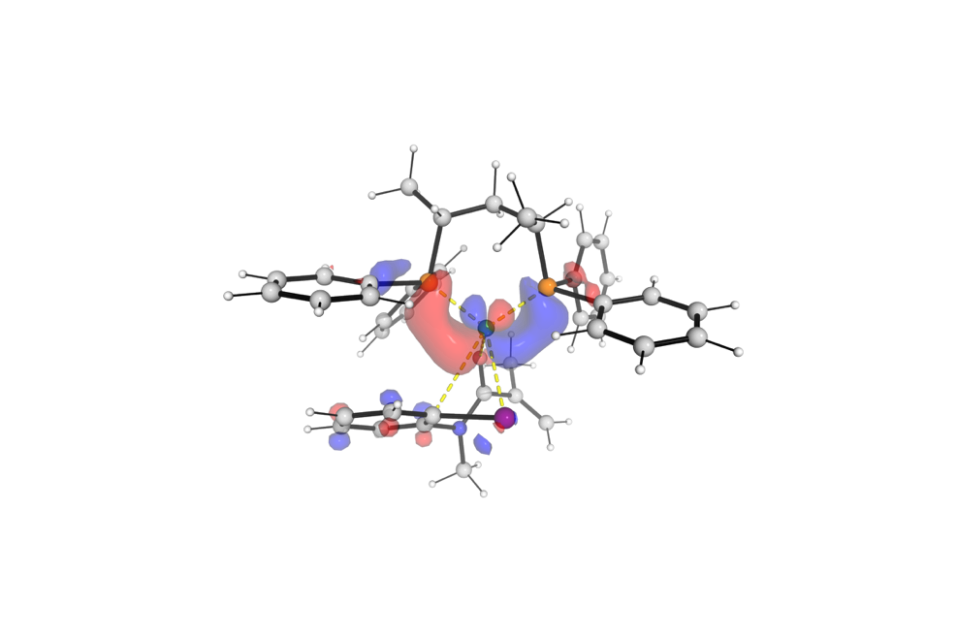 |
| **LUMO** | 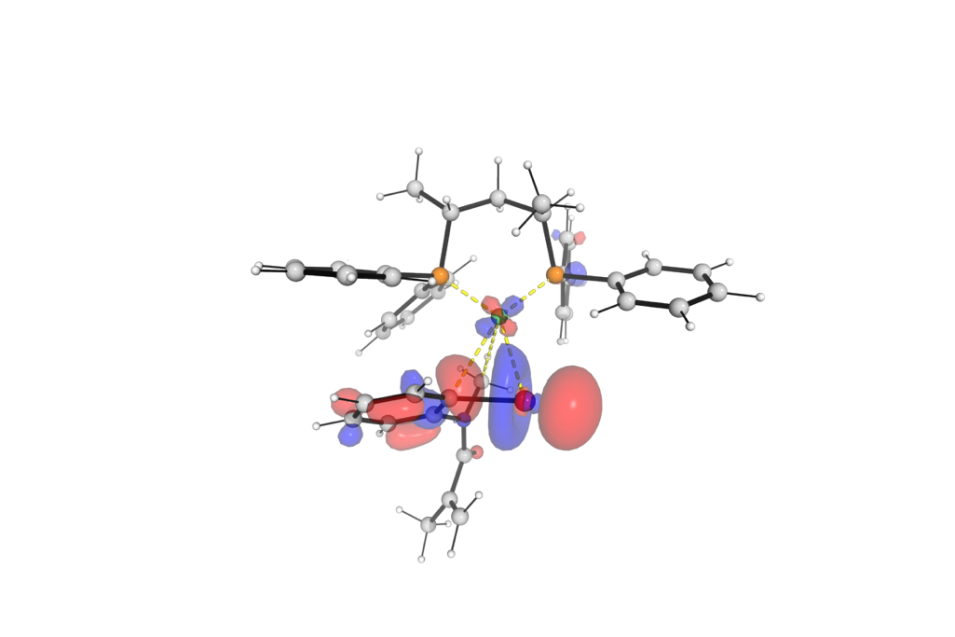 | 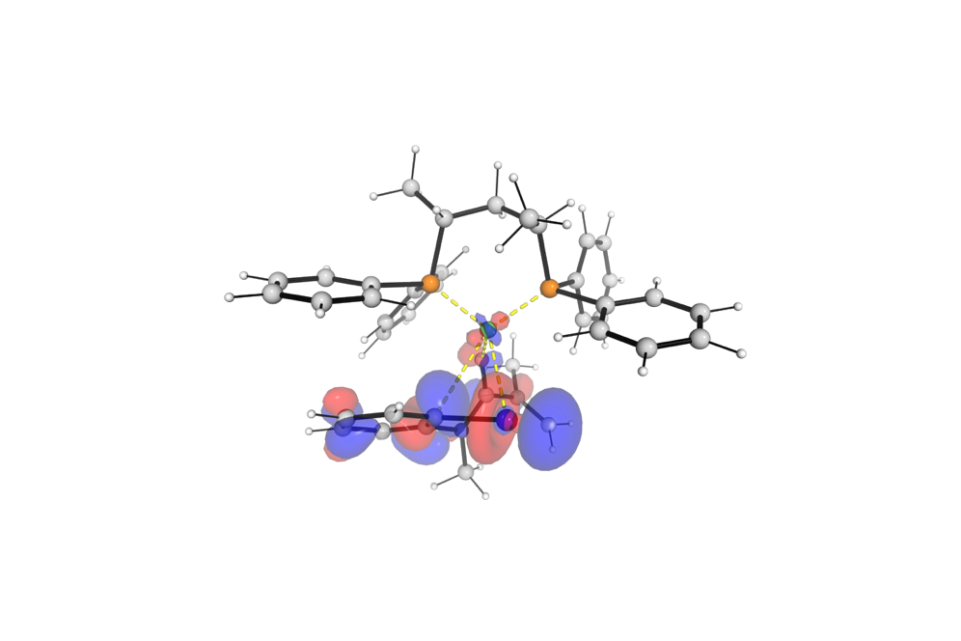 |
| **NCI** | 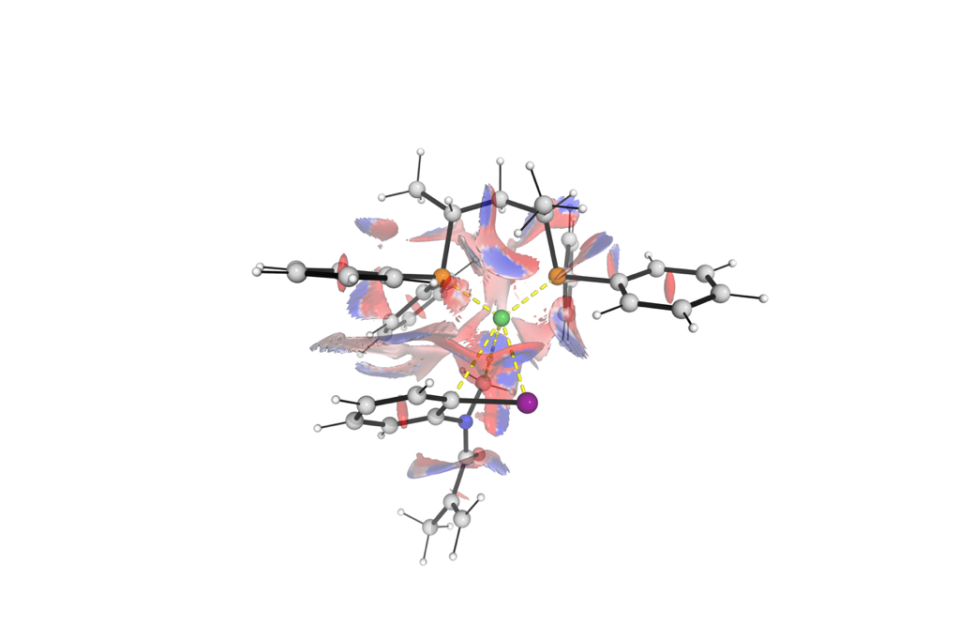 | 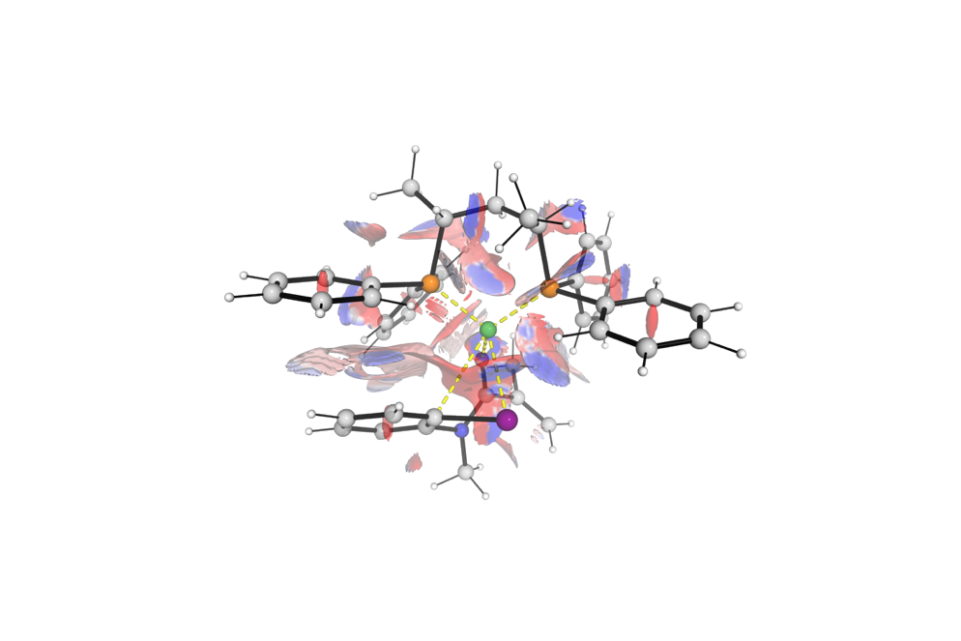 |
|  | 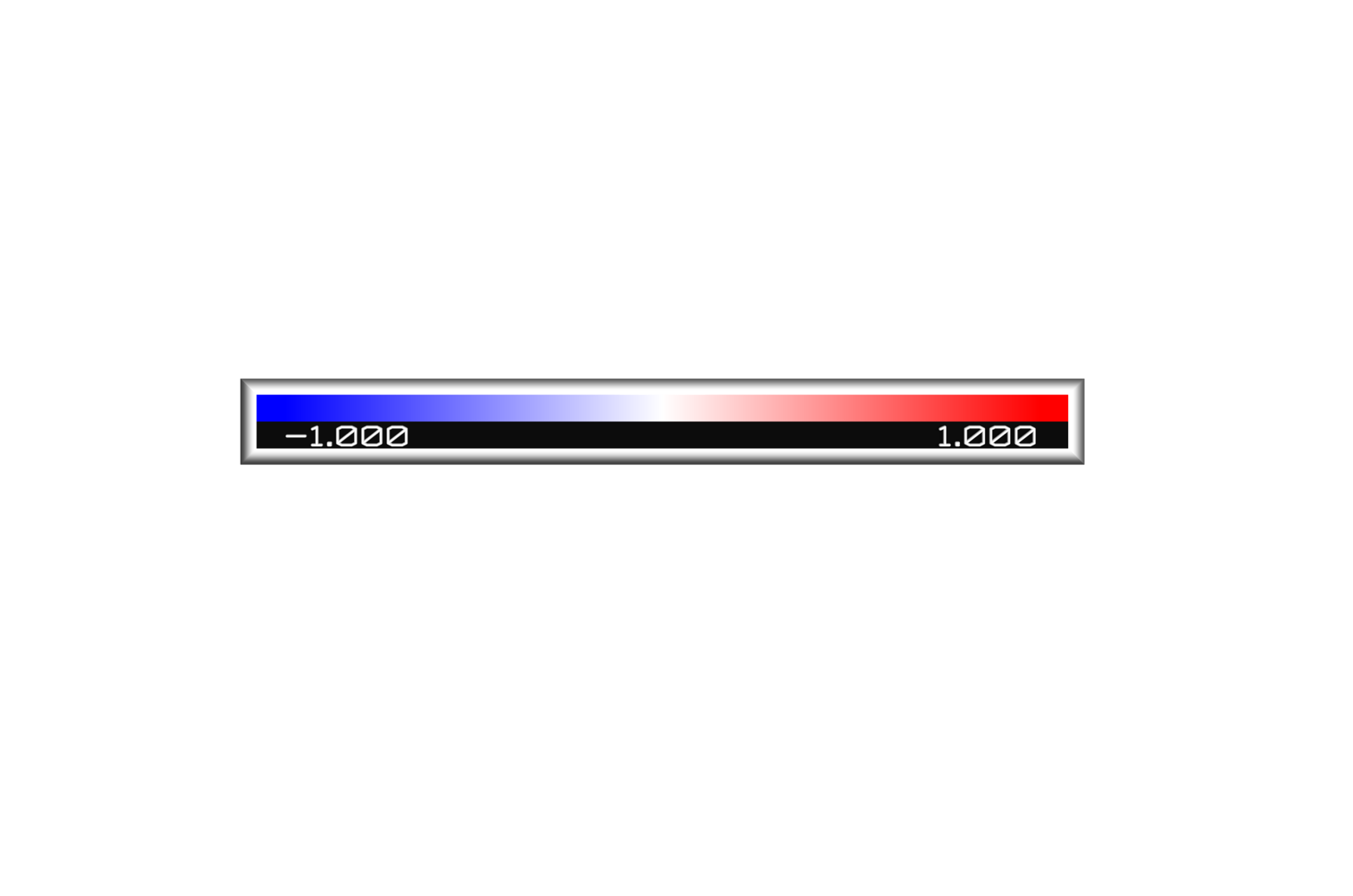 | |

**Figure S3.** DFT-optimized structures, frontier molecular orbitals (HOMO and LUMO) and non-covalent interaction (NCI) plots for selected transition state (**TS1a-I, TS1a-I-O, TS1c-I, TS1c-I-O**) for oxidative addition transition states (TSs) using aryl iodide as starting material.

Distortion-interaction analysis^19,20^ is applied to key TSs. The transition state structures are decomposed by dividing (*R*,*R*)-BDPP, coordinated with Ni, and aryl iodide as components. Single point calculations at SMD(N,N-dimethylformamide) solvent correction were applied performed at MN15/def2-QZVP level of theory to obtain distortion and interaction energies. The distortion energy is given by:

*E_dist_ =E_TS,frag1_ + E_TS,frag2_ − (E_eq,frag1_ + E_eq,frag2_ )*

where *TS,frag1,2* represent individual fragments in their distorted transition state geometries; and *eq,frag1,2* represent individual fragments in their optimized, equilibrium ground-state geometries; the interaction energy is given by:

*E_int_ =E_TS_ − (E_TS,frag1_ + E_TS,frag2_ )*

which accounts for the stabilizing interactions (e.g., electrostatic, orbital, dispersion) between the distorted fragments in the TS.

Thus, the total activation energy is given by:

Δ 𝐸^‡^ = *E_dist_* + *E_int._*

Note that this single point activation energy and the activation energy differences ΔΔ𝐸^‡^ between the major and minor TSs may be different from the Gibbs energy differences ΔΔG^‡^ that is computed fully (including vibrational frequencies analysis) at SMD(N,N-dimethylformamide)-MN15/def2-QZVP//MN15/def2-SVP level of theory.

**Table S1.** **Distortion-interaction analysis for oxidative addition step using aryl iodide as starting material.**

| **Transition State** | **Δ 𝐸^‡^** | ***E_dist_*** | ***E_int_*** |
| --- | --- | --- | --- |
| **TS1a-I** | -21.2 | 4.2 | -25.5 |
| **TS1c-I** | -19.4 | 5.5 | -25.0 |
| **TS1a-I-O** | -19.9 | 11.5 | -31.4 |
| **TS1c-I-O** | -17.7 | 7.9 | -25.6 |

The analysis reveals that **TS1a-I** possesses the lowest reaction barrier and is 1.2 kcal mol⁻¹ more stable than **TS1c-I**, primarily due to its lower distortion energy (by 1.3 kcal·mol⁻¹) and a slightly more favorable interaction energy (by 0.5 kcal·mol⁻¹) when the two fragments approach each other. **TS1a-I** is favored over **TS1c-I**, by ΔΔ𝐸^‡^ = 1.8 kcal·mol⁻¹.

In contrast, in the presence of O–Ni interaction, although **TS1a-I-O** exhibits a 5.9 kcal·mol⁻¹ more stabilizing interaction energy, it requires overcoming a 7.3 kcal·mol⁻¹ higher distortion energy, which consequently results in a 1.8 kcal·mol⁻¹ higher barrier compared to **TS1a-I**.

**7.3.3 Competing transition states for migratory insertion step**

As mentioned above, due to the highly exergonic Gibbs energy of reaction from **TS1** to **INT2**, oxidative addition step is considered irreversible. Moreover, interconversion between the post-oxidative insertion intermediates (e.g., **INT2a-I** and **INT2c-I**) is prohibited by substantial steric hindrance. Therefore, based on the Eyring equation (see **Section 6.3.4** for detailed calculations), the majority of reactants are expected to proceed through **TS1a-I** and **TS1c-I** before undergoing subsequent transformations. Consequently, only these two transition states are taken as the basis for evaluating the subsequent pathways.


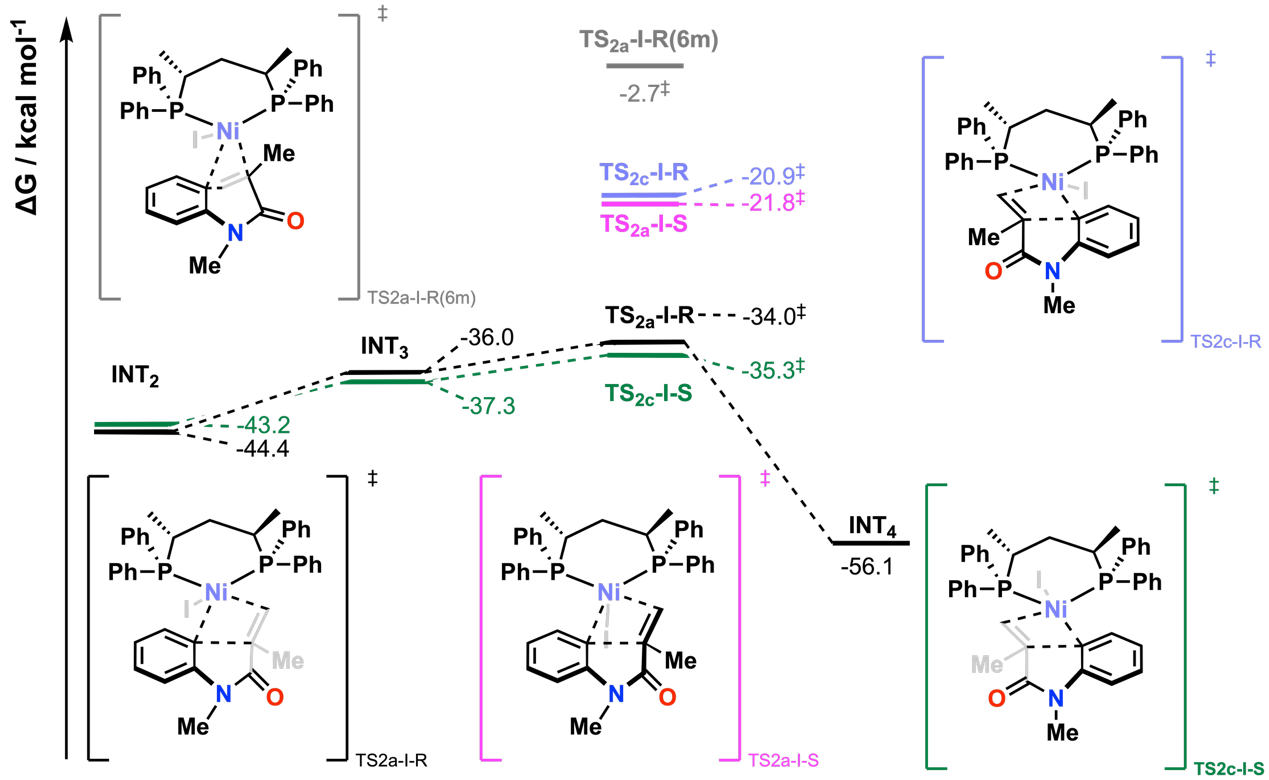


**Figure S4.** Gibbs energy detail for **TS2**s. Gibbs energies are given in SMD(N,N-dimethylformamide)-MN15/def2-QZVP//MN15/def2-SVP level of theories.

As a note, transition states named in the form of **TS2a-I-X** originate from **TS1a-I**, whereas those denoted as **TS2c-I-X** derive from **TS1c-I**. The label “(6m)” in the TS name indicates that the corresponding transition state leads to a six-membered-ring intermediate (which is not the target product), while the letters “R” and “S” denote the formation of *R*- and *S*-configured chiral centers, respectively.

The DFT-structures for transition states of migratory insertion step are shown in Figure S5.

| **TS2a-I-R** | **TS2a-I-S** |
| --- | --- |
| ΔG^‡^ = -34.0 | ΔG^‡^ = -21.8 |
| 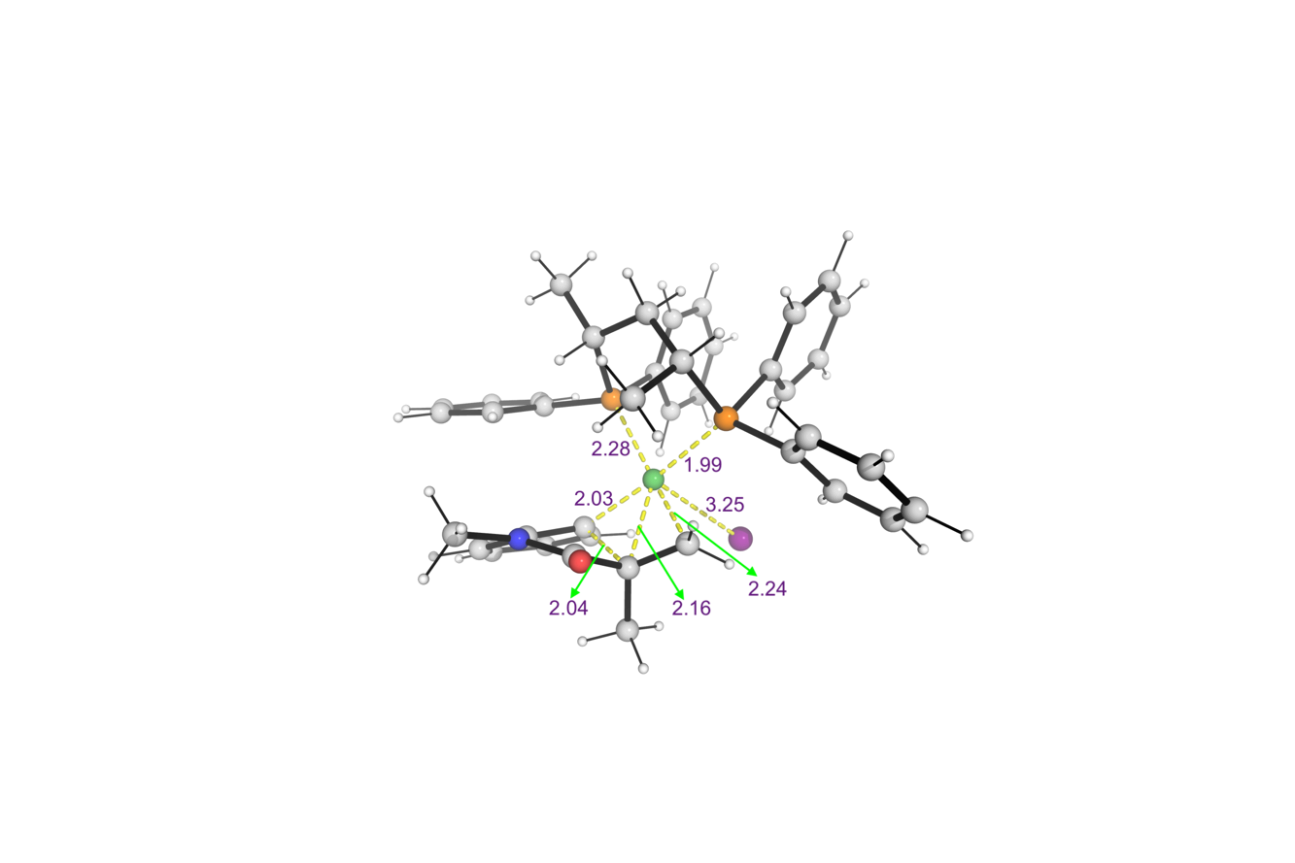 | 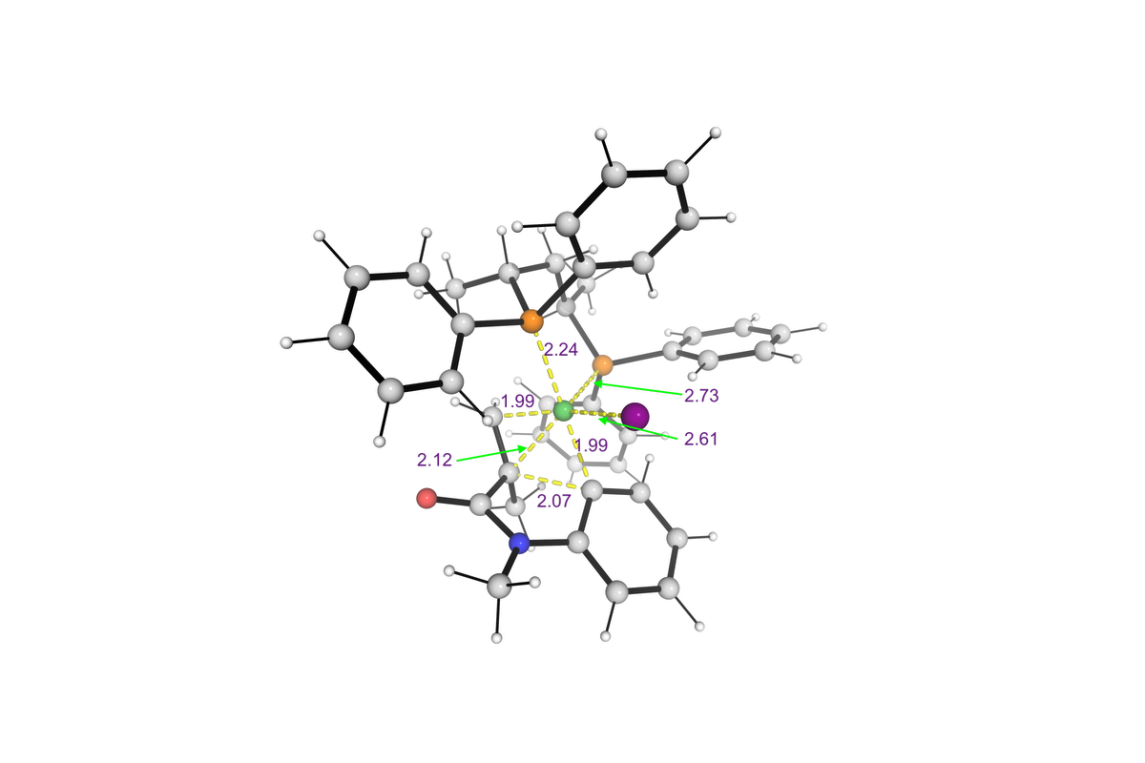 |
| **TS2c-I-S** | **TS2c-I-R** |
| ΔG^‡^ = -35.3 | ΔG^‡^ = -20.9 |
| 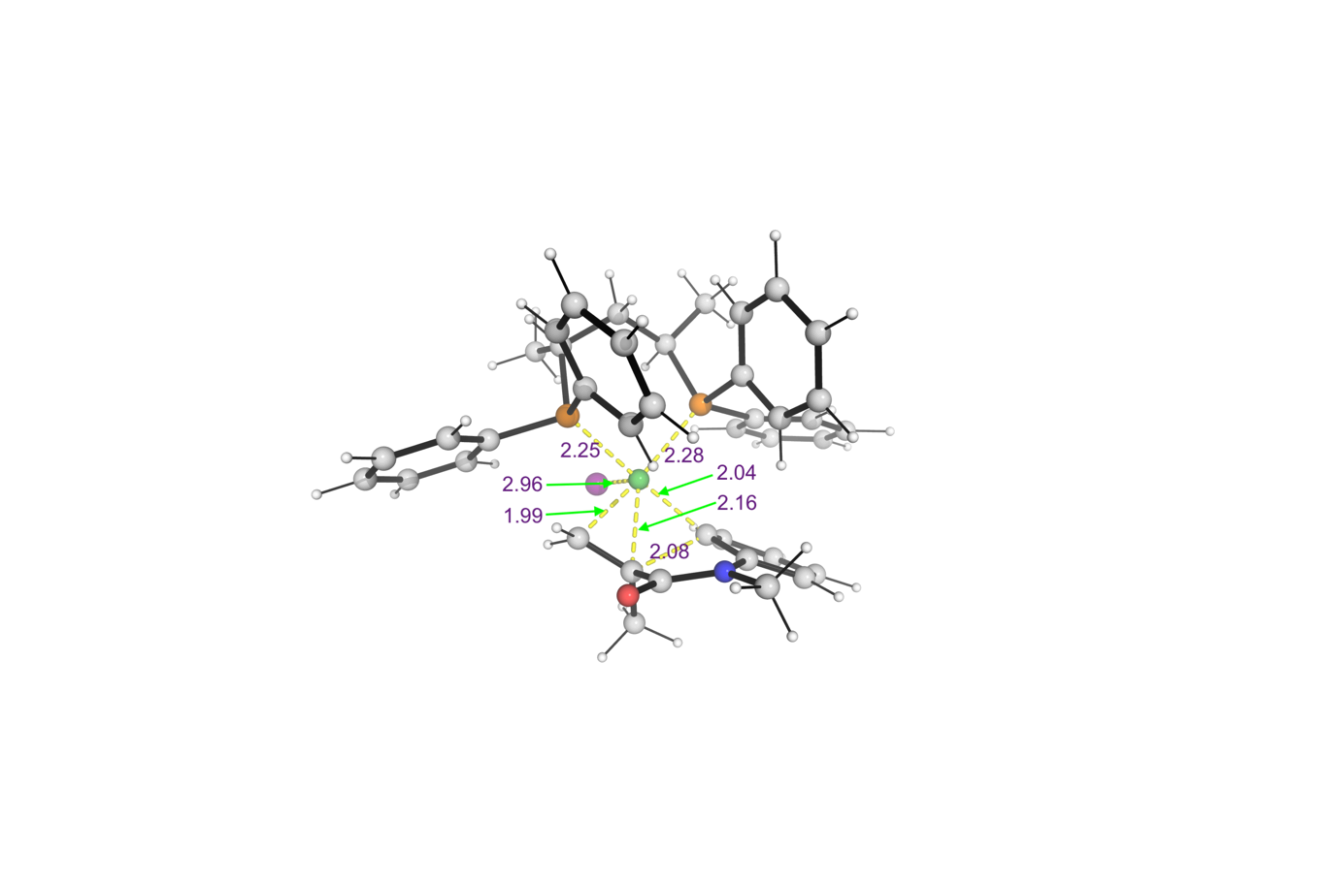 | 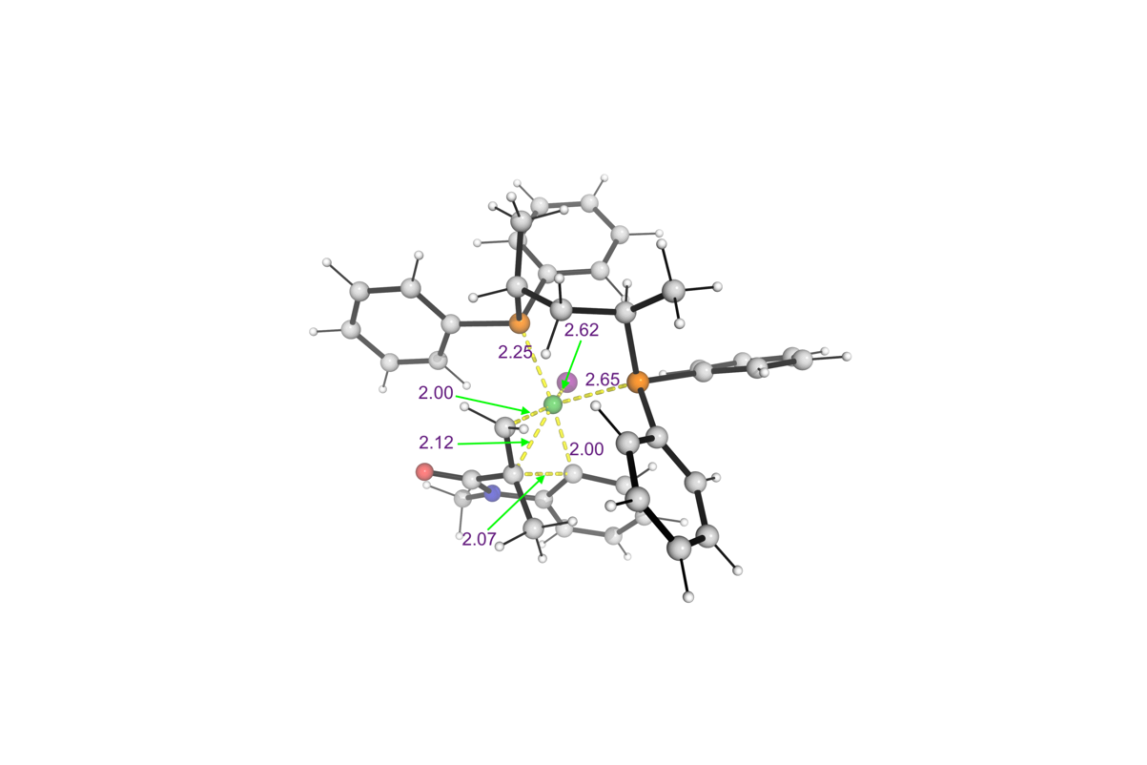 |
| **TS2a-I-R (6m)** |  |
| ΔG^‡^ = -2.7 |  |
| 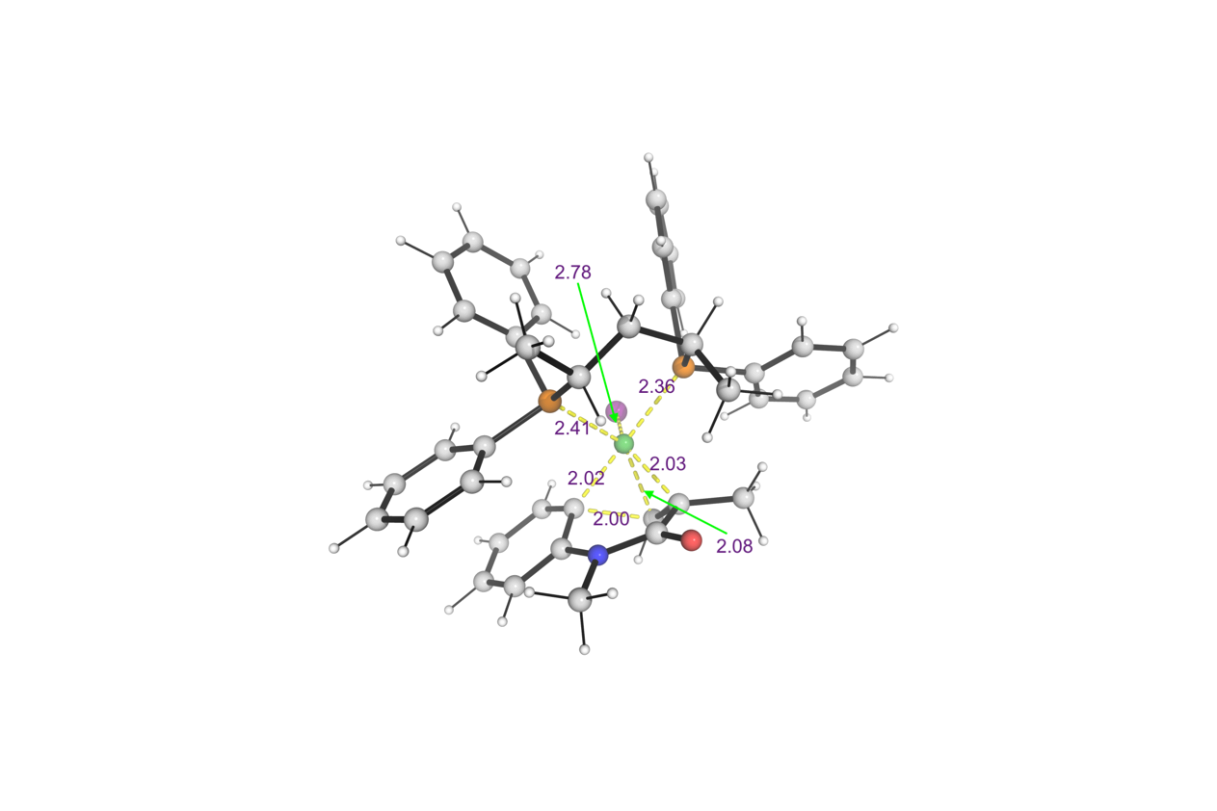 |  |

**Figure S5.** DFT-optimized structures of competing transition state in migratory insertion step, these structures were derived from selected **TS1**s that using aryl iodide as starting material, **TS1a-I** and **TS1c-I**. Gibbs free energies are given relative to the ground state of the combined reactants and ligand. Gibbs energies are given in SMD(N,N-dimethylformamide)-MN15/def2-QZVP//MN15/def2-SVP level of theories.

After the oxidative addition step, the acrylamide side chain can approach the reaction site from two orientations to undergo the subsequent Heck cyclization: (i) from the side adjacent to the iodide leaving group, or (ii) from the side opposite to iodide. Upon approach, the C=C bond of the acrylamide may also engage in a pathway that forms a six-membered ring; these possibilities are included in our set of competing TS2 structures.

As shown in Figure S5, the TSs in which the acrylamide side chain approaches from the iodide side exhibit >10 kcal·mol⁻¹ lower barriers than those approaching from the opposite side. Specifically, **TS2a-I-R** is 12.2 kcal·mol⁻¹ lower in barrier than **TS2a-I-S**, and **TS2c-I-S** is 14.4·kcal·mol⁻¹ lower than **TS2c-I-R**. Moreover, the TSs leading to six-membered rings are much higher in energy than the normal five-membered migratory-insertion TSs; for example, the barrier of **TS2a-I-R (6m)** is 31.3 kcal·mol⁻¹ higher than that of **TS2a-I-R**.

Based on estimates using the Eyring equation, the chirality of the final product is essentially predetermined once **INT2** is formed following the oxidative addition step. Detailed reasoning and calculations are provided in **Section 6.3.4**.

Analyses of FMOs and NCI were also performed for all competing **TS2** structures to elucidate the factors influencing the reaction barriers. The results are presented in Figure S6.

|  | **TS2a-I-R** | **TS2a-I-S** |
| --- | --- | --- |
| **barrier** | ΔG^‡^ = -34.0 | ΔG^‡^ = -21.8 |
| **DFT**  **Structure** | 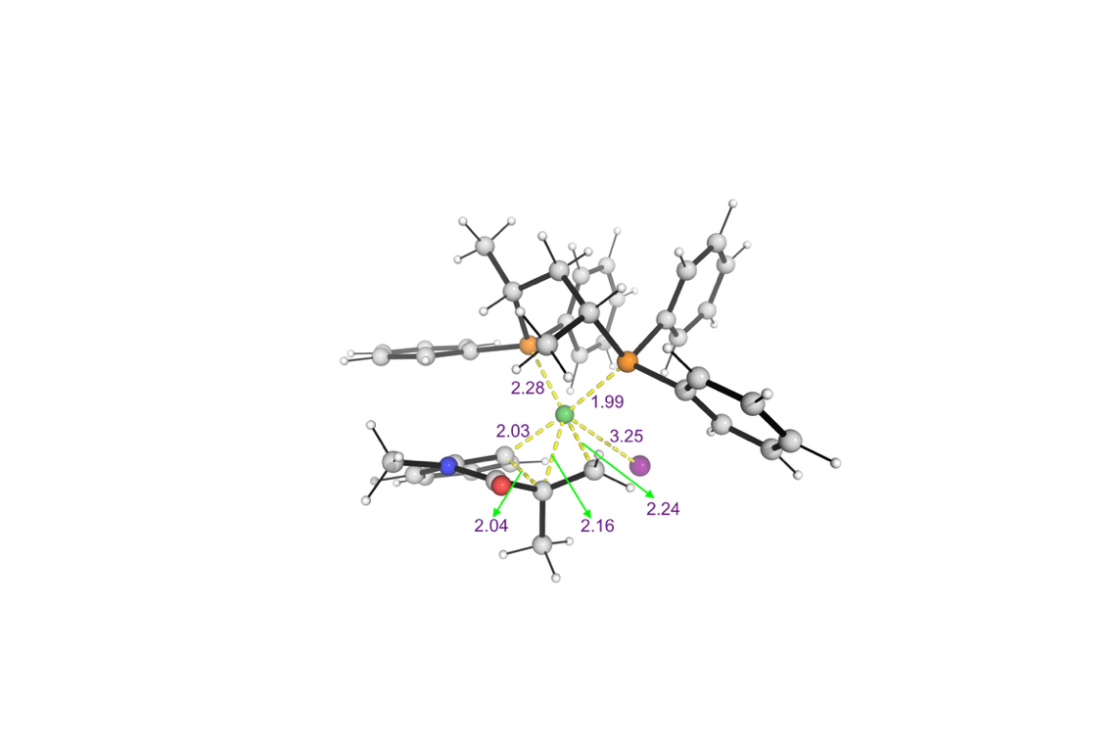 | 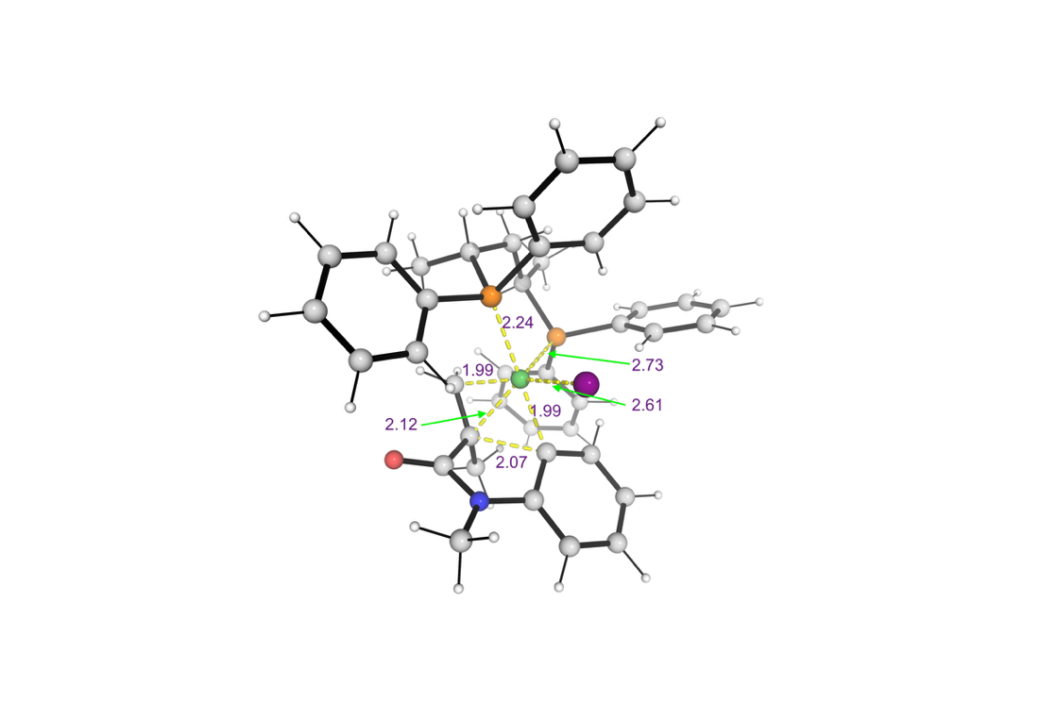 |
| **HOMO** | 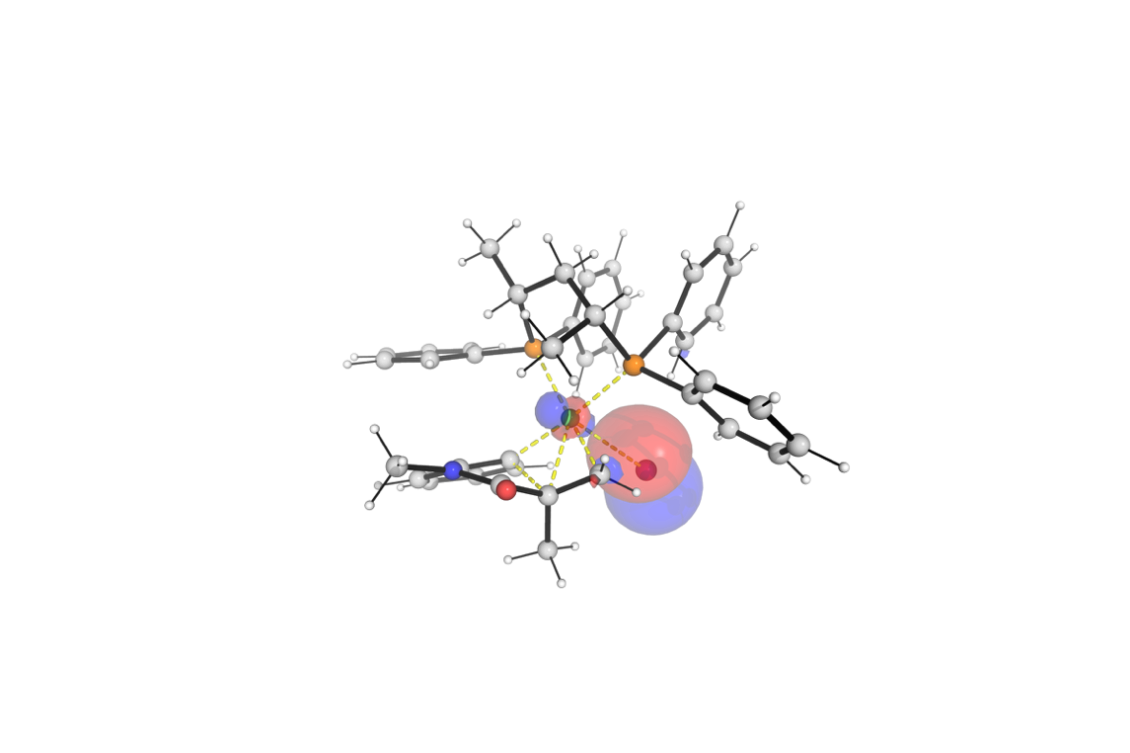 | 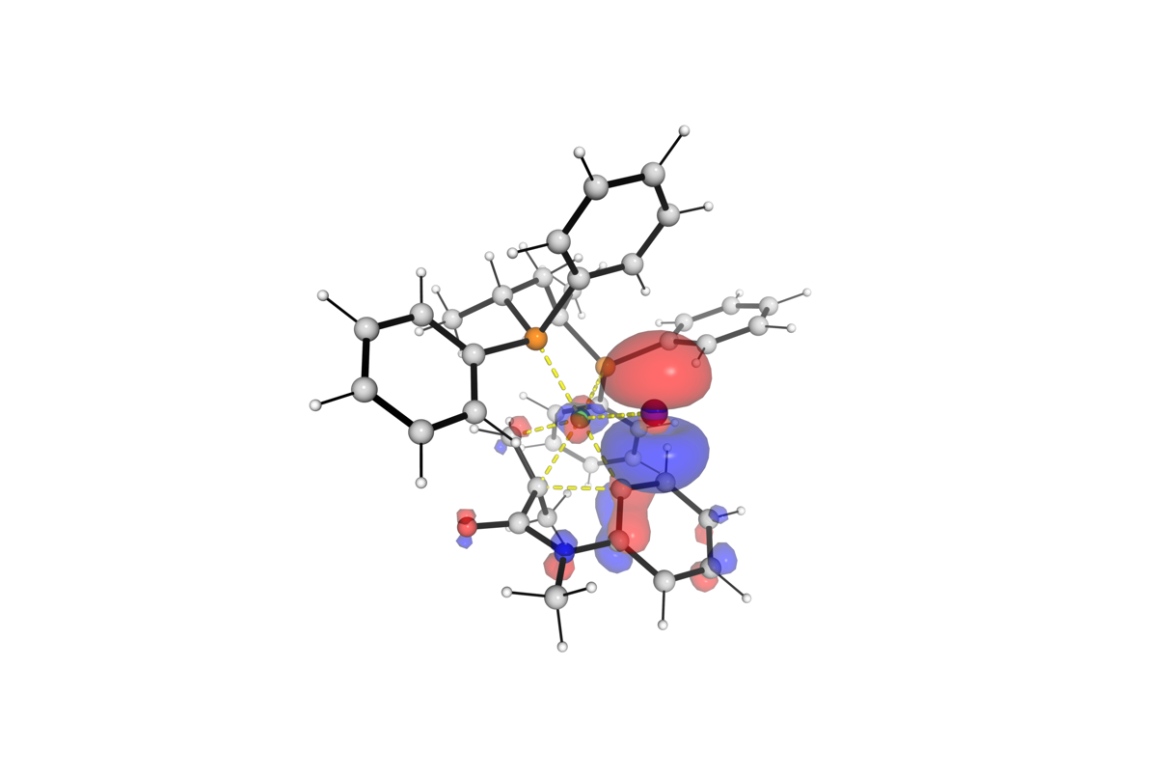 |
| **LUMO** | 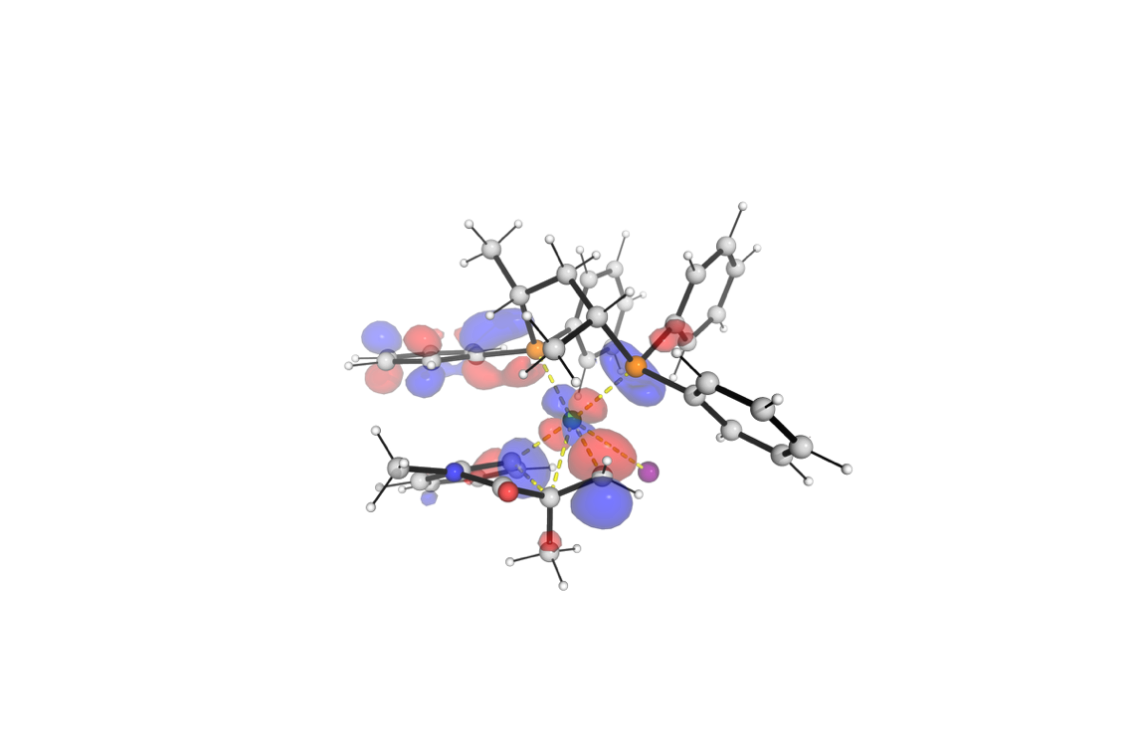 | 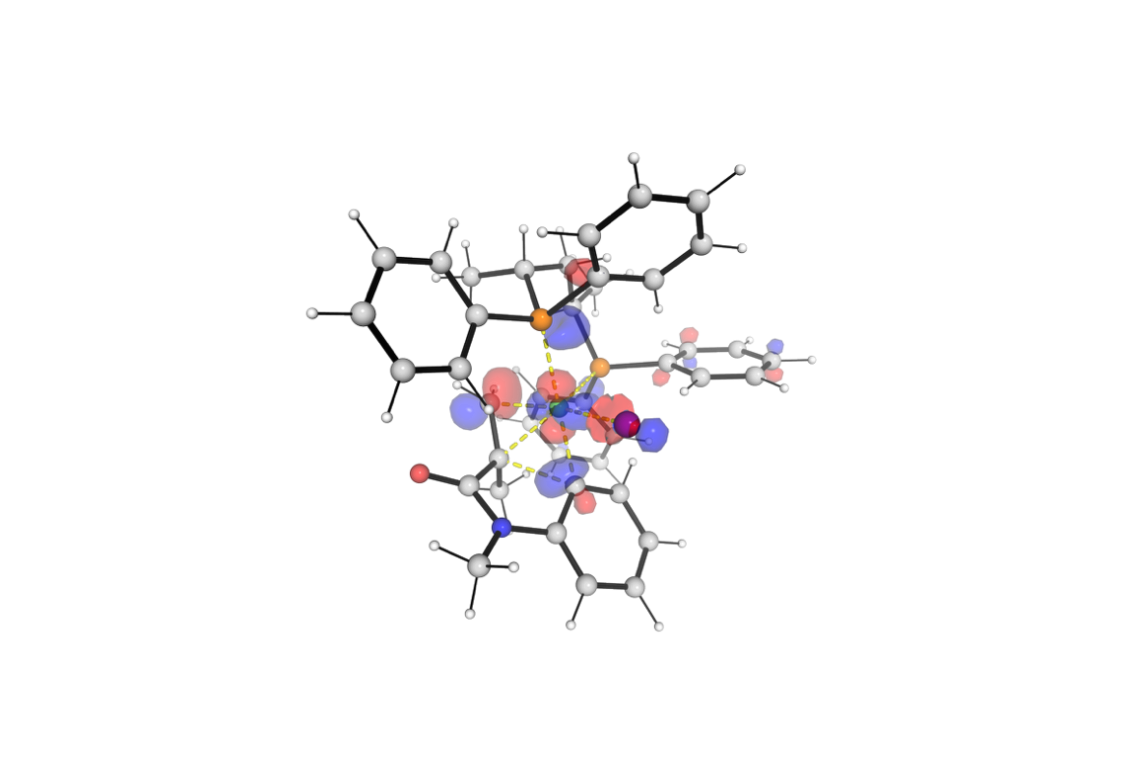 |
| **NCI** | 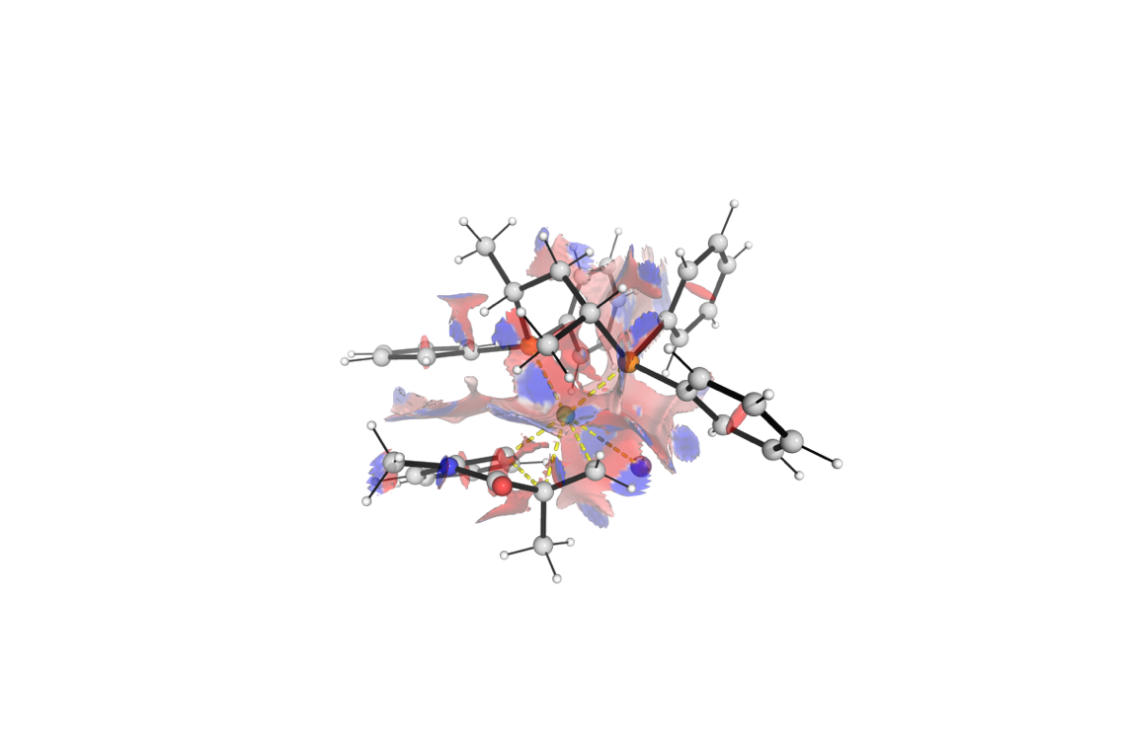 | 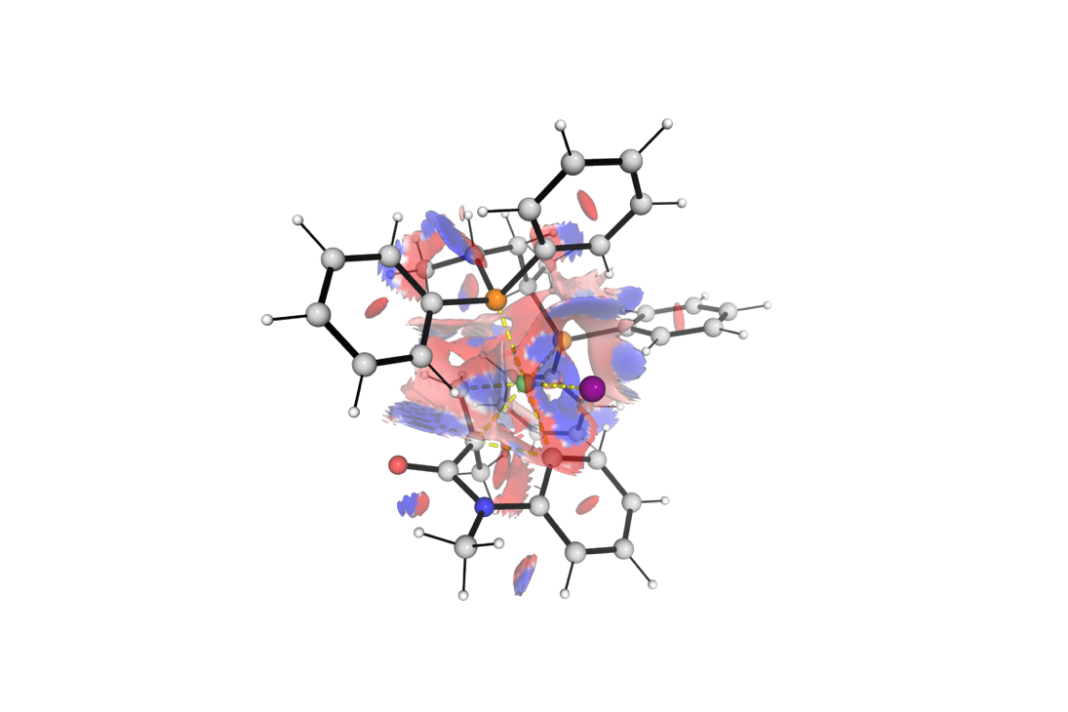 |
|  | 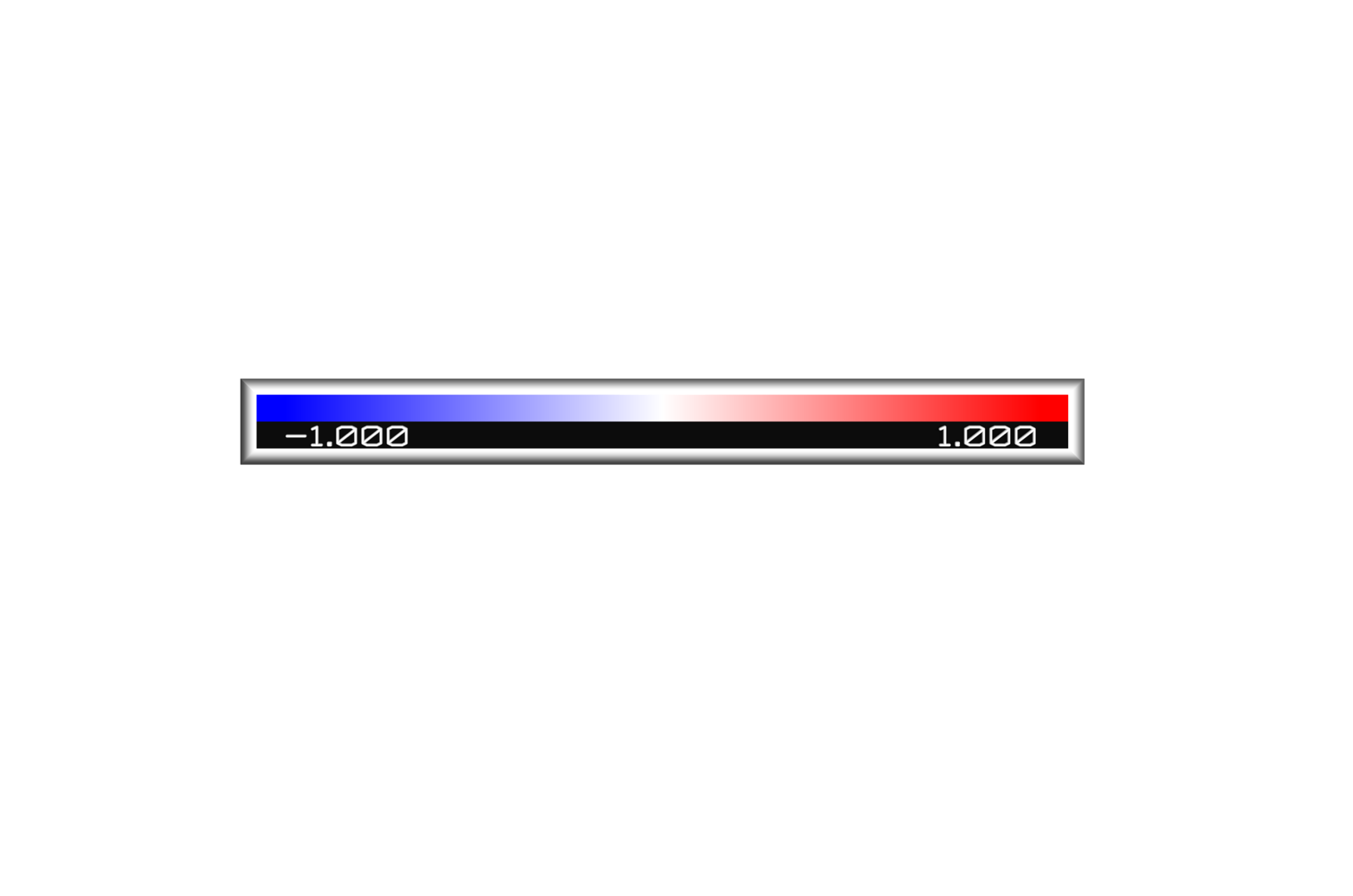 | |
|  | **TS2c-I-S** | **TS2c-I-R** |
| **barrier** | ΔG^‡^ = -35.3 | ΔG^‡^ = -20.9 |
| **DFT**  **Structure** | 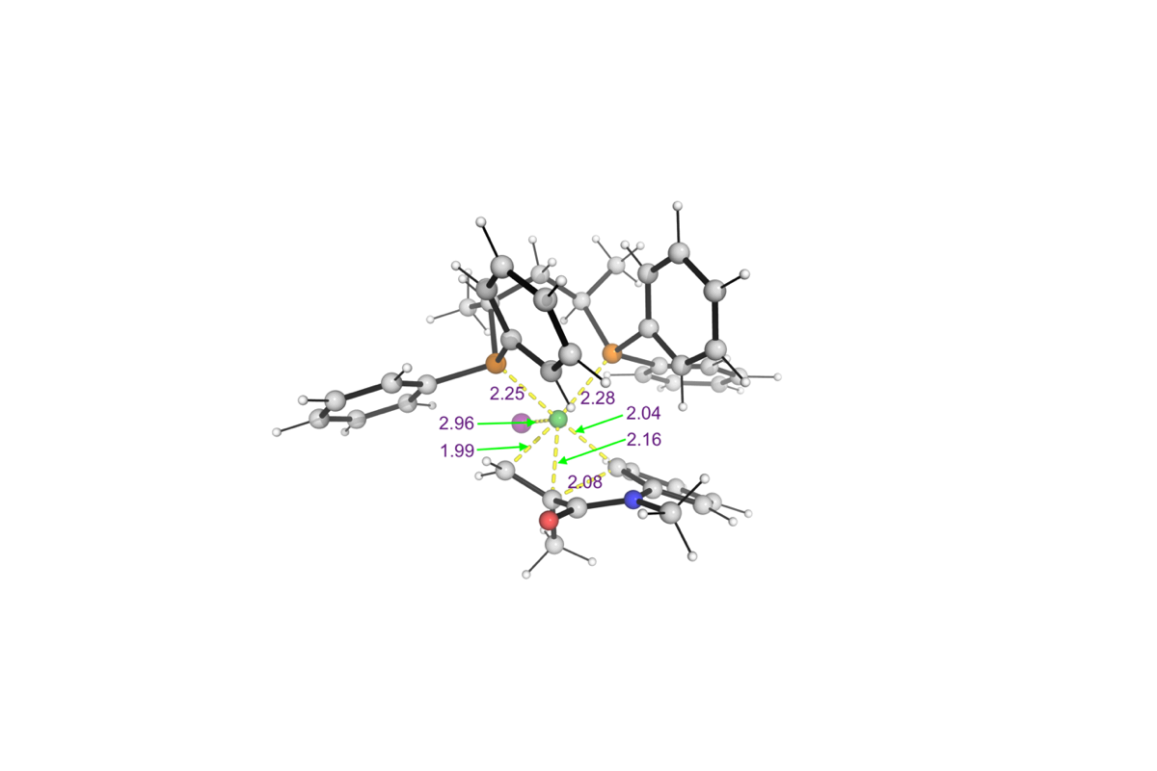 | 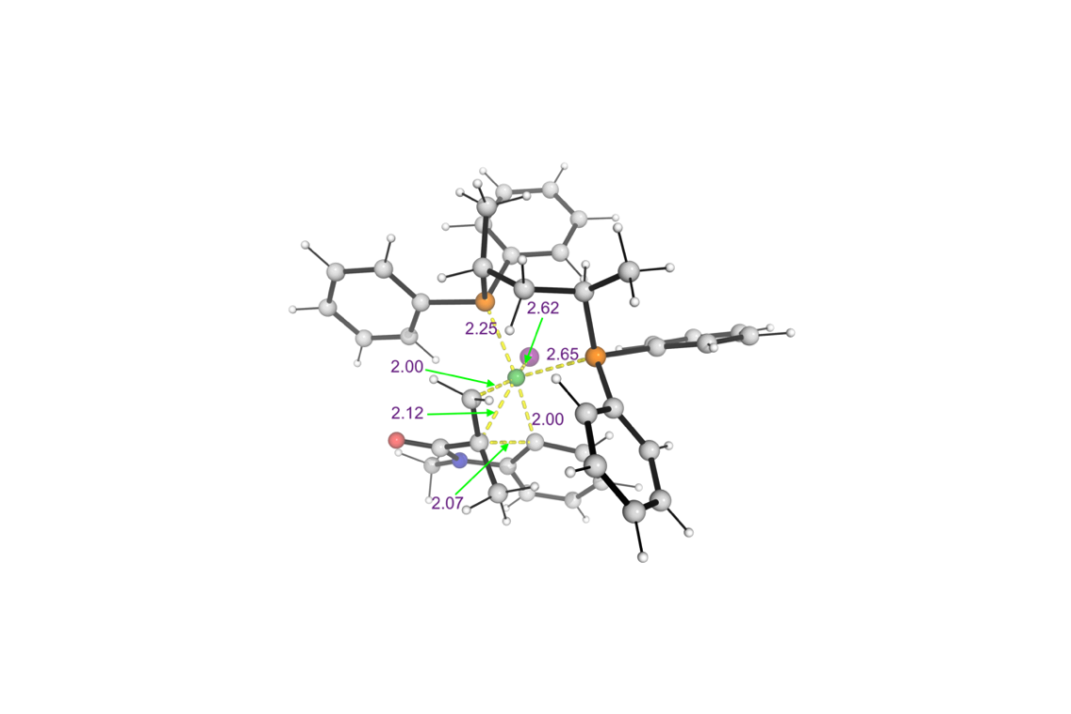 |
| **HOMO** | 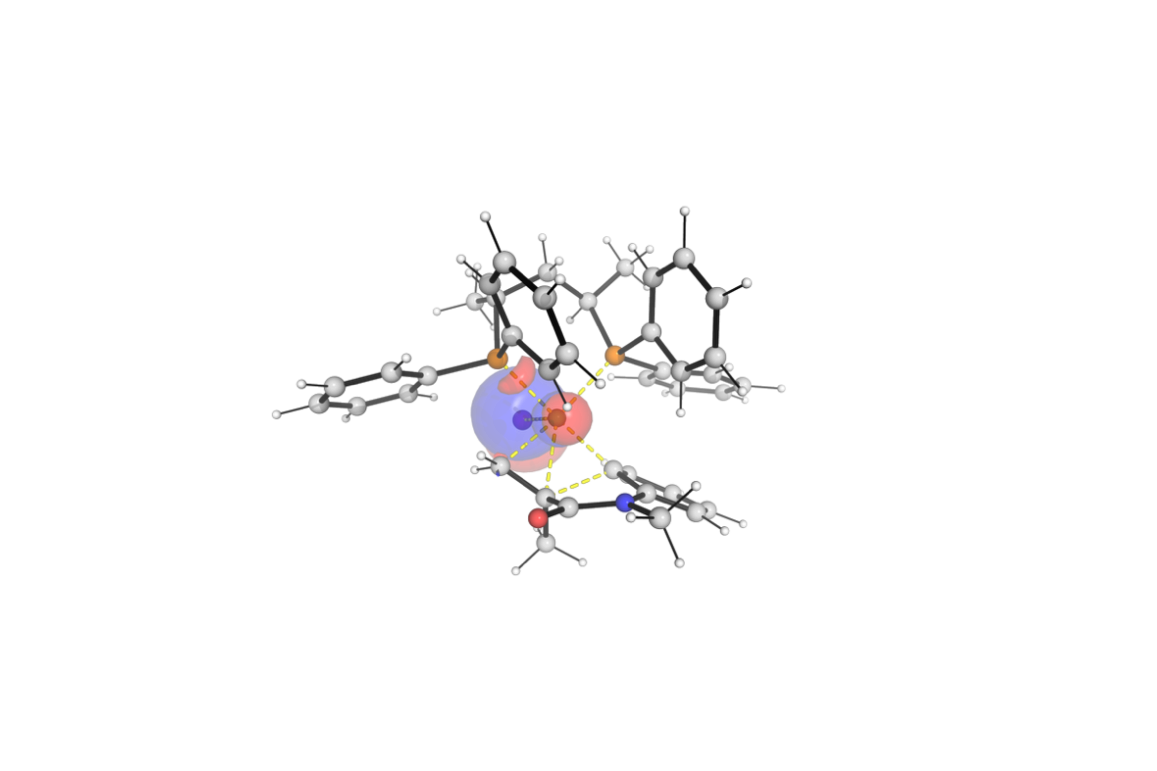 | 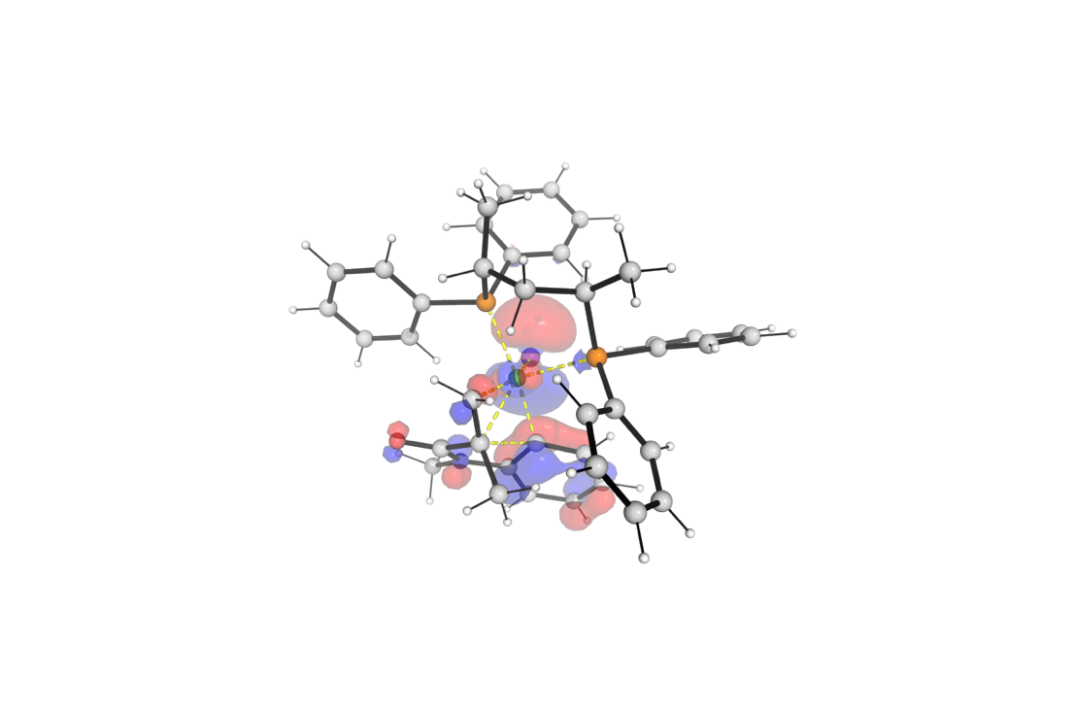 |
| **LUMO** | 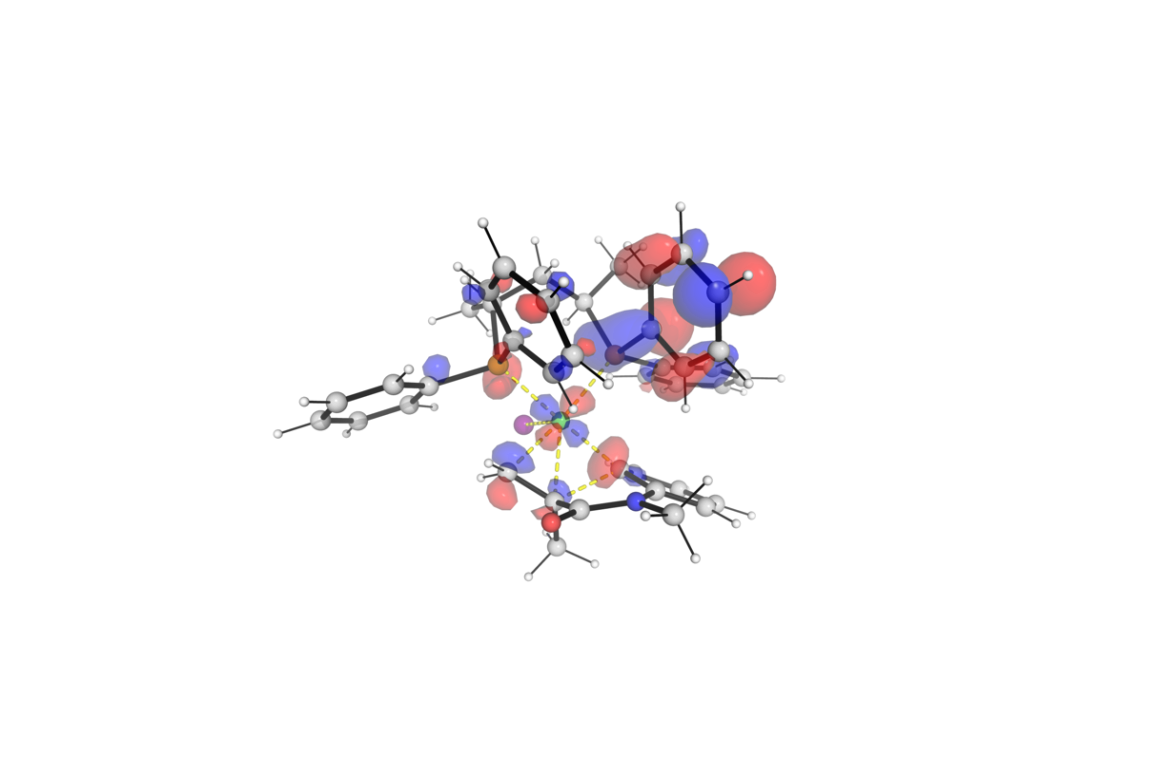 | 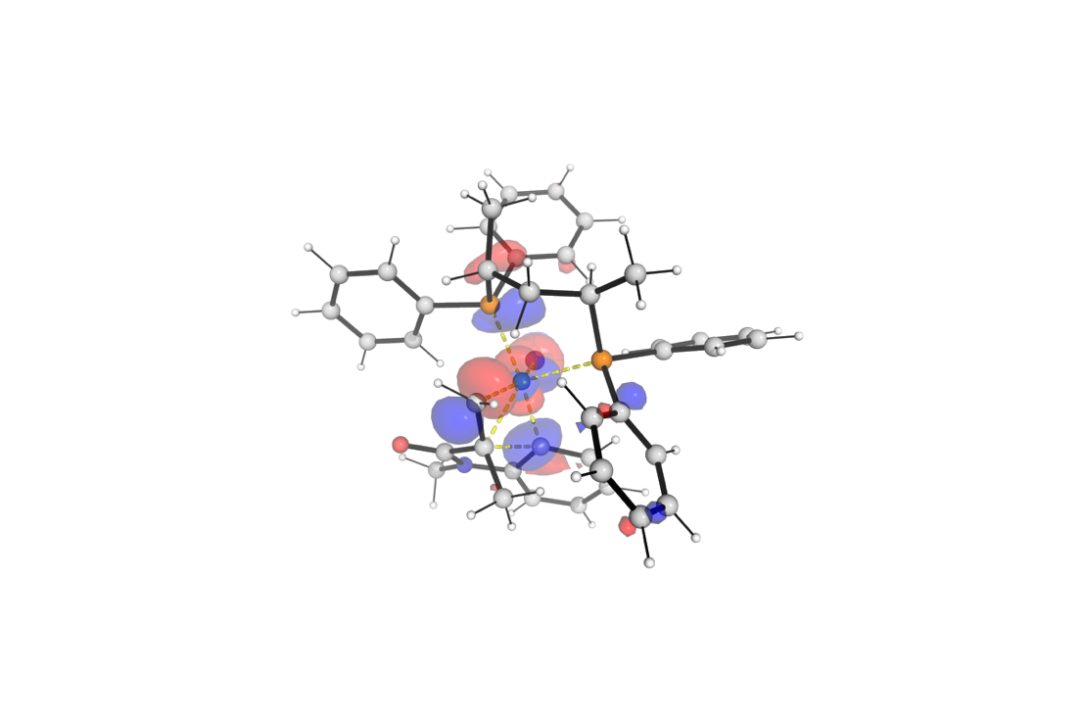 |
| **NCI** | 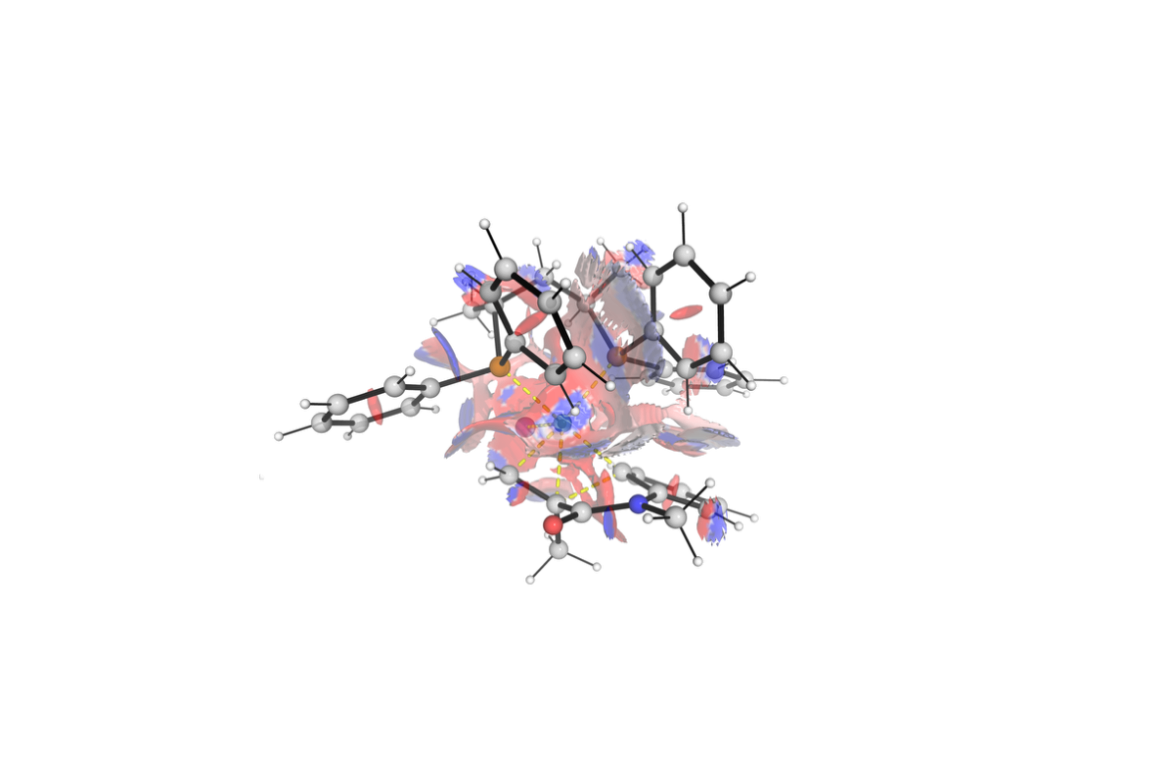 | 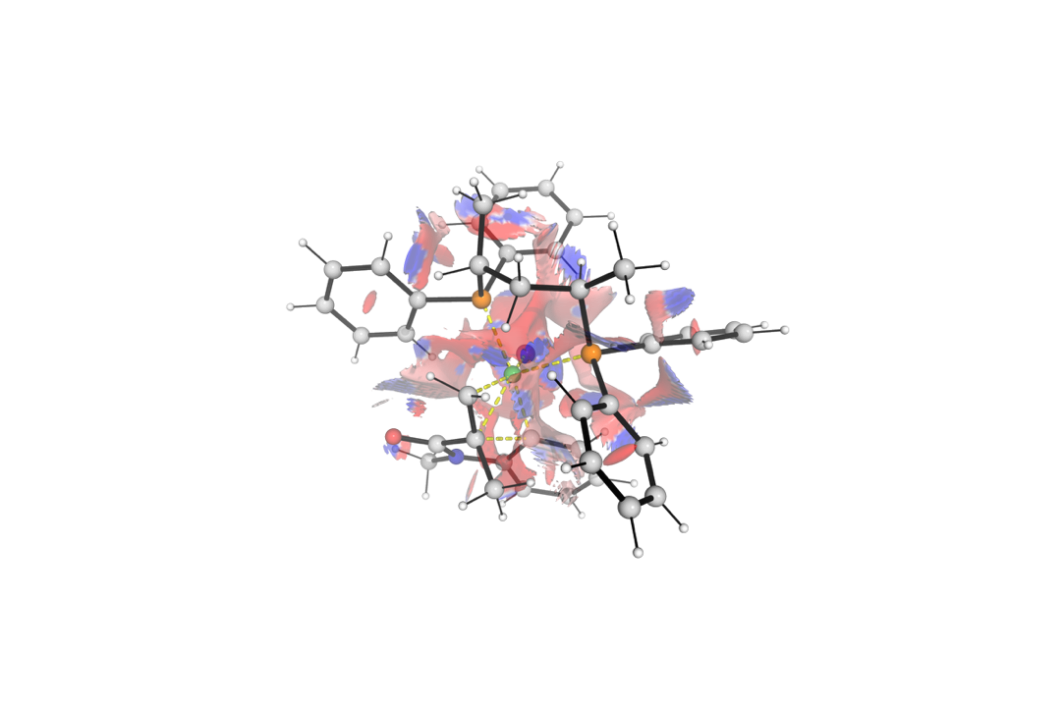 |
|  | 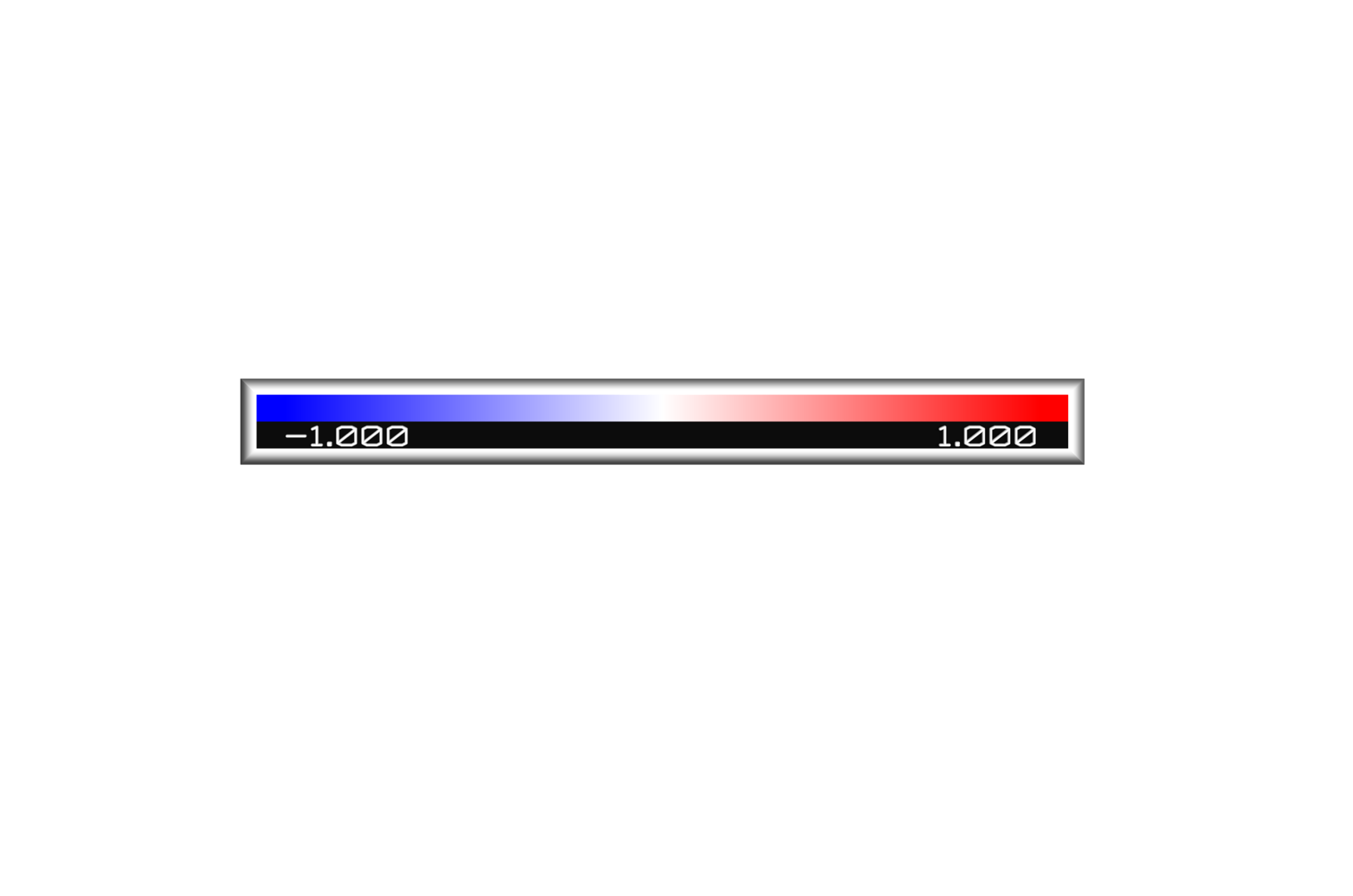 | |
|  | **TS2a-I-R (6m)** |  |
| **barrier** | ΔG^‡^ = -2.7 |  |
| **DFT**  **Structure** | 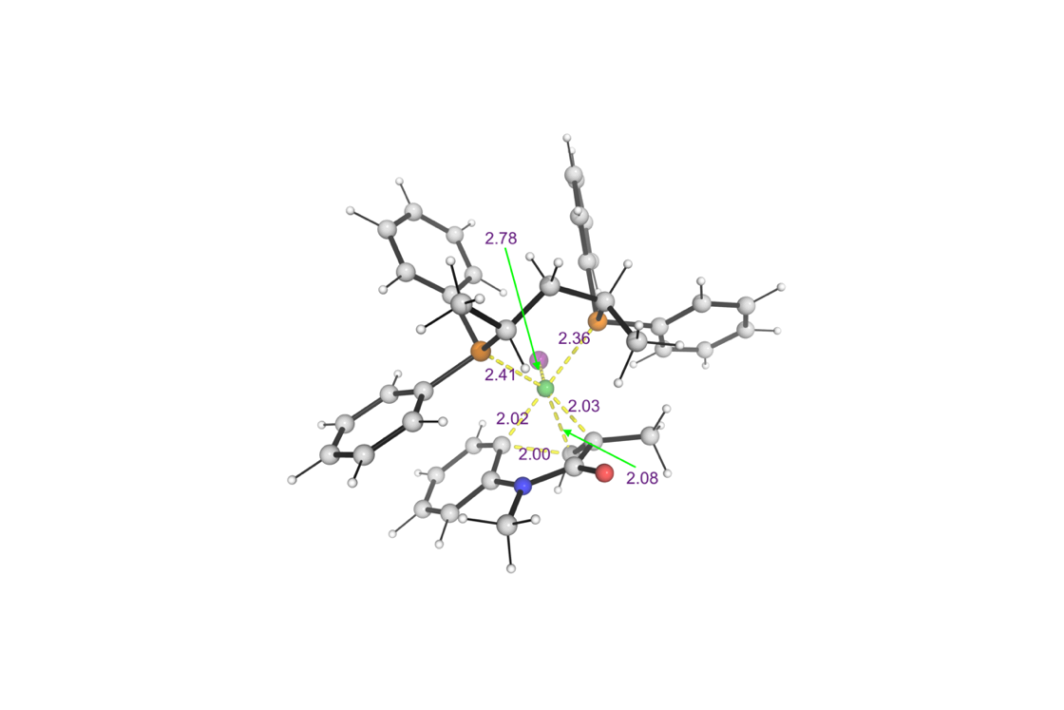 |  |
| **HOMO** | 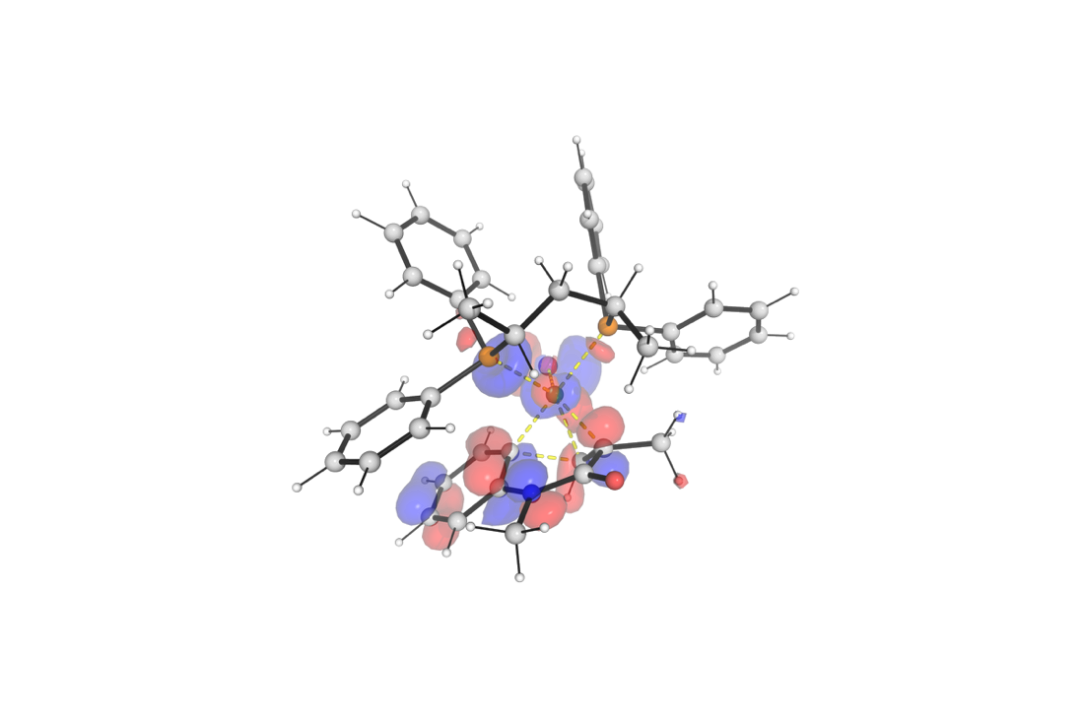 |  |
| **LUMO** | 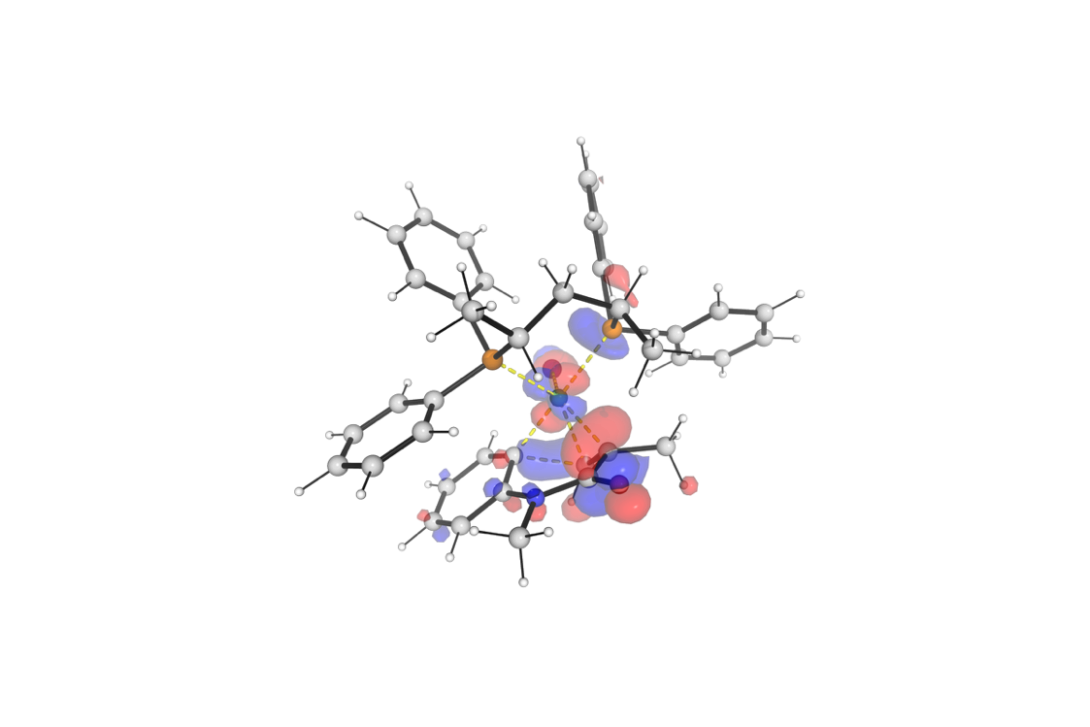 |  |
| **NCI** | 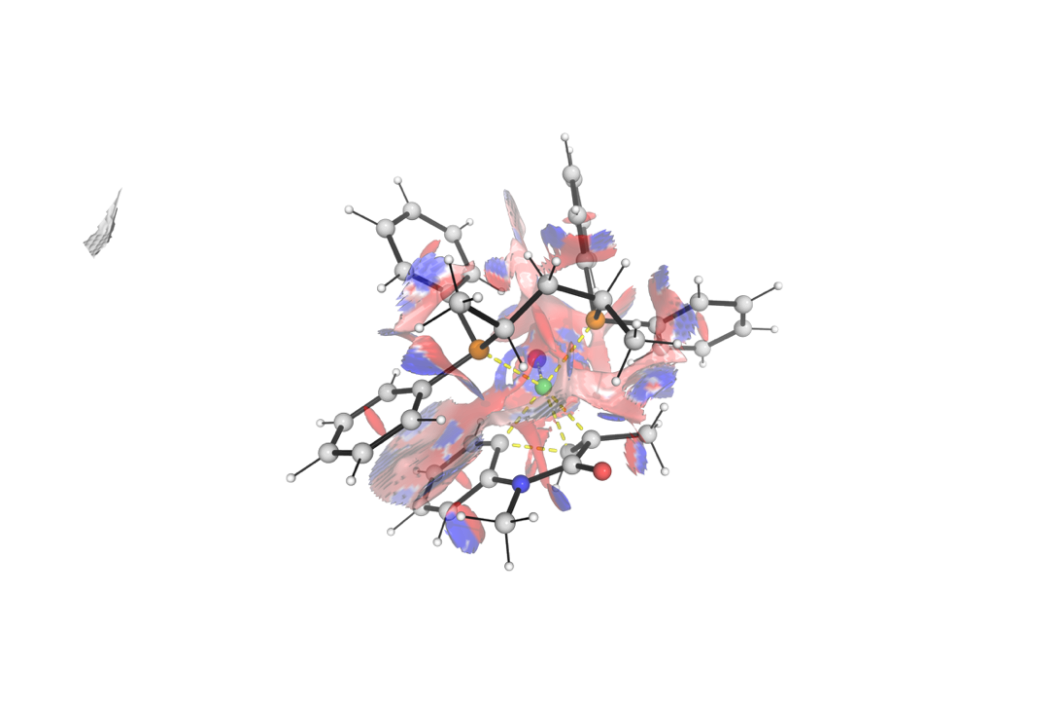 |  |
|  | 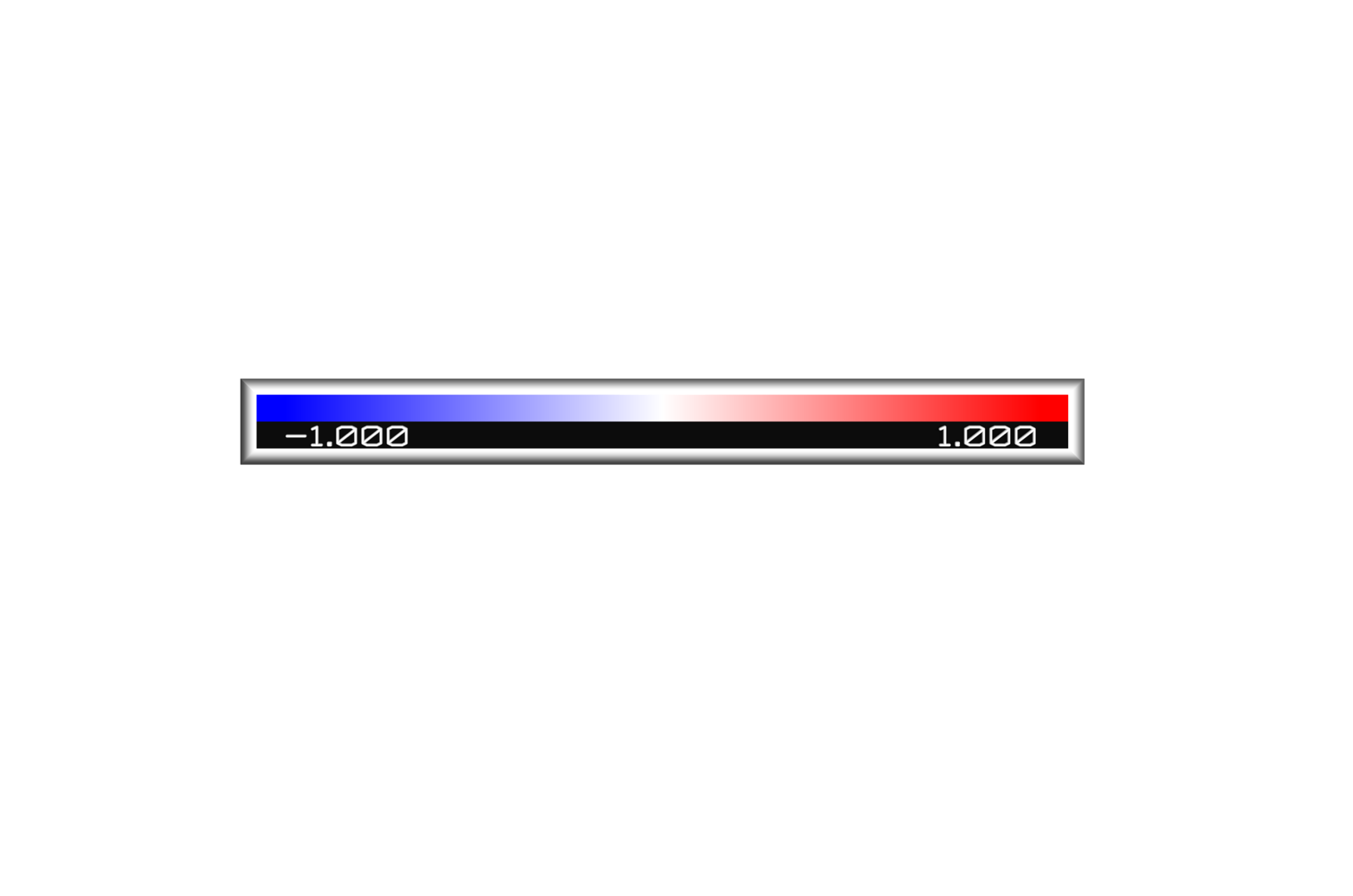 | |

**Figure S6.** DFT-optimized structures, frontier molecular orbitals (HOMO and LUMO) and non-covalent interaction (NCI) plots for all transition state for migratory insertion transition states (TSs) using aryl iodide as starting material.

For the migratory insertion step, that is, the Heck cyclization process, there are no distinct fragments that can be clearly defined before and after the reaction. Therefore, distortion–interaction analysis was not performed for the transition states in this step.

We propose that when the acrylamide side chain approaches the reaction site from the iodide side, it pushes the ligand in such a way that one of the phenyl rings of the ligand becomes aligned parallel to the aryl ring of the aryl iodide, leading to favourable π–π conjugative interaction, thereby stabilizing the transition state (Figure S6).

In contrast, when the acrylamide side chain approaches from the opposite direction, the ligand is displaced away from this region of potential π–π interaction, and the acrylamide side chain experiences steric repulsion from two phenyl rings of the ligand as well as the methyl group attached to one of its chiral centers (Figure S6). These combined steric effects form a cyclic congested region, resulting in a higher energy barrier and a less stable TS structure.

**7.3.4 Estimation of R/S product ratio under kinetic control**

Under kinetic control, the product ratio of two competing pathways can be estimated from the ratio of their reaction rates. The barrier difference (*ΔΔG^‡^*) between two competing transition states determines the kinetic preference for the major product over the minor one.

According to the transition state theory (TST), the rate constant (*k*) can be expressed by the Eyring equation:


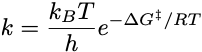


where *k_B_* is the Boltzmann constant, *h* is Planck’s constant, *R* is the gas constant, *T* is the temperature, and *ΔG^‡^* is the activation free energy.

For two competing pathways A and B, the ratio of their rate constants is given by:


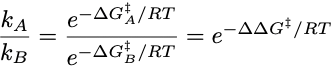


Here, *kₓ* is the rate constant of pathway X (X = A or B), *ΔGₓ^‡^* is the activation barrier for pathway X, and *ΔΔG^‡^* denotes the difference in barrier heights between the two competing transition states. The pre-exponential factor in the Eyring equation cancels when comparing the ratio of rate constants.

After confirming that the overall reaction proceeds through a Heck cyclization followed by anion exchange, we applied the Eyring equation to estimate the ratio of the two enantiomeric products by identifying the competing reaction pathways.

As discussed earlier, the step from **TS1** to **INT2** is highly thermodynamically exergonic, indicating that this process is essentially irreversible. Moreover, interconversion between the post-oxidative insertion intermediates (e.g., **INT2a-I** and **INT2c-I**) is prohibited by substantial steric hindrance. Therefore, **TS1a-I** and **TS1c-I** can be considered as the transition states of two distinct, competing reaction channels.

Subsequently, in the migratory insertion step responsible for chiral-center formation, each pathway further bifurcates into two competing TSs leading to the formation of opposite chiralities (*R* and *S*). Thus, a total of four reaction channels need to be considered in principle.

However, within each pair of **TS2** structures derived from the same **TS1**, a significant difference in activation barriers is observed. Taking the pair **TS2a-I-R** and **TS2a-I-S** as an example, their energy barriers differ by 12.2 kcal·mol⁻¹. Substituting this barrier difference into the Eyring equation at the reaction temperature of 308.15 K, the estimated rate ratio for the subsequent intermediates is k[*R*]/k[*S*] ≈ 4.6 × 10⁸, indicating that the formation of the *S*-configured intermediate is negligible. A similar trend is found for the other pair of competing **TS2** structures.

Consequently, after the formation of **INT2**, only one enantiomeric pathway effectively contributes to product formation. Therefore, the overall product ratio can be determined solely from the barrier difference between **TS1a-I** and **TS1c-I**, which represent the kinetically competing transition states leading to the two possible chiral products. Similar idea had been mentioned in other research.^21^Based on the above consideration, when reaction proceeds through **TS1a-I** to form the intermediate **INT2**, the final product can be regarded as pure *R*-configured. Conversely, when the reaction proceeds through **TS1c-I**, the final product can be regarded as pure *S*-configured.

Therefore, by comparing the barriers of **TS1a-I** and **TS1c-I**, the ratio of rate constants for forming the *R*-product and *S*-product can be estimated as k[*R*]/k[*S*] ≈ 7.1 at 308.15 K, corresponding to an 87.7% proportion of the *R*-enantiomer and a theoretical enantiomeric excess (ee) of approximately 75%. Following is the formula for calculating the theoretical enantiomeric excess (ee) value:


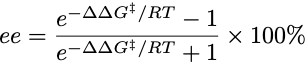


**7.4 Aryl Triflate as Starting Material**

**7.4.1 Gibbs energy profile**

The Gibbs energy profile for reaction using aryl triflate as starting material was prepared similar to **section 6.3.1**, as shown in Figure S7. It should be noticed that the reaction condition was different from aryl iodide’s reaction, the reaction temperature was 25 ºC in dimethyl sulfoxide solvent.

According to Figure S7, the oxidative addition step in this reaction is also highly exergonic, where Gibbs free energy of **INT2** decreases sharply relative to **TS1**, by approximately 61.3 kcal·mol⁻¹.Such phenomenon supports the assumption that this step is irreversible. Moreover, unlike the case of aryl iodide, this reaction prefers anion exchange prior to the Heck cyclization. The transition state for migratory insertion without anion exchange exhibits a barrier that is 32.4 kcal·mol⁻¹ higher than that of the pathway involving anion exchange followed by migratory insertion.

For the reaction employing aryl triflate as the starting material, the preferred mechanism proceeds through oxidative addition, followed by anion exchange that replaces the leaving group with a deprotonated phosphine oxide, then Heck cyclization to establish the chiral center, and finally reductive elimination to afford the product.


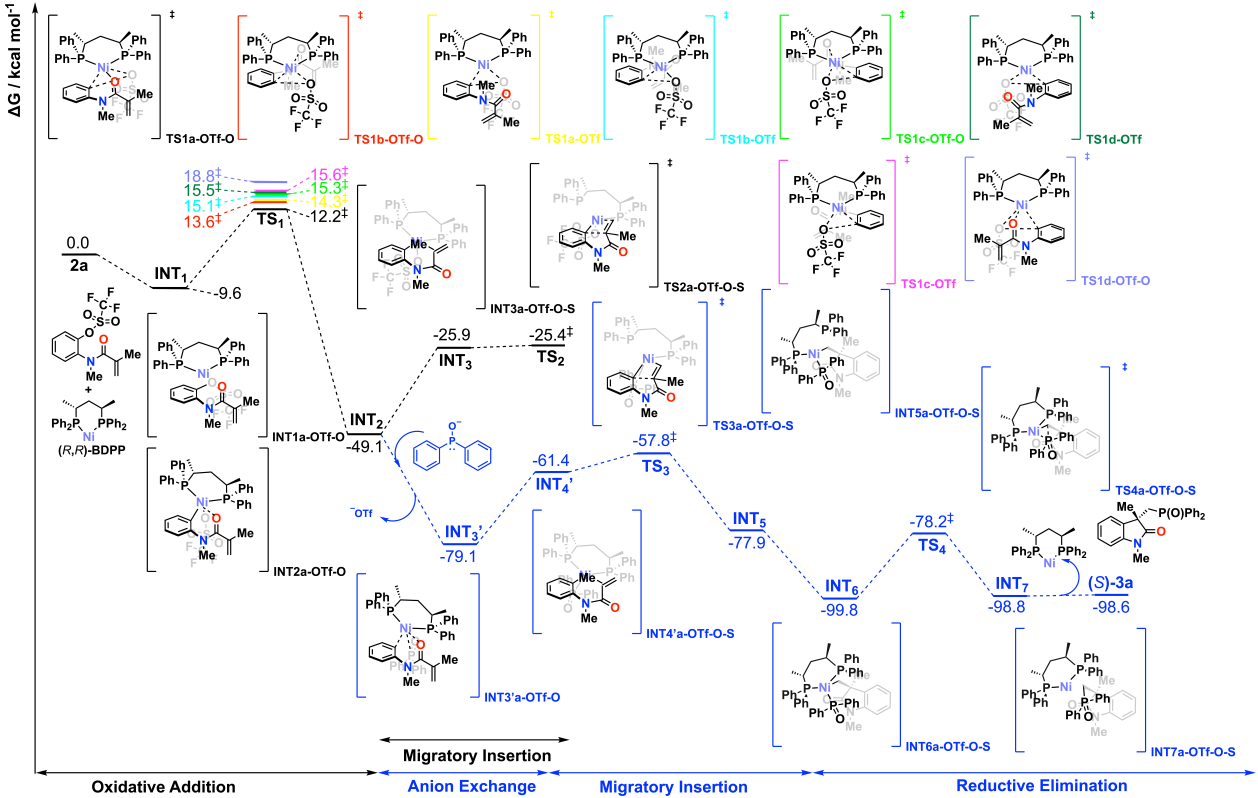


**Figure S7.** Gibbs energy profile for reaction using aryl triflate as starting material. Gibbs energies are given in SMD(dimethyl sulfoxide)-MN15/def2-QZVP//MN15/def2-SVP level of theories.

**7.4.2 Competing transition states for oxidative addition step**

The 8 transition state structures of oxidative addictive step are shown in Figure S8.

| **TS1a-OTf** | **TS1a-OTf-O** |
| --- | --- |
| ΔG^‡^ = 14.3 | ΔG^‡^ = 12.2 |
| 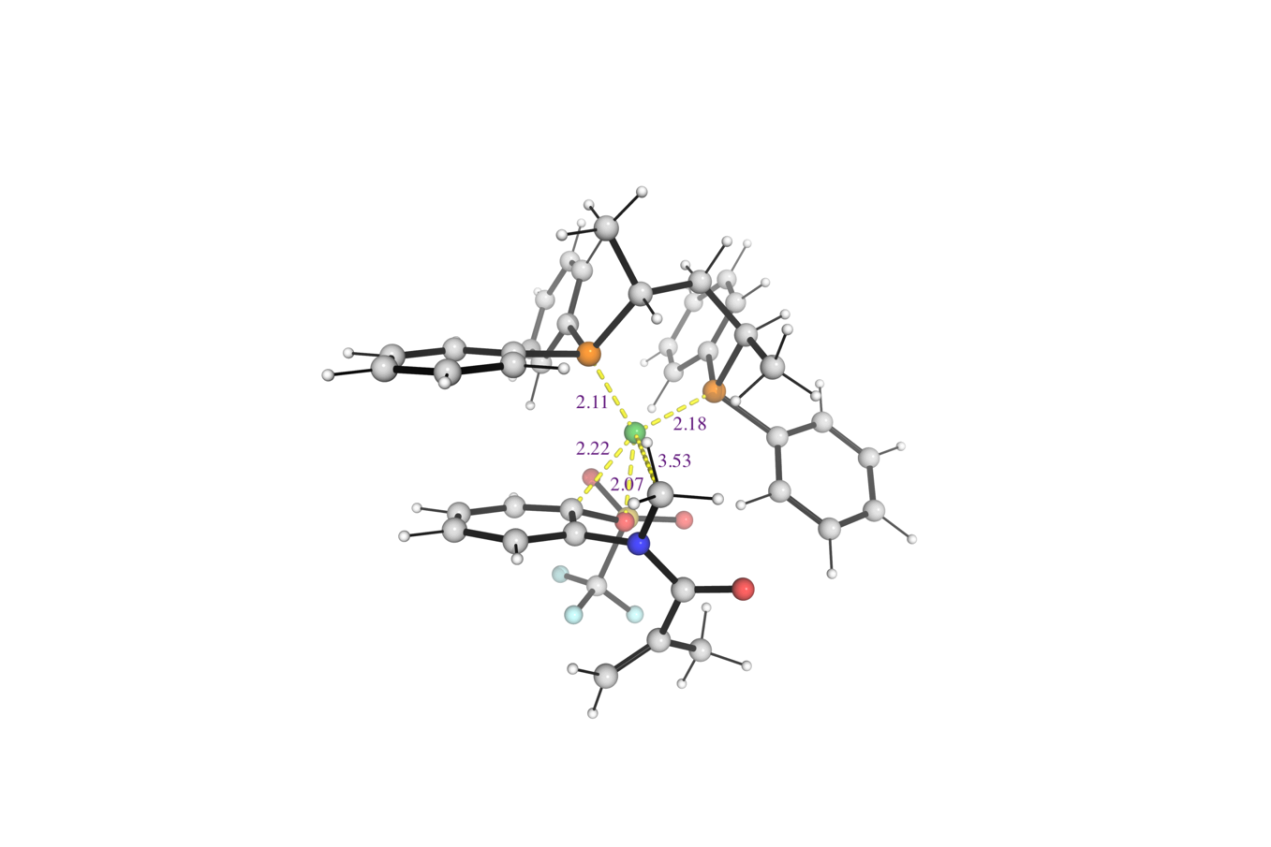 | 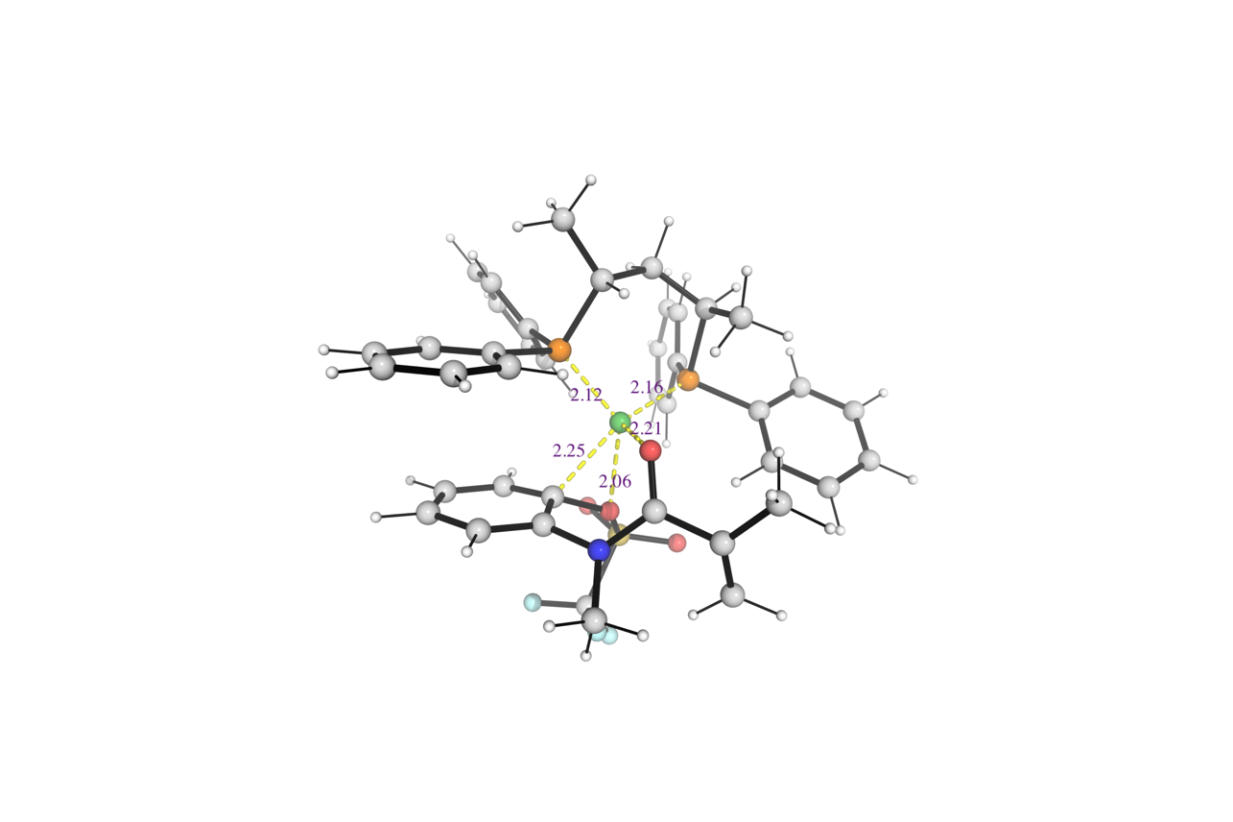 |
| **TS1b-OTf** | **TS1b-OTf-O** |
| ΔG^‡^ = 15.1 | ΔG^‡^ = 13.6 |
| 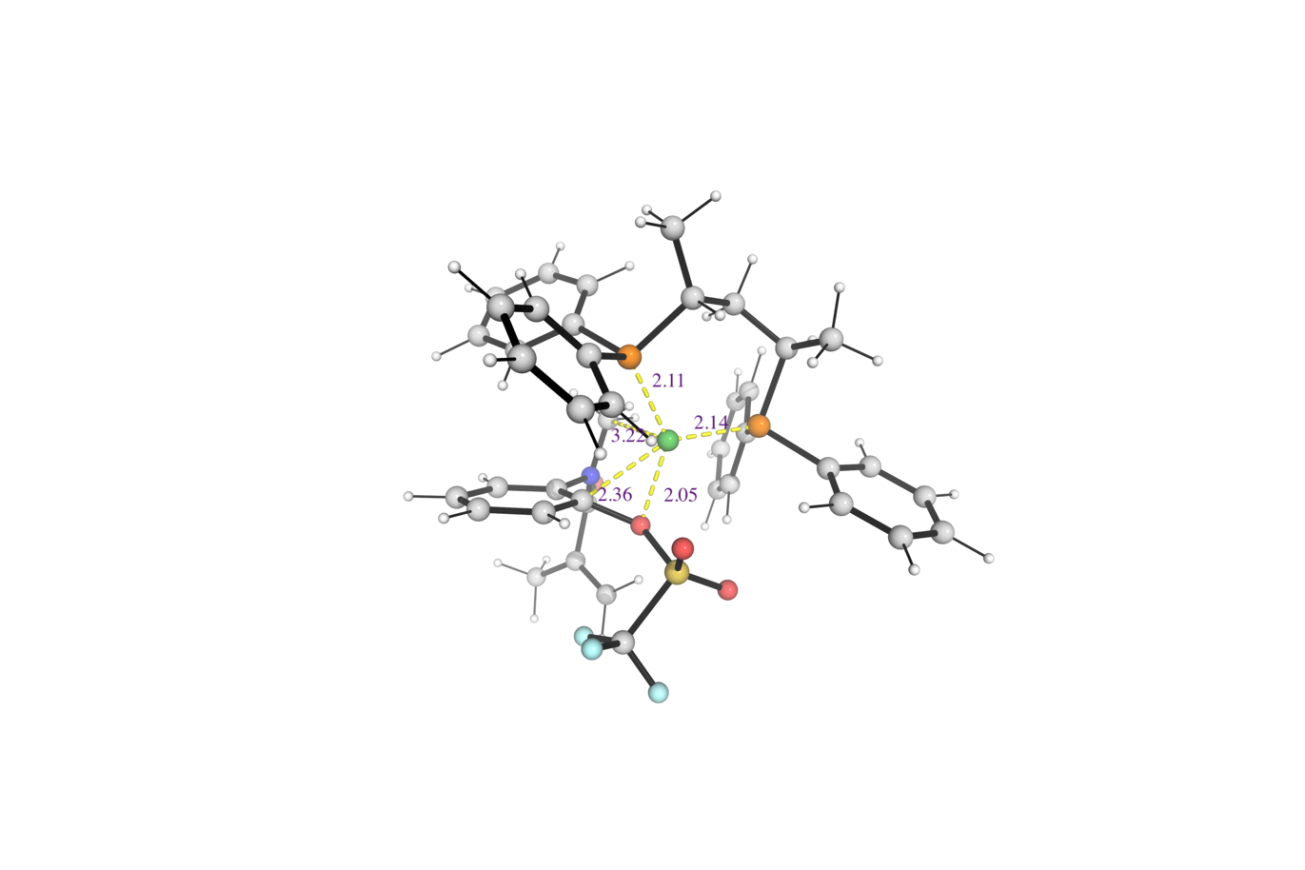 | 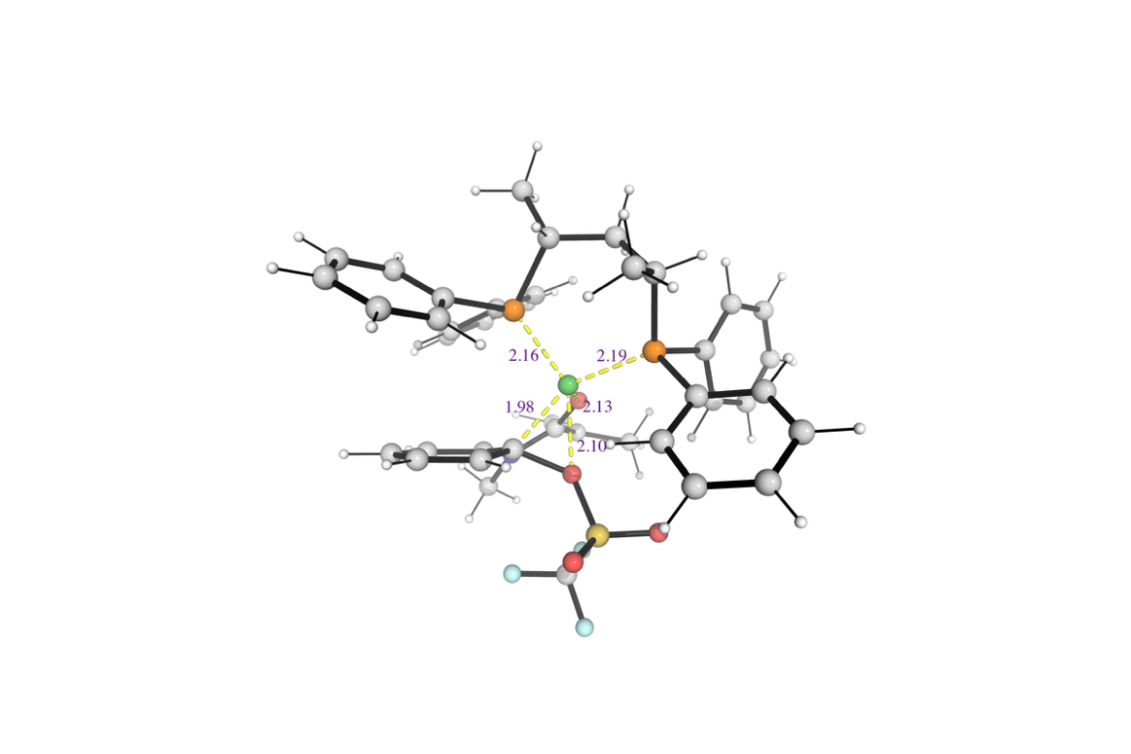 |
| **TS1c-OTf** | **TS1c-OTf-O** |
| ΔG^‡^ = 15.6 | ΔG^‡^ = 15.3 |
| 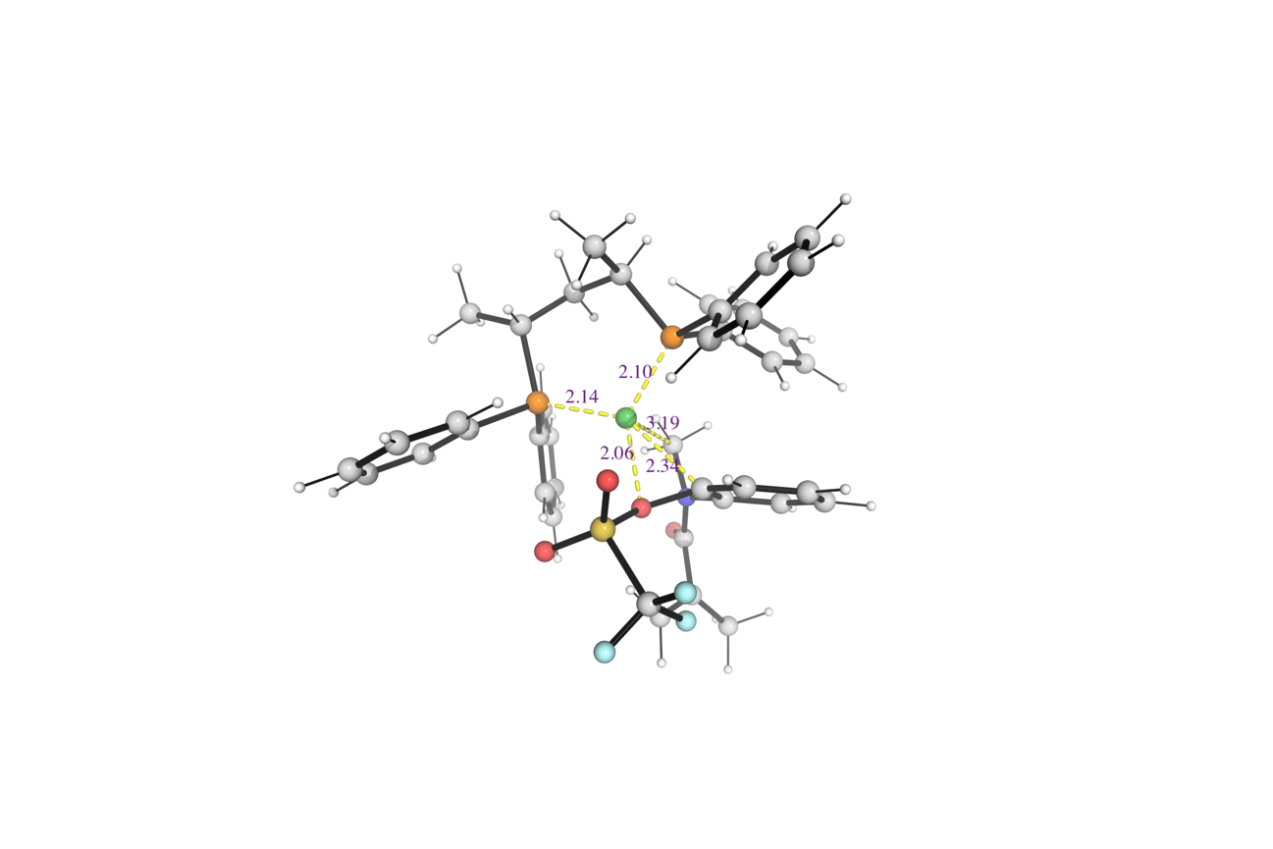 | 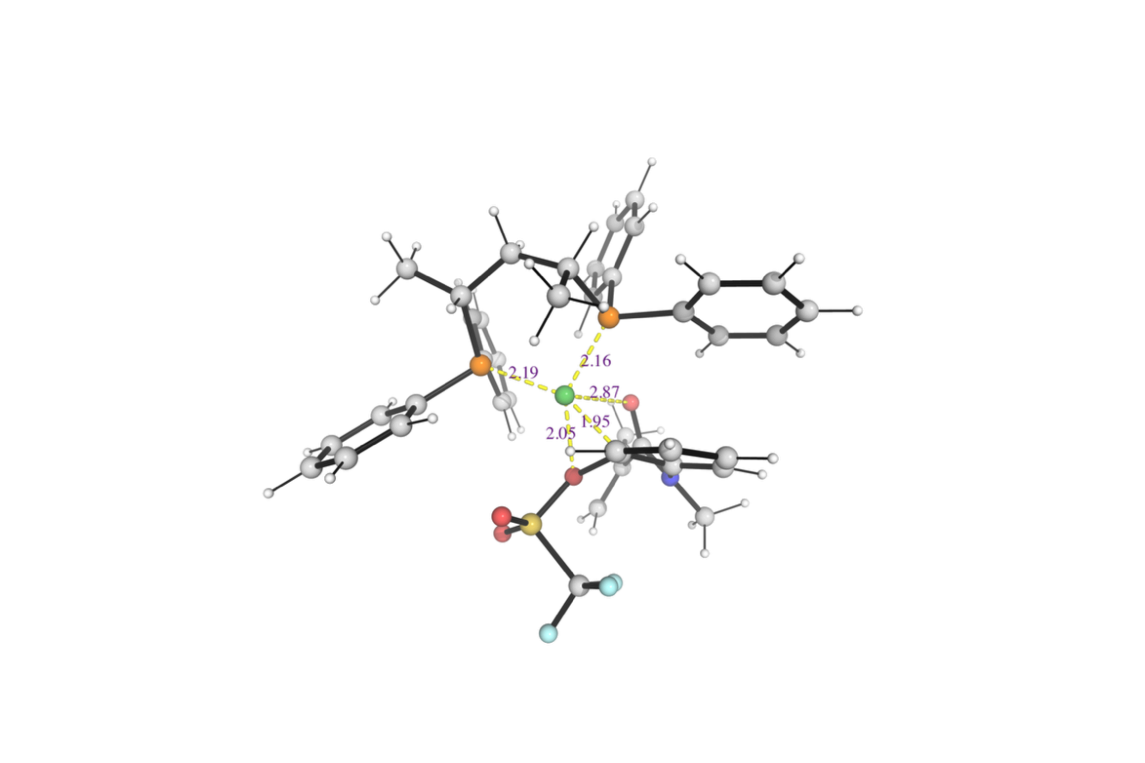 |
| **TS1d-OTf** | **TS1d-OTf-O** |
| ΔG^‡^ = 15.5 | ΔG^‡^ = 18.8 |
| 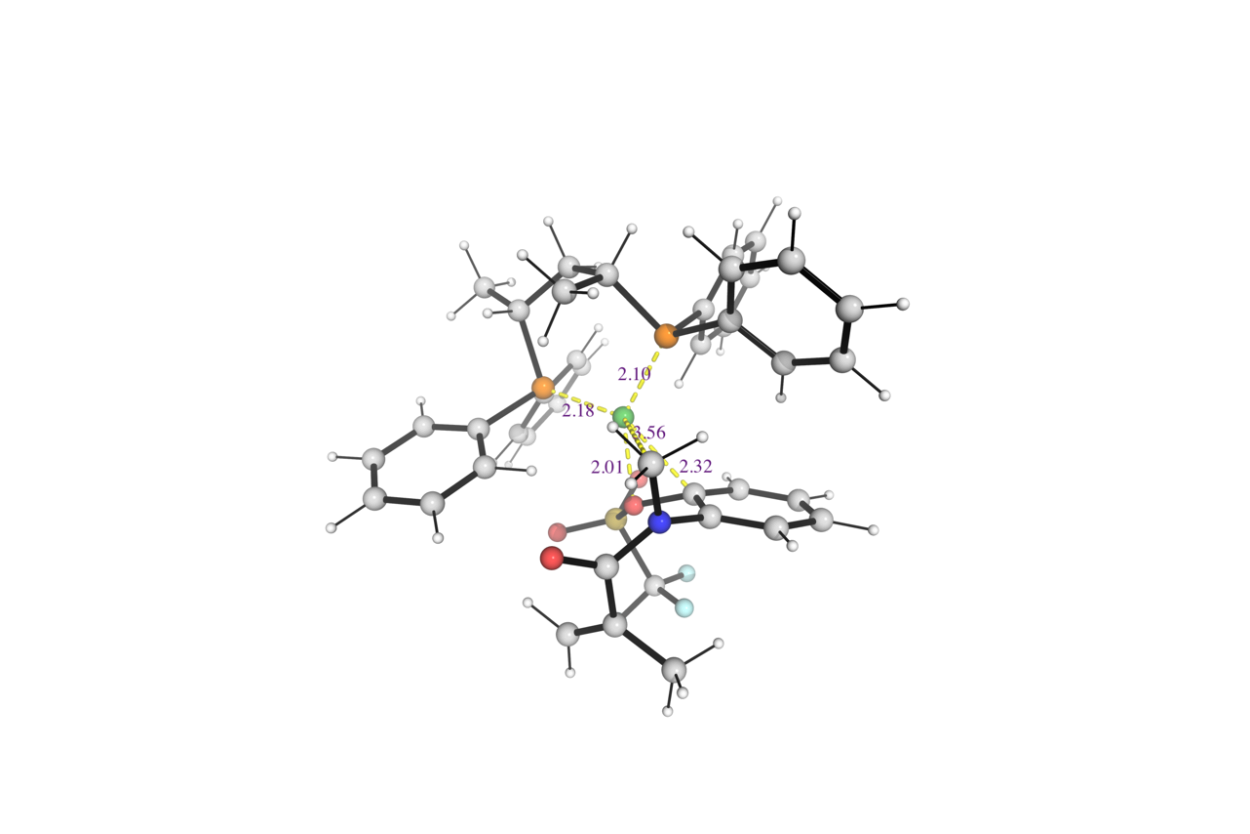 | 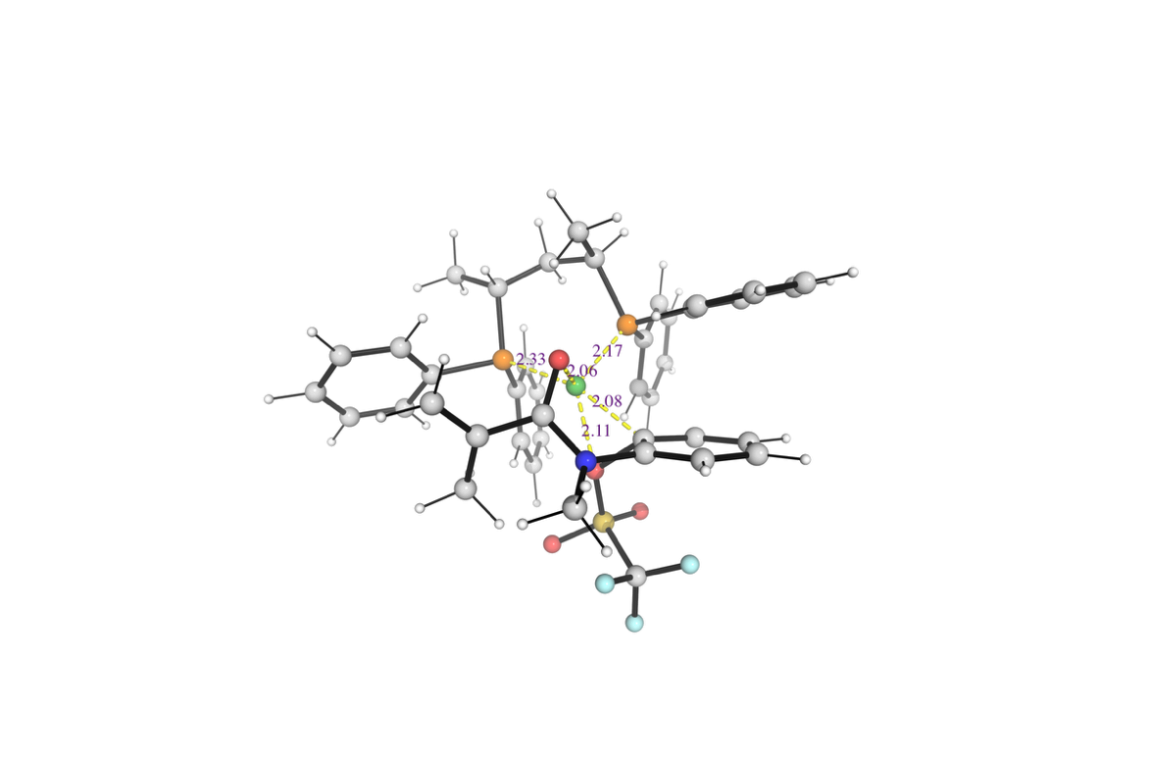 |

**Figure S8.** DFT-optimized structures of different conformers of Ni insertion (oxidative addition) transition states (**TS1**s) using aryl triflate as starting material. Gibbs free energies are given relative to the ground state of the combined reactants and ligand. All Gibbs energies were calculated at the SMD(dimethyl sulfoxide)–MN15/def2-QZVP//MN15/def2-SVP level of theories.

FMOs, NCI and distortion-interaction analysis in the lowest energy competing TSs, **TS1a-OTf-O**, **TS1a-OTf**, **TS1b-OTf-O** and **TS1b-OTf** were further conducted to analyze the factors influencing the barriers. Results are shown in Figure S9 and Table S2.

|  | **TS1a-OTf** | **TS1a-OTf-O** |
| --- | --- | --- |
| **barrier** | ΔG^‡^ = 14.3 | ΔG^‡^ = 12.2 |
| **DFT**  **Structure** | 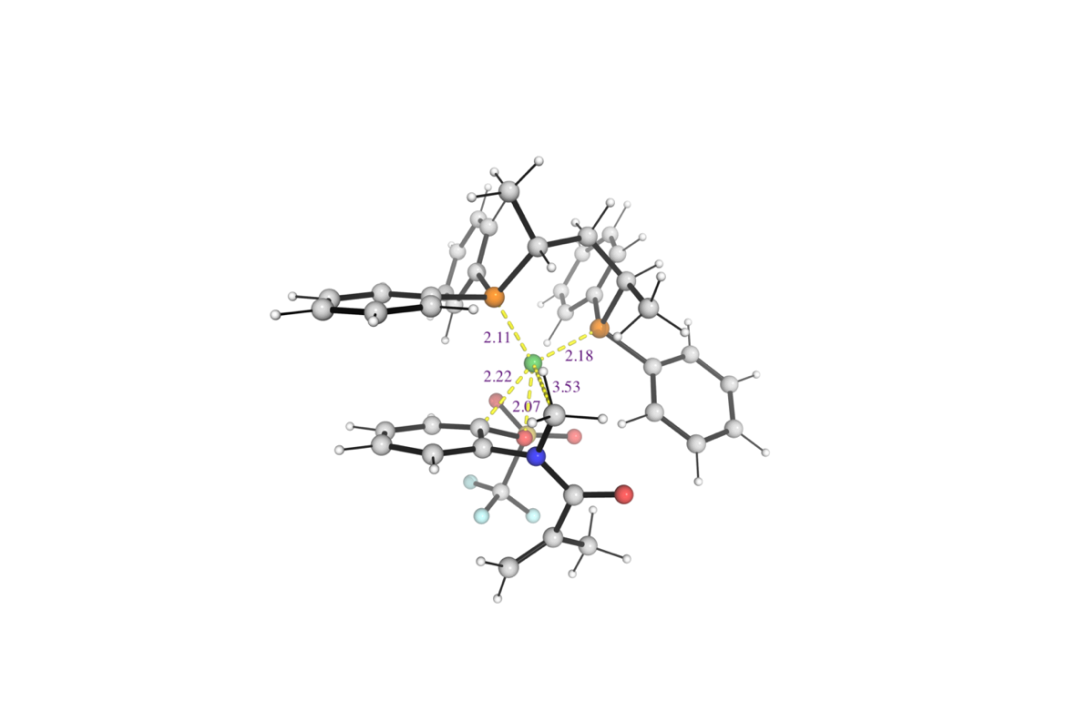 | 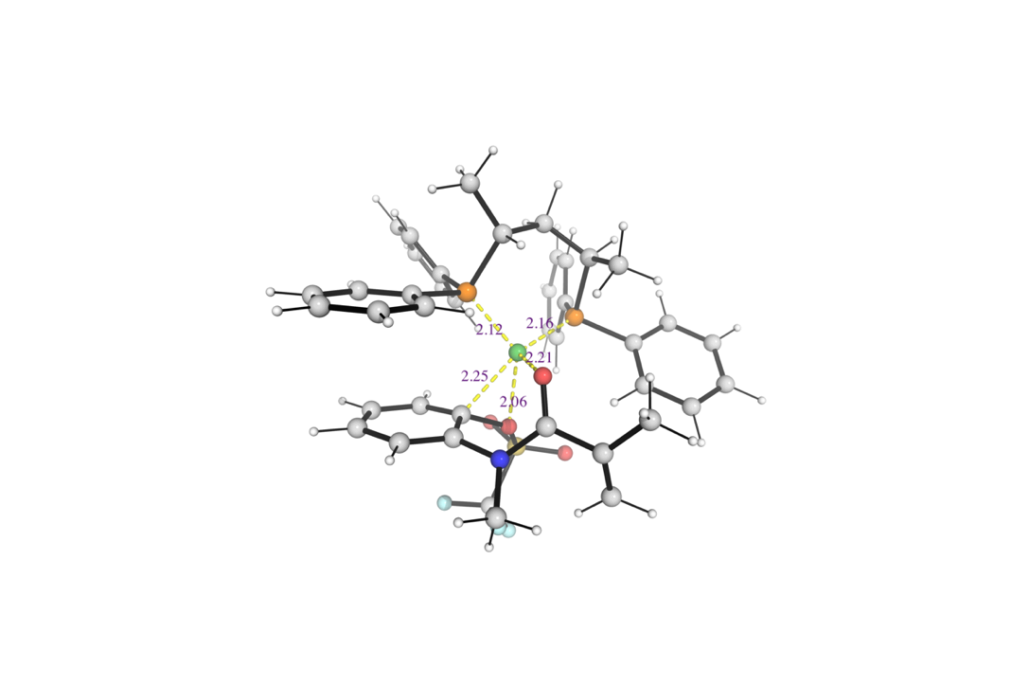 |
| **HOMO** | 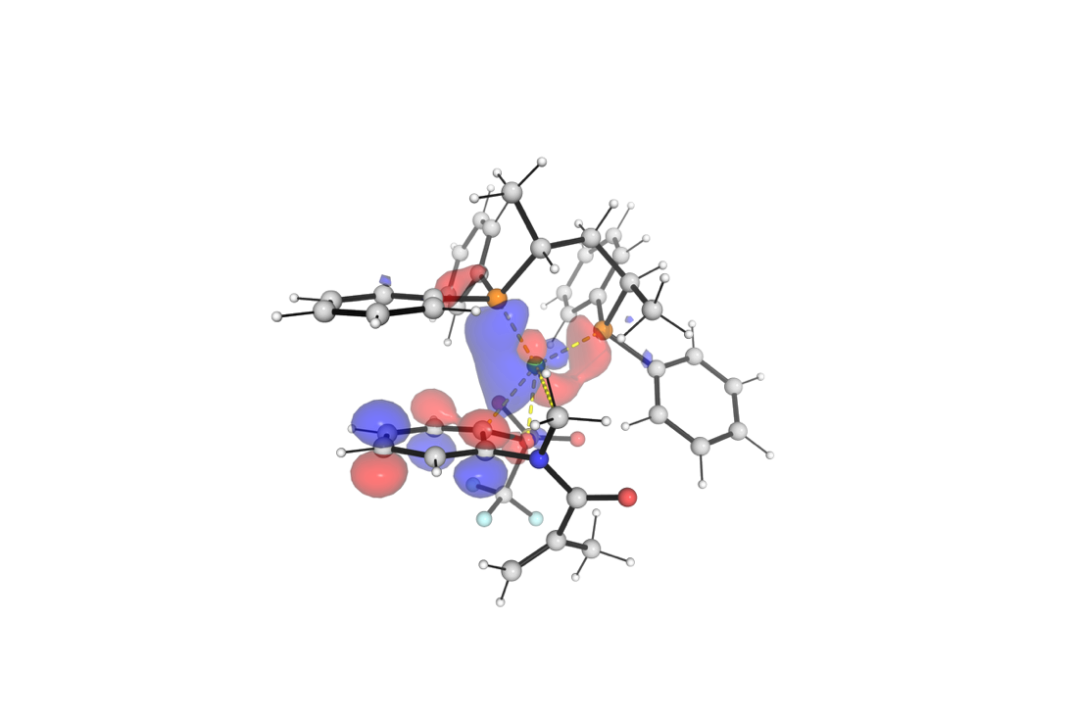 | 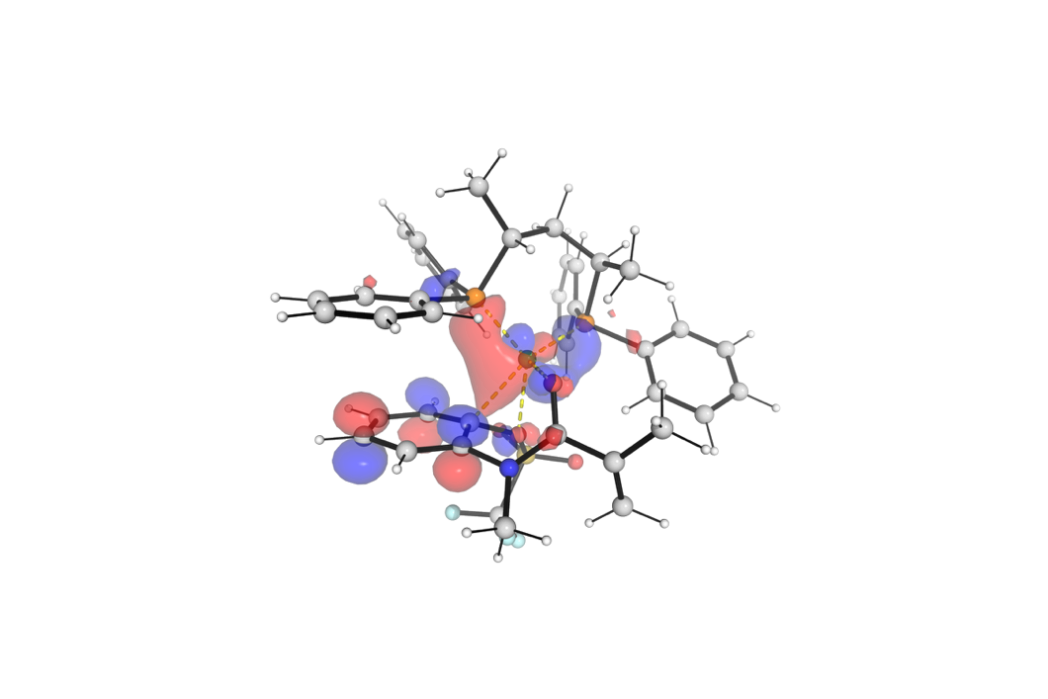 |
| **LUMO** | 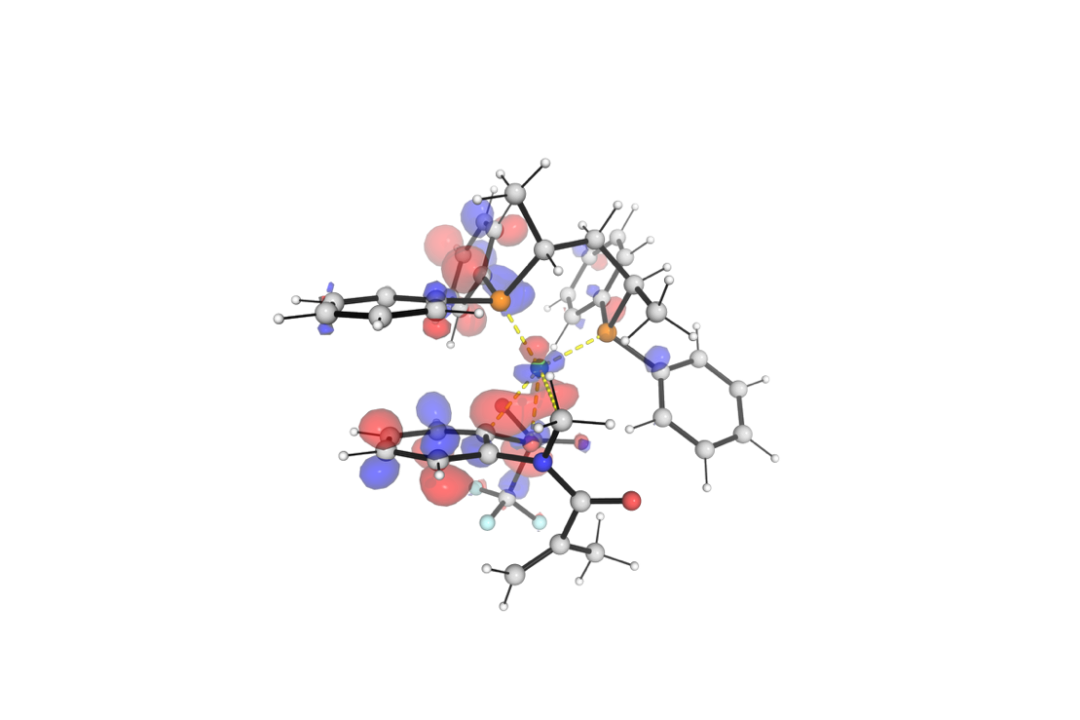 | 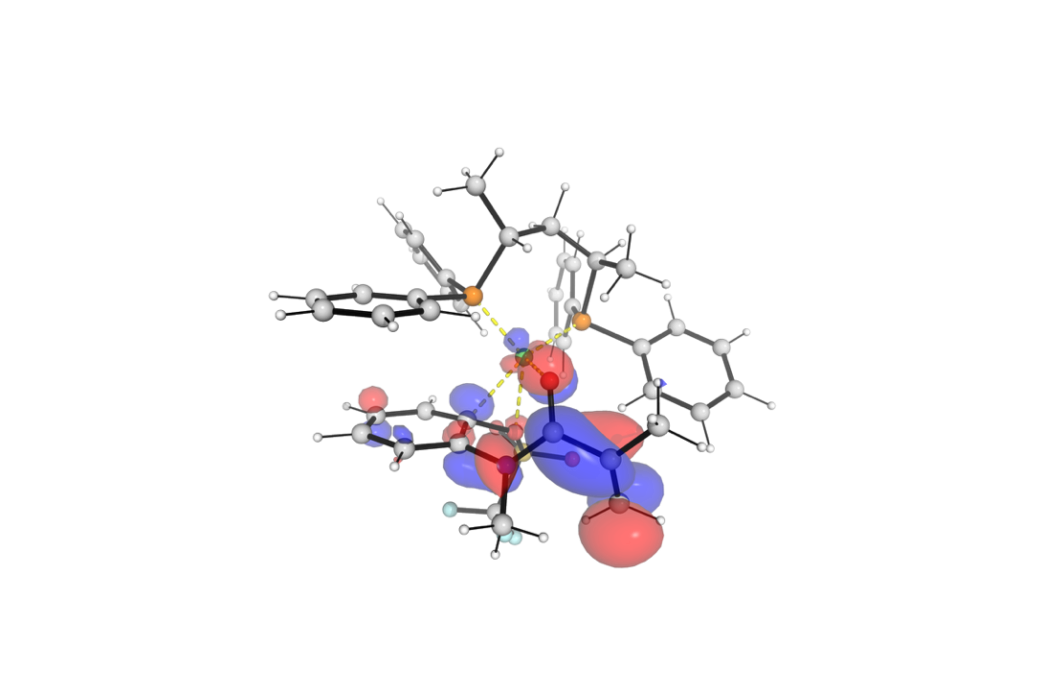 |
| **NCI** | 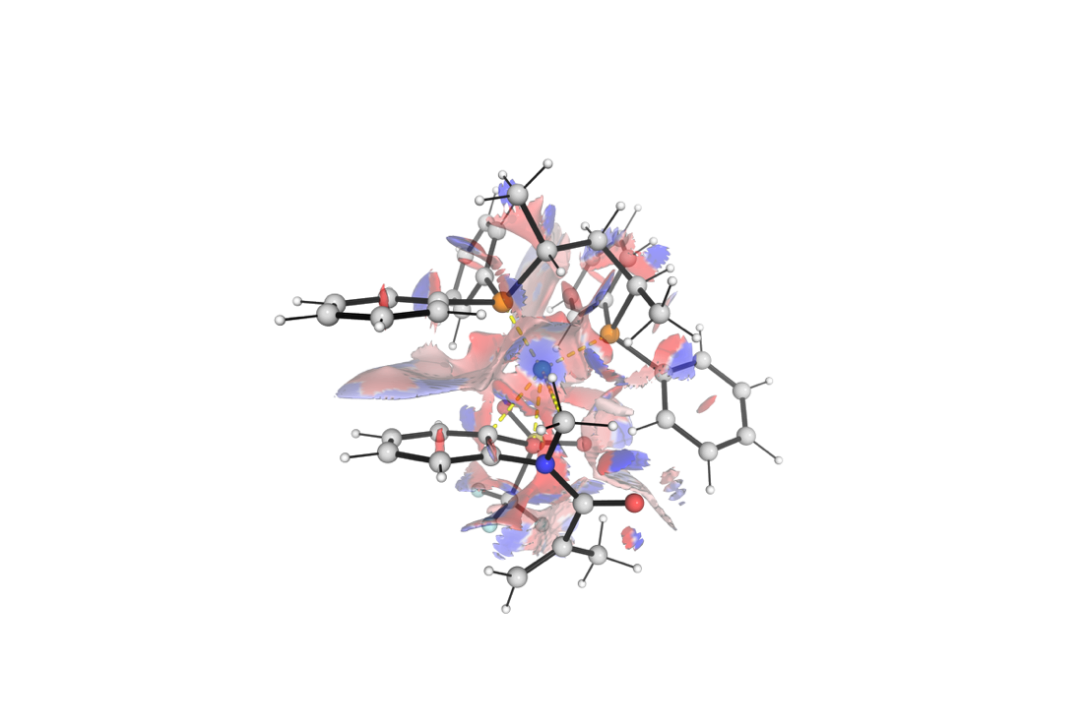 | 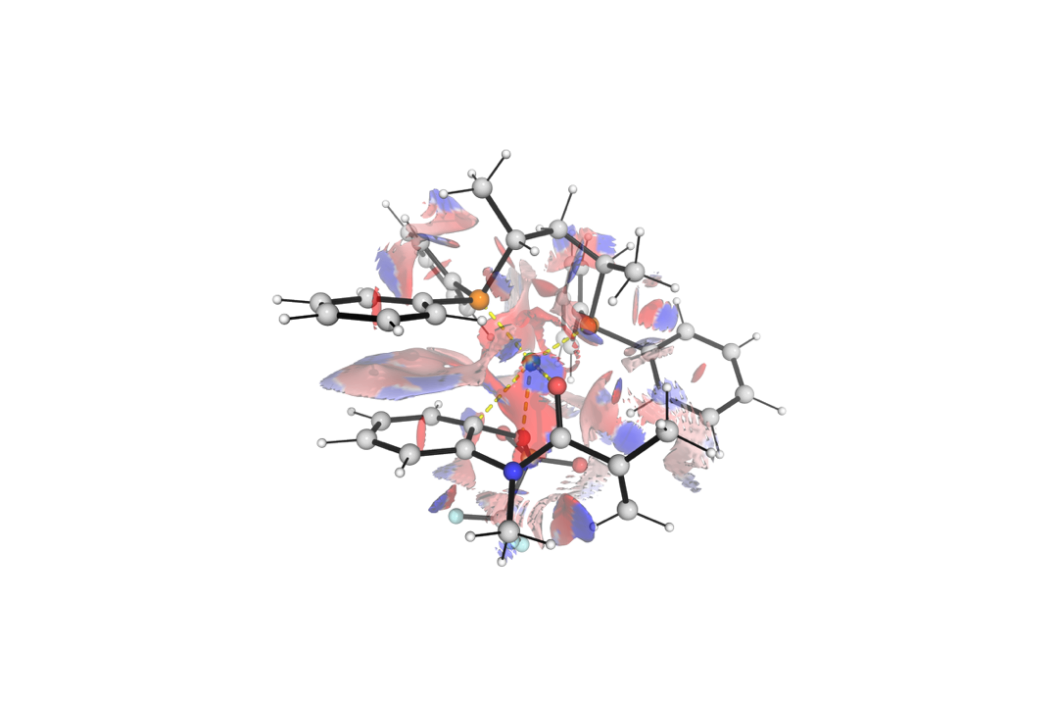 |
|  | 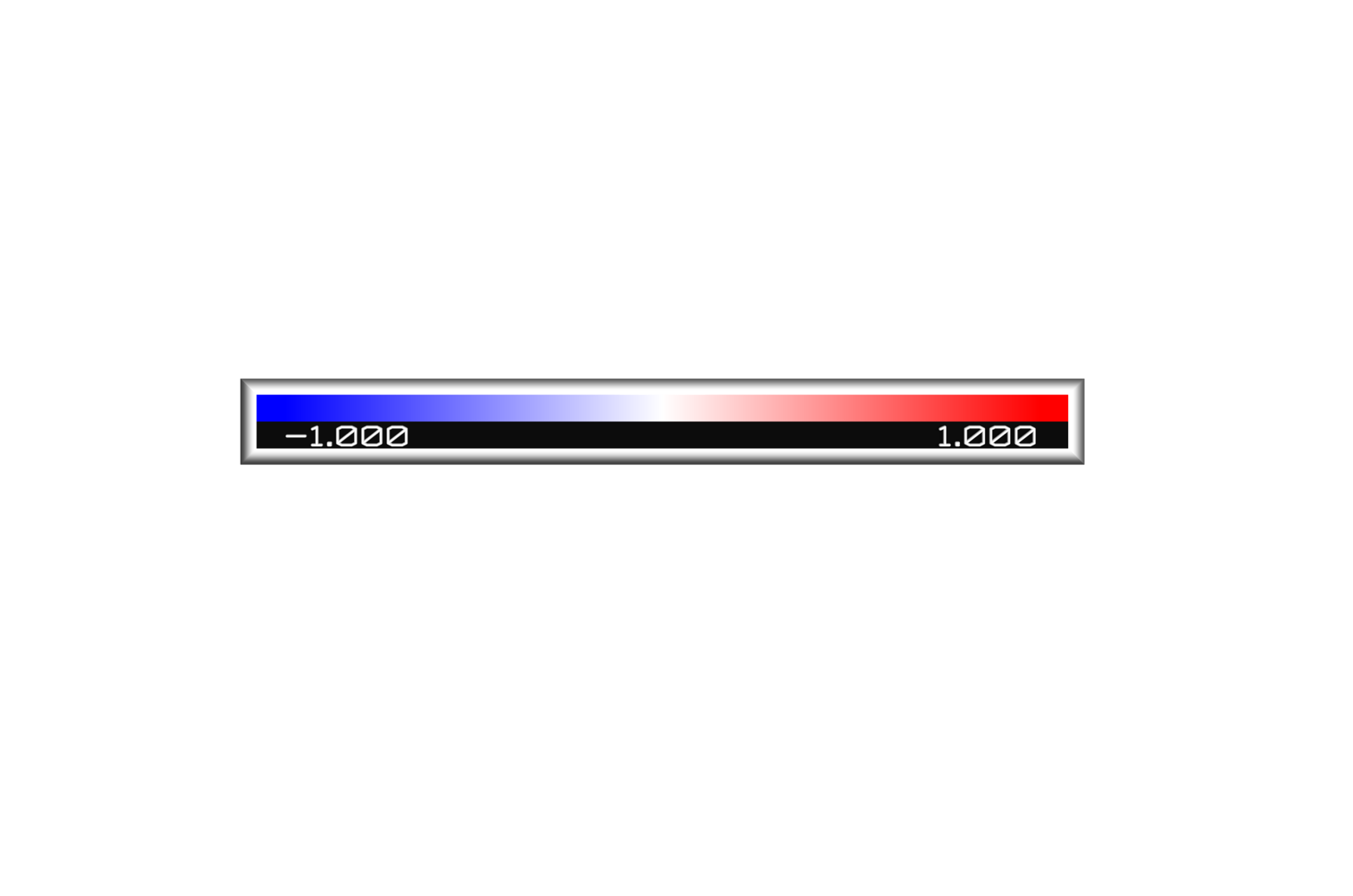 | |
|  | **TS1b-OTf** | **TS1b-OTf-O** |
| **barrier** | ΔG^‡^ = 15.1 | ΔG^‡^ = 13.6 |
| **DFT**  **Structure** | 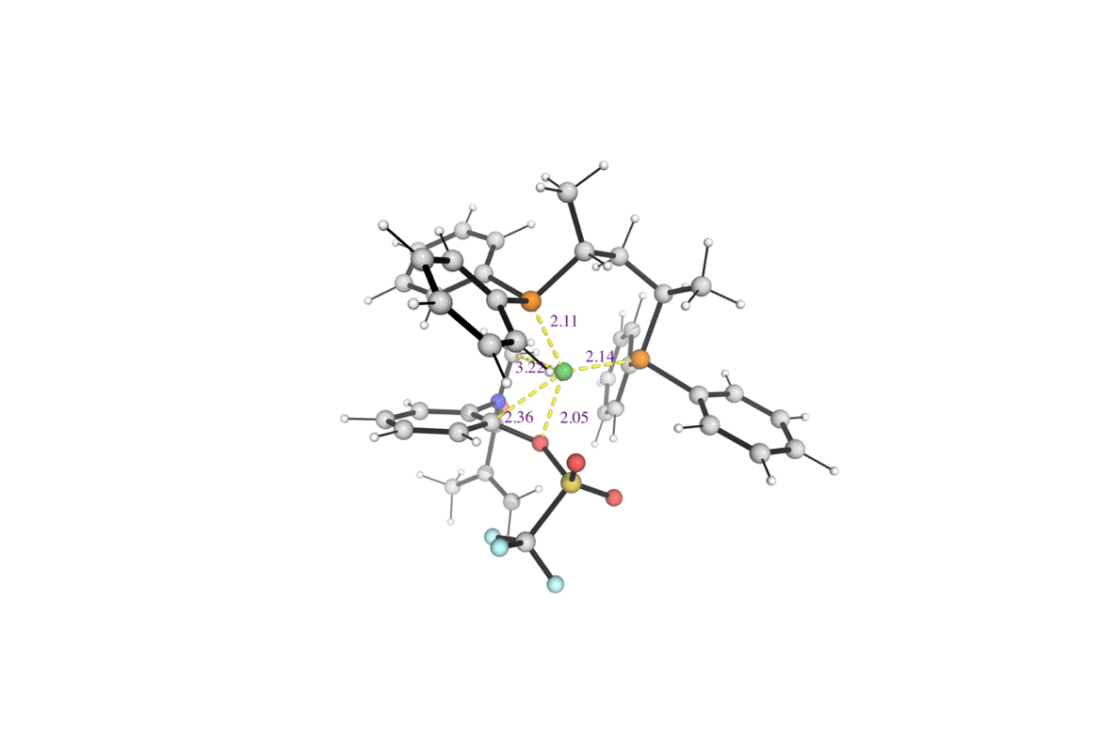 | 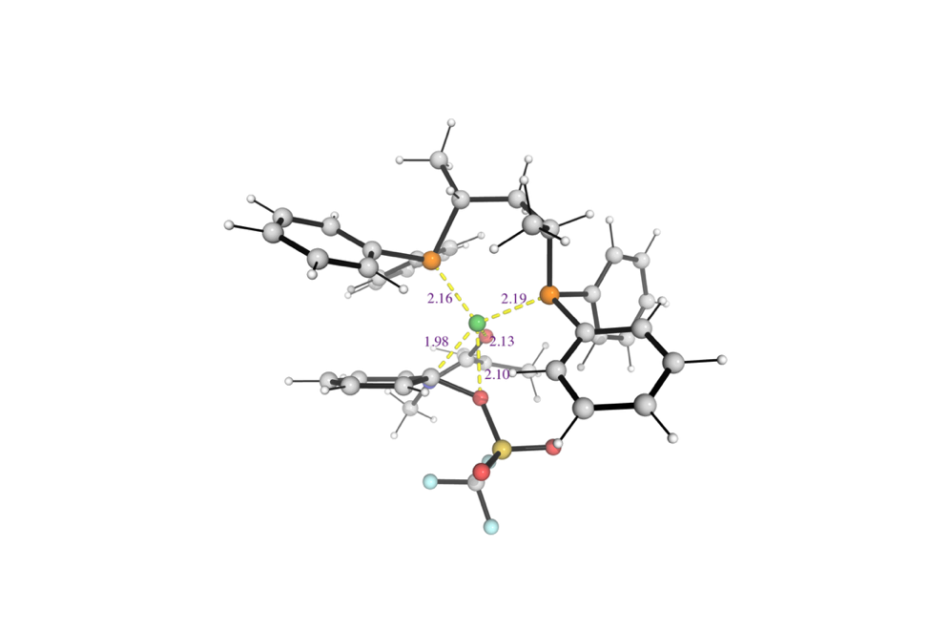 |
| **HOMO** | **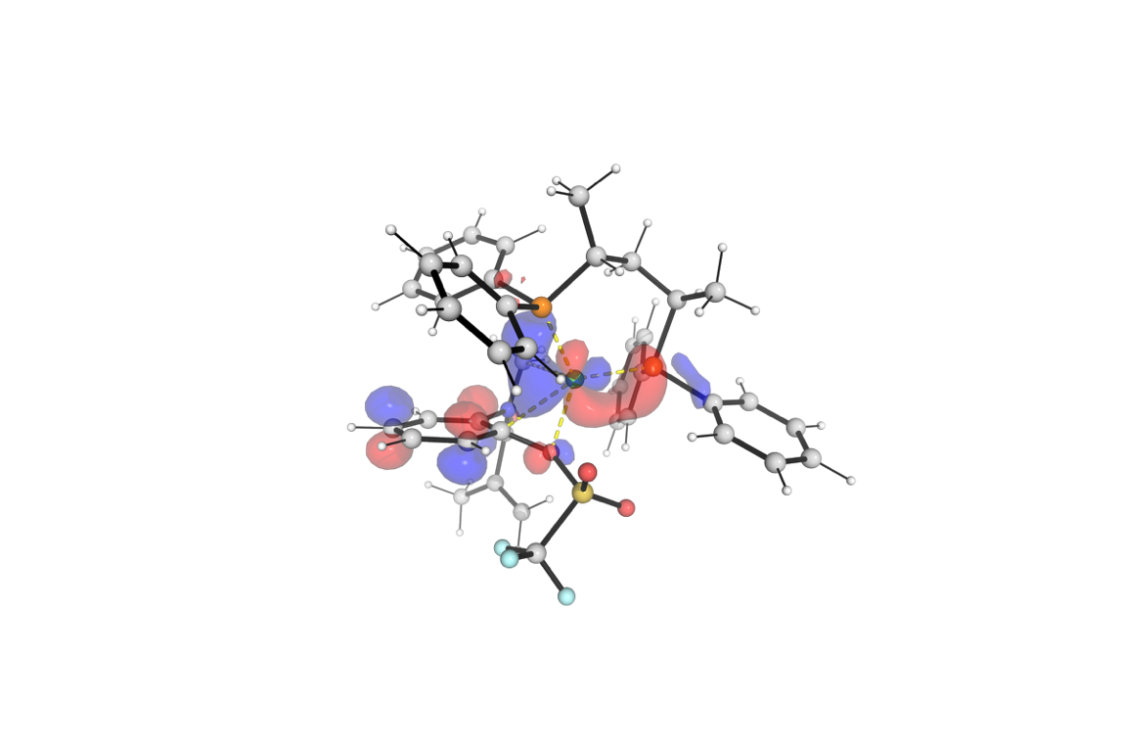** | **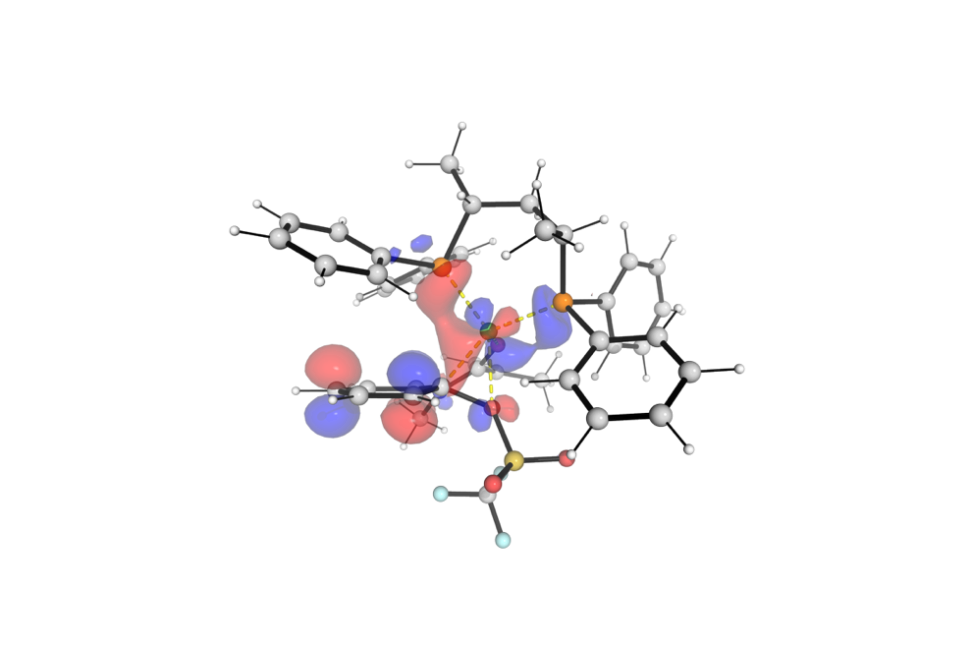** |
| **LUMO** | **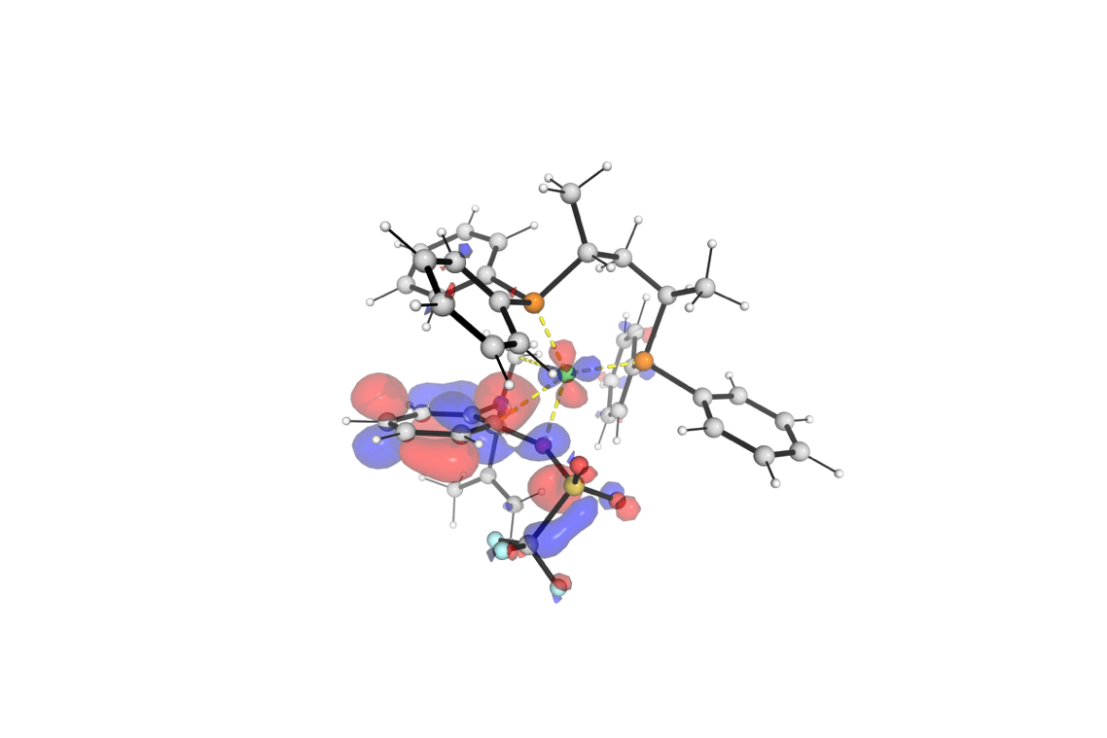** | **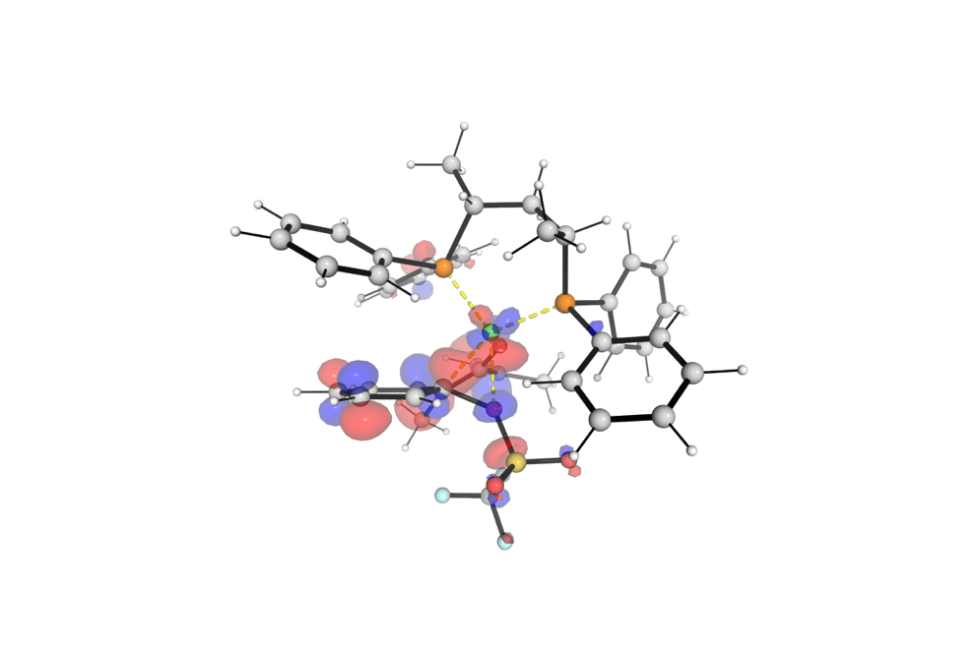** |
| **NCI** | **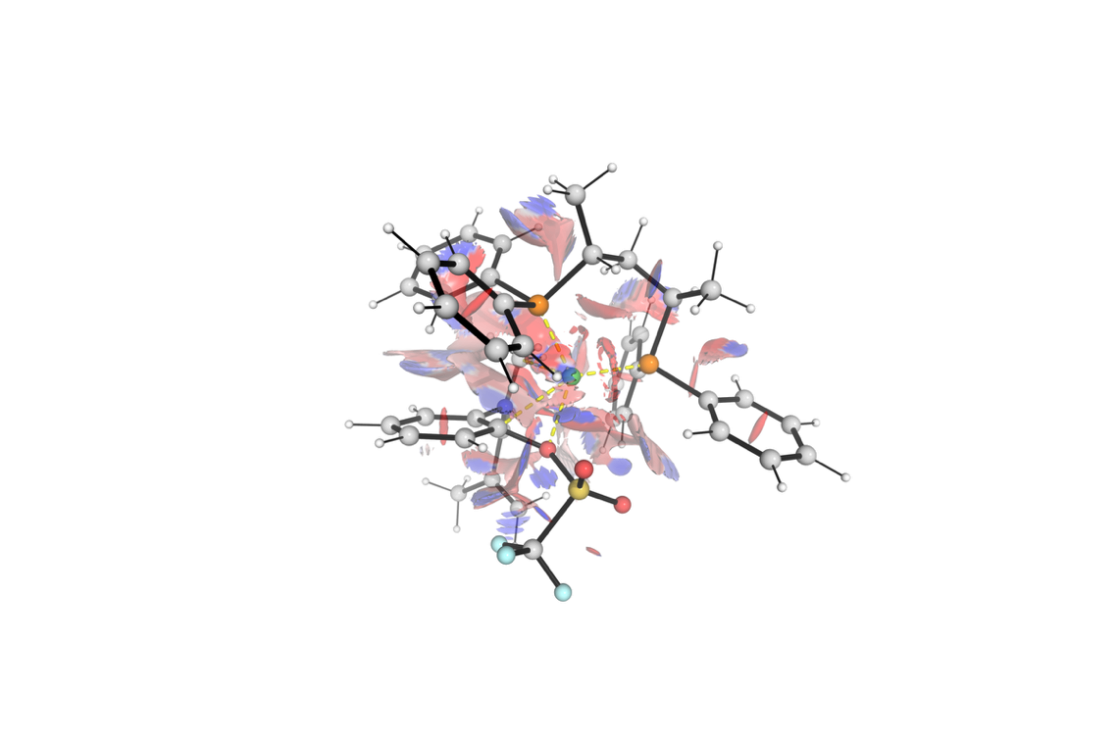** | **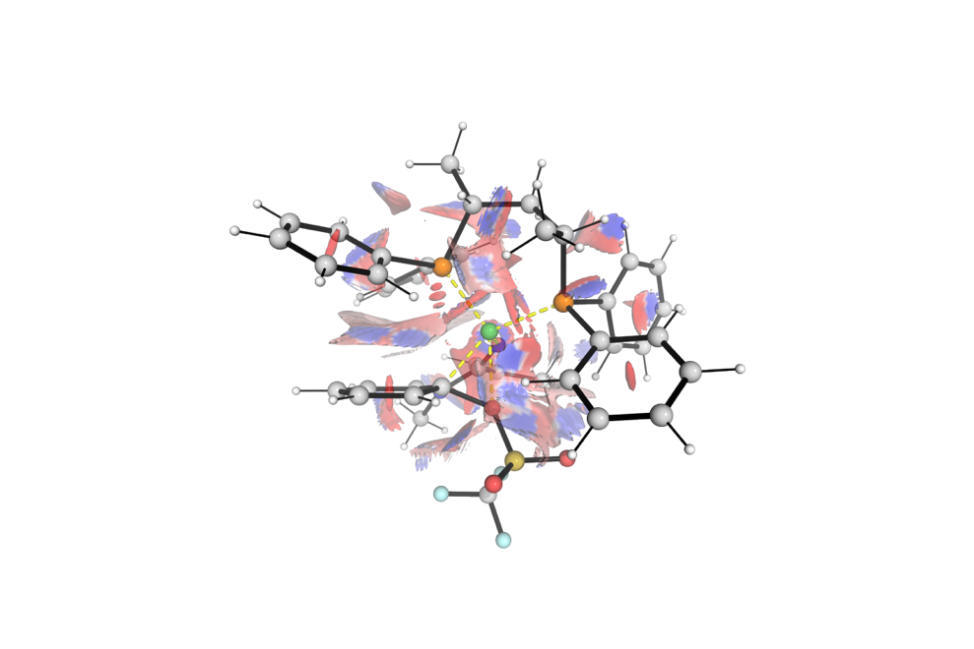** |
|  | 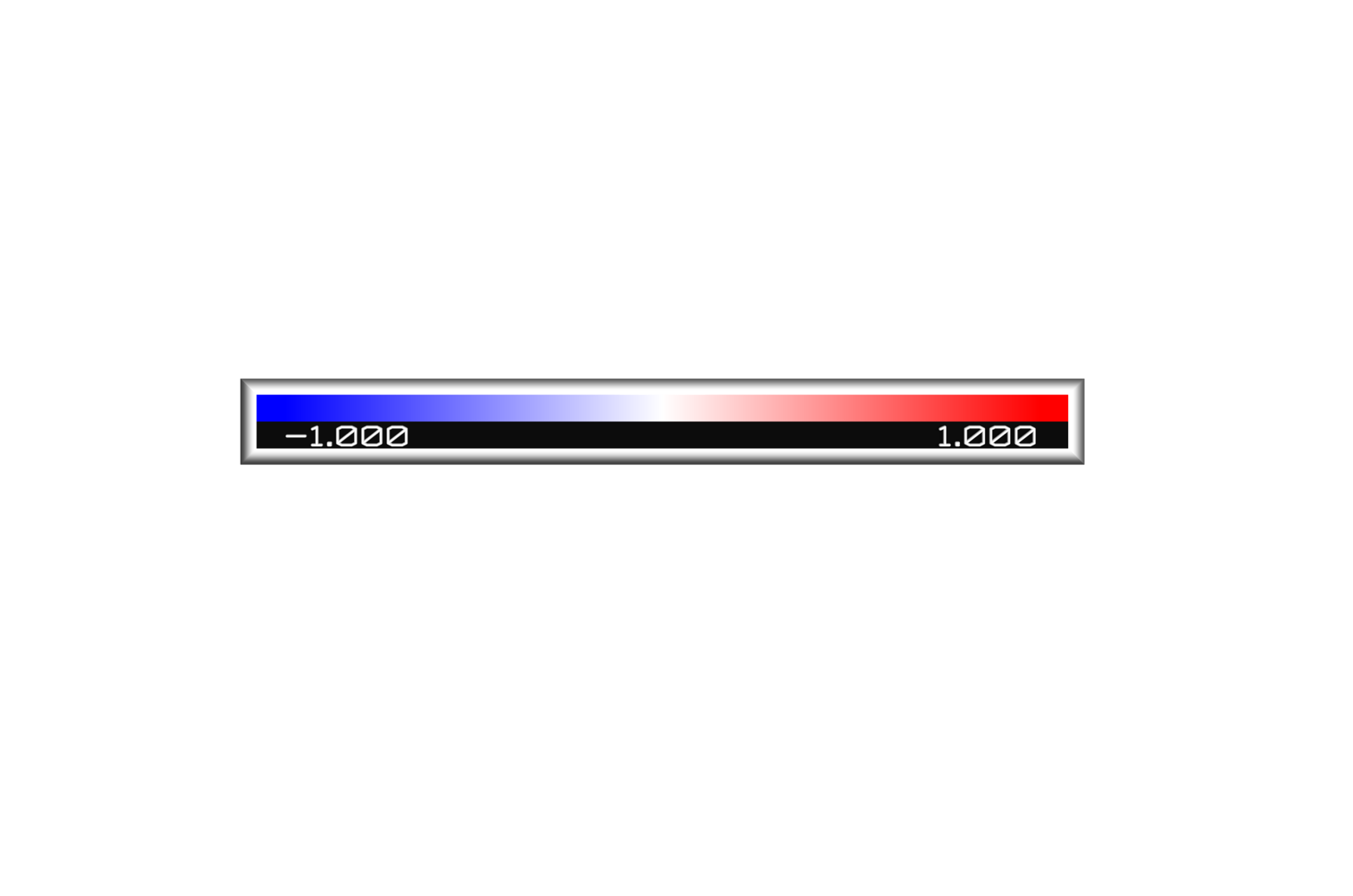 | |

**Figure S9.** DFT-optimized structures, frontier molecular orbitals (HOMO and LUMO) and non-covalent interaction (NCI) plots for the lowest barrier transition state for oxidative addition transition states (TSs) using aryl triflate as starting material.

Similar to the reaction using aryl iodide as the starting material, we propose that the steric and conjugative interactions between the aryl ring of the ligand and that of the aryl triflate play a crucial role in determining the stability of the transition states.

Compared with **TS1b-OTf-O**, the **TS1a-OTf-O** structure exhibits a more pronounced π–π conjugative interaction between the ligand phenyl ring and the aryl ring of the substrate, resulting in a more stable configuration with a 1.4 kcal·mol⁻¹ lower barrier, making it the lowest-energy transition state in the oxidative addition step.

Distortion-interaction analysis indicates that **TS1a-OTf-O** has a much lower distortion energy than **TS1b-OTf-O**, by 10.6 kcal·mol^-1^, despite **TS1b-OTf-O** has a 9.5 kcal·mol^-1^ more favorable interaction energy. Together, **TS1a-OTf-O** is favored over **TS1b-OTf-O**, by ΔΔ 𝐸^‡^ = 1.1 kcal·mol⁻¹.

**Table S2.** **Distortion-interaction analysis for oxidative addition step using aryl triflate as starting material.**

| **Transition State** | **Δ 𝐸^‡^** | ***E_dist_*** | ***E_int_*** |
| --- | --- | --- | --- |
| **TS1a-OTf** | -8.0 | 15.9 | -23.9 |
| **TS1b-OTf** | -7.5 | 16.3 | -23.8 |
| **TS1a-OTf-O** | -8.1 | 15.3 | -23.4 |
| **TS1b-OTf-O** | -7.0 | 25.9 | -32.9 |

**7.4.3 Competing transition states for migratory insertion step**

As discussed above, the oxidative addition step for the reaction using aryl triflate as the starting material can be regarded as irreversible, and interconversion between the post-oxidative insertion intermediates is prohibited by substantial steric hindrance. Therefore, only the transition states derived from **TS1a-OTf-O** and **TS1b-OTf-O**, which possess the lowest relative barriers, were considered in the following analysis. In addition, a transition state potentially leading to a six-membered-ring structure was also examined and compared with **TS1a-OTf-O**.

Due to the significant steric hindrance from the phenyl groups on the phosphine oxide, the acrylamide side chain can approach the reaction site from only one direction—opposite to the phosphine oxide group (Figure S12). The detailed Gibbs energy profiles for the migratory insertion transition states are shown in Figure S10.

**Figure S10.** Gibbs energy detail for **TS3**s. Gibbs energies are given in SMD(dimethyl sulfoxide)-MN15/def2-QZVP//MN15/def2-SVP level of theories.

All **TS3** DFT-structure are shown in Figure S11. We noticed that the distance between one of the phosphorus atoms on the ligand and the nickel center was extended to over 3.2 Å. In these structures, the phosphorus atom from the phosphine oxide, two carbon atoms from the aryl triflate, and the other phosphorus atom on the ligand formed a nearly planar coordination geometry around the nickel center, indicating that the phosphorus atom had become partially uncoordinated from nickel. Subsequent QRC analysis confirmed that these transition state structures indeed connected the two intermediates before and after the cyclization reaction.

Transition state **TS3a-OTf-O-S (6m)**, which leads to the formation of a six-membered ring, has a 23.6 kcal·mol^-1^ higher barrier than **TS3a-OTf-O-S** that forming five-membered ring, suggesting that **TS3a-OTf-O-S (6m)** can be excluded from further consideration.

| **TS3a-OTf-O-S** | **Ts3b-OTf-O-R** |
| --- | --- |
| ΔG^‡^ = -57.8 | ΔG^‡^ = -57.5 |
|  |  |
| **TS3a-OTf-O-S (6m)** |  |
| ΔG^‡^ = -34.2 |  |
|  |  |

**Figure S11.** DFT-optimized structures of competing transition state in migratory insertion step, these structures were derived from lowest barriers **TS1**s that using aryl triflate as starting material, **TS1a-OTf-O** and **TS1b-OTf-O**. Gibbs free energies are given relative to the ground state of the combined reactants and ligand. Gibbs energies are given in SMD(dimethyl sulfoxide)-MN15/def2-QZVP//MN15/def2-SVP level of theories.

Analyses of FMOs and NCI on **TS3**s were performed, and the results are shown in Figure S12.

|  | **TS3a-OTf-O-S** | **Ts3b-OTf-O-R** |
| --- | --- | --- |
| **barrier** | ΔG^‡^ = -57.8 | ΔG^‡^ = -57.5 |
| **DFT**  **Structure** |  |  |
| **HOMO** |  |  |
| **LUMO** |  |  |
| **NCI** |  |  |
|  |  | |
|  | **TS3a-OTf-O-S (6m)** |  |
| **barrier** | ΔG^‡^ = -34.2 |  |
| **DFT**  **Structure** |  |  |
| **HOMO** |  |  |
| **LUMO** |  |  |
| **NCI** |  |  |
|  |  | |

**Figure S12.** DFT-optimized structures, frontier molecular orbitals (HOMO and LUMO) and non-covalent interaction (NCI) plots for the transition state for migratory insertion transition states (TSs) derived from **TS1** using aryl triflate as starting material.

In summary, for the reaction using aryl triflate as the starting material, the chirality of the final product is determined at the oxidative addition step. When the reaction proceeds through **TS1a-OTf-O**, it subsequently follows the **TS3a-OTf-O-S** pathway to yield the *S*-configured product. Conversely, when proceeding via **TS1b-OTf-O**, the reaction continues through **TS3b-OTf-O-R**, leading to the formation of the *R*-configured product.

**7.4.4 Estimation of R/S product ratio under kinetic control**

Since the oxidative addition step is irreversible, interconversion between post-oxidative insertion intermediates is prohibited by steric hindrance, and the subsequent steps lead to the formation of a single, well-defined chirality, the ratio of enantiomeric products and the corresponding ee value can be estimated based on the barrier difference of the **TS1** structures, using the equation provided in **Section 6.3.4**.

In the calculations, only the two lowest-energy transition states, **TS1a-OTf-O** and **TS1b-OTf-O**, were considered. The former leads to the formation of the *S*-configured product, while the latter affords the *R*-configured product. The energy barrier difference between these two transition states is 1.4 kcal·mol⁻¹, giving a calculated k[*R*]/k[*S*] ≈ 0.094 at 298.15K, corresponding to an *S*-enantiomer ratio of approximately 91.4% and a theoretical ee value of about 83%, which is in good agreement with the experimental result.

**7.5 Optimized structures and absolute energies**

Geometries of all optimized structures (in .xyz format with their associated gas-phase energy in Hartrees) are included in a separate folder named *DFT_models*. All these data have been uploaded to [https://zenodo.org/records/17636365](https://zenodo.org/uploads/17636365) (DOI: 10.5281/zenodo.17636365)

Absolute values (in Hartrees) for SCF energy, zero-point vibrational energy (ZPE), enthalpy and quasi-harmonic Gibbs free energy for optimized structures are given below, as shown in Table S3 and Table S4. Single point corrections in SMD(N,N-dimethylformamide/dimethyl sulfoxide)-MN15/def2-QZVP//MN15/def2-SVP level of theory are also included.

**Table S3. Thermochemical and electronic data for selected species in the reaction using aryl iodide as the starting material, calculated at the SMD(N,N-dimethylformamide)–MN15/def2-QZVP//MN15/def2-SVP level of theory at 308.15 K.**

| **Structure** | **E/au** | **ZPE**  **/au** | **H/au** | **T.S/au** | **qh-G/au** | **SP SMD(N,N-dimethylformamide)** |
| --- | --- | --- | --- | --- | --- | --- |
| **aryl_iodide** | -852.187479 | 0.206393 | -851.964954 | 0.061050 | -852.026181 | -853.228646 |
| **bdpp_nil(I)** | -3311.362643 | 0.511211 | -3310.818532 | 0.097225 | -3310.914826 | -3314.166431 |
| **ts1b_I** | -4163.557578 | 0.718190 | -4162.790141 | 0.134536 | -4162.922950 | -4167.401706 |
| **ts1b_I_O** | -4163.571939 | 0.719053 | -4162.803997 | 0.130203 | -4162.934168 | -4167.401687 |
| **ts1a_I** | -4163.561340 | 0.717284 | -4162.794417 | 0.134314 | -4162.927689 | -4167.406869 |
| **ts1a_I_O** | -4163.568551 | 0.719104 | -4162.800510 | 0.131249 | -4162.931245 | -4167.407671 |
| **ts1c_I_O** | -4163.558812 | 0.718429 | -4162.791122 | 0.132961 | -4162.923136 | -4167.400383 |
| **ts1d_I** | -4163.558580 | 0.718311 | -4162.790948 | 0.134259 | -4162.923600 | -4167.404603 |
| **ts1d_I_O** | -4163.567573 | 0.718895 | -4162.799500 | 0.133109 | -4162.93136 | -4167.406597 |
| **ts1c_I** | -4163.558943 | 0.718381 | -4162.791106 | 0.134949 | -4162.924265 | -4167.406022 |
| **int1a_I** | -4163.573436 | 0.718931 | -4162.804616 | 0.134203 | -4162.937895 | -4167.414929 |
| **int1c_I** | -4163.573364 | 0.719560 | -4162.803887 | 0.135631 | -4162.937908 | -4167.424876 |
| **int2a_I** | -4163.641893 | 0.720599 | -4162.871912 | 0.131503 | -4163.003494 | -4167.479568 |
| **int3'a_I** | -4745.188117 | 0.910169 | -4744.217676 | 0.152130 | -4744.369989 | -4750.191475 |
| **int4'a_I_R** | -4745.172355 | 0.912547 | -4744.200538 | 0.149642 | -4744.349924 | -4750.171514 |
| **ts3a_I_R** | -4745.146641 | 0.911168 | -4744.176436 | 0.149844 | -4744.326010 | -4750.145839 |
| **int3a_I_R** | -4163.629969 | 0.722070 | -4162.858916 | 0.129935 | -4162.988992 | -4167.468792 |
| **ts2a_I_R** | -4163.623641 | 0.722278 | -4162.853650 | 0.127475 | -4162.980889 | -4167.467042 |
| **ts2am6_I_R** | -4163.594710 | 0.723167 | -4162.824126 | 0.126078 | -4162.950119 | -4167.419264 |
| **ts2a_I_S** | -4163.612230 | 0.721371 | -4162.842559 | 0.130167 | -4162.971835 | -4167.445425 |
| **ts2c_I_R** | -4163.611839 | 0.721159 | -4162.842254 | 0.129658 | -4162.971603 | -4167.443843 |
| **ts2c_I_S** | -4163.635929 | 0.722003 | -4162.865830 | 0.128236 | -4162.994077 | -4167.468400 |
| **int4a_I_R** | -4163.668194 | 0.724129 | -4162.89584 | 0.129079 | -4163.024493 | -4167.503495 |
| **int5a_I_R** | -4745.181122 | 0.911030 | -4744.210031 | 0.153713 | -4744.363016 | -4750.182414 |
| **int6a_I_R** | -4745.213808 | 0.912742 | -4744.241436 | 0.152884 | -4744.393231 | -4750.217221 |
| **ts4a_I_R** | -4745.186024 | 0.910982 | -4744.215744 | 0.151172 | -4744.366489 | -4750.190366 |
| **int7a_I_R** | -4745.228545 | 0.911180 | -4744.257643 | 0.153370 | -4744.409895 | -4750.231961 |
| **3a_R** | -1433.831302 | 0.398700 | -1433.406273 | 0.082558 | -1433.488354 | -1436.049510 |
| **dppo_anion(I)** | -878.562159 | 0.185303 | -878.36336 | 0.054724 | -878.417721 | -879.846608 |
| **I_anion** | -297.047556 | 0.000000 | -297.045116 | 0.019933 | -297.06505 | -297.172673 |

**Table S4. Thermochemical and electronic data for selected species in the reaction using aryl triflate as the starting material, calculated at the SMD(dimethyl sulfoxide)–MN15/def2-QZVP//MN15/def2-SVP level of theory at 298.15 K.**

| **Structure** | **E/au** | **ZPE**  **/au** | **H/au** | **T.S/au** | **qh-G/au** | **SP SMD(N,N-dimethylformamide)** |
| --- | --- | --- | --- | --- | --- | --- |
| **aryl_triflate** | -1515.425138 | 0.237752 | -1515.166134 | 0.070898 | -1515.237198 | -1517.710105 |
| **bdpp_nil(OTf)** | -3311.362643 | 0.511211 | -3310.820496 | 0.092139 | -3310.911900 | -3314.161302 |
| **ts1b_OTf** | -4826.785362 | 0.748841 | -4825.984052 | 0.137746 | -4826.121367 | -4831.856917 |
| **ts1b_OTf_O** | -4826.79122 | 0.748683 | -4825.990223 | 0.138272 | -4826.127263 | -4831.859297 |
| **ts1a_OTf** | -4826.788334 | 0.749076 | -4825.986834 | 0.137343 | -4826.123723 | -4831.858841 |
| **ts1a_OTf_O** | -4826.800186 | 0.749602 | -4825.998411 | 0.13674 | -4826.134700 | -4831.863169 |
| **ts1c_OTf_O** | -4826.789181 | 0.747911 | -4825.988535 | 0.138402 | -4826.126446 | -4831.855438 |
| **ts1d_OTf_O** | -4826.794716 | 0.748662 | -4825.99383 | 0.135318 | -4826.129523 | -4831.85229 |
| **ts1d_OTf** | -4826.789777 | 0.749304 | -4825.988226 | 0.137175 | -4826.124869 | -4831.85718 |
| **ts1c_OTf** | -4826.78469 | 0.748743 | -4825.983426 | 0.137907 | -4826.120963 | -4831.855984 |
| **int1a_OTf_O** | -4826.834738 | 0.751681 | -4826.03103 | 0.137277 | -4826.167242 | -4831.899764 |
| **int1b_OTf_O** | -4826.838900 | 0.751832 | -4826.035229 | 0.135342 | -4826.170026 | -4831.900376 |
| **int2a_OTf_O** | -4826.892574 | 0.752602 | -4826.087744 | 0.136439 | -4826.223851 | -4831.963974 |
| **int3a_OTf_O_S** | -4826.855634 | 0.752774 | -4826.050977 | 0.134511 | -4826.185772 | -4831.928162 |
| **ts2a_OTf_O_S** | -4826.855540 | 0.752362 | -4826.052018 | 0.132887 | -4826.185161 | -4831.927979 |
| **int3'a_OTf_O** | -4745.209995 | 0.911527 | -4744.242309 | 0.142574 | -4744.385139 | -4750.192483 |
| **int4'a_OTf_O_S** | -4745.166183 | 0.910522 | -4744.199029 | 0.143566 | -4744.342965 | -4750.162721 |
| **ts3b_OTf_O_R** | -4745.164680 | 0.909768 | -4744.199150 | 0.141904 | -4744.341301 | -4750.156679 |
| **ts3a_OTf_O_S** | -4745.162552 | 0.909633 | -4744.196946 | 0.142606 | -4744.339673 | -4750.156656 |
| **ts3am6_OTf_O_S** | -4745.126159 | 0.909528 | -4744.160681 | 0.142780 | -4744.303399 | -4750.118904 |
| **int5a_OTf_O_S** | -4745.197098 | 0.909953 | -4744.230314 | 0.146465 | -4744.37603 | -4750.186898 |
| **int6a_OTf_O_S** | -4745.239010 | 0.912267 | -4744.270785 | 0.143907 | -4744.414007 | -4750.225691 |
| **ts4a_OTf_O_S** | -4745.19492 | 0.911293 | -4744.228134 | 0.141097 | -4744.369684 | -4750.191506 |
| **int7a_OTf_O_S** | -4745.219244 | 0.911071 | -4744.251894 | 0.145003 | -4744.396344 | -4750.221979 |
| **3a_S** | -1433.834335 | 0.398563 | -1433.411054 | 0.078024 | -1433.488836 | -1436.047925 |
| **dppo_anion(OTf)** | -878.562159 | 0.185303 | -878.364134 | 0.052187 | -878.416019 | -879.844633 |
| **OTf_anion** | -960.287401 | 0.02857 | -960.2508 | 0.040595 | -960.291384 | -961.669917 |

**7.6 References**

**Full reference Gaussian 16:**

Gaussian 16, Revision B.01, Frisch, M. J.; Trucks, G. W.; Schlegel, H. B.; Scuseria, G. E.; Robb, M. A.; Cheeseman, J. R.; Scalmani, G.; Barone, V.; Mennucci, B.; Petersson, G. A.; Nakatsuji, H.; Caricato, M.; Li, X.; Hratchian, H. P.; Izmaylov, A. F.; Bloino, J.; Zheng, G.; Sonnenberg, J. L.; Hada, M.; Ehara, M.; Toyota, K.; Fukuda, R.; Hasegawa, J.; Ishida, M.; Nakajima, T.; Honda, Y.; Kitao, O.; Nakai, H.; Vreven, T.; Montgomery Jr., J. A.; Peralta, J. E.; Ogliaro, F.; Bearpark, M.; Heyd, J. J.; Brothers, E.; Kudin, K. N.; Staroverov, V. N.; Kobayashi, R.; Normand, J.; Raghavachari, K.; Rendell, A.; Burant, J. C.; Iyengar, S. S.; Tomasi, J.; Cossi, M.; Rega, N.; Millam, J. M.; Klene, M.; Knox, J. E.; Cross, J. B.; Bakken, V.; Adamo, C.; Jaramillo, J.; Gomperts, R.; Stratmann, R. E.; Yazyev, O.; Austin, A. J.; Cammi, R.; Pomelli, C.; Ochterski, J. W.; Martin, R. L.; Morokuma, K.; Zakrzewski, V. G.; Voth, G. A.; Salvador, P.; Dannenberg, J. J.; Dapprich, S.; Daniels, A. D.; Farkas, Ö.; Foresman, J. B.; Ortiz, J. V; Cioslowski, J.; Fox, D. J. Gaussian, Inc., Wallingford CT, 2016.

1. Grimme, S. Exploration of Chemical Compound, Conformer, and Reaction Space with Meta-Dynamics Simulations Based on Tight-Binding Quantum Chemical Calculations. *J Chem Theory Comput* **15**, 2847–2862 (2019).

2. Pracht, P., Bohle, F. & Grimme, S. Automated exploration of the low-energy chemical space with fast quantum chemical methods. *Physical Chemistry Chemical Physics* **22**, 7169–7192 (2020).

3. Bannwarth, C., Ehlert, S. & Grimme, S. GFN2-xTB - An Accurate and Broadly Parametrized Self-Consistent Tight-Binding Quantum Chemical Method with Multipole Electrostatics and Density-Dependent Dispersion Contributions. *J Chem Theory Comput* **15**, 1652–1671 (2019).

4. Grimme, S., Bannwarth, C. & Shushkov, P. A Robust and Accurate Tight-Binding Quantum Chemical Method for Structures, Vibrational Frequencies, and Noncovalent Interactions of Large Molecular Systems Parametrized for All spd-Block Elements (Z = 1-86). *J Chem Theory Comput* **13**, 1989–2009 (2017).

5. Bannwarth, C. *et al.* Extended tight-binding quantum chemistry methods. *Wiley Interdisciplinary Reviews: Computational Molecular Science* vol. 11 Preprint at https://doi.org/10.1002/wcms.1493 (2021).

6. Frisch, M. J. .; Trucks, G. W. .; Schlegel, H. B. .; Scuseria, G. E. .; Robb, M. A. .; Cheeseman, J. R. .; Scalmani, G. .; Barone, V. .; Petersson, G. A. .; Nakatsuji, H. .; et al. Gaussian 16, Revision B.01. 2016.

7. Yu Xiao and Li Shaohong L and Truhlar Donald G, H. S. and H. MN15: A Kohn-Sham global-hybrid exchange-correlation density functional with broad accuracy for multi-reference and single-reference systems and noncovalent interactions. *Chem Sci* **7**, 5031—5051 (2016).

8. Weigend, F. & Ahlrichs, R. Balanced basis sets of split valence, triple zeta valence and quadruple zeta valence quality for H to Rn: Design and assessment of accuracy. *Physical Chemistry Chemical Physics* 3297–3305 (2005) doi:10.1039/b508541a.

9. Weigend, F. Accurate Coulomb-fitting basis sets for H to Rn. *Physical Chemistry Chemical Physics* **8**, 1057–1065 (2006).

10. Goodman, J. M. & Silva, M. A. QRC: A rapid method for connecting transition structures to reactants in the computational analysis of organic reactivity. *Tetrahedron Lett* **44**, 8233–8236 (2003).

11. Marenich, A. V., Cramer, C. J. & Truhlar, D. G. Universal solvation model based on solute electron density and on a continuum model of the solvent defined by the bulk dielectric constant and atomic surface tensions. *Journal of Physical Chemistry B* **113**, 6378–6396 (2009).

12. Grimme, S. Supramolecular binding thermodynamics by dispersion-corrected density functional theory. *Chemistry - A European Journal* **18**, 9955–9964 (2012).

13. Li, Y. P., Gomes, J., Sharada, S. M., Bell, A. T. & Head-Gordon, M. Improved force-field parameters for QM/MM simulations of the energies of adsorption for molecules in zeolites and a free rotor correction to the rigid rotor harmonic oscillator model for adsorption enthalpies. *Journal of Physical Chemistry C* **119**, 1840–1850 (2015).

14. Zhang, X. *et al.* *CHEMSMART: Chemistry Simulation and Modeling Automation Toolkit for High-Efficiency Computational Chemistry Workflows*. https://github.com/xinglong-zhang/chemsmart.

15. Contreras-García, J. *et al.* NCIPLOT: A program for plotting noncovalent interaction regions. *J Chem Theory Comput* **7**, 625–632 (2011).

16. Sosa, C. *et al.* *A Local Density Functional Study of the Structure and Vibrational Frequencies of Molecular Transition-Metal Compounds1*. *J. Phys. Chem* vol. 96 https://pubs.acs.org/sharingguidelines (1992).

17. Godbout, N., Salahub’, D. R., Andzelm’ And, J. & Wimmer, E. *Optimization of Gaussian-Type Basis Sets for Local Spin Density Functional Calculations. Part I. Boron through Neon, Optimization Technique and Validation*.

18. Schrödinger, L. The PyMOL Molecular Graphics Development Component, Version 1.8; 2015.

19. Ess, D. H. & Houk, K. N. Distortion/interaction energy control of 1,3-dipolar cycloaddition reactivity. *J Am Chem Soc* **129**, 10646–10647 (2007).

20. Bickelhaupt, F. M. & Houk, K. N. Das Distortion/Interaction‐Activation‐Strain‐Modell zur Analyse von Reaktionsgeschwindigkeiten. *Angewandte Chemie* **129**, 10204–10221 (2017).

21. Wang, G., Shen, C., Ren, X. & Dong, K. Ni-Catalyzed enantioselective reductive arylcyanation/cyclization of: N -(2-iodo-aryl) acrylamide. *Chemical Communications* **58**, 1135–1138 (2022).

## 8. X-Ray crystallographic analysis.

**X-ray crystal structure of (*S*)-3-((bis(3,5-dimethylphenyl)phosphoryl)methyl)-1,3,7-trimethylindolin-2-one (3u)**

Crystal data and structure refinement for cxy6142_0m

| Identification code | cxy6142_0m |
| --- | --- |
| Empirical formula | C_28_ H_34_ N O_3_ P |
| Formula weight | 463.53 |
| Temperature/K | 100 K |
| Crystal system | orthorhombic |
| Space group | P2_1_2_1_2_1_ |
| a/Å | 8.3132(8) |
| b/Å | 14.0329(13) |
| c/Å | 21.183(2) |
| α/° | 90 |
| β/° | 90 |
| γ/° | 90 |
| Volume/Å^3^ | 2471.1(4) |

**X-ray crystal structure of (*R*)-5-chloro-3-((diphenylphosphoryl)methyl)-1,3-dimethylindolin-2-one (3j)**

Crystal data and structure refinement for cxy6113_0m.

| Identification code | cxy6113_0m |
| --- | --- |
| Empirical formula | C_24_H_22_Cl_4_NO_2_P |
| Formula weight | 529.19 |
| Temperature/K | 100 K |
| Crystal system | orthorhombic |
| Space group | P2_1_2_1_2_1_ |
| a/Å | 6.2921(5) |
| b/Å | 15.7869(12) |
| c/Å | 23.8781(18) |
| α/° | 90 |
| β/° | 90 |
| γ/° 90 | 90 |
| Volume/Å^3^ | 2371.9(3) |

## 9. Characterization of products

**3-((diphenylphosphoryl)methyl)-1,3-dimethylindolin-2-one (3a)**

**^1^H NMR** (600 MHz, Chloroform-*d*) δ 7.59 – 7.52 (m, 2H), 7.50 – 7.44 (m, 2H), 7.43 – 7.37 (m, 2H), 7.37 – 7.28 (m, 4H), 7.15 – 7.13 (m, 2H), 6.80 – 6.74 (m, 1H), 6.69 – 6.25 (m,, 1H), 3.08 (dd, *J* = 15.3, 10.2 Hz, 1H), 3.00 (s, 3H), 2.85 (dd, *J* = 15.3, 10.8 Hz, 1H), 1.42 (d, *J* = 1.8 Hz, 3H).

**^13^C NMR** (150 MHz, Chloroform-*d*) δ 179.48 (d, *J* = 3.9 Hz), 142.97, 133.62 (d, *J* = 99.8 Hz), 132.87 (d, *J* = 98.6 Hz), 131.44 (d, *J* = 2.7 Hz), 131.42, 131.30 (d, *J* = 2.7 Hz), 130.75 (d, *J* = 9.4 Hz), 130.51 (d, *J* = 9.2 Hz), 128.43 (d, *J* = 11.8 Hz), 128.21 (d, *J* = 11.8 Hz), 127.97, 124.82, 122.20, 107.88, 45.47 (d, *J* = 3.8 Hz), 37.50 (d, *J* = 71.5 Hz), 26.92 (d, *J* = 12.1 Hz), 26.35.

**^31^P NMR** (240 MHz, Chloroform-*d*) δ 26.05.

**HRMS (ESI) m/z**: calcd for C_16_H_20_NOP [M+H]^+^ 274.1355, found 274.1351.

(*R*)-**3a** was synthesized from the **1a**; 90% yield, 84% ee; [α]^20^_D_= 28.5 (c = 1.4, CHCl_3_); Colorless oil; HPLC analysis: Chiralcel ID column (hexane / 2-propanol 50:50, 1.0 mL/min, 254 nm); t_r_ (minor) = 14.0 min, t_r_ (major) = 19.1 min. **HRMS (ESI) m/z**: calcd for C_23_H_22_NO_2_P [M+H]^+^ 376.1466, found 376.1461.

(*S*)-**3a** was synthesized from the **2a**; 94% yield, 83% ee; [α]^20^_D_= -27.4 (c = 1.3, CHCl_3_); Colorless oil; HPLC analysis: Chiralcel ID column (hexane / 2-propanol 50:50, 1.0 mL/min, 254 nm); t_r_ (major) = 13.7 min, t_r_ (minor) = 19.3 min. **HRMS (ESI) m/z**: calcd for C_23_H_22_NO_2_P [M+H]^+^ 376.1466, found 376.1458.

**3-((diphenylphosphoryl)methyl)-1,3,6-trimethylindolin-2-one (3b)**

**^1^H NMR** (600 MHz, Chloroform-*d*) δ 7.61 – 7.53 (m, 2H), 7.51 – 7.45 (m, 2H), 7.45 – 7.37 (m, 2H), 7.37 – 7.28 (m, 4H), 7.02 (d, *J* = 7.5 Hz, 1H), 6.58 (d, *J* = 7.5 Hz, 1H), 6.49 (s, 1H), 3.06 (dd, *J* = 15.2, 10.2 Hz, 1H), 2.98 (s, 3H), 2.84 (dd, *J* = 15.2, 10.8 Hz, 1H), 2.33 (s, 3H), 1.40 (d, *J* = 1.8 Hz, 3H).

**^13^C NMR** (150 MHz, Chloroform-*d*) δ 179.76 (d, *J* = 4.2 Hz), 143.01, 138.05, 133.68 (d, *J* = 99.5 Hz), 132.95 (d, *J* = 98.7 Hz), 131.41 (d, *J* = 2.8 Hz), 131.25 (d, *J* = 2.9 Hz), 130.76 (d, *J* = 9.5 Hz), 130.53 (d, *J* = 9.0 Hz), 128.53 (d, *J* = 2.7 Hz), 128.35 (d, *J* = 11.9 Hz), 128.18 (d, *J* = 11.9 Hz), 124.56, 122.74, 108.78, 45.26 (d, *J* = 3.7 Hz), 37.67 (d, *J* = 71.5 Hz), 26.85 (d, *J* = 12.0 Hz), 26.31, 21.78.

**^31^P NMR** (240 MHz, Chloroform-*d*) δ 26.28.

(*R*)-**3b** was synthesized from the **1b**; 87% yield, 81% ee; [α]^20^_D_= 21.4 (c = 1.2, CHCl_3_); Colorless oil; HPLC analysis: Chiralcel ID column (hexane / 2-propanol 50:50, 1.0 mL/min, 254 nm); t_r_ (minor) = 12.5 min, t_r_ (major) = 19.9 min. **HRMS (ESI) m/z**: calcd for C_24_H_24_NO_2_P [M+H]^+^ 390.1623, found 390.1617.

(*S*)-**3b** was synthesized from the **2b**; 97% yield, 84% ee; [α]^20^_D_= -20.5 (c = 1.5, CHCl_3_); Colorless oil; HPLC analysis: Chiralcel ID column (hexane / 2-propanol 50:50, 1.0 mL/min, 254 nm); t_r_ (major) = 12.2 min, t_r_ (minor) = 20.1 min. **HRMS (ESI) m/z**: calcd for C_24_H_24_NO_2_P [M+H]^+^ 390.1623, found 390.1615.

**3-((diphenylphosphoryl)methyl)-6-methoxy-1,3-dimethylindolin-2-one (3c)**

**^1^H NMR** (600 MHz, Chloroform-*d*) δ 7.63 – 7.56 (m, 2H), 7.53 – 7.47 (m, 2H), 7.47 – 7.40 (m, 2H), 7.40 – 7.31 (m, 4H), 7.06 (d, *J* = 8.2 Hz, 1H), 6.34 – 6.22 (m, 2H), 3.81 (s, 3H), 3.08 (dd, *J* = 15.3, 10.1 Hz, 1H), 2.99 (s, 3H), 2.85 (dd, *J* = 15.3, 10.9 Hz, 1H), 1.41 (d, *J* = 1.8 Hz, 3H). ^1^H NMR (400 MHz, Chloroform-*d*) δ 7.62 – 7.52 (m, 2H), 7.51 – 7.44 (m, 2H), 7.44 – 7.28 (m, 6H), 7.02 (d, *J* = 8.0 Hz, 1H), 6.34 – 6.21 (m, 2H), 3.78 (s, 3H), 3.05 (dd, *J* = 15.2, 10.1 Hz, 1H), 2.97 (s, 3H), 2.82 (dd, *J* = 15.2, 10.9 Hz, 1H), 1.38 (d, *J* = 1.9 Hz, 3H).

**^13^C NMR** (150 MHz, Chloroform-*d*) δ 180.07 (d, *J* = 4.2 Hz), 160.07, 144.22, 133.80 (d, *J* = 99.3 Hz), 132.99 (d, *J* = 98.4 Hz), 131.41 (d, *J* = 2.7 Hz), 131.27 (d, *J* = 2.7 Hz), 130.73 (d, *J* = 9.5 Hz), 130.51 (d, *J* = 9.2 Hz), 128.39 (d, *J* = 11.6 Hz), 128.20 (d, *J* = 11.7 Hz), 125.53, 123.44 (d, *J* = 2.9 Hz), 105.91, 95.96, 55.51, 45.00 (d, *J* = 3.7 Hz), 37.77 (d, *J* = 71.6 Hz), 26.99 (d, *J* = 11.8 Hz), 26.37.

**^31^P NMR** (240 MHz, Chloroform-*d*) δ 26.09.

(*S*)-**3c** was synthesized from the **2c**; 97% yield, 84% ee; [α]^20^_D_= -29.8 (c = 1.6, CHCl_3_); Colorless oil; HPLC analysis: Chiralcel ID column (hexane / 2-propanol 50:50, 1.0 mL/min, 254 nm); t_r_ (major) = 17.9 min, t_r_ (minor) = 30.0 min. **HRMS (ESI) m/z**: calcd for C_24_H_24_NO_3_P [M+H]^+^ 406.1572, found 406.1565.

**(*S*)-3-((diphenylphosphoryl)methyl)-1,3-dimethyl-6-(tert-pentyl)indolin-2-one (3d)**

**^1^H NMR** (600 MHz, Chloroform-*d*) δ 7.61 – 7.55 (m, 2H), 7.49 – 7.42 (m, 3H), 7.42 – 7.38 (m, 1H), 7.38 – 7.34 (m, 2H), 7.32 – 7.29 (m, 2H), 7.05 (d, *J* = 7.8 Hz, 1H), 6.78 – 6.73 (m, 1H), 6.61 (d, *J* = 1.7 Hz, 1H), 3.12 (dd, *J* = 15.3, 10.0 Hz, 1H), 3.02 (s, 3H), 2.86 (dd, *J* = 15.3, 11.0 Hz, 1H), 1.69 – 1.59 (m, 2H), 1.43 (d, *J* = 1.8 Hz, 3H), 1.28 (d, *J* = 6.5 Hz, 6H), 0.75 (t, *J* = 7.4 Hz, 3H).

**^13^C NMR** (150 MHz, Chloroform-*d*) δ 179.96 (d, *J* = 3.9 Hz), 149.92, 142.87, 133.84 (d, *J* = 99.3 Hz), 133.00 (d, *J* = 98.4 Hz), 131.32 (d, *J* = 2.7 Hz), 131.23 (d, *J* = 2.7 Hz), 130.78 (d, *J* = 9.3 Hz), 130.55 (d, *J* = 9.1 Hz), 128.38 (d, *J* = 11.9 Hz), 128.27 (d, *J* = 2.9 Hz), 128.13 (d, *J* = 11.6 Hz), 124.37, 119.69, 105.73, 45.22 (d, *J* = 3.7 Hz), 38.16, 37.56 (d, *J* = 71.5 Hz), 36.88, 28.63 (d, *J* = 2.3 Hz), 27.09 (d, *J* = 12.0 Hz), 26.33, 9.28.

**^31^P NMR** (240 MHz, Chloroform-*d*) δ 26.04.

(*S*)-**3d** was synthesized from the **2d**; 99% yield, 87% ee; [α]^20^_D_= -21.0 (c = 1.8, CHCl_3_); Colorless oil; HPLC analysis: Chiralcel ID column (hexane / 2-propanol 50:50, 1.0 mL/min, 254 nm); t_r_ (major) = 8.9 min, t_r_ (minor) = 13.4 min. **HRMS (ESI) m/z**: calcd for C_28_H_32_NO_2_P [M+H]^+^ 446.2249, found 446.2241.

**(*S*)-6-(tert-butyl)-3-((diphenylphosphoryl)methyl)-1,3-dimethylindolin-2-one (3e)**

**^1^H NMR** (600 MHz, Chloroform-*d*) δ 7.55 – 7.49 (m, 2H), 7.48 – 7.42 (m, 2H), 7.42 – 7.35 (m, 2H), 7.35 – 7.27 (m, 4H), 6.99 (d, *J* = 8.2 Hz, 1H), 6.76 (d, *J* = 7.1 Hz, 1H), 6.65 (d, *J* = 2.2 Hz, 1H), 3.13 – 3.06 (m, 1H), 3.03 (s, 3H), 2.83 (dd, *J* = 15.6, 10.2 Hz, 1H), 1.41 (s, 3H), 1.31 (s, 9H).

**^13^C NMR** (150 MHz, Chloroform-*d*) δ 179.95 (d, *J* = 3.9 Hz), 151.50, 142.91, 133.72 (d, *J* = 93.4 Hz), 133.06 (d, *J* = 93.0 Hz), 131.33 (d, *J* = 2.8 Hz), 131.18 (d, *J* = 2.8 Hz), 130.71 (d, *J* = 9.4 Hz), 130.54 (d, *J* = 9.2 Hz), 128.35 (d, *J* = 11.9 Hz), 128.31, 128.16 (d, *J* = 11.7 Hz), 124.33, 118.91, 105.21, 45.17 (d, *J* = 3.5 Hz), 37.69 (d, *J* = 71.9 Hz), 34.92, 31.50, 26.90 (d, *J* = 12.2 Hz), 26.36.

**^31^P NMR** (243 MHz, Chloroform-*d*) δ 26.15.

(*S*)-**3e** was synthesized from the **2e**; 95% yield, 88% ee; [α]^20^_D_= -17.4 (c = 1.5, CHCl_3_); Colorless oil; HPLC analysis: Chiralcel ID column (hexane / 2-propanol 50:50, 1.0 mL/min, 254 nm); t_r_ (major) = 9.4 min, t_r_ (minor) = 15.2 min. **HRMS (ESI) m/z**: calcd for C_27_H_30_NO_2_P [M+H]^+^ 432.2092, found 432.2086.

**(*S*)-3-((diphenylphosphoryl)methyl)-1,3-dimethyl-6-phenylindolin-2-one (3f)**

**^1^H NMR** (400 MHz, Chloroform-*d*) δ 7.58 – 7.37 (m, 11H), 7.36 – 7.29 (m, 4H), 7.13 (d, *J* = 7.7 Hz, 1H), 6.94 (dd, *J* = 7.7, 1.6 Hz, 1H), 6.86 (d, *J* = 1.6 Hz, 1H), 3.15 (dd, *J* = 15.2, 10.7 Hz, 1H), 3.10 (s, 3H), 2.90 (dd, *J* = 15.2, 10.3 Hz, 1H), 1.47 (d, *J* = 1.8 Hz, 3H).

**^13^C NMR** (150 MHz, Chloroform-*d*) δ 179.70 (d, *J* = 3.6 Hz), 143.62, 141.51, 141.28, 133.51 (d, *J* = 81.9 Hz), 132.85 (d, *J* = 81.7 Hz), 131.51 (d, *J* = 2.6 Hz), 131.29 (d, *J* = 2.6 Hz), 130.73 (d, *J* = 9.3 Hz), 130.53 (d, *J* = 9.2 Hz), 130.37 (d, *J* = 2.7 Hz), 128.82, 128.41 (d, *J* = 11.8 Hz), 128.26 (d, *J* = 11.7 Hz), 127.49, 127.18, 124.93, 121.17, 106.82, 45.33 (d, *J* = 3.7 Hz), 37.84 (d, *J* = 71.4 Hz), 26.86 (d, *J* = 12.0 Hz), 26.49.

**^31^P NMR** (240 MHz, Chloroform-*d*) δ 26.37.

(*S*)-**3f** was synthesized from the **2f**; 92% yield, 73% ee; [α]^20^_D_= -1.4 (c = 1.8, CHCl_3_); Colorless oil; HPLC analysis: Chiralcel IA column (hexane / 2-propanol 70:30, 1.0 mL/min, 254 nm); t_r_ (major) = 8.3 min, t_r_ (minor) = 12.7 min. **HRMS (ESI) m/z**: calcd for C_29_H_26_NO_2_P [M+H]^+^ 452.1779, found 452.1774.

**3-((diphenylphosphoryl)methyl)-6-fluoro-1,3-dimethylindolin-2-one (3g)**

**^1^H NMR** (400 MHz, Chloroform-*d*) δ 7.58 – 7.45 (m, 4H), 7.45 – 7.38 (m, 2H), 7.38 – 7.27 (m, 4H), 7.05 – 6.92 (m, 1H), 6.51 – 6.30 (mm, 2H), 3.08 (dd, *J* = 15.2, 10.4 Hz, 1H), 2.99 (s, 3H), 2.83 (dd, *J* = 15.2, 10.3 Hz, 1H), 1.39 (d, *J* = 1.9 Hz, 3H).

**^13^C NMR** (151 MHz, Chloroform-*d*) δ 179.83 (d, *J* = 3.8 Hz), 163.79, 162.17, 144.55 (d, *J* = 11.6 Hz), 133.46 (d, *J* = 89.8 Hz), 132.80 (d, *J* = 89.4 Hz), 131.50 (dd, *J* = 26.3, 2.7 Hz), 130.67 (d, *J* = 9.5 Hz), 130.44 (d, *J* = 9.2 Hz), 128.46 (d, *J* = 11.8 Hz), 128.31 (d, *J* = 11.8 Hz), 126.61 (t, *J* = 2.9 Hz), 125.80 (d, *J* = 9.8 Hz), 108.07 (d, *J* = 22.3 Hz), 96.62 (d, *J* = 27.4 Hz), 45.07 (d, *J* = 3.8 Hz), 37.69 (d, *J* = 71.5 Hz), 26.90 (d, *J* = 12.0 Hz), 26.52.

**^31^P NMR** (162 MHz, Chloroform-*d*) δ 26.20.

**^19^F NMR** (565 MHz, Chloroform-*d*) δ -112.60.

(*R*)-**3g** was synthesized from the **1g**; 99% yield, 79% ee; [α]^20^_D_= 15.3 (c = 1.6, CHCl_3_); Colorless oil; HPLC analysis: Chiralcel ID column (hexane / 2-propanol 50:50, 1.0 mL/min, 254 nm); t_r_ (minor) = 13.5 min, t_r_ (major) = 16.5 min. **HRMS (ESI) m/z**: calcd for C_23_H_21_FNO_2_P [M+H]^+^ 394.1372, found 394.1365.

(*S*)-**3g** was synthesized from the **2g**; 99% yield, 70% ee; [α]^20^_D_= -11.9 (c = 1.7, CHCl_3_); Colorless oil; HPLC analysis: Chiralcel ID column (hexane / 2-propanol 50:50, 1.0 mL/min, 254 nm); t_r_ (major) = 13.3 min, t_r_ (minor) = 16.7 min. **HRMS (ESI) m/z**: calcd for C_23_H_21_FNO_2_P [M+H]^+^ 394.1372, found 394.1368.

**3-((diphenylphosphoryl)methyl)-1,3,5-trimethylindolin-2-one （3h）**

**^1^H NMR** (600 MHz, Chloroform-*d*) δ 7.59 – 7.44 (m, 4H), 7.44 – 7.36 (m, 2H), 7.38 – 7.27 (m, 4H), 6.96 – 6.90 (m, 1H), 6.79 – 6.71 (m, 1H), 6.58 (d, *J* = 7.9 Hz, 1H), 3.09 (dd, *J* = 15.1, 9.7 Hz, 1H), 3.05 (s, 3H), 2.81 (dd, *J* = 15.1, 9.6 Hz, 1H), 2.06 (s, 3H), 1.41 (d, *J* = 1.9 Hz, 3H).

**^13^C NMR** (151 MHz, Chloroform-*d*) δ 179.47 (d, *J* = 4.0 Hz), 140.87, 133.82 (d, *J* = 42.8 Hz), 133.16 (d, *J* = 42.5 Hz), 131.44 (d, *J* = 2.7 Hz), 131.39, 131.27 (d, *J* = 2.7 Hz), 131.12 (d, *J* = 2.7 Hz), 130.67 (d, *J* = 9.4 Hz), 130.53 (d, *J* = 9.2 Hz), 128.30, 128.27 (d, *J* = 6.6 Hz), 128.20 (d, *J* = 7.0 Hz), 125.60, 107.64, 45.47 (d, *J* = 3.8 Hz), 37.58 (d, *J* = 71.6 Hz), 26.78 (d, *J* = 12.2 Hz), 26.47, 20.91.

**^31^P NMR** (243 MHz, Chloroform-*d*) δ 25.99.

(*R*)-**3h** was synthesized from the **1h**; 93% yield, 82% ee; [α]^20^_D_= 38.3 (c = 1.5, CHCl_3_); Colorless oil; HPLC analysis: Chiralcel ID column (hexane / 2-propanol 50:50, 1.0 mL/min, 254 nm); t_r_ (minor) = 14.8 min, t_r_ (major) = 19.8 min. **HRMS (ESI) m/z**: calcd for C_24_H_24_NO_2_P [M+H]^+^ 390.1623, found 390.1613.

(*S*)-**3h** was synthesized from the **2h**; 98% yield, 89% ee; [α]^20^_D_= -39.5 (c = 1.4, CHCl_3_); Colorless oil; HPLC analysis: Chiralcel ID column (hexane / 2-propanol 50:50, 1.0 mL/min, 254 nm); t_r_ (major) = 14.4 min, t_r_ (minor) = 20.0 min. **HRMS (ESI) m/z**: calcd for C_24_H_24_NO_2_P [M+H]^+^ 390.1623, found 390.1616.

**3-((diphenylphosphoryl)methyl)-5-fluoro-1,3-dimethylindolin-2-one (3i)**

**^1^H NMR** (600 MHz, Chloroform-*d*) δ 7.58 – 7.48 (m, 4H), 7.47 – 7.40 (m, 2H), 7.35 (tt, *J* = 7.4, 3.3 Hz, 4H), 6.84 (td, *J* = 8.8, 2.6 Hz, 1H), 6.77 (dd, *J* = 8.1, 2.6 Hz, 1H), 6.61 (dd, *J* = 8.5, 4.1 Hz, 1H), 3.08 (dd, *J* = 15.2, 9.4 Hz, 1H), 3.06 (s, 3H), 2.81 (dd, *J* = 15.2, 9.3 Hz, 1H), 1.41 (d, *J* = 1.8 Hz, 3H).

**^13^C NMR** (151 MHz, Chloroform-*d*) δ 179.17 (d, *J* = 4.2 Hz), 158.83 (d, *J* = 240.3 Hz), 139.12 (d, *J* = 1.9 Hz), 133.48 (d, *J* = 35.5 Hz), 133.00 (dd, *J* = 8.3, 2.9 Hz), 132.82 (d, *J* = 35.4 Hz), 131.64 (d, *J* = 2.7 Hz), 131.50 (d, *J* = 2.9 Hz), 130.64 (d, *J* = 9.3 Hz), 130.47 (d, *J* = 9.2 Hz), 128.45 (d, *J* = 12.1 Hz), 128.37 (d, *J* = 12.1 Hz), 114.31 (d, *J* = 23.5 Hz), 112.91 (d, *J* = 25.1 Hz), 108.27 (d, *J* = 8.2 Hz), 45.92 (d, *J* = 4.5 Hz), 37.49 (d, *J* = 71.4 Hz), 26.65, 26.58.

**^31^P NMR** (243 MHz, Chloroform-*d*) δ 25.97.

**^19^F NMR** (565 MHz, Chloroform-*d*) δ -121.19.

(*R*)-**3i** was synthesized from the **1i**; 93% yield, 82% ee; [α]^20^_D_= 0.5 (c = 1.5, CHCl_3_); Colorless oil; HPLC analysis: Chiralcel IB column (hexane / 2-propanol 50:50, 1.0 mL/min, 270 nm); t_r_ (major) = 5.7 min, t_r_ (minor) = 7.0 min. **HRMS (ESI) m/z**: calcd for C_23_H_21_FNO_2_P [M+H]^+^ 394.1372, found 394.1364.

(*S*)-**3i** was synthesized from the **2i**; 98% yield, 89% ee; [α]^20^_D_= -3.0 (c = 1.5, CHCl_3_); Colorless oil; HPLC analysis: Chiralcel IB column (hexane / 2-propanol 50:50, 1.0 mL/min, 270 nm); t_r_ (minor) = 5.9 min, t_r_ (major) = 6.9 min. **HRMS (ESI) m/z**: calcd for C_23_H_21_FNO_2_P [M+H]^+^ 394.1372, found 394.1367.

**5-chloro-3-((diphenylphosphoryl)methyl)-1,3-dimethylindolin-2-one (3j)**

**H NMR** (400 MHz, Chloroform-*d*) δ 7.66 – 7.53 (m, 2H), 7.51 – 7.41 (m, 4H), 7.41 – 7.30 (m, 4H), 7.10 (dd, *J* = 8.3, 2.1 Hz, 1H), 6.82 (d, *J* = 2.1 Hz, 1H), 6.66 (d, *J* = 8.3 Hz, 1H), 3.12 (s, 3H), 3.09 (dd, *J* = 15.1, 8.6 Hz, 1H), 2.79 (dd, *J* = 15.1, 8.4 Hz, 1H), 1.42 (d, *J* = 2.0 Hz, 3H).

**^13^C NMR** (101 MHz, Chloroform-*d*) δ 179.08 (d, *J* = 3.6 Hz), 141.98, 133.54 (d, *J* = 39.5 Hz), 132.81 (d, *J* = 2.8 Hz), 132.56 (d, *J* = 39.5 Hz), 131.71 (d, *J* = 2.8 Hz), 131.63 (d, *J* = 2.8 Hz), 130.56 (d, *J* = 9.4 Hz), 130.37 (d, *J* = 9.4 Hz), 128.48 (d, *J* = 5.6 Hz), 128.37 (d, *J* = 5.7 Hz), 128.04, 127.31, 125.07, 108.88, 45.59 (d, *J* = 4.0 Hz), 37.66 (d, *J* = 71.4 Hz), 26.64 (d, *J* = 12.3 Hz), 26.64.

**^31^P NMR** (162 MHz, Chloroform-*d*) δ 25.82.

(*R*)-**3j** was synthesized from the **1j**; 99% yield, 82% ee; [α]^20^_D_= 38.6 (c = 1.9, CHCl_3_); Colorless oil; HPLC analysis: Chiralcel AD-H column (hexane / 2-propanol 80:20, 1.0 mL/min, 254 nm); t_r_ (major) = 12.8 min, t_r_ (minor) = 15.2 min. **HRMS (ESI) m/z**: calcd for C_23_H_21_ClNO_2_P [M+H]^+^ 410.1077, found 410.1070.

(*S*)-**3j** was synthesized from the **2j**; 99% yield, 54% ee; [α]^20^_D_= -29.4 (c = 1.6, CHCl_3_); Colorless oil; HPLC analysis: Chiralcel AD-H column (hexane / 2-propanol 80:20, 1.0 mL/min, 254 nm); t_r_ (minor) = 12.9 min, t_r_ (major) = 15.2 min. **HRMS (ESI) m/z**: calcd for C_23_H_21_ClNO_2_P [M+H]^+^ 410.1077, found 410.1071.

**(*S*)-3-((diphenylphosphoryl)methyl)-1,3,7-trimethylindolin-2-one (3k)**

**^1^H NMR** (600 MHz, Chloroform-*d*) δ 7.60 (ddd, *J* = 11.4, 8.2, 1.4 Hz, 2H), 7.50 – 7.40 (m, 4H), 7.35 (dtd, *J* = 21.1, 7.7, 2.8 Hz, 4H), 7.05 – 6.96 (m, 1H), 6.90 (d, *J* = 7.7 Hz, 1H), 6.71 (t, *J* = 7.5 Hz, 1H), 3.26 (s, 3H), 3.15 (dd, *J* = 15.3, 9.7 Hz, 1H), 2.84 (dd, *J* = 15.3, 11.6 Hz, 1H), 2.47 (s, 3H), 1.42 (d, *J* = 2.0 Hz, 3H).

**^13^C NMR** (151 MHz, Chloroform-*d*) δ 180.17 (d, *J* = 3.3 Hz), 140.75, 133.74 (d, *J* = 99.5 Hz), 132.77 (d, *J* = 98.6 Hz), 131.97 (d, *J* = 2.8 Hz), 131.72, 131.31 (d, *J* = 2.9 Hz), 131.26 (d, *J* = 2.9 Hz), 130.94 (d, *J* = 9.6 Hz), 130.55 (d, *J* = 9.2 Hz), 128.39 (d, *J* = 11.9 Hz), 128.04 (d, *J* = 11.9 Hz), 122.71, 122.16, 119.35, 44.80 (d, *J* = 3.8 Hz), 37.95 (d, *J* = 71.2 Hz), 29.71, 27.53 (d, *J* = 12.5 Hz), 18.97.

**^31^P NMR** (243 MHz, Chloroform-*d*) δ 26.03.

(*S*)-**3k** was synthesized from the **2k**; 99% yield, 94% ee; [α]^20^_D_= -18.4 (c = 1.5, CHCl_3_); Colorless oil; HPLC analysis: Chiralcel ID column (hexane / 2-propanol 50:50, 1.0 mL/min, 254 nm); t_r_ (major) = 13.7 min, t_r_ (minor) = 15.9 min. **HRMS (ESI) m/z**: calcd for C_24_H_24_NO_2_P [M+H]^+^ 390.1623, found 390.1614.

**(*S*)-diethyl ((1,3,7-trimethyl-2-oxoindolin-3-yl)methyl)phosphonate (3l)**

**^1^H NMR** (400 MHz, Chloroform-*d*) δ 7.19 – 7.12 (m, 1H), 7.07 – 6.86 (m, 2H), 3.88 – 3.73 (m, 3H), 3.69 – 3.57 (m, 1H), 3.51 (s, 3H), 2.57 (s, 3H), 2.48 – 2.26 (m, 2H), 1.38 (d, *J* = 2.9 Hz, 3H), 1.10 (td, *J* = 7.1, 4.3 Hz, 6H).

**^13^C NMR** (101 MHz, Chloroform-*d*) δ 180.11 (d, *J* = 4.7 Hz), 140.97, 132.93 (d, *J* = 2.5 Hz), 131.86, 122.17, 121.73, 119.69, 61.70 (d, *J* = 6.5 Hz), 61.32 (d, *J* = 6.5 Hz), 44.09 (d, *J* = 3.6 Hz), 33.88 (d, *J* = 143.3 Hz), 29.82, 26.71 (d, *J* = 17.5 Hz), 19.12, 16.22 (d, *J* = 6.3 Hz), 16.13 (d, *J* = 6.5 Hz).

**^31^P NMR** (162 MHz, Chloroform-*d*) δ 26.09.

(*S*)-**3l** was synthesized from the **2k**; 78% yield, 84% ee; [α]^20^_D_= -0.8 (c = 1.1, CHCl_3_); Colorless oil; HPLC analysis: Chiralcel OD-H column (hexane / 2-propanol 80:20, 1.0 mL/min, 254 nm); t_r_ (major) = 13.7 min, t_r_ (minor) = 15.9 min. **HRMS (ESI) m/z**: calcd for C_16_H_24_NO_4_P [M+H]^+^ 326.1521, found 326.1513.

**(*S*)-3-((di-p-tolylphosphoryl)methyl)-1,3,7-trimethylindolin-2-one (3m)**

**^1^H NMR** (600 MHz, Chloroform-*d*) δ 7.50 – 7.42 (m, 2H), 7.32 – 7.26 (m, 2H), 7.15 (dd, *J* = 8.1, 2.5 Hz, 2H), 7.10 (dd, *J* = 8.1, 2.5 Hz, 2H), 7.05 (d, *J* = 7.4 Hz, 1H), 6.88 (d, *J* = 7.7 Hz, 1H), 6.72 (t, *J* = 7.5 Hz, 1H), 3.20 (s, 3H), 3.07 (dd, *J* = 15.2, 9.5 Hz, 1H), 2.77 (dd, *J* = 15.3, 12.0 Hz, 1H), 2.43 (s, 3H), 2.34 (s, 3H), 2.32 (s, 3H), 1.37 (d, *J* = 1.9 Hz, 3H).

**^13^C NMR** (151 MHz, Chloroform-*d*) δ 180.23 (d, *J* = 3.3 Hz), 141.57 (d, *J* = 2.7 Hz), 141.52 (d, *J* = 2.8 Hz), 140.67, 132.19 (d, *J* = 2.8 Hz), 131.58, 130.99 (d, *J* = 9.8 Hz), 130.69 (d, *J* = 101.5 Hz), 130.57 (d, *J* = 9.3 Hz), 129.63 (d, *J* = 100.8 Hz), 129.10 (d, *J* = 12.0 Hz), 128.69 (d, *J* = 12.3 Hz), 122.83, 122.12, 119.24, 44.85 (d, *J* = 3.6 Hz), 38.03 (d, *J* = 71.0 Hz), 29.64, 27.56 (d, *J* = 12.4 Hz), 21.48 (d, *J* = 3.0 Hz), 18.95.

**^31^P NMR** (243 MHz, Chloroform-*d*) δ 26.43.

(*S*)-**3m** was synthesized from the **2k**; 95% yield, 94% ee; [α]^20^_D_= -28.2 (c = 1.6, CHCl_3_); Colorless oil; HPLC analysis: Chiralcel IA column (hexane / 2-propanol 80:20, 1.0 mL/min, 254 nm); t_r_ (major) = 18.4 min, t_r_ (minor) = 22.2 min. **HRMS (ESI) m/z**: calcd for C_26_H_28_NO_2_P [M+H]^+^ 418.1936, found 418.1931.

**(*S*)-3-((bis(4-methoxyphenyl)phosphoryl)methyl)-1,3,7-trimethylindolin-2-one (3n)**

**^1^H NMR** (400 MHz, Chloroform-*d*) δ 7.57 – 7.46 (m, 2H), 7.32 – 7.26 (m, 2H), 7.09 – 6.99 (m, 1H), 6.90 – 6.69 (m, 6H), 3.80 (s, 3H), 3.78 (s, 3H), 3.21 (s, 3H), 3.07 (dd, *J* = 15.2, 9.4 Hz, 1H), 2.75 (dd, *J* = 15.3, 12.4 Hz, 1H), 2.43 (s, 3H), 1.36 (d, *J* = 2.1 Hz, 3H).

**^13^C NMR** (101 MHz, Chloroform-*d*) δ 180.24 (d, *J* = 3.2 Hz), 161.95 (d, *J* = 2.9 Hz), 161.90 (d, *J* = 2.9 Hz), 140.62, 132.90 (d, *J* = 10.9 Hz), 132.39 (d, *J* = 10.5 Hz), 132.16 (d, *J* = 2.9 Hz), 131.63, 125.02 (d, *J* = 106.2 Hz), 123.85 (d, *J* = 105.1 Hz), 122.72, 122.18, 119.32, 113.96 (d, *J* = 12.7 Hz), 113.48 (d, *J* = 12.8 Hz), 55.31 (d, *J* = 1.6 Hz), 44.86 (d, *J* = 3.8 Hz), 38.16 (d, *J* = 71.9 Hz), 29.69, 27.80 (d, *J* = 12.7 Hz), 18.97.

**^31^P NMR** (162 MHz, Chloroform-*d*) δ 26.83.

(*S*)-**3n** was synthesized from the **2k**; 98% yield, 92% ee; [α]^20^_D_= -34.3 (c = 1.9, CHCl_3_); Colorless oil; HPLC analysis: Chiralcel AD-H column (hexane / 2-propanol 80:20, 1.0 mL/min, 254 nm); t_r_ (minor) = 26.4 min, t_r_ (major) = 29.6 min. **HRMS (ESI) m/z**: calcd for C_26_H_28_NO_4_P [M+H]^+^ 450.1834, found 450.1827.

**(*S*)-3-((di([1,1'-biphenyl]-4-yl)phosphoryl)methyl)-1,3,7-trimethylindolin-2-one (3o)**

**^1^H NMR** (400 MHz, Chloroform-*d*) δ 7.73 – 7.66 (m, 2H), 7.64 – 7.50 (m, 10H), 7.49 – 7.43 (m, 4H), 7.42 – 7.34 (m, 2H), 7.10 – 6.98 (m, 1H), 6.91 (d, *J* = 7.6 Hz, 1H), 6.73 (t, *J* = 7.5 Hz, 1H), 3.28 – 3.20 (m, 4H), 2.90 (dd, *J* = 15.2, 12.2 Hz, 1H), 2.41 (s, 3H), 1.44 (d, *J* = 1.9 Hz, 3H).

**^13^C NMR** (101 MHz, Chloroform-*d*) δ 180.16 (d, *J* = 2.7 Hz), 144.15 (d, *J* = 2.6 Hz), 144.10 (d, *J* = 2.8 Hz), 140.73, 139.91 (d, *J* = 20.5 Hz), 132.14 (d, *J* = 100.4 Hz), 131.92 (d, *J* = 2.8 Hz), 131.69 (d, *J* = 9.9 Hz), 131.54, 131.11 (d, *J* = 9.5 Hz), 130.97 (d, *J* = 99.7 Hz), 129.01 (d, *J* = 6.9 Hz), 128.16 (d, *J* = 8.3 Hz), 127.19 (d, *J* = 12.5 Hz), 127.17 (d, *J* = 12.0 Hz), 126.66 (d, *J* = 12.0 Hz), 122.78, 122.23, 119.43, 44.84 (d, *J* = 3.6 Hz), 38.22 (d, *J* = 71.4 Hz), 29.72, 27.81 (d, *J* = 12.8 Hz), 19.04.

**^31^P NMR** (162 MHz, Chloroform-*d*) δ 26.56.

(*S*)-**3o** was synthesized from the **2k**; 99% yield, 94% ee; [α]^20^_D_= -27.5 (c = 2.3, CHCl_3_); Colorless oil; HPLC analysis: Chiralcel IA column (hexane / 2-propanol 70:30, 1.0 mL/min, 254 nm); t_r_ (major) = 15.5 min, t_r_ (minor) = 22.0 min. **HRMS (ESI) m/z**: calcd for C_36_H_32_NO_2_P [M+H]^+^ 542.2240, found 542.2249.

**(*S*)-3-((bis(4-(dimethylamino)phenyl)phosphoryl)methyl)-1,3,7-trimethylindolin-2-one (3p)**

**^1^H NMR** (400 MHz, Chloroform-*d*) δ 7.49 – 7.42 (m, 2H), 7.24 – 7.08 (m, 3H), 6.92 – 6.85 (m, 1H), 6.84 – 6.77 (m, 1H), 6.66 – 6.59 (m, 2H), 6.55 – 6.44 (m, 2H), 3.16 (s, 3H), 3.05 – 3.00 (m, 1H), 2.95 (d, *J* = 7.8 Hz, 12H), 2.78 – 2.61 (m, 1H), 2.42 (s, 3H), 1.35 (d, *J* = 1.9 Hz, 3H).

**^13^C NMR** (101 MHz, Chloroform-*d*) δ 180.49 (d, *J* = 3.4 Hz), 151.97 (d, *J* = 2.4 Hz), 151.83 (d, *J* = 2.3 Hz), 140.56, 132.64 (d, *J* = 2.9 Hz), 132.52 (d, *J* = 11.0 Hz), 131.94 (d, *J* = 10.3 Hz), 131.44, 123.07, 122.18, 119.25 (d, *J* = 110.4 Hz), 119.07, 117.75 (d, *J* = 109.4 Hz), 111.36 (d, *J* = 12.4 Hz), 110.84 (d, *J* = 12.6 Hz), 45.00 (d, *J* = 3.6 Hz), 40.06, 38.20 (d, *J* = 71.9 Hz), 29.65, 27.88 (d, *J* = 12.4 Hz), 19.02.

**^31^P NMR** (243 MHz, Chloroform-*d*) δ 28.74.

(*S*)-**3p** was synthesized from the **2k**; 86% yield, 90% ee; [α]^20^_D_= -59.6 (c = 1.9, CHCl_3_); Colorless oil; HPLC analysis: Chiralcel AD-H column (hexane / 2-propanol 70:30, 1.0 mL/min, 254 nm); t_r_ (minor) = 27.2 min, t_r_ (major) = 38.4 min. **HRMS (ESI) m/z**: calcd for C_28_H_34_N_3_O_2_P [M+H]^+^ 476.2467, found 476.2460.

**(*S*)-3-((bis(4-(tert-butyl)phenyl)phosphoryl)methyl)-1,3,7-trimethylindolin-2-one (3q)**

**^1^H NMR** (400 MHz, Chloroform-*d*) δ 7.53 – 7.47 (m, 2H), 7.42 – 7.28 (m, 6H), 6.98 (dd, *J* = 7.4, 1.3 Hz, 1H), 6.86 (d, *J* = 7.6 Hz, 1H), 6.66 (t, *J* = 7.5 Hz, 1H), 3.19 (s, 3H), 3.07 (dd, *J* = 15.2, 9.5 Hz, 1H), 2.78 (dd, *J* = 15.2, 11.8 Hz, 1H), 2.43 (s, 3H), 1.38 (d, *J* = 1.9 Hz, 3H), 1.28 (d, *J* = 6.4 Hz, 18H).

**^13^C NMR** (101 MHz, Chloroform-*d*) δ 180.24 (d, *J* = 3.2 Hz), 154.58 (d, *J* = 2.9 Hz), 154.54 (d, *J* = 3.0 Hz), 140.68, 131.95 (d, *J* = 2.9 Hz), 131.59, 130.88 (d, *J* = 9.8 Hz), 130.40 (d, *J* = 9.4 Hz), 130.36 (d, *J* = 101.6 Hz), 129.36 (d, *J* = 100.0 Hz), 125.36 (d, *J* = 11.9 Hz), 125.06 (d, *J* = 12.0 Hz), 122.83, 122.07, 119.19, 44.77 (d, *J* = 3.7 Hz), 38.26 (d, *J* = 71.1 Hz), 34.89 (d, *J* = 2.8 Hz), 31.12 (d, *J* = 1.7 Hz), 29.64, 27.50 (d, *J* = 12.6 Hz), 19.01.

**^31^P NMR** (162 MHz, Chloroform-*d*) δ 26.12.

(*S*)-**3q** was synthesized from the **2k**; 98% yield, 93% ee; [α]^20^_D_= -15.2 (c = 2.4, CHCl_3_); Colorless oil; HPLC analysis: Chiralcel AD-H column (hexane / 2-propanol 80:20, 1.0 mL/min, 254 nm); t_r_ (major) = 9.8 min, t_r_ (minor) = 12.1 min. **HRMS (ESI) m/z**: calcd for C_32_H_40_NO_2_P [M+H]^+^ 502.2875, found 502.2865.

**(*S*)-3-((bis(4-fluorophenyl)phosphoryl)methyl)-1,3,7-trimethylindolin-2-one (3r)**

**^1^H NMR** (400 MHz, Chloroform-*d*) δ 7.59 – 7.48 (m, 2H), 7.47 – 7.36 (m, 2H), 7.02 (dtd, *J* = 13.3, 8.7, 2.2 Hz, 4H), 6.95 – 6.84 (m, 2H), 6.71 (t, *J* = 7.6 Hz, 1H), 3.26 (s, 3H), 3.13 (dd, *J* = 15.2, 9.8 Hz, 1H), 2.78 (dd, *J* = 15.2, 11.7 Hz, 1H), 2.46 (s, 3H), 1.39 (d, *J* = 2.2 Hz, 3H).

**^13^C NMR** (101 MHz, Chloroform-*d*) δ 179.99 (d, *J* = 2.9 Hz), 165.99 (d, *J* = 3.4 Hz), 163.48 (d, *J* = 3.4 Hz), 140.70, 133.46 (dd, *J* = 11.0, 8.8 Hz), 132.99 (dd, *J* = 10.7, 8.8 Hz), 131.87, 131.69 (d, *J* = 2.9 Hz), 129.25 (dd, *J* = 91.7, 3.4 Hz), 128.24 (dd, *J* = 90.7, 3.4 Hz), 122.43, 122.22, 119.58, 115.85 (dd, *J* = 21.4, 12.9 Hz), 115.45 (dd, *J* = 21.3, 13.0 Hz), 44.74 (d, *J* = 3.7 Hz), 38.15 (d, *J* = 72.3 Hz), 29.75, 27.76 (d, *J* = 13.1 Hz), 18.94.

**^31^P NMR** (162 MHz, Chloroform-*d*) δ 25.60.

**^19^F NMR** (376 MHz, Chloroform-*d*) δ -107.14 (d, *J* = 1.7 Hz), -107.37 (d, *J* = 1.8 Hz).

(*S*)-**3r** was synthesized from the **2k**; 70% yield, 94% ee; [α]^20^_D_= -20.5 (c = 1.2, CHCl_3_); Colorless oil; HPLC analysis: Chiralcel ID column (hexane / 2-propanol 70:30, 1.0 mL/min, 254 nm); t_r_ (major) = 14.4 min, t_r_ (minor) = 17.1 min. **HRMS (ESI) m/z**: calcd for C_24_H_22_F_2_NO_2_P [M+H]^+^ 426.1434, found 426.1425.

**(*S*)-3-((di-m-tolylphosphoryl)methyl)-1,3,7-trimethylindolin-2-one (3s)**

**^1^H NMR** (400 MHz, Chloroform-*d*) δ 7.40 – 7.29 (m, 2H), 7.29 – 7.17 (m, 6H), 7.00 – 6.92 (m, 1H), 6.87 (d, *J* = 7.6 Hz, 1H), 6.66 (t, *J* = 7.6 Hz, 1H), 3.25 (s, 3H), 3.12 (dd, *J* = 15.2, 9.8 Hz, 1H), 2.79 (dd, *J* = 15.2, 11.6 Hz, 1H), 2.44 (s, 3H), 2.27 (d, *J* = 8.6 Hz, 6H), 1.38 (d, *J* = 2.0 Hz, 3H).

**^13^C NMR** (101 MHz, Chloroform-*d*) δ 180.24 (d, *J* = 2.9 Hz), 140.81, 138.20 (d, *J* = 11.6 Hz), 137.95 (d, *J* = 11.5 Hz), 133.97, 132.99, 132.12 (d, *J* = 2.8 Hz), 132.05 (d, *J* = 2.8 Hz), 131.94 (d, *J* = 2.8 Hz), 131.56 (d, *J* = 9.3 Hz), 131.52, 131.25 (d, *J* = 8.7 Hz), 128.18 (d, *J* = 12.5 Hz), 127.89 (d, *J* = 6.9 Hz), 127.78 (d, *J* = 4.4 Hz), 127.38 (d, *J* = 9.8 Hz), 122.75, 121.99, 119.22, 44.75 (d, *J* = 3.7 Hz), 38.09 (d, *J* = 70.9 Hz), 29.69, 27.58 (d, *J* = 12.7 Hz), 21.34 (d, *J* = 5.8 Hz), 18.98.

**^31^P NMR** (162 MHz, Chloroform-*d*) δ 26.52.

(*S*)-**3s** was synthesized from the **2k**; 70% yield, 94% ee; [α]^20^_D_= -15.4 (c = 1.6, CHCl_3_); Colorless oil; HPLC analysis: Chiralcel IA column (hexane / 2-propanol 90:10, 1.0 mL/min, 254 nm); t_r_ (major) = 15.6 min, t_r_ (minor) = 19.8 min. **HRMS (ESI) m/z**: calcd for C_26_H_28_NO_2_P [M+H]^+^ 418.1936, found 418.1927.

**(*S*)-3-((bis(3-methoxyphenyl)phosphoryl)methyl)-1,3,7-trimethylindolin-2-one (3t)**

**^1^H NMR** (400 MHz, Chloroform-*d*) δ 7.29 – 7.22 (m, 2H), 7.17 – 7.00 (m, 3H), 6.99 – 6.89 (m, 4H), 6.90 – 6.83 (m, 1H), 6.72 – 6.61 (t, *J* = 7.5 Hz, 1H), 3.72 (d, *J* = 9.3 Hz, 6H), 3.26 (s, 3H), 3.14 (dd, *J* = 15.2, 9.8 Hz, 1H), 2.80 (dd, *J* = 15.2, 11.8 Hz, 1H), 2.44 (s, 3H), 1.38 (d, *J* = 2.1 Hz, 3H).

**^13^C NMR** (101 MHz, Chloroform-*d*) δ 180.15 (d, *J* = 2.7 Hz), 159.46 (d, *J* = 14.3 Hz), 159.18 (d, *J* = 14.3 Hz), 140.87, 135.04 (d, *J* = 98.7 Hz), 133.83 (d, *J* = 97.8 Hz), 131.88 (d, *J* = 3.0 Hz), 131.70, 129.54 (d, *J* = 14.1 Hz), 129.21 (d, *J* = 14.1 Hz), 122.92 (d, *J* = 10.0 Hz), 122.77, 122.39 (d, *J* = 9.7 Hz), 122.00, 119.25, 118.14 (d, *J* = 2.7 Hz), 117.84 (d, *J* = 2.7 Hz), 115.19 (d, *J* = 4.8 Hz), 115.09 (d, *J* = 5.2 Hz), 55.31 (d, *J* = 3.1 Hz), 44.71 (d, *J* = 3.8 Hz), 38.17 (d, *J* = 71.4 Hz), 29.70, 27.73 (d, *J* = 12.9 Hz), 18.92.

**^31^P NMR** (162 MHz, Chloroform-*d*) δ 26.37.

(*S*)-**3t** was synthesized from the **2k**; 99% yield, 93% ee; [α]^20^_D_= -10.0 (c = 1.9, CHCl_3_); Colorless oil; HPLC analysis: Chiralcel AD-H column (hexane / 2-propanol 90:10, 1.0 mL/min, 254 nm); t_r_ (major) = 10.2 min, t_r_ (minor) = 12.2 min. **HRMS (ESI) m/z**: calcd for C_26_H_28_NO_4_P [M+H]^+^ 450.1834, found 450.1826.

**(*S*)-3-((bis(3,5-dimethylphenyl)phosphoryl)methyl)-1,3,7-trimethylindolin-2-one (3u)**

**^1^H NMR** (400 MHz, Chloroform-*d*) δ 7.18 – 7.09 (m, 2H), 7.09 – 6.99 (m, 4H), 6.99 – 6.93 (m, 1H), 6.91 – 6.84 (d, *J* = 7.6 Hz, 1H), 6.70 – 6.62 (m, 1H), 3.25 (s, 3H), 3.10 (dd, *J* = 15.2, 9.9 Hz, 1H), 2.77 (dd, *J* = 15.2, 11.6 Hz, 1H), 2.45 (s, 3H), 2.25 (d, *J* = 7.6 Hz, 12H), 1.38 (d, *J* = 2.0 Hz, 3H).

**^13^C NMR** (101 MHz, Chloroform-*d*) δ 180.32 (d, *J* = 3.2 Hz), 140.86, 137.93 (d, *J* = 12.4 Hz), 137.69 (d, *J* = 12.4 Hz), 133.45 (d, *J* = 98.8 Hz), 133.07 (d, *J* = 2.9 Hz), 133.01 (d, *J* = 2.9 Hz), 132.42 (d, *J* = 98.0 Hz), 131.98 (d, *J* = 2.9 Hz), 131.53, 128.49 (d, *J* = 9.5 Hz), 128.12 (d, *J* = 9.2 Hz), 122.89, 121.90, 119.11, 44.75 (d, *J* = 3.7 Hz), 38.03 (d, *J* = 70.6 Hz), 30.96, 29.62, 27.54 (d, *J* = 12.6 Hz), 21.26 (d, *J* = 4.6 Hz), 18.97.

**^31^P NMR** (162 MHz, Chloroform-*d*) δ 26.75.

(*S*)-**3u** was synthesized from the **2k**; 99% yield, 92% ee; [α]^20^_D_= -14.9 (c = 1.5, CHCl_3_); Colorless oil; HPLC analysis: Chiralcel OD-H column (hexane / 2-propanol 90:10, 1.0 mL/min, 254 nm); t_r_ (minor) = 7.6 min, t_r_ (major) = 13.9 min. **HRMS (ESI) m/z**: calcd for C_28_H_32_NO_2_P [M+H]^+^ 446.2249, found 446.2243.

**(*S*)-3-((di(naphthalen-2-yl)phosphoryl)methyl)-1,3,7-trimethylindolin-2-one (3v)**

**^1^H NMR** (600 MHz, Chloroform-*d*) δ 8.28 – 8.19 (m, 1H), 8.01 – 7.91 (d, *J* = 13.5 Hz, 1H), 7.86 – 7.81 (m, 3H), 7.80 – 7.73 (m, 3H), 7.70 – 7.65 (m, 1H), 7.56 – 7.44 (m, 5H), 7.12 – 7.06 (m, 1H), 6.77 (d, *J* = 7.7 Hz, 1H), 6.64 (t, *J* = 7.5 Hz, 1H), 3.38 (dd, *J* = 15.4, 8.8 Hz, 1H), 2.96 (dd, *J* = 15.3, 13.1 Hz, 1H), 2.92 (s, 3H), 2.11 (s, 3H), 1.42 (d, *J* = 2.1 Hz, 3H).

**^13^C NMR** (151 MHz, Chloroform-*d*) δ 180.04 (d, *J* = 2.7 Hz), 140.57, 134.44 (d, *J* = 2.2 Hz), 133.22 (d, *J* = 8.3 Hz), 132.71 (d, *J* = 8.1 Hz), 132.53 (d, *J* = 12.8 Hz), 132.24 (d, *J* = 12.8 Hz), 131.89, 131.82 (d, *J* = 3.1 Hz), 130.70 (d, *J* = 99.8 Hz), 129.23 (d, *J* = 98.4 Hz), 129.00 (d, *J* = 20.3 Hz), 128.29 (d, *J* = 11.8 Hz), 128.08 (d, *J* = 16.8 Hz), 127.65 (d, *J* = 22.6 Hz), 127.58, 127.54 (d, *J* = 12.0 Hz), 126.76 (d, *J* = 17.5 Hz), 126.04 (d, *J* = 11.0 Hz), 125.55 (d, *J* = 10.4 Hz), 122.83, 122.09, 119.42, 44.83 (d, *J* = 3.6 Hz), 37.57 (d, *J* = 71.1 Hz), 29.38, 28.11 (d, *J* = 13.0 Hz), 18.63.

**^31^P NMR** (243 MHz, Chloroform-*d*) δ 26.51.

(*S*)-**3v** was synthesized from the **2k**; 99% yield, 93% ee; [α]^20^_D_= -56.1 (c = 2.1, CHCl_3_); Colorless oil; HPLC analysis: Chiralcel IA column (hexane / 2-propanol 80:20, 1.0 mL/min, 254 nm); t_r_ (major) = 21.9 min, t_r_ (minor) = 37.8 min. **HRMS (ESI) m/z**: calcd for C_32_H_28_NO_2_P [M+H]^+^ 490.1936, found 490.1932.

**(*S*)-3-((bis(benzo[d][1,3]dioxol-5-yl)phosphoryl)methyl)-1,3,7-trimethylindolin-2-one (3w)**

**^1^H NMR** (400 MHz, Chloroform-*d*) δ 7.04 (ddd, *J* = 12.1, 8.0, 1.5 Hz, 1H), 7.00 – 6.80 (m, 5H), 6.79 – 6.63 (m, 3H), 6.01 – 5.91 (m, 4H), 3.33 (s, 3H), 3.03 (dd, *J* = 15.1, 10.3 Hz, 1H), 2.72 (dd, *J* = 15.1, 11.1 Hz, 1H), 2.48 (s, 3H), 1.38 (d, *J* = 2.1 Hz, 3H).

**^13^C NMR** (101 MHz, Chloroform-*d*) δ 180.15 (d, *J* = 3.3 Hz), 150.33 (d, *J* = 3.6 Hz), 150.30 (d, *J* = 3.4 Hz), 147.81 (d, *J* = 17.9 Hz), 147.58 (d, *J* = 17.8 Hz), 140.81, 132.06 (d, *J* = 2.8 Hz), 131.72, 126.90 (d, *J* = 58.6 Hz), 126.08 (d, *J* = 10.8 Hz), 125.87 (d, *J* = 57.7 Hz), 125.74 (d, *J* = 10.3 Hz), 122.48, 121.99, 119.44, 110.60 (d, *J* = 12.0 Hz), 110.23 (d, *J* = 11.9 Hz), 108.53 (d, *J* = 14.8 Hz), 108.24 (d, *J* = 15.0 Hz), 101.50 (d, *J* = 1.6 Hz), 44.77 (d, *J* = 3.8 Hz), 38.34 (d, *J* = 72.5 Hz), 29.76, 27.51 (d, *J* = 12.9 Hz), 18.98.

**^31^P NMR** (162 MHz, Chloroform-*d*) δ 27.04.

(*S*)-**3w** was synthesized from the **2k**; 99% yield, 92% ee; [α]^20^_D_= -9.9 (c = 1.8, CHCl_3_); Colorless oil; HPLC analysis: Chiralcel AD-H column (hexane / 2-propanol 80:20, 1.0 mL/min, 254 nm); t_r_ (major) = 29.3 min, t_r_ (minor) = 40.5 min. **HRMS (ESI) m/z**: calcd for C_26_H_24_NO_6_P [M+H]^+^ 478.1419, found 478.1412.

**(*S*)-3-((di(naphthalen-1-yl)phosphoryl)methyl)-1,3,7-trimethylindolin-2-one (3x)**

**^1^H NMR** (400 MHz, Chloroform-*d*) δ 8.67 – 8.56 (m, 1H), 8.23 (d, *J* = 8.5 Hz, 1H), 7.96 – 7.77 (m, 5H), 7.69 – 7.63 (m, 1H), 7.47 – 7.31 (m, 6H), 6.72 – 6.64 (m, 1H), 6.43 (d, *J* = 7.3 Hz, 1H), 6.30 (t, *J* = 7.5 Hz, 1H), 3.63 (dd, *J* = 14.9, 11.3 Hz, 1H), 3.27 (s, 3H), 3.16 (dd, *J* = 15.0, 8.7 Hz, 1H), 2.43 (s, 3H), 1.43 (d, *J* = 2.1 Hz, 3H).

**^13^C NMR** (101 MHz, Chloroform-*d*) δ 180.12 (d, *J* = 3.2 Hz), 140.91, 133.75 (d, *J* = 8.9 Hz), 133.66 (d, *J* = 8.7 Hz), 133.21 (d, *J* = 8.5 Hz), 132.99 (d, *J* = 2.9 Hz), 132.75 (d, *J* = 9.5 Hz), 132.63 (d, *J* = 3.2 Hz), 131.94 (d, *J* = 10.9 Hz), 131.80 (d, *J* = 2.5 Hz), 131.41, 130.05 (d, *J* = 8.8 Hz), 129.10 (d, *J* = 8.0 Hz), 128.84 (d, *J* = 41.9 Hz), 127.08 (d, *J* = 56.0 Hz), 126.71 (d, *J* = 5.2 Hz), 126.14 (d, *J* = 4.6 Hz), 126.11 (d, *J* = 38.9 Hz), 124.72 (d, *J* = 13.3 Hz), 124.12 (d, *J* = 13.8 Hz), 121.94, 121.60, 119.40, 44.85 (d, *J* = 3.5 Hz), 37.44 (d, *J* = 72.9 Hz), 29.74, 27.82 (d, *J* = 13.1 Hz), 19.03.

**^31^P NMR** (162 MHz, Chloroform-*d*) δ 30.26.

(*S*)-**3x** was synthesized from the **2k**; 70% yield, 94% ee; [α]^20^_D_= -68.0 (c = 1.4, CHCl_3_); Colorless oil; HPLC analysis: Chiralcel IF column (hexane / 2-propanol 80:20, 1.0 mL/min, 254 nm); t_r_ (major) = 35.0 min, t_r_ (minor) = 43.0 min. **HRMS (ESI) m/z**: calcd for C_32_H_28_NO_2_P [M+H]^+^ 490.1936, found 490.1932.

**(*R*)-3-((diphenylphosphoryl)methyl)-5-methoxy-1,3-dimethylindolin-2-one (3aa)**

**^1^H NMR** (600 MHz, Chloroform-*d*) δ 7.58 – 7.47 (m, 4H), 7.41 (qd, *J* = 7.6, 1.5 Hz, 2H), 7.38 – 7.27 (m, 4H), 6.72 (d, *J* = 2.5 Hz, 1H), 6.69 (dd, *J* = 8.4, 2.5 Hz, 1H), 6.58 (d, *J* = 8.4 Hz, 1H), 3.62 (s, 3H), 3.08 (dd, *J* = 15.2, 10.8 Hz, 1H), 3.02 (s, 3H), 2.82 (dd, *J* = 15.2, 10.0 Hz, 1H), 1.41 (d, *J* = 1.8 Hz, 3H).

**^13^C NMR** (151 MHz, Chloroform-*d*) δ 179.20 (d, *J* = 4.3 Hz), 155.54, 136.59, 133.65 (d, *J* = 82.4 Hz), 132.99 (d, *J* = 82.2 Hz), 132.56 (d, *J* = 2.8 Hz), 131.49 (d, *J* = 2.9 Hz), 131.26 (d, *J* = 2.7 Hz), 130.69 (d, *J* = 9.3 Hz), 130.54 (d, *J* = 9.3 Hz), 128.34 (d, *J* = 11.7 Hz), 128.27 (d, *J* = 11.6 Hz), 113.25, 111.46, 108.31, 55.57, 45.96 (d, *J* = 3.9 Hz), 37.46 (d, *J* = 71.4 Hz), 26.81 (d, *J* = 11.9 Hz), 26.50.

**^31^P NMR** (243 MHz, Chloroform-*d*) δ 26.37.

(*R*)-**3aa** was synthesized from the **1aa**; 93% yield, 80% ee; [α]^20^_D_= 48.9 (c = 1.4, CHCl_3_); Colorless oil; HPLC analysis: Chiralcel ID column (hexane / 2-propanol 50:50, 1.0 mL/min, 254 nm); t_r_ (minor) = 19.5 min, t_r_ (major) = 23.6 min. **HRMS (ESI) m/z**: calcd for C_24_H_24_NO_3_P [M+H]^+^ 406.1572, found 406.1563.

**(*R*)-3-((diphenylphosphoryl)methyl)-1,3,5,6-tetramethylindolin-2-one (3ab)**

**^1^H NMR** (400 MHz, Chloroform-*d*) δ 7.57 – 7.27 (m, 10H), 6.69 (s, 1H), 6.51 (s, 1H), 3.07 (s, 4H), 2.81 (dd, *J* = 15.0, 9.4 Hz, 1H), 2.22 (s, 3H), 1.95 (s, 3H), 1.41 (d, *J* = 1.9 Hz, 3H).

**^13^C NMR** (101 MHz, Chloroform-*d*) δ 179.63 (d, *J* = 4.0 Hz), 141.22, 136.24, 133.89 (d, *J* = 9.4 Hz), 132.91 (d, *J* = 8.8 Hz), 131.46 (d, *J* = 2.7 Hz), 131.02 (d, *J* = 2.9 Hz), 130.65 (d, *J* = 9.4 Hz), 130.53 (d, *J* = 9.4 Hz), 129.63, 128.58 (d, *J* = 2.7 Hz), 128.26 (d, *J* = 11.8 Hz), 128.07 (d, *J* = 11.8 Hz), 125.91, 109.41, 45.27 (d, *J* = 3.9 Hz), 37.85 (d, *J* = 72.0 Hz), 26.72 (d, *J* = 12.3 Hz), 26.47, 20.24, 19.31.

**^31^P NMR** (162 MHz, Chloroform-*d*) δ 26.24.

(*R*)-**3ab** was synthesized from the **1ab**; 93% yield, 85% ee; [α]^20^_D_= 25.8 (c = 1.5, CHCl_3_); Colorless oil; HPLC analysis: Chiralcel ID column (hexane / 2-propanol 50:50, 1.0 mL/min, 254 nm); t_r_ (minor) = 13.5 min, t_r_ (major) = 21.9 min. **HRMS (ESI) m/z**: calcd for C_25_H_26_NO_2_P [M+H]^+^ 404.1779, found 404.1770.

**(*R*)-5-(tert-butyl)-3-((diphenylphosphoryl)methyl)-1,3-dimethylindolin-2-one (3ac)**

**^1^H NMR** (400 MHz, Chloroform-*d*) δ 7.66 – 7.54 (m, 2H), 7.45 – 7.32 (m, 7H), 7.30 – 7.26 (m, 2H), 7.22 – 7.15 (m, 1H), 6.56 (d, *J* = 8.2 Hz, 1H), 3.12 (dd, *J* = 15.4, 9.8 Hz, 1H), 2.99 (s, 3H), 2.91 (dd, *J* = 15.3, 11.5 Hz, 1H), 1.44 (d, *J* = 1.8 Hz, 3H), 1.21 (s, 9H).

**^13^C NMR** (101 MHz, Chloroform-*d*) δ 179.83 (d, *J* = 4.3 Hz), 145.37, 140.56, 133.85 (d, *J* = 94.6 Hz), 132.87 (d, *J* = 94.2 Hz), 131.47 (d, *J* = 2.9 Hz), 131.28 (d, *J* = 2.8 Hz), 131.22 (d, *J* = 3.0 Hz), 130.77 (d, *J* = 9.6 Hz), 130.57 (d, *J* = 9.2 Hz), 128.47 (d, *J* = 11.7 Hz), 128.12 (d, *J* = 11.8 Hz), 124.76, 122.13, 107.35, 45.85 (d, *J* = 3.9 Hz), 37.29 (d, *J* = 71.4 Hz), 34.50, 31.57, 27.33 (d, *J* = 11.9 Hz), 26.39.

**^31^P NMR** (162 MHz, Chloroform-*d*) δ 26.82.

(*R*)-**3ac** was synthesized from the **1ac**; 58% yield, 85% ee; [α]^20^_D_= 53.7 (c = 0.7, CHCl_3_); Colorless oil; HPLC analysis: Chiralcel ID column (hexane / 2-propanol 50:50, 1.0 mL/min, 254 nm); t_r_ (minor) = 8.7 min, t_r_ (major) = 10.1 min. **HRMS (ESI) m/z**: calcd for C_27_H_30_NO_2_P [M+H]^+^ 432.2092, found 432.2086.

**3-((di-p-tolylphosphoryl)methyl)-1,3,5-trimethylindolin-2-one (3ba)**

**^1^H NMR** (400 MHz, Chloroform-*d*) δ 7.44 – 7.29 (m, 4H), 7.12 (ddd, *J* = 8.0, 5.3, 2.6 Hz, 4H), 6.95 – 6.89 (m, 1H), 6.75 (d, *J* = 1.7 Hz, 1H), 6.58 (d, *J* = 7.9 Hz, 1H), 3.04 (s, 4H), 2.77 (dd, *J* = 15.1, 9.6 Hz, 1H), 2.33 (d, *J* = 8.6 Hz, 6H), 2.06 (s, 3H), 1.40 (d, *J* = 1.8 Hz, 3H).

**^13^C NMR** (101 MHz, Chloroform-*d*) δ 179.53 (d, *J* = 4.0 Hz), 141.79 (d, *J* = 2.7 Hz), 141.42 (d, *J* = 2.8 Hz), 140.82, 131.38, 131.31 (d, *J* = 2.7 Hz), 130.85, 130.71 (d, *J* = 9.8 Hz), 130.56 (d, *J* = 9.7 Hz), 129.72 (d, *J* = 22.8 Hz), 129.03 (d, *J* = 3.4 Hz), 128.91 (d, *J* = 3.3 Hz), 128.14, 125.62, 107.61, 45.47 (d, *J* = 3.9 Hz), 37.65 (d, *J* = 71.9 Hz), 26.82 (d, *J* = 12.3 Hz), 26.43, 21.48 (d, *J* = 1.5 Hz), 20.85.

**^31^P NMR** (162 MHz, Chloroform-*d*) δ 26.95.

(*R*)-**3ba** was synthesized from the **1h**; 78% yield, 88% ee; [α]^20^_D_= 36.4 (c = 1.3, CHCl_3_); Colorless oil; HPLC analysis: Chiralcel AD-H column (hexane / 2-propanol 80:20, 1.0 mL/min, 254 nm); t_r_ (major) = 24.1 min, t_r_ (minor) = 29.1 min. **HRMS (ESI) m/z**: calcd for C_26_H_28_NO_2_P [M+H]^+^ 418.1936, found 418.1928.

(*S*)-**3ba** was synthesized from the **2h**; 96% yield, 85% ee; [α]^20^_D_= -40.0(c = 1.7, CHCl_3_); Colorless oil; HPLC analysis: Chiralcel AD-H column (hexane / 2-propanol 80:20, 1.0 mL/min, 254 nm); t_r_ (minor) = 24.0 min, t_r_ (major) = 29.2 min. **HRMS (ESI) m/z**: calcd for C_26_H_28_NO_2_P [M+H]^+^ 418.1936, found 418.1926.

**3-((bis(4-methoxyphenyl)phosphoryl)methyl)-1,3,5-trimethylindolin-2-one (3ca)**

**^1^H NMR** (400 MHz, Chloroform-*d*) δ 7.49 – 7.31 (m, 4H), 6.99 – 6.89 (m, 1H), 6.88 – 6.68 (m, 5H), 6.58 (d, *J* = 7.9 Hz, 1H), 3.79 (d, *J* = 8.3 Hz, 6H), 3.10 – 2.97 (m, 4H), 2.72 (dd, *J* = 15.1, 9.9 Hz, 1H), 2.10 (s, 3H), 1.39 (d, *J* = 1.8 Hz, 3H).

**^13^C NMR** (101 MHz, Chloroform-*d*) δ 179.56 (d, *J* = 3.9 Hz), 161.97 (d, *J* = 2.9 Hz), 161.82 (d, *J* = 2.9 Hz), 140.78, 132.58 (d, *J* = 10.8 Hz), 132.36 (d, *J* = 10.6 Hz), 131.45, 131.43, 128.18, 125.56, 125.35 (d, *J* = 24.1 Hz), 124.30 (d, *J* = 23.4 Hz), 113.81 (d, *J* = 12.7 Hz), 113.74 (d, *J* = 12.7 Hz), 107.61, 55.29 (d, *J* = 4.1 Hz), 45.51 (d, *J* = 3.8 Hz), 37.86 (d, *J* = 72.5 Hz), 26.95 (d, *J* = 12.3 Hz), 26.46, 20.90.

**^31^P NMR** (162 MHz, Chloroform-*d*) δ 26.41.

(*R*)-**3ca** was synthesized from the **1h**; 70% yield, 89% ee; [α]^20^_D_= 51.4 (c = 1.2, CHCl_3_); Colorless oil; HPLC analysis: Chiralcel IA column (hexane / 2-propanol 85:15, 1.0 mL/min, 254 nm); t_r_ (major) = 46.7 min, t_r_ (minor) = 53.7 min. **HRMS (ESI) m/z**: calcd for C_26_H_28_NO_4_P [M+H]^+^ 450.1834, found 450.1826.

(*S*)-**3ca** was synthesized from the **2h**; 89% yield, 87% ee; [α]^20^_D_= -50.7 (c = 1.5, CHCl_3_); Colorless oil; HPLC analysis: Chiralcel IA column (hexane / 2-propanol 85:15, 1.0 mL/min, 254 nm); t_r_ (minor) = 43.9 min, t_r_ (major) = 54.7 min. **HRMS (ESI) m/z**: calcd for C_26_H_28_NO_4_P [M+H]^+^ 450.1834, found 450.1826.

**3-((di([1,1'-biphenyl]-4-yl)phosphoryl)methyl)-1,3,5-trimethylindolin-2-one (3da)**

**^1^H NMR** (400 MHz, Chloroform-*d*) δ 7.64 – 7.51 (m, 12H), 7.48 – 7.37 (m, 6H), 6.99 – 6.92 (m, 1H), 6.85 (d, *J* = 1.7 Hz, 1H), 6.59 (d, *J* = 7.9 Hz, 1H), 3.19 (dd, *J* = 15.1, 10.6 Hz, 1H), 3.05 (s, 3H), 2.90 (dd, *J* = 15.1, 10.0 Hz, 1H), 2.05 (s, 3H), 1.46 (d, *J* = 1.9 Hz, 3H).

**^13^C NMR** (101 MHz, Chloroform-*d*) δ 179.45 (d, *J* = 3.5 Hz), 144.27 (d, *J* = 2.7 Hz), 144.00 (d, *J* = 2.8 Hz), 140.89, 139.92, 132.36 (d, *J* = 43.9 Hz), 131.57, 131.53, 131.28 (d, *J* = 9.7 Hz), 131.18 (d, *J* = 2.7 Hz), 131.07 (d, *J* = 9.5 Hz), 128.99, 128.28, 128.13 (d, *J* = 3.4 Hz), 127.21, 127.03 (d, *J* = 3.8 Hz), 126.91 (d, *J* = 3.8 Hz), 125.67, 107.72, 45.49 (d, *J* = 3.7 Hz), 37.83 (d, *J* = 71.9 Hz), 27.03 (d, *J* = 12.5 Hz), 26.50, 21.01.

**^31^P NMR** (162 MHz, Chloroform-*d*) δ 26.29.

(*R*)-**3da** was synthesized from the **1h**; 94% yield, 79% ee; [α]^20^_D_= 21.9 (c = 2.4, CHCl_3_); Colorless oil; HPLC analysis: Chiralcel AD-H column (hexane / 2-propanol 70:30, 1.0 mL/min, 254 nm); t_r_ (major) = 18.1 min, t_r_ (minor) = 25.5 min. **HRMS (ESI) m/z**: calcd for C_36_H_32_NO_2_P [M+H]^+^ 542.2249, found 542.2241.

(*S*)-**3da** was synthesized from the **2h**; 92% yield, 84% ee; [α]^20^_D_= -26.4 (c = 1.9, CHCl_3_); Colorless oil; HPLC analysis: Chiralcel AD-H column (hexane / 2-propanol 70:30, 1.0 mL/min, 254 nm); t_r_ (minor) = 18.0 min, t_r_ (major) = 25.6 min. **HRMS (ESI) m/z**: calcd for C_36_H_32_NO_2_P [M+H]^+^ 542.2249, found 542.2242.

**3-((bis(4-fluorophenyl)phosphoryl)methyl)-1,3,5-trimethylindolin-2-one (3ea)**

**^1^H NMR** (400 MHz, Chloroform-*d*) δ 7.59 – 7.35 (m, 4H), 7.16 – 6.85 (m, 5H), 6.79 – 6.67 (m, 1H), 6.60 (d, *J* = 7.9 Hz, 1H), 3.22 – 2.99 (m, 4H), 2.76 (dd, *J* = 15.1, 9.6 Hz, 1H), 2.10 (s, 3H), 1.41 (d, *J* = 2.1 Hz, 3H).

**^13^C NMR** (101 MHz, Chloroform-*d*) δ 179.24 (d, *J* = 3.5 Hz), 165.98 (dd, *J* = 12.4, 3.2 Hz), 163.47 (dd, *J* = 11.9, 3.2 Hz), 140.84, 133.19 (dd, *J* = 10.9, 8.8 Hz), 132.96 (dd, *J* = 10.8, 8.7 Hz), 131.56, 130.93 (d, *J* = 2.9 Hz), 129.48 (dd, *J* = 25.6, 3.4 Hz), 128.46 (dd, *J* = 25.0, 3.4 Hz), 128.45, 125.30, 115.80 (d, *J* = 13.0 Hz), 115.59 (d, *J* = 13.0 Hz), 107.82, 45.35 (d, *J* = 3.9 Hz), 37.87 (d, *J* = 72.7 Hz), 27.02 (d, *J* = 13.0 Hz), 26.49, 20.85.

**^31^P NMR** (162 MHz, Chloroform-*d*) δ 25.37.

**^19^F NMR** (376 MHz, Chloroform-*d*) δ -106.90 (d, *J* = 1.9 Hz), -107.74 (d, *J* = 1.9 Hz).

(*R*)-**3ea** was synthesized from the **1h**; 94% yield, 80% ee; [α]^20^_D_= 39.3 (c = 1.7, CHCl_3_); Colorless oil; HPLC analysis: Chiralcel ID column (hexane / 2-propanol 70:30, 1.0 mL/min, 254 nm); t_r_ (minor) = 16.1 min, t_r_ (major) = 22.0 min. **HRMS (ESI) m/z**: calcd for C_24_H_22_F_2_NO_2_P [M+H]^+^ 426.1434, found 426.1430.

(*S*)-**3ea** was synthesized from the **2h**; 90% yield, 86% ee; [α]^20^_D_= -37.5 (c = 1.8, CHCl_3_); Colorless oil; HPLC analysis: Chiralcel ID column (hexane / 2-propanol 70:30, 1.0 mL/min, 254 nm); t_r_ (major) = 16.1 min, t_r_ (minor) = 22.5 min. **HRMS (ESI) m/z**: calcd for C_24_H_22_F_2_NO_2_P [M+H]^+^ 426.1434, found 426.1429.

**3-((bis(3-methoxyphenyl)phosphoryl)methyl)-1,3,5-trimethylindolin-2-one (3fa)**

**^1^H NMR** (400 MHz, Chloroform-*d*) δ 7.28 – 7.22 (m, 2H), 7.14 – 6.99 (m, 3H), 6.98 – 6.85 (m, 4H), 6.70 (s, 1H), 6.59 (d, *J* = 7.9 Hz, 1H), 3.71 (d, *J* = 3.6 Hz, 6H), 3.15 – 2.99 (m, 4H), 2.79 (dd, *J* = 15.1, 9.5 Hz, 1H), 2.06 (s, 3H), 1.40 (d, *J* = 2.0 Hz, 3H).

**^13^C NMR** (101 MHz, Chloroform-*d*) δ 179.47 (d, *J* = 3.3 Hz), 159.37 (d, *J* = 7.3 Hz), 159.22 (d, *J* = 7.2 Hz), 141.02, 135.20 (d, *J* = 43.4 Hz), 134.22 (d, *J* = 43.1 Hz), 131.29, 131.20 (d, *J* = 2.9 Hz), 129.48 (d, *J* = 14.1 Hz), 129.34 (d, *J* = 14.3 Hz), 128.29, 125.56, 122.62 (d, *J* = 9.7 Hz), 122.28 (d, *J* = 10.0 Hz), 118.07 (d, *J* = 2.7 Hz), 117.74 (d, *J* = 2.8 Hz), 115.19 (d, *J* = 5.0 Hz), 115.09 (d, *J* = 5.6 Hz), 107.53, 55.33 (d, *J* = 1.4 Hz), 45.36 (d, *J* = 3.7 Hz), 37.84 (d, *J* = 72.0 Hz), 26.99 (d, *J* = 12.7 Hz), 26.49, 20.86.

**^31^P NMR** (162 MHz, Chloroform-*d*) δ 26.28.

(*R*)-**3fa** was synthesized from the **1h**; 78% yield, 85% ee; [α]^20^_D_= 35.6 (c = 1.6, CHCl_3_); Colorless oil; HPLC analysis: Chiralcel AD-H column (hexane / 2-propanol 90:10, 1.0 mL/min, 254 nm); t_r_ (minor) = 28.8 min, t_r_ (major) = 43.6 min. **HRMS (ESI) m/z**: calcd for C_26_H_28_NO_4_P [M+H]^+^ 450.1834, found 450.1829.

(*S*)-**3fa** was synthesized from the **2h**; 98% yield, 84% ee; [α]^20^_D_= -33.2 (c = 2.0, CHCl_3_); Colorless oil; HPLC analysis: Chiralcel AD-H column (hexane / 2-propanol 90:10, 1.0 mL/min, 254 nm); t_r_ (major) = 28.5 min, t_r_ (minor) = 43.6 min. **HRMS (ESI) m/z**: calcd for C_26_H_28_NO_4_P [M+H]^+^ 450.1834, found 450.1832.

**3-((di(naphthalen-2-yl)phosphoryl)methyl)-1,3,5-trimethylindolin-2-one (3ga)**

**^1^H NMR** (400 MHz, Chloroform-*d*) δ 8.16 – 7.96 (m, 2H), 7.87 – 7.73 (m, 6H), 7.61 – 7.46 (m, 6H), 6.82 – 6.70 (m, 1H), 6.66 (s, 1H), 6.52 (d, *J* = 7.9 Hz, 1H), 3.33 (dd, *J* = 15.0, 11.0 Hz, 1H), 3.02 – 2.84 (m, 4H), 1.63 (s, 3H), 1.45 (d, *J* = 1.9 Hz, 3H).

**^13^C NMR** (101 MHz, Chloroform-*d*) δ 179.44 (d, *J* = 3.2 Hz), 140.87, 134.52 (d, *J* = 2.3 Hz), 134.37 (d, *J* = 2.3 Hz), 132.91 (d, *J* = 7.9 Hz), 132.60 (d, *J* = 8.6 Hz), 132.51, 132.37 (d, *J* = 2.1 Hz), 132.24, 131.41, 130.91 (d, *J* = 2.7 Hz), 130.68 (d, *J* = 12.2 Hz), 129.69 (d, *J* = 11.3 Hz), 128.97, 128.45, 128.11 (d, *J* = 11.6 Hz), 128.06 (d, *J* = 12.4 Hz), 127.69 (d, *J* = 3.3 Hz), 126.81 (d, *J* = 2.2 Hz), 125.78 (d, *J* = 10.6 Hz), 125.32 (d, *J* = 5.0 Hz), 125.27 (d, *J* = 6.0 Hz), 107.68, 45.43 (d, *J* = 3.7 Hz), 37.21 (d, *J* = 72.0 Hz), 27.08 (d, *J* = 12.8 Hz), 26.38, 20.33.

**^31^P NMR** (162 MHz, Chloroform-*d*) δ 26.61.

(*R*)-**3ga** was synthesized from the **1h**; 83% yield, 80% ee; [α]^20^_D_= 21.6 (c = 1.8, CHCl_3_); Colorless oil; HPLC analysis: Chiralcel IF column (hexane / 2-propanol 50:50, 1.0 mL/min, 254 nm); t_r_ (minor) = 23.5 min, t_r_ (major) = 28.7 min. **HRMS (ESI) m/z**: calcd for C_32_H_28_NO_2_P [M+H]^+^ 490.1936, found 490.1933.

(*S*)-**3ga** was synthesized from the **2h**; 94% yield, 86% ee; [α]^20^_D_= -22.8 (c = 2.0, CHCl_3_); Colorless oil; HPLC analysis: Chiralcel IF column (hexane / 2-propanol 50:50, 1.0 mL/min, 254 nm); t_r_ (major) = 23.1 min, t_r_ (minor) = 28.9 min. **HRMS (ESI) m/z**: calcd for C_32_H_28_NO_2_P [M+H]^+^ 490.1936, found 490.1932.

**3-((bis(2,3-dihydrobenzo[b][1,4]dioxin-6-yl)phosphoryl)methyl)-1,3,5-trimethylindolin-2-one (3ha)**

**^1^H NMR** (400 MHz, Chloroform-*d*) δ 7.05 – 6.85 (m, 5H), 6.85 – 6.71 (m, 3H), 6.63 (d, *J* = 7.9 Hz, 1H), 4.23 (qd, *J* = 5.8, 3.7 Hz, 8H), 3.13 (s, 3H), 2.97 (dd, *J* = 15.0, 11.6 Hz, 1H), 2.71 (dd, *J* = 15.0, 8.9 Hz, 1H), 2.12 (s, 3H), 1.41 (d, *J* = 1.9 Hz, 3H).

**^13^C NMR** (101 MHz, Chloroform-*d*) δ 179.52 (d, *J* = 4.2 Hz), 146.38 (d, *J* = 2.8 Hz), 146.11 (d, *J* = 2.9 Hz), 143.43 (d, *J* = 7.4 Hz), 143.26 (d, *J* = 7.6 Hz), 140.92, 131.34 (d, *J* = 2.6 Hz), 131.14, 128.16, 126.46 (d, *J* = 15.2 Hz), 125.42 (d, *J* = 15.5 Hz), 125.38, 124.05 (d, *J* = 9.8 Hz), 123.91 (d, *J* = 9.9 Hz), 119.95 (d, *J* = 9.4 Hz), 119.84 (d, *J* = 9.2 Hz), 117.43 (d, *J* = 11.1 Hz), 117.29 (d, *J* = 11.3 Hz), 107.59, 64.50 (d, *J* = 2.9 Hz), 64.13, 45.41 (d, *J* = 3.9 Hz), 37.90 (d, *J* = 72.7 Hz), 26.59 (d, *J* = 12.9 Hz), 26.51, 20.98.

**^31^P NMR** (162 MHz, Chloroform-*d*) δ 25.84.

(*R*)-**3ha** was synthesized from the **1h**; 84% yield, 89% ee; [α]^20^_D_= 10.5 (c = 1.9, CHCl_3_); Colorless oil; HPLC analysis: Chiralcel AD-H column (hexane / 2-propanol 70:30, 1.0 mL/min, 254 nm); t_r_ (minor) = 22.2 min, t_r_ (major) = 40.0 min. **HRMS (ESI) m/z**: calcd for C_28_H_28_NO_6_P [M+H]^+^ 506.1732, found 506.1730.

(*S*)-**3ha** was synthesized from the **2h**; 99% yield, 86% ee; [α]^20^_D_= -11.5 (c = 2.0, CHCl_3_); Colorless oil; HPLC analysis: Chiralcel AD-H column (hexane / 2-propanol 70:30, 1.0 mL/min, 254 nm); t_r_ (major) = 22.0 min, t_r_ (minor) = 40.9 min. **HRMS (ESI) m/z**: calcd for C_28_H_28_NO_6_P [M+H]^+^ 506.1732, found 506.1727.

## 10. NMR spectrum

^1^H NMR spectrum of compound **1ab**

^13^C NMR spectrum of compound **1ab**

^1^H NMR spectrum of compound **2d**

^13^C NMR spectrum of compound **2d**

^19^F NMR spectrum of compound **2d**

^1^H NMR spectrum of compound **2j**

^13^C NMR spectrum of compound **2j**

^19^F NMR spectrum of compound **2j**

^1^H NMR spectrum of compound **3a**

^13^C NMR spectrum of compound **3a**

^31^P NMR spectrum of compound **3a**

^1^H NMR spectrum of compound **3b**

^13^C NMR spectrum of compound **3b**

^31^P NMR spectrum of compound **3b**

^1^H NMR spectrum of compound **3c**

^13^C NMR spectrum of compound **3c**

^31^P NMR spectrum of compound **3c**

^1^H NMR spectrum of compound **3d**

^13^C NMR spectrum of compound **3d**

^31^P NMR spectrum of compound **3d**

^1^H NMR spectrum of compound **3e**

^13^C NMR spectrum of compound **3e**

^31^P NMR spectrum of compound **3e**

^1^H NMR spectrum of compound **3f**

^13^C NMR spectrum of compound **3f**

^31^P NMR spectrum of compound **3f**

^1^H NMR spectrum of compound **3g**

^13^C NMR spectrum of compound **3g**

^31^P NMR spectrum of compound **3g**

^19^F NMR spectrum of compound **3g**

^1^H NMR spectrum of compound **3h**

^13^C NMR spectrum of compound **3h**

^31^P NMR spectrum of compound **3h**

^1^H NMR spectrum of compound **3i**

^13^C NMR spectrum of compound **3i**

^31^P NMR spectrum of compound **3i**

^19^F NMR spectrum of compound **3i**

^1^H NMR spectrum of compound **3j**

^13^C NMR spectrum of compound **3j**

^31^P NMR spectrum of compound **3j**

^1^H NMR spectrum of compound **3k**

^13^C NMR spectrum of compound **3k**

^31^P NMR spectrum of compound **3k**

^1^H NMR spectrum of compound **3l**

^13^C NMR spectrum of compound **3l**

^31^P NMR spectrum of compound **3l**

^1^H NMR spectrum of compound **3m**

^13^C NMR spectrum of compound **3m**

^31^P NMR spectrum of compound **3m**

^1^H NMR spectrum of compound **3n**

^13^C NMR spectrum of compound **3n**

^31^P NMR spectrum of compound **3n**

^1^H NMR spectrum of compound **3o**

^13^C NMR spectrum of compound **3o**

^31^P NMR spectrum of compound **3o**

^1^H NMR spectrum of compound **3p**

^13^C NMR spectrum of compound **3p**

^31^P NMR spectrum of compound **3p**

^1^H NMR spectrum of compound **3q**

^13^C NMR spectrum of compound **3q**

^31^P NMR spectrum of compound **3q**

^1^H NMR spectrum of compound **3r**

^13^C NMR spectrum of compound **3r**

^31^P NMR spectrum of compound **3r**

^19^F NMR spectrum of compound **3r**

^1^H NMR spectrum of compound **3s**

^13^C NMR spectrum of compound **3s**

^31^P NMR spectrum of compound **3s**

^1^H NMR spectrum of compound **3t**

^13^C NMR spectrum of compound **3t**

^31^P NMR spectrum of compound **3t**

^1^H NMR spectrum of compound **3u**

^13^C NMR spectrum of compound **3u**

^31^P NMR spectrum of compound **3u**

^1^H NMR spectrum of compound **3v**

^13^C NMR spectrum of compound **3v**

^31^P NMR spectrum of compound **3v**

^1^H NMR spectrum of compound **3w**

^13^C NMR spectrum of compound **3w**

^31^P NMR spectrum of compound **3w**

^1^H NMR spectrum of compound **3x**

^13^C NMR spectrum of compound **3x**

^31^P NMR spectrum of compound **3x**

^1^H NMR spectrum of compound **3aa**

^13^C NMR spectrum of compound **3aa**

^31^P NMR spectrum of compound **3aa**

^1^H NMR spectrum of compound **3ab**

^13^C NMR spectrum of compound **3ab**

^31^P NMR spectrum of compound **3ab**

^1^H NMR spectrum of compound **3ac**

^13^C NMR spectrum of compound **3ac**

^31^P NMR spectrum of compound **3ac**

^1^H NMR spectrum of compound **3ba**

^13^C NMR spectrum of compound **3ba**

^31^P NMR spectrum of compound **3ba**

^1^H NMR spectrum of compound **3ca**

^13^C NMR spectrum of compound **3ca**

^31^P NMR spectrum of compound **3ca**

^1^H NMR spectrum of compound **3da**

^13^C NMR spectrum of compound **3da**

^31^P NMR spectrum of compound **3da**

^1^H NMR spectrum of compound **3ea**

^13^C NMR spectrum of compound **3ea**

^31^P NMR spectrum of compound **3ea**

^19^F NMR spectrum of compound **3ea**

^1^H NMR spectrum of compound **3fa**

^13^C NMR spectrum of compound **3fa**

^31^P NMR spectrum of compound **3fa**

^1^H NMR spectrum of compound **3ga**

^13^C NMR spectrum of compound **3ga**

^31^P NMR spectrum of compound **3ga**

^1^H NMR spectrum of compound **3ha**

^13^C NMR spectrum of compound **3ha**

^31^P NMR spectrum of compound **3ha**

^1^H NMR spectrum of compound **4**

^13^C NMR spectrum of compound **4**

^31^P NMR spectrum of compound **4**

^1^H NMR spectrum of compound **5**

^13^C NMR spectrum of compound **5**

^31^P NMR spectrum of compound **5**

^1^H NMR spectrum of compound **6**

^13^C NMR spectrum of compound **6**

^31^P NMR spectrum of compound **6**

^1^H NMR spectrum of compound **7**

^13^C NMR spectrum of compound **7**

^31^P NMR spectrum of compound **7**

^1^H NMR spectrum of compound **8**

^13^C NMR spectrum of compound **8**

^31^P NMR spectrum of compound **8**

## 11. HPLC spectrum

**3-((diphenylphosphoryl)methyl)-1,3-dimethylindolin-2-one (3a)**

(*R*)-**3a**

(*S*)-**3a**

**3-((diphenylphosphoryl)methyl)-1,3,6-trimethylindolin-2-one (3b)**

(*R*)-**3b**

(*S*)-**3b**

**3-((diphenylphosphoryl)methyl)-6-methoxy-1,3-dimethylindolin-2-one (3c)**

**(*S*)-3-((diphenylphosphoryl)methyl)-1,3-dimethyl-6-(tert-pentyl)indolin-2-one (3d)**

**(*S*)-6-(tert-butyl)-3-((diphenylphosphoryl)methyl)-1,3-dimethylindolin-2-one (3e)**

**(*S*)-3-((diphenylphosphoryl)methyl)-1,3-dimethyl-6-phenylindolin-2-one (3f)**

**3-((diphenylphosphoryl)methyl)-6-fluoro-1,3-dimethylindolin-2-one (3g)**

(*R*)-**3g**

(*S*)-**3g**

**3-((diphenylphosphoryl)methyl)-1,3,5-trimethylindolin-2-one （3h）**

(*R*)-**3h**

(*S*)-**3h**

**3-((diphenylphosphoryl)methyl)-5-fluoro-1,3-dimethylindolin-2-one (3i)**

(*R*)-**3i**

(*S*)-**3i**

**5-chloro-3-((diphenylphosphoryl)methyl)-1,3-dimethylindolin-2-one (3j)**

(*R*)-**3j**

(*S*)-**3j**

**(*S*)-3-((diphenylphosphoryl)methyl)-1,3,7-trimethylindolin-2-one (3k)**

**(*S*)-diethyl ((1,3,7-trimethyl-2-oxoindolin-3-yl)methyl)phosphonate (3l)**

**(*S*)-3-((di-p-tolylphosphoryl)methyl)-1,3,7-trimethylindolin-2-one (3m)**

**(*S*)-3-((bis(4-methoxyphenyl)phosphoryl)methyl)-1,3,7-trimethylindolin-2-one (3n)**

**(*S*)-3-((di([1,1'-biphenyl]-4-yl)phosphoryl)methyl)-1,3,7-trimethylindolin-2-one (3o)**

**(*S*)-3-((bis(4-(dimethylamino)phenyl)phosphoryl)methyl)-1,3,7-trimethylindolin-2-one (3p)**

**(*S*)-3-((bis(4-(tert-butyl)phenyl)phosphoryl)methyl)-1,3,7-trimethylindolin-2-one (3q)**

**(*S*)-3-((bis(4-fluorophenyl)phosphoryl)methyl)-1,3,7-trimethylindolin-2-one (3r)**

**(*S*)-3-((di-m-tolylphosphoryl)methyl)-1,3,7-trimethylindolin-2-one (3s)**

**(*S*)-3-((bis(3-methoxyphenyl)phosphoryl)methyl)-1,3,7-trimethylindolin-2-one (3t)**

**(*S*)-3-((bis(3,5-dimethylphenyl)phosphoryl)methyl)-1,3,7-trimethylindolin-2-one (3u)**

**(*S*)-3-((di(naphthalen-2-yl)phosphoryl)methyl)-1,3,7-trimethylindolin-2-one (3v)**

**(*S*)-3-((bis(benzo[d][1,3]dioxol-5-yl)phosphoryl)methyl)-1,3,7-trimethylindolin-2-one (3w)**

**(*S*)-3-((di(naphthalen-1-yl)phosphoryl)methyl)-1,3,7-trimethylindolin-2-one (3x)**

**(*R*)-3-((diphenylphosphoryl)methyl)-5-methoxy-1,3-dimethylindolin-2-one (3aa)**

**(*R*)-3-((diphenylphosphoryl)methyl)-1,3,5,6-tetramethylindolin-2-one (3ab)**

**(*R*)-5-(tert-butyl)-3-((diphenylphosphoryl)methyl)-1,3-dimethylindolin-2-one (3ac)**

**3-((di-p-tolylphosphoryl)methyl)-1,3,5-trimethylindolin-2-one (3ba)**

(*R*)-**3ba**

(*S*)-**3ba**

\

**3-((bis(4-methoxyphenyl)phosphoryl)methyl)-1,3,5-trimethylindolin-2-one (3ca)**

(*R*)-**3ca**

(*S*)-**3ca**

**3-((di([1,1'-biphenyl]-4-yl)phosphoryl)methyl)-1,3,5-trimethylindolin-2-one (3da)**

(*R*)-**3da**

(*S*)-**3da**

**3-((bis(4-fluorophenyl)phosphoryl)methyl)-1,3,5-trimethylindolin-2-one (3ea)**

(*R*)-**3ea**

(*S*)-**3ea**

**3-((bis(3-methoxyphenyl)phosphoryl)methyl)-1,3,5-trimethylindolin-2-one (3fa)**

(*R*)-**3fa**

(*S*)-**3fa**

**3-((di(naphthalen-2-yl)phosphoryl)methyl)-1,3,5-trimethylindolin-2-one (3ga)**

(*R*)-**3ga**

(*S*)-**3ga**

**3-((bis(2,3-dihydrobenzo[b][1,4]dioxin-6-yl)phosphoryl)methyl)-1,3,5-trimethylindolin-2-one (3ha)**

(*R*)-**3ha**

(*S*)-**3ha**

**(*S*)-diphenyl((1,3,7-trimethylindolin-3-yl)methyl)phosphine oxide (4)**

**(*S*)-3-((diphenylphosphorothioyl)methyl)-1,3,7-trimethylindoline-2-thione (5)**

**(*S*)-5-bromo-3-((diphenylphosphoryl)methyl)-1,3,7-trimethylindolin-2-one (6)**

**(*S*)-3-((diphenylphosphoryl)methyl)-1,3,7-trimethyl-5-(phenylethynyl)indolin-2-one (7)**

**(*S*)-5-(benzo[d][1,3]dioxol-5-yl)-3-((diphenylphosphoryl)methyl)-1,3,7-trimethylindolin-2-one (8)**
